# Cascading continental-scale floods across Europe in 1342–1343

**DOI:** 10.1038/s41586-026-10888-8

**Published:** 2026-08-12

**Authors:** Andrea Kiss, Alberto Viglione, Mariano Barriendos, Silvia Enzi, Martin Bauch, Kris Decker, Nicolas Maughan, Dag Retsö, Astrid Ogilvie, Miriam Bertola, Tim Soens, Libor Elleder, Rudolf Brázdil, Kathleen Pribyl, Juraj Parajka, Ioannis Telelis, Inês Amorim, Josep Barriendos, Oliver Böhm, Gerardo Benito, Chiara Bertolin, Dario Camuffo, Denis Coeur, Gaston Demarée, Radoslaw Doktor, Markus Dotterweich, Rüdiger Glaser, Jürgen Komma, Neil Macdonald, Hrvoje Petrić, Christian Rohr, Petra Schmocker-Fackel, Lothar Schulte, Willem H. J. Toonen, Oliver Wetter, Günter Blöschl

**Affiliations:** 1 Institute of Hydraulic Engineering and Water Resources Management, Vienna University of Technology, Vienna, Austria; 2 Department of Environment, Land and Infrastructure Engineering, Politecnico di Torino, Turin, Italy; 3 Institute of Environmental Assessment and Water Research (IDAEA-CSIC), Barcelona, Spain; 4 National Research Council of Italy, Institute of Atmospheric Sciences and Climate (CNR-ISAC), Padova, Italy; 5 Leibniz Institute for the History and Culture of Eastern Europe (GWZO), Leipzig, Germany; 6 Department of Cultural and Science Studies, University of Lucerne, Lucerne, Switzerland; 7 Environmental Sciences Department, Aix-Marseille University, Marseille, France; 8 Department of Economic History and International Relations, Stockholm University, Stockholm, Sweden; 9 Stefansson Arctic Institute, Akureyri, Iceland; 10 Institute of Arctic and Alpine Research (INSTAAR), University of Colorado, Boulder, CO USA; 11 Centre for Urban History and the Urban Studies Institute, University of Antwerp, Antwerp, Belgium; 12 Czech Hydrometeorological Institute, Prague, Czech Republic; 13 Institute of Geography, Masaryk University, Brno, Czech Republic; 14 Global Change Research Institute, Czech Academy of Sciences, Brno, Czech Republic; 15 Climate Research Unit, University of East Anglia, Norwich, UK; 16 Research Center for Greek and Latin Literature, Academy of Athens, Athens, Greece; 17 Department of History and of Political and International Studies (CITCEM), University of Porto, Porto, Portugal; 18 Department of Geography, Autonomous University of Barcelona, Barcelona, Spain; 19 Institute of Geography, University of Augsburg, Augsburg, Germany; 20 National Museum of Natural Sciences, Spanish Research Council (CSIC), Madrid, Spain; 21 Norwegian University of Science and Technology, Trondheim, Norway; 22 ACTHYS-Diffusion, Grenoble, France; 23 Meteorological Research and Development Department, Royal Meteorological Institute of Belgium, Brussels, Belgium; 24 Centre for Flood and Drought Modelling, Institute of Meteorology and Water Management – National Research Institute, Warsaw, Poland; 25 GEOarch – Applied Geoarchaeology, Landau, Germany; 26 Institute of Physical Geography, University of Freiburg, Freiburg, Germany; 27 Department of Geography and Planning, School of Environmental Sciences, University of Liverpool, Liverpool, UK; 28 Department of History, Faculty of Humanities and Social Sciences, University of Zagreb, Zagreb, Croatia; 29 Institute of History, University of Bern, Bern, Switzerland; 30 Oeschger Centre for Climate Change Research, University of Bern, Bern, Switzerland; 31 Hydrology Division, Federal Office for the Environment (FOEN), Bern, Switzerland; 32 FluvAlps-PaleoRisk Research Group, Department of Geography, University of Barcelona, Barcelona, Spain; 33 Earth and Climate Cluster, Vrije Universiteit Amsterdam, Amsterdam, The Netherlands; 34 Present Address: Institute of History, University of Bern, Bern, Switzerland

**Keywords:** Hydrology, Natural hazards

## Abstract

Europe has experienced extreme floods in recent decades. However, even larger floods are possible and must be considered in flood risk management^1,2^. Their characteristics can be clarified by analysing the largest documented historical floods. In Central Europe, the Magdalena Flood of July 1342 is usually considered the largest of the last millennium; however, knowledge of its characteristics is incomplete^3–7^. Here we show that 16 major flood events occurred across much of Europe between late 1341 and 1343. Four of these events had return periods of 500–1,000 years (the Magdalena, Bartholomew, Candlemas and Jacob Floods). Although Magdalena was thought previously to be the only extreme European flood in 1342^3,7^, our new documentary dataset suggests that it formed part of a broader sequence. The year with the greatest number of extreme floods during the past 700 years was 1342, and 1343 ranks among the top ten. This highly unusual sequence of floods had substantial socio-economic impacts, including a paradigm shift in flood mitigation measures in Europe. A series of volcanic eruptions along with multi-annual Arctic sea ice retreat is a plausible cause of this flood sequence. Clusters of extreme floods occurring within a few months are rarely considered in risk management^8^. Quick and proactive risk strategies are needed that account for this eventuality.

## Abstract (web summary)

Tracing cascading continental-scale medieval megafloods across Europe indicates that current flood management is unprepared for such sequences, highlighting the urgent need for proactive flood risk management strategies that account for unusual series of extreme flood events.

## Main

Numerous major floods have occurred in recent decades^1,8^, and their magnitude and frequency are increasing in many parts of Europe^2^. Defence against the impacts of such events include flood management measures, such as the building of levees and the implementation of land use planning. These measures are generally based on knowledge of events in the immediate past. However, it is possible that even larger floods than those in the recent times may occur, some of them in close sequence. The ability to evaluate the possibilities and consequences of such events will be enhanced by the analysis of flood events in the more distant past.

## The Magdalena Flood in Europe

In Central Europe, the Magdalena Flood is usually considered to have been the largest flood in the last millennium, and the most severe single erosion event during the Holocene^3^. The flood peaked in the central part of the Holy Roman Empire (Germany) in late July 1342. Based on documentary records and soil erosion patterns, the return period of the driving precipitation was estimated as 10,000 years^4^. It was probably caused by an extensive Vb weather pattern, that is, one that transports atmospheric moisture from the Mediterranean into Central Europe^5^. Flood levels of the Rhine and Main rivers had return periods of between 100 and 1,000 years^6^. The losses were enormous, with thousands of fatalities, most bridges and the settlements along the main rivers destroyed and the agricultural damage caused dearth^7^. In Prague, a catastrophic flood occurred in February 1342^9^ and, in the Carpathian Basin, the greatest annual number of floods of the Middle Ages were recorded in 1342^10^.

However, so far, the exact magnitude, spatial extent, frequency and impact of the European floods during 1342 and 1343 remain unclear. The analysis presented here encompasses a new dataset on floods, weather extremes and socio-economic consequences collected from more than 1,000 original source entries and natural proxy evidence, to infer the flood characteristics in Europe during 1342–1343.

## Flood and meteorological data

The data are based on the collation of published and manuscript chronicles, annals, economic and legal administrative records, a weather diary, scientific essays and private and official correspondence (Supplementary Data 1–3). To ensure reliability of the data, we use almost exclusively contemporary documentation written during or shortly after 1342–1343. Up until now, most of these data have not been used in flood and weather reconstructions.

Flood magnitudes are quantified by a standard three-scaled index of notable, great and outstanding floods^11^ based on direct indicators, such as the level and spatial extent of flood waters relative to identifiable landmarks and, to a lesser extent, indirect indicators such as environmental or socio-economic impacts. For air temperature, precipitation and socio-economic consequences we use indices based on analogous indicators. Furthermore, the uncertainty (Supplementary Data 1 and 2) of the indices is quantified. These results are then compared with a historical flood dataset^11^, extended in quantity and to cover the period 1300–2020 (Supplementary Data 4; Methods), and to instrumental flood data from 1960–2020^1,2^.

**Fig. 1:**
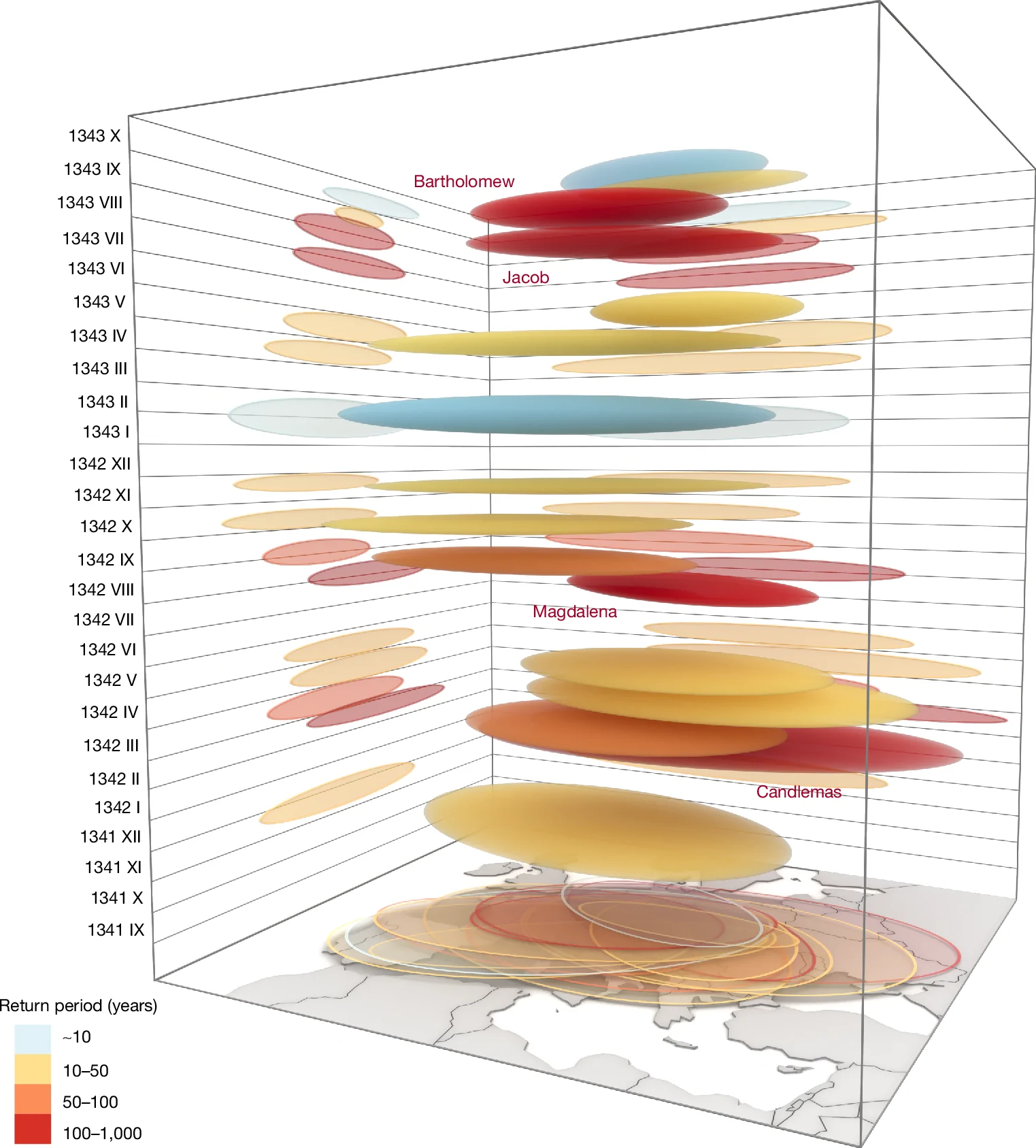
Conceptual reconstruction of the 1342–1343 floods in Europe. Sixteen events are rendered as ellipsoids based on historical evidence on floods (161 data points) and meteorological conditions (more than 217 data points), and on modern analogue events for spatial extent (Supplementary Data 1, 2, 4 and 5; Methods). Colours represent the severity of the events. For a dynamic visualization, see Supplementary Video 1.

**Extended Data Fig. 1:**
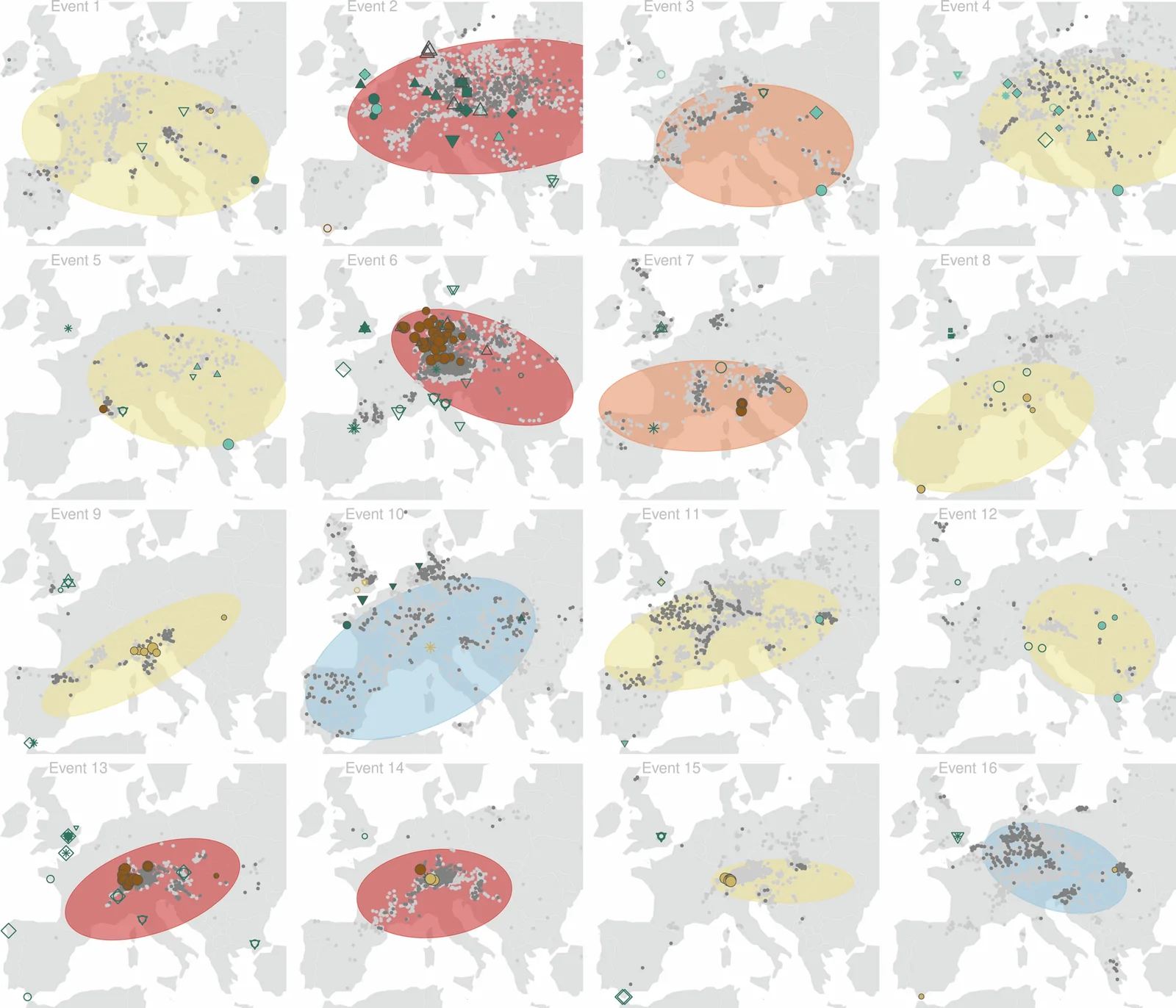
Conceptual reconstruction of the 1342–1343 floods in Europe. 16 events rendered as ellipses based on historical evidence on floods (161 data points) and meteorological conditions (>217 data points), and on modern analogue events for spatial extent (see Methods section, Supplementary Data 1, 2, 4, 6).

Here we find that the year 1342 had the greatest number of great and outstanding floods reported in the past 700 years in Europe. The year 1343 was also an outstanding flood year. Within these two years, 16 major flood events occurred in Europe (Fig. 1, Extended Data Fig. 1 and Extended Data Tables 1 and 2), and four of these had return periods of 500–1,000 years. This finding contrasts with the previous understanding of only one extreme flood in this period.

## String of pearls: the 1342–1343 floods

**Fig. 2:**
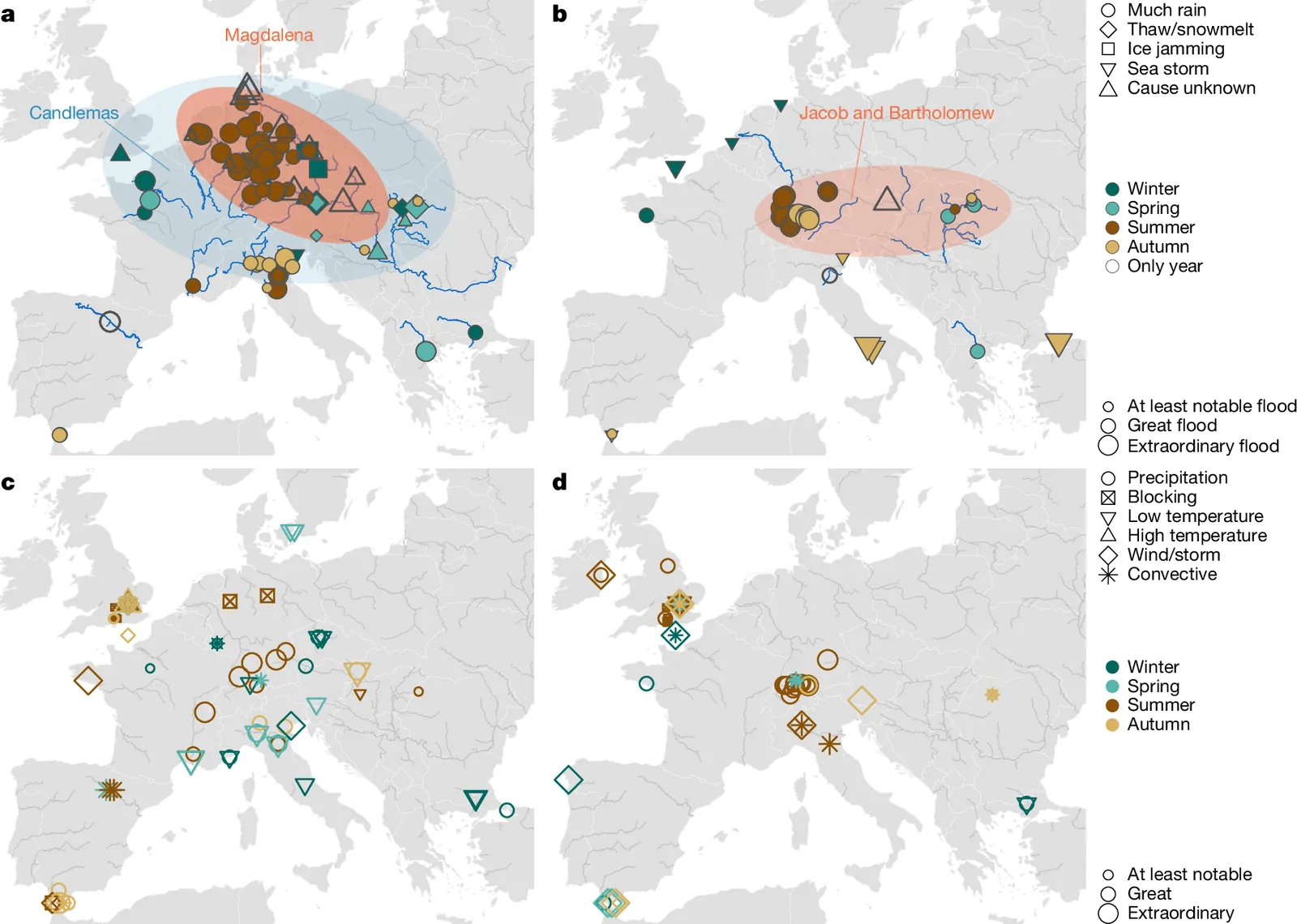
Spatiotemporal distribution of floods and weather information in 1342 and 1343. **a**,**b**, Locations of reported flooding and the affected principal rivers. Ellipses, spatial coverage of the four main floods indicated by documentary evidence in 1342 (**a**) and 1343 (**b**). **c**,**d**, Locations of reported weather events in 1342 (**c**) and 1343 (**d**). Symbol type, flood causes and weather phenomena; colours, seasons; symbol size, event intensity. For details, see Supplementary Data 1a and 2a.

The autumn of 1341 was wet, with great mid-November and December floods in East Central and Southeast Europe (Figs. 1 and 2a,c, Extended Data Tables 1 and 2 and Supplementary Data 1 and 2). The subsequent cold and snowy winter and sudden thaw caused an outstanding flood in mid-February 1342 in Western and Central Europe (approximate return periods of *T* = 200–1,000 years) in Prague and along the Danube. We term this event the ‘Candlemas Flood’ after the Christian feast on 2 February (Julian calendar) mentioned by the sources. After three spring floods in Central, Western and Southeast Europe (*T* = 50–100 years) the extraordinary Magdalena Flood hit Southern Germany, in particular the Main catchment, in late July, traditionally on the day of Saint Maria Magdalena, with estimated flood return periods of 1,000–10,000 years^6^. This event was followed by a great late summer flood in Northern Italy (*T* = 50–100 years) and two autumn floods in the Mediterranean and the Carpathian Basin (*T* = 20–100 years).

The wet January and February of 1343 caused flooding in Western and Central Europe, followed by a Europe-wide wet spring with floods in Southeast Spain and the Carpathians (*T* = 10–100 years), and prolonged flooding in Northern Italy, the Carpathians and in the Balkans (Figs. 1 and 2b,d, Extended Data Tables 1 and 2 and Supplementary Data 1 and 2). In late July an outstanding flood (termed here the Jacob Flood; *T* = 100–1,000 years) affected primarily the northern and central Alps and the Carpathians, and in late August an even bigger flood (termed here the Bartholomew Flood; *T* = 200–1,000 years) affected the same area. In mid-September, a third event hit the tributaries of Lake Constance, and floods continued in the Carpathians in October (*T* = 10–100 years).

## Short-term and long-term consequences

**Fig. 3:**
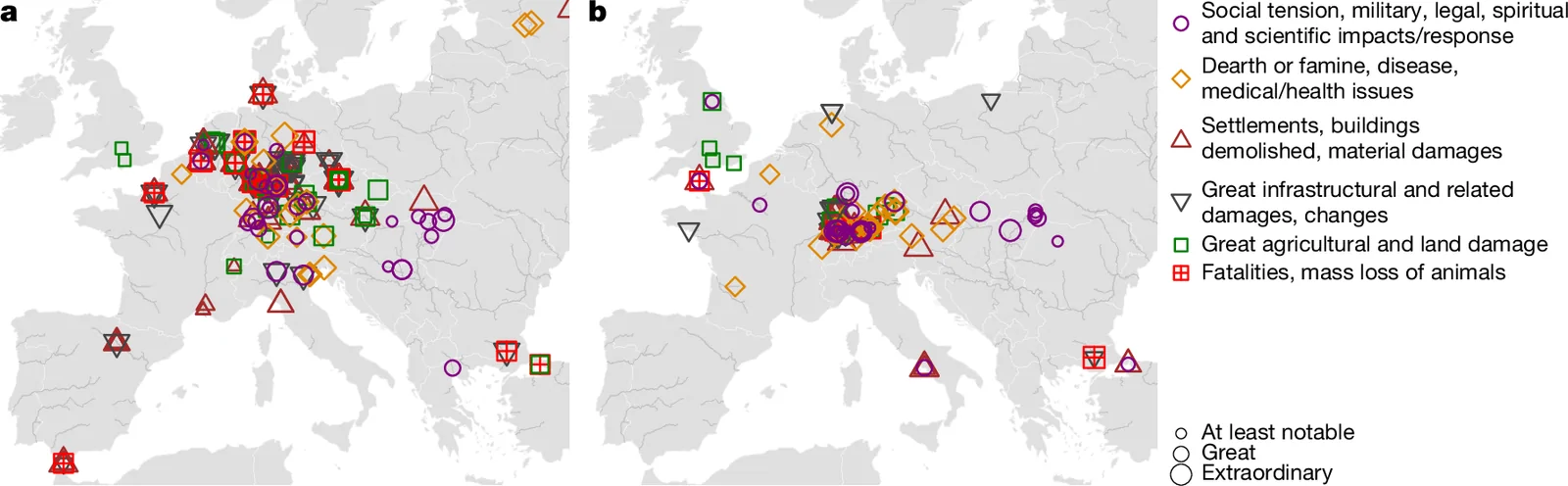
Socio-economic impacts of floods in 1342 and 1343. **a**,**b**, Data documented in 1342 (**a**) and 1343 (**b**). Symbol type, main group of impact type; symbol size, intensity of impact. For details, see Extended Data Table 4 and Supplementary Data 3.

The 1342–1343 floods caused many thousands of direct fatalities in Europe and many more indirect fatalities caused by food shortages and disease (Fig. 3). Thus, for example, the Franciscan friar in Lindau, Johann von Winterthur, reported a two miles extension of the Candlemas Flood along the Danube and some 6,000 deaths: “ut fertur, et quod de hominibus VI milia absorbuit” (Supplementary Data 3). Concurrently, Venice was also inundated heavily. The Magdalena Flood destroyed most settlements and all bridges^7^, roads and mills along the Middle Rhine, Main and its tributaries. The Jacob and Bartholomew Floods had the same effect along the Rhine from Schaffhausen to Basel, and so had the Candlemas Flood, especially along the Elbe and its tributaries. Even the iconic stone bridge across the Vltava in Prague, standing since 1172, was swept away. In fact, during these 2 years the bridges across the main European rivers were damaged, and roads on a continental scale were disrupted, which had a fundamental effect on European transportation and travel capabilities (Fig. 3 and Extended Data Table 3).

The repetitive, widespread and long-lasting flooding in these densely populated areas provoked fear and a feeling of emergency among the European population, given that relatively few floods had occurred in the previous decades (Supplementary Data 4)^12,13^. According to Johann von Winterthur, Paris astronomers suggested that this very unusual sequence of flooding happens only once every 500 years and attributed it to a specific stellar constellation^14^. According to the annalists in Stift Neuberg, Austria, the year 1342 seemed as if the ‘four elements had been disturbed all around the world’ (“In toto universo in quatuor elementis diversis temporibus fuit confusio et disturbium”) when ‘floods caused numerous great losses’ (“per inundacionem aquarum diversa dispensia provenerunt”)^13^. These several floods caused the abandonment of village sites such as Stollhofen (today part of Traismauer) on the Danube in Lower Austria—a decision taken only rarely after a single flood (Supplementary Data 3).

## Flood mitigation, dearth and banking crisis

These several floods also produced a paradigm shift in flood mitigation strategies across Europe. More robust bridges were made partly or entirely of stone (for example, in Prague, Dresden, Säckingen), stronger town walls were designed for flood protection (for example, in Strasbourg), river sections regulated and substantial dykes (for example, Danube at Oberaltaich, the River Nogat/Vistula near Gdansk) and riverside fortifications amenable to flood warning (for example, in Säckingen) were constructed (Extended Data Table 2, Fig. 3, Supplementary Data 3 and Supplementary Information sections 1 and 2). For example, the redesign of the Charles Bridge in Prague resulted in a more massive structure, with broader and higher arches, sharply pointed ice-breaking piers, and riverbanks elevated by up to 9 m—design choices that address directly the impacts of floodwater, ice and debris experienced in 1342 (Supplementary Information section 1).

During 1342–1344, lost harvests and transportation problems resulted in dearth and famine in much of Europe, particularly in Southern Germany, Switzerland and Austria, and food prices soared in Western and Central Europe (Fig. 3 and Supplementary Information section 3). Based on merchants’ reports, the abbot of Saint-Martin de Tournai even suggested a ‘worldwide’ (that is, Europe-wide) dearth in 1342^13^. Very unusual salt production problems in Italy in 1342–1343, presumably caused by flooding of salt pans, led to imports from locations as far away as Cyprus, as evidenced by contemporary accounts noting that salt was produced everywhere only in ‘small quantities’ because of the ‘weather conditions’ (“cum isto anno [1342] propter condicionem temporis sal factus sit in modica quantitate in omni parte”; Supplementary Information section 3). Disruption of transportation routes hindered military operations (for example, siege of Algeciras, Byzantine civil war; Supplementary Data 1, 3) and, apart from other historical reasons^14^, could also be a contributing factor to the economic turmoil and escalation of the great crisis in the Italian banking system from 1343^15^ (Fig. 3, Supplementary Data 3 and Supplementary Information section 3).

In extensive areas, the flood waters did not recede between events, causing major multi-annual health problems. Thus, for example, in late 1344, the Hungarian archbishop and royal physician László Kaboli, living in Kalocsa by the Danube, died a few months after complaining to the pope about the ‘unhealthy waters’ (“quod terra, in qua Colocensis ecclesia situata existit, paludibus et diversi aquis immundis et fetidis circumdata”)^16^. Water-borne diseases^17^ such as the mortal ‘bleeding illness’ (presumably dysentery or another bloody diarrhoea) were reported in 1342–1343 in the flood- and famine-affected regions and beyond (Johann von Winterthur; Supplementary Data 3 and Supplementary Information section 3).

## Most unusual flood biennium in 700 years

**Fig. 4:**
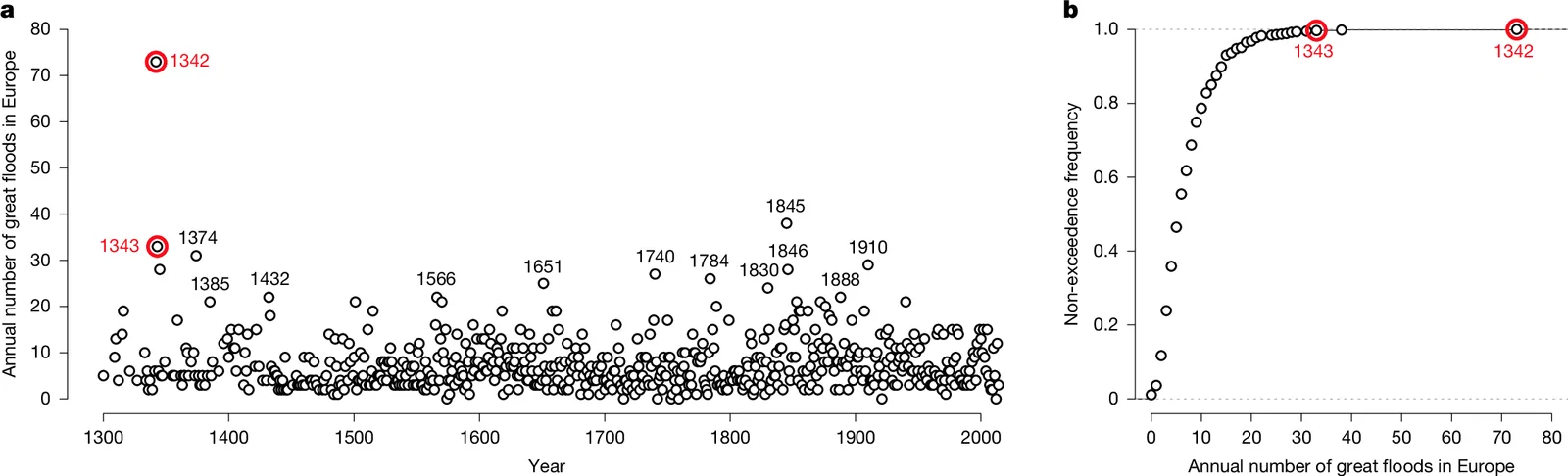
Annual frequency of great and extraordinary floods in Europe in the last 700 years. **a**, Time series. **b**, Associated frequency plot. Homogenized time series with bias correction of the sum of class 2 and 3 floods. In the context of the past 700 years, 1342 stands out as the year with the largest number of great and extraordinary floods in Europe. For similar plots without bias correction, see Extended Data Fig. 1. For details, see Supplementary Data 4.

In context of the past 700 years, 1342 stands out as the year with the largest number of great and extraordinary floods in Europe, and 1343 is the third most important (Fig. 4). Although the major twentieth and twenty-first century floods happened more or less randomly in time, the fourteenth century floods were clustered in 1342–1343. The 16 flood events across Europe are reflected in 121 individual class 2 and 3 floods (Fig. 4). Some rivers were affected particularly often during these two years. The Main, Rhine, Danube and Tisza flooded at least three times in 1342, and the Rhine, Bodensee and Tisza at least two or three times in 1343 (Extended Data Table 1). The flood sequence of 1342–1343 is a model example of a more frequent than expected occurrence of mega-events^1,8^.

## Analogues: 1342–1343 and recent floods

**Fig. 5:**
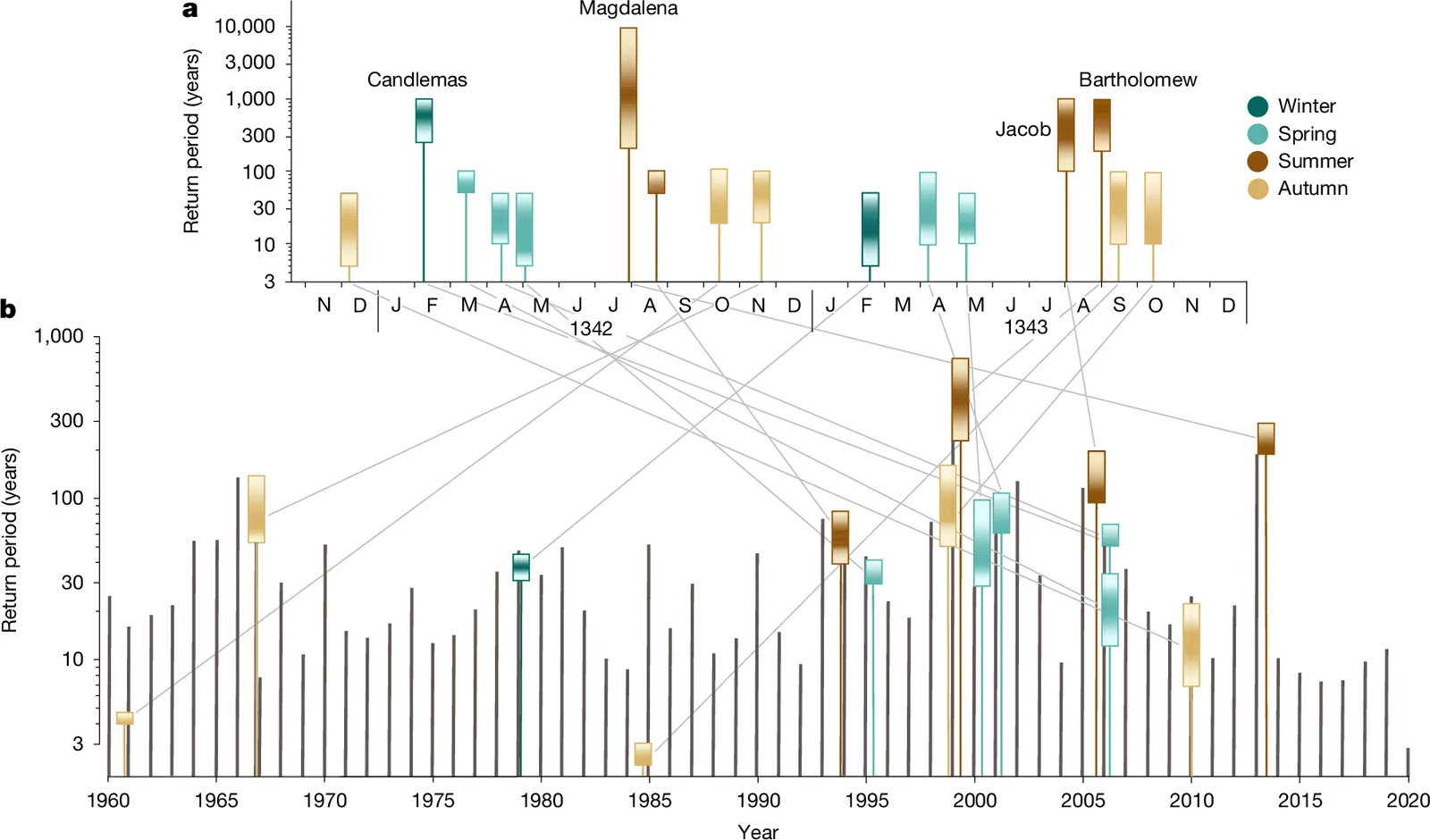
Closest-match analogues in the last six decades to the 16 European floods in 1341–1343. **a**, The largest known return periods of the 16 events in 1342–1343. Coloured boxes indicate the uncertainty ranges of the estimates with darker colours representing the most probable return periods. Letters on the x axis represent months of the year. **b**, Coloured boxes show the analogue events in 1960–2020 from Extended Data Tables 1 and 2 in terms of the 25%, 50% and 75% quantiles of the ten largest return periods in the flooded area^1^. Grey bars show the medians of the ten largest events at all locations of the analogue events; colours indicate the season of the floods.

To better understand the relevance of the 1342–1343 floods to present-day flood management, we placed them into the context of recent European floods. We chose analogue events in the last 60 years that were similar to the historical events in terms of affected area, runoff generation processes, seasonality and, where possible, flood magnitude (Fig. 5 and Extended Data Tables 1 and 2).

The Jacob and Bartholomew Floods show characteristics similar to those of some of the greatest twentieth century Vb-induced Central European floods (2005 and 1999, respectively), when much of the atmospheric moisture originated mainly from the Mediterranean^5^ (Supplementary Information section 4). The similar characteristics were occurrence in summer, extreme precipitation over the northern Alps indicative of Vb storm tracks and sunshine in Scandinavia indicative of atmospheric blocking. The Magdalena flood exhibited elements of a Vb pattern, but with greater structural complexity, and showed similarities to the events of 2013 and 2021 (Supplementary Information section 4). However, the spatial extent of the Magdalena flood was much larger than that of the recent floods. The comparison shows that the 1342–1343 double flood year was truly extraordinary, as if the extreme 1999, 2002, 2005, 2006 and the 2013 great European flood events and a dozen of other 10- to 100-year return period floods had all happened within less than 2 years. In 1342–1343, more European scale outstanding floods occurred than in the previous 60 years, and they were mostly larger (Fig. 5). These return periods, presented in Fig. 5, are expert-based, subjective estimates, derived from documentary, archaeological and sedimentary water-level evidence.

The 1342–1343 period was also rather unusual in its flood timing. Along the Main and the Middle Rhine, the Magdalena Flood occurred in summer while the largest twentieth to twenty-first century floods occurred in winter or spring^11,18^. In August 1342, a major flood occurred in northern Italy at the Reno, but essentially all floods in the twentieth century have been in late autumn or winter.

## Process causes: volcanoes and sea ice

The extraordinary magnitudes and frequency of the 1342–1343 floods are consistent with the low temperatures evidenced by tree-ring analysis^19,20^ and a historically negative correlation between temperature and floods^11^. Although no direct causal explanation is yet possible based on the available climate models and simulations, our systematic analysis of documentary-based and high-resolution paleoclimate, terrestrial and extra-terrestrial evidence suggests that Arctic sea ice reduction, volcanic eruptions and related internal climate variability together could be responsible for the exceptional flood sequence.

**Fig. 6:**
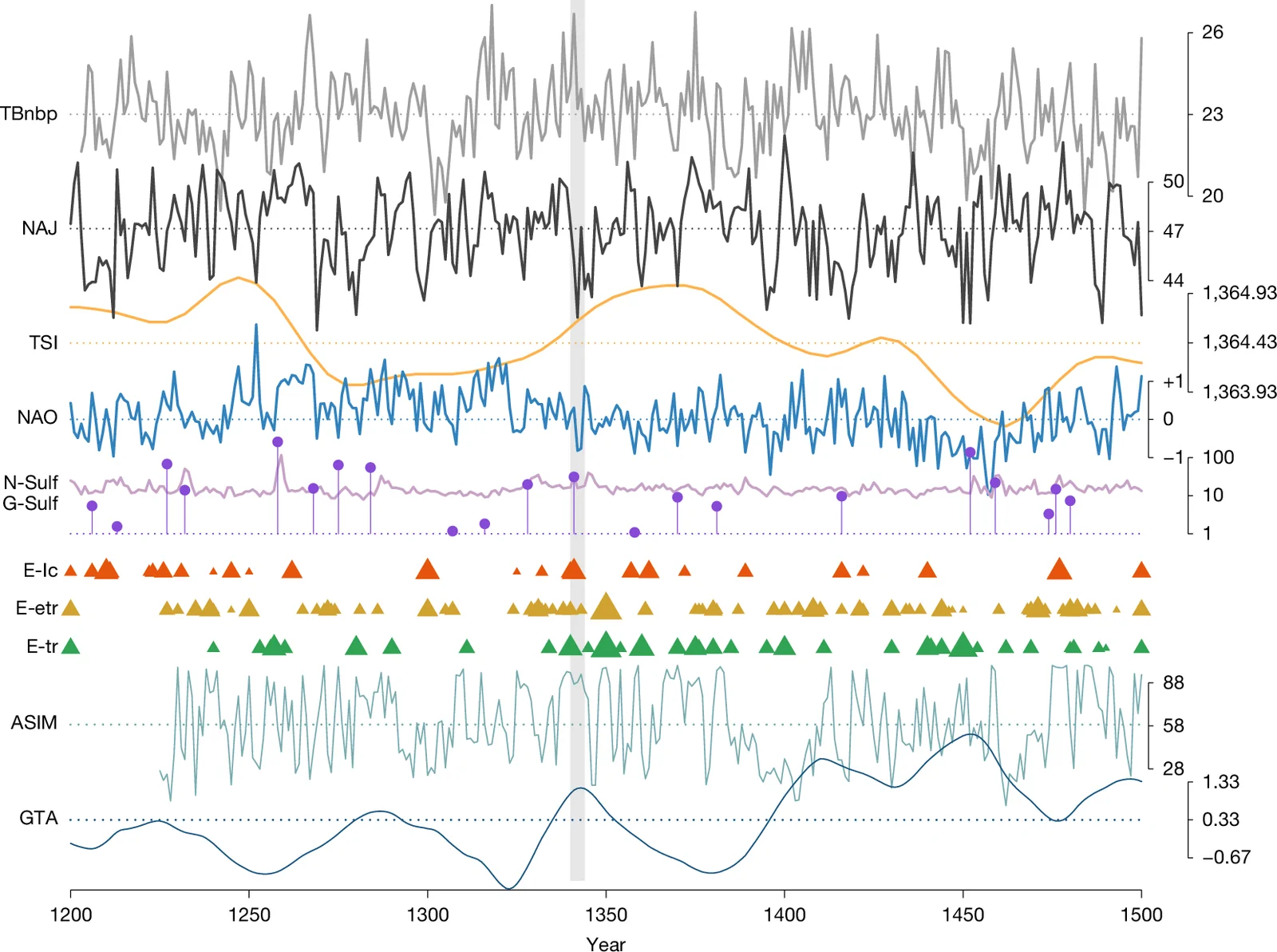
Possible extra- and intra-terrestrial causes of European extreme weather and floods. The graph shows possible causes of European extreme weather and floods. Coloured triangles, number of volcanic eruptions (size of triangles indicates the number of known eruptions per year from one to seven). ASIM, ratio of melted Arctic sea ice in summer (Franz Josef Land); E-Ic, Icelandic eruptions; E-etr, extratropical eruptions except Icelandic; E-tr, tropical eruptions; G-Sulf, global sulfate injection; GTA, Greenland annual temperature anomaly (normalized data; Supplementary Data 6); NAJ position, North Atlantic Jet Stream position; N-Sulf, Northern Hemisphere sulfate injection; TBnbp, tropical belt northern boundary position (indicator of the north boundary position of the Hadley cell); TSI, total solar irradiance. Grey bar, period 1340–1344. The unusual flood sequence can be explained by volcanic eruptions and Arctic sea ice decrease. For details on the variables, see Methods.

Based on the Smithsonian Institution Global Volcanism Programme (GVP)^21^, at least five tropical volcanoes (E-tr in Fig. 6)—including Pelée in the Caribbean for which the volcanic explosivity index (VEI) is 4—erupted around 1340, although their dating is uncertain. Ice-core analyses suggest a major tropical eruption between 1341 and 1345, reflected in global sulfate injection and Northern Hemisphere sulfate injection^22–24^, as shown in Fig. 6 (Supplementary Information section 5.1). Out of the eight extratropical eruptions in 1340–1341, according to the GVP database, an unusually high number of five occurred in Iceland (E-etr, E-Ic in Fig. 6). The Icelandic eruptions include the May–June 1341 explosive eruption of the Hekla (VEI = 3 (ref. ^25^) or 4 (ref. ^26^)), which induced mass livestock losses, land degradation, dearth and an important long-term migration in South-Iceland, supported by four reliable, local contemporary sources (for example. the Skálholt annals; Supplementary Information section 5.1). An extratropical eruption, probably the Hekla event, is also evidenced by atmospheric aerosol injections derived from ice-core deposition data^20^ (N-Sulf in Fig. 6).

Although modest in terms of global aerosol forcing^20^, recent observations and modelling studies indicate that, per unit sulfur injected into the stratosphere, extratropical eruptions of this size have a disproportionately large atmospheric impact^24,27^, and can generate a Northern Hemisphere climate response around 7.5 times stronger than tropical eruptions, potentially exceeding the atmospheric impact of the 1991 Pinatubo eruption^28^, especially if the eruption occurs in June^29^.

Observations and climate simulations suggest that aerosol-induced stratospheric warming, caused by volcanic eruptions, combined with reduced incoming solar radiation tends to lead to Northern Hemisphere near-surface air cooling^30,31^. This cooling has probably induced the southward shift of the Intertropical convergence zone and the northern boundary of the Hadley cell from 1341 to 1342 (the tropical belt northern boundary position moved from 27° to 23°; Fig. 6) and moved the North Atlantic Jet Stream to a very southern position of 42° by 1342^32,33^ (Fig. 6). Climate scenario simulations and reconstructions (Supplementary Information section 5.2; Methods) support a weakening effect of these processes on the polar vortex and polar jet, resulting in enhanced Rossby-wave meandering, a shift of the Arctic Oscillation and North Atlantic Oscillation (NAO) from positive to negative phases (NAO drops from +0.3 to −0.8 in Fig. 6), and a transition of El Niño–Southern Oscillation (ENSO) to a positive (El Niño) state in 1342^27,34,35^. Together, these circulation anomalies support an increased likelihood of high-intensity cyclones, prolonged rainfall and thus high-intensity flooding^27,36^ (Supplementary Information section 5.2).

Another plausible contributing factor to the unusual sequence of floods is multi-annual Arctic warming (GTA in Fig. 6) and the decline in Arctic sea ice (ASIM in Fig. 6) from the mid-1330s onward (Supplementary Data 5 and Supplementary Information section 5.2). Reduced sea ice lowers surface albedo and increases North Atlantic sea surface temperatures^37^. The warmer sea surface conditions in the early 1340s^38^, combined with lower near-surface temperatures related to volcanic activity, probably favoured more frequent and intense cyclones and increased mid-latitude European precipitation in the winters and summers of 1342–1343^37,39^. Arctic sea ice decline may also have contributed to the shift of the northern boundary of the Hadley cell between 1341 and 1342 (TBnbp in Fig. 6), and to a strong positive summer NAO^40,41^, in combination with global climate modes^39^ (Supplementary Information section 5.2; Methods). In addition, reduced sea ice probably amplified the eruption-induced shift of the Arctic Oscillation toward a negative phase and weakened the polar vortex, facilitating cold-air intrusions into Europe and enhancing rainfall in both winter and summer^40,41^ (Supplementary Information section 5.2). Together, volcanic forcing, Arctic sea ice decline and their interaction represent plausible contributors to the exceptional flood sequence, alongside internal climate variability.

## Why the other floods were forgotten

Although most of the literature^3,6,7,12^ acknowledged only a single large-scale event in the period 1342 to 1343, known as the Magdalena Flood, the new dataset provides evidence of numerous major floods. Previous focus on a single event is due partly to the low temporal resolution of soil erosion and sedimentary evidence (Supplementary Data 2b). It also reflects the fragmentary survival of medieval documentary sources and the uneven geographical distribution of archives, which tend to privilege politically and economically central regions where no major destruction of medieval documents occurred later^16,42^.

It is also surprising that, out of the 16 events described here, only the Magdalena Flood survived in public memory. This may be because it was the first time in the German areas when numerous flood marks and memorial plaques were mounted by local authorities and as a community effort and warning on the walls of iconic buildings^12^ whereas, for example, no flood marks were set for the Candlemas Flood in Prague^43^. The establishment of such material markers has been shown to play a crucial role in shaping long-term risk awareness and collective memory^7,12,43^. Moreover, narrative amplification in chronicles and other documents may have reinforced the prominence of a single catastrophic episode, whereas closely spaced subsequent floods were perceived as continuations rather than distinct disasters. Institutional responses often crystallize around emblematic events, further marginalizing others in historiography^12,44^.

## Global extremes and future perspectives

The unusual flood years of the early 1340s in Europe align with extreme hydro-climatological conditions in other continents, suggesting a possible link through teleconnections (Supplementary Information section 5). Multi-annual Barents Sea ice retreat was aligned with extreme summer precipitation in Eastern Mongolia and Northeast China during 1342–1344, as well as with severe flooding of the Yangtze River, resulting in widespread dyke breaches and major disruptions to the Grand Canal, China’s most important waterway^45,46^. On the other hand, precipitation on the Tibetan Plateau and streamflow of the Yellow River further in the south and of the Brahmaputra were very low^47,48^, because of Monsoon failure—a typical response after major extratropical volcanic eruptions and Arctic sea ice decrease^43^ (Supplementary Information section 5). Similarly, the 1341 to 1344 droughts in Yucatan, Guatemala^49^ and New Mexico have been linked with volcanic eruptions and Arctic sea ice decrease^50^. In contrast, the White River in Arkansas shifted from extreme flooding in 1341 to very low flows in 1342, reflecting pronounced hydroclimatic variability in the south-central United States^51^.

Current flood management decisions are based routinely on observations from recent decades and occasionally incorporate isolated historical floods. This study shows that sequences of very large floods, more extreme than those in recent decades, are possible and may occur again. Furthermore, climate projections suggest an increase of extreme extratropical cyclones in Europe^52^ and observations point to increases in high-intensity convective events^53^. Megafloods that may affect the same, extensive areas within a few months have important implications for flood risk management. This is because current response of societies to extraordinary floods is usually slow, and it may take decades for flood mitigation measures to be implemented for political, financial and administrative reasons^54^. Current flood management is thus not necessarily prepared for such sequences. The results of this study suggest strongly that these implementation processes should be accelerated, and proactive flood risk management strategies that explicitly account for unusual sequences of extreme flood events are urgently needed, for example through adaptive planning approaches that consider cumulative impacts, flexible response capacities and local flood-proofing measures.

## Methods

### Development of the historical flood database

Flood evidence in Europe over the period 1341–1343 was collected from narrative sources (chronicles, annals) and legal and economic administrative documentation (charters, accounts) as well as other documentary source types (thanksgiving inscriptions/memorials, scientific discussion, flood marks) (Supplementary Data 1–3). Although chronicles provided most of the information in most parts of Europe, in some areas, such as the Carpathian Basin, charters were more relevant^16^. Chronicles and annals often captured the greatest floods in a systematic way, but did not cover smaller floods or floods that occurred shortly after a great one. The other source types provided non-systematic evidence on individual flood events and were usually more precise in location.

We applied critical historical source evaluation, removing evidence deemed unreliable, and separating contemporary and non-contemporary sources: around 77% of the flood data entries come from contemporary sources, the rest from medieval sources based on earlier, contemporary source evidence. All floods are covered by contemporary sources, and other medieval sources were only used as complementary evidence. For over 70% of the floods, several types of independent contemporary source evidence was available. Giving priority to contemporary sources sets this study apart from previous research and enhances its reliability, as contemporary sources are first-hand information. This process resulted in 168 flood entries in the database on 60 European rivers, and four large lakes and wetlands.

In the period 1341–1343, several major floods occurred on the same river, including the Rhine, Tisza, Po, Elbe, Danube, Axios, Main, Inn, Drava and the Altmühl. For example, in the upper sections of the Tisza River, three major and two notable independent floods were recorded. Inspired by the term ‘Magdalena Flood’ used to describe the flood that peaked on the day of Saint Maria Magdalena, we propose similar names for three other major floods: the Candlemas, Jacob and Bartholomew Floods (Extended Data Tables 1 and 2).

### Magnitude class and return period estimation

We assess the magnitudes of each event in two ways. In a first step, we categorize each flood during 1341–1343 into one of three classes, consistent with the 500-year European database^11^ using the source texts, where class 1 represents notable floods, class 2 great floods and class 3 extraordinary floods. As more detailed information was available for the 1341–1343 floods than for the 500-year series, we also assign return periods in a second step. As expert-based, interpretative assessments, the return periods (Fig. 5) draw on a comprehensive collation of local and regional historical quantitative and qualitative water-level data, complemented by archaeological and sedimentary evidence. Even where quantitative indicators such as preserved flood marks are available, their interpretation necessarily reflects the substantial long-term modification of catchments and river systems. Accordingly, the estimates should be understood as contextually informed reconstructions that integrate several lines of evidence within an evolving environmental framework. Direct comparisons between medieval and modern floods are complicated by substantial anthropogenic changes in river morphology, floodplains and catchment hydrology since 1342. Accordingly, our return period estimates do not rely on direct comparisons of absolute flood levels or damage with modern gauged floods, but instead use a relative, rank-based approach that is standard practice in historical flood hydrology. We both identify most likely return periods and an uncertainty range. The method, detailed in Supplementary Information section 1, depends on the information available^55^:

1. Exact numerical information on the maximum water level or the maximum flood extent: these cases were compared with modern floods where the relationship between water level and return period are known, taking into consideration the possible uncertainties and changes that occurred in the landscape, hydromorphology, hydrology and river dynamics over time. Most of the maximum water-level estimations presented in Fig. 5 belong to this category. For example, based on flood marks and maximum water-level descriptions with exact localization, with additional information about changes in the environmental conditions such as the landscape, the town wall and the river morphology, the Magdalena Flood on the Main at Würzburg was estimated as a 1,000-year return period flood, and uncertainty ranges were identified^6^. Based on the report by local monk Johann von Winterthur^56^ that the Barfüsser Church was flooded up to the upper church (or podium), and on the account by Heinrich von Diessenhofen that, at roughly the same time, the town wall of Konstanz was overtopped only at the Dominican Friary, we estimated the return period of the Bartholomew flood in Lindau to be around 1,000 years^57–60^ (Supplementary Information section 1). This estimate accounts for lake sedimentation rates, differences in church floor elevations in Lindau and the known height of the medieval town wall in Konstanz Return period estimate with uncertainties for the same flood are also available for the Upper Rhine at Basel^55,60^ (Supplementary Information section 1). Contemporary chronicler Fritsche Closener^58^ wrote that the Bartholomew Flood was 2 feet higher than the Jacob Flood in Strasbourg, from which the differences in the return periods on the Rhine were estimated. Contemporary sources also report the height of water standing in churches (for example, in Vendôme and Luzern) and note that churches in Rouen and Luzern could be entered by large boat during the Candlemas and Jacob Floods, respectively. From these accounts water levels and return periods were inferred, considering elevation changes. In many cases hydro-morphological, and archaeological data were used to assist in the comparison between fourteenth century and modern conditions (Supplementary Information section 1).
2. Descriptive, quasi-numerical information related to the flood magnitude. In this case, the return period was inferred from the assessed frequency of how often a similar situation occurred over the centuries and how unusual it was in terms of infrastructure damage. For instance, during the Candlemas Flood in Prague^59^, where two-thirds of the famous Judith stone bridge were destroyed, we estimated a return period of at least 300 years. This estimate is based on the fact that the bridge was constructed in the twelfth century, taking previous floods into consideration. The Candlemas Flood was probably the most outstanding flood in several hundred years.
  The exceptionally severe damage to riverine infrastructure and town buildings, together with mass fatalities and the destruction of entire villages, supports this estimate (Franciscus Pragensis)^59^. Based on this and other information, such as a late medieval flood mark, and alluviation data from archaeological profiles, the return period of Candlemas in Prague was estimated as 500 years with a lower limit of the uncertainty range of 300 years. The upper limit of the uncertainty range was estimated as 1,000 years from the fact that the Roudnice bridge 50 km downstream of Prague was not damaged (Supplementary Information section 1). These estimates are similar to the Candlemas return periods for Vendôme estimated in step 1.
  Similarly, Heinrich von Diessenhofen^60^ reported that during the Jacob Flood all bridges along the Rhine between Schaffhausen and Strasbourg were completely destroyed and Johann von Winterthur^56^ noted that the vineyards (never placed in flood-prone areas) and entire districts of Lindau were destroyed, suggesting a return period of around or greater than 100 years (Supplementary Information section 1).
3. Information on the flood magnitude expressed by the choice of language and the broader environmental, legal or military implications: Here again, the return period was inferred from the assessed frequency of the event and, in some cases, from infrastructure damage, which provided indications of how far flood waters extended across the landscape and thus allowed estimation of water levels. The mid-March 1342 flood^61^ at the lower sections of the Upper Tisza obstructed the legal processes over debated land boundaries to the extent that survey participants could not even reach the region of the settlements in question, suggesting an event that does not happen more often than once or twice a century^16^. In the same area the medieval environment was described in this and other charters with exact dating, and detailed maps are also available in the eighteenth and nineteenth centuries, which make it possible to locate the flood and its approximate extension^16^.

The Strassburger Chronik of Fritsche Closener^58^ describes the Jacob Flood at the Rhine “Do man zalte 1343 jor, do wart (der) Rin also groß und ging also sere us, daz nieman do zemol lebete der üt gedohte oder ie hette gehoret sagen, daz er so groß würde.” (The flood level and extent was beyond imagination of anybody living or any reports), from which we inferred a minimum 100-year return period (three generations), based on an interpretation of communicative memory^62^. More broadly, the term ‘diluvium’ (deluge) appears only rarely in medieval Latin texts and is generally understood to denote events with a return period of several hundred years, and therefore related to cultural memory^62^. It is used for all four major floods, for example, by Heinrich von Taube^63^ for the Magdalena and Jacob floods in Eichstätt, and by Franciscus Pragensis for the Candlemas Flood in Prague^59^.

Further details are available in Supplementary Information section 1, where case studies of return period estimations are presented on the examples of the Candlemas, Magdalena, Jacob and Bartholomew Floods, and are based on refs. ^64–157^.

### Development of historical weather database

We have developed a European weather database for the 1341–1343 period (Supplementary Data 2a). It contains 213 entries, of which 98% are from contemporary sources and 2% stem from the late fourteenth century and fifteenth century (Extended Data Table 2). The sources include weather reports, providing daily, monthly and seasonal resolution, from chronicles, legal administrative sources (charters, official letters), manorial accounts and, occasionally, contemporary scientific discussions. To understand the continental-scale weather, we also use part of the contemporary weather diary of William Merle^158^, which contains daily entries with monthly and seasonal summaries for Central and Southeast England during 1341–1343. About 20% of the weather data come from the same sources as the flood reports and identify explicitly the cause of flooding. They are listed in Supplementary Data 1a (column U). The remaining 80% of the weather data were reported in sources different from those of the floods, and provide corroborative information. They are shown in Fig. 2c,d and given in Supplementary Data 2a. We identify weather events including heavy precipitation, low temperature, high temperature, wind and hail/storms, and classify them into three classes, similar to the floods. We also identify the presence of atmospheric blocking, when prolonged clear skies, sunshine and extended periods of dry (and in summer hot) weather were reported. Two events were classified as Vb events^159^ (Extended Data Table 2 and Supplementary Information section 1), which are associated with cyclones originating near the western Mediterranean or Gulf of Genoa and moving northeastward across northern Italy toward central/eastern Europe, turning counterclockwise. The classification was based on several characteristics known to be associated with Vb events^5^, sometimes reported by eye witnesses, such as heavy rainfall in the Northern Alps, flooding in the Upper Danube basin and of Lake Constance, and a preceding shift to strong southwesterly winds in southern England (Supplementary Data 6a). Tree-ring based cloud cover^160^, sea surface^38^ and air temperature^19^ reconstructions provide indirect information on atmospheric pressure, suggesting stationary summer high pressure systems over the North Sea and the Atlantic coastline of Northern Europe and thus blocking in 1342 and 1343.

The temporal coherence and wide spatial extent of the reports can be used to infer large-scale hydroclimatic conditions even where local weather data are missing. For example, although direct weather information is lacking for western France during the Candlemas Flood (event 2), contemporary sources (column AB in Supplementary Data 1) provide precise timing, duration and the causes of flooding on the Loir, Eure and Seine, which match closely flood reports from Germany, Czechia and Central Europe as far east as the Western Carpathians (rows 9–16 in Supplementary Data 1a), and are consistent with daily weather observations from southern England (Supplementary Data 2a).

### Development of the historical impact database

A concise historical flood and extreme weather impact database was developed comprising 363 recorded impacts at more than 100 locations, 98% of which come from contemporary sources. Direct, immediate impacts include damage to infrastructure, agriculture, livestock and human life, as well as direct and immediate societal reactions. In 99% of cases, the contemporary sources attribute these impacts explicitly to flooding or extreme weather. In the remaining few cases, impacts are not attributed explicitly but are inferred from clear contextual indicators such as grain prices and salt production problems. These direct, immediate impacts are listed in Supplementary Data 3, with links to Extended Data Table 4, where impacts were classified into 28 categories and aggregated into six main impact groups. Original source texts on flood damages, and information on examples of associated prevention measures are given in Supplementary Information section 2, and are based on refs. ^161–169^.

Indirect, longer-term flood and extreme weather impacts, food shortage, grain prices, dearth, famines and associated socio-economic responses are discussed and interpreted in a broader, multi-annual European context in Supplementary Information section 3, and are based on refs. ^170–207^.

### Comparison of the number of great floods per year with past 700 years

**Extended Data Fig. 2:**
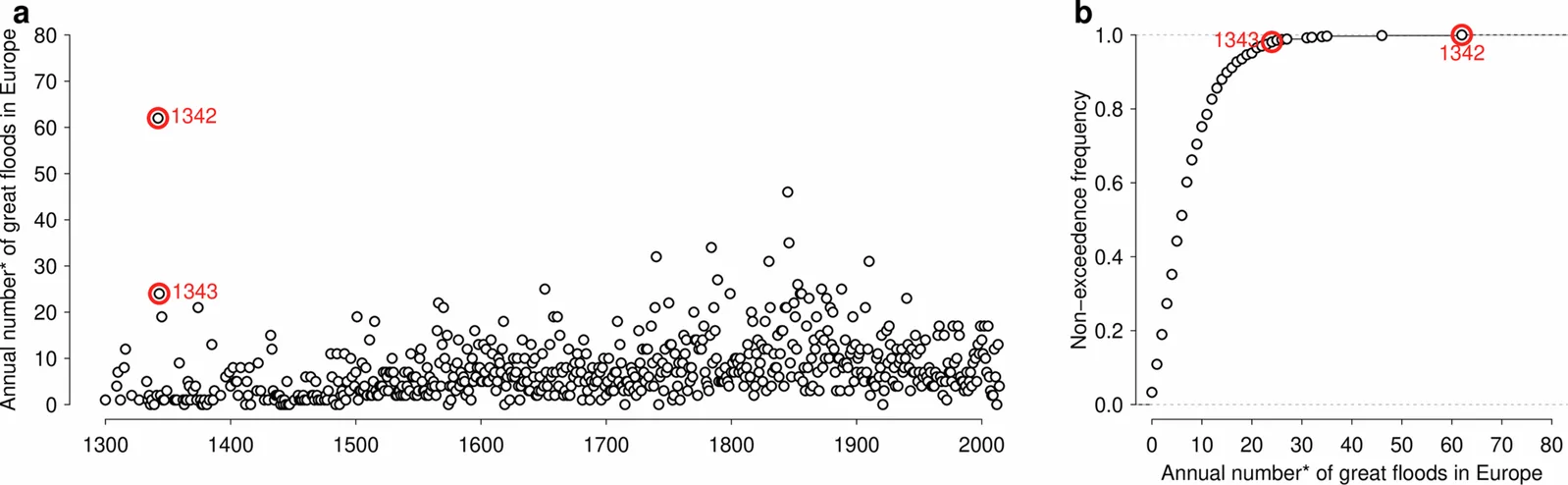
Annual frequency of great and extraordinary floods in Europe in the last 700 years. Original data without bias correction (*). Source of data: Supplementary Table 4.

For placing the 1342–1343 period into a longer historical context, we extended the 103 documentary-based European flood series from 1500 to 2010^11^ to the beginning of the fourteenth century (Fig. 4, Extended Data Fig. 2 and Supplementary Data 4), utilizing the published collection of great and outstanding medieval floods in Europe with an emphasis on Central Europe^16^. This database was further enhanced with the data based on a systematic check of the medieval historical data (using critical source analysis) available in the grand European collection of weather and weather-related hydrological phenomena^208^. We included in our database only the (major) flood events of more important flood years that could be traced and critically evaluated in the original historical source context. We also added 59 new flood series to increase the overlap with the rivers affected by the 1342–1343 floods, using principal data collections^209,210^ and local sources^9,12,16,211^, applying critical source evaluation and giving priority to contemporary source evidence. The new database used here thus contains 161 flood series from 1300 to 2010. Because of the enormous quantity of databases and original sources that had to be checked through and critically evaluated, regarding the additional flood data (added to the 500-year database) we focused on major flood years and the floods marked as great or outstanding (index 2 and 3) in magnitude. We applied the same bias correction to the new database as that applied to the original 1500 to 2010 database^11^, which corrects for likely missed small or no-events in years without records.

### Identification of modern analogue events

To gain a better understanding of the potential characteristics of the historical events, we identified modern analogues to the 1342–1343 flood events, similar to the facial reconstruction sometimes done in palaeontology (Extended Data Tables 1 and 2 and Supplementary Information section 4). We selected the modern analogues based on the following similarity criteria: timing and seasonality, duration, causes and weather background, magnitude, location and minimum spatial extent. We scrutinized scientific publications, hydrological reports and relevant newspaper and online news entries related to damaging floods and their weather conditions in 1960–2020, and also use the European flood database of annual maximum discharge^1^. Given that much of the historical information is qualitative, the similarity criteria were applied manually by qualitative judgement. The comparison resulted in between three and five analogue events, ranked by their similarity, for each of the 16 historical events (Supplementary Information section 4). The top ranked events are presented in Extended Data Tables 1 and 2 and are shown in Fig. 5b. The modern analogues fill in the gaps where no historical information is available in 1342–1343, but they result in potential, reconstructed spatial distributions and durations (Supplementary Data 5).

The final reconstructed flood patterns are shown as ellipsoids in Fig. 1 and represent a conceptual depiction of the likely spatio-temporal extent of flooding, based on historical flood and meteorological evidence, complemented by modern analogues where the historical record is incomplete (Extended Data Fig. 1). The use of ellipsoids is a deliberate choice to balance overfitting and underfitting of the available historical data.

Each ellipsoid is based on the number, timing and spatial coherence of reported flood events (Supplementary Information section 4 and Extended Data Fig. 1). When only two or three contemporaneous floods were documented on different European rivers, we applied a multi-step validation procedure. We first consulted the pan-European flood database^1^ to identify modern analogue events in which the same rivers flooded simultaneously or near-simultaneously, thereby assessing whether sparse historical reports could represent a larger-scale hydroclimatic event rather than isolated local extremes. Second, for each candidate analogue, we compared documentary weather information from before and during the 1342–1343 floods (Supplementary Data 2) with modern meteorological data from analogous flood events at both continental and regional scales. At the continental scale, we assessed reconstructed and observed European synoptic circulation patterns to evaluate whether the historical floods were associated with coherent large-scale atmospheric configurations (for example, persistent low-pressure systems or zonal flow anomalies). At the regional scale, we examined weather conditions within the affected river catchments, including precipitation persistence, temperature anomalies and antecedent soil-moisture conditions where available. These comparisons drew on pan-European reanalysis products, national daily and monthly weather records, and publicly accessible archives such as NOAA and Climate Explorer. A full description of the comparison between historical flood, weather and the modern analogues, and the coverage of the flood is given in Supplementary Data 5 and Supplementary Information section 4, and are based on refs. ^212–333^.

The agreement between the spatial structure of modern analogue floods, associated atmospheric circulation patterns, and fourteenth century documentary evidence informed the size, orientation and placement of each ellipsoid, ensuring that the reconstructed flood patterns reflect both the historical reports and their physical plausibility within a continental-scale hydroclimatic framework.

### Attribution of the 1341–1343 floods

To shed light on the physical reasons for the unusual 1342–1343 flooding (Fig. 6), data were obtained from the following sources (Supplementary Data 5). The tropical belt northern boundary position—an indicator of the north boundary position of the Hadley cell—is based on spring-precipitation reconstruction from tree-ring evidence of five mid-latitude regions^334^. The North Atlantic Jet Stream position is derived from an ensemble of water–isotope based climate model simulations, and the mean annual water isotope (*δ*^18^O) and annually accumulated snowfall records detected in Greenland ice cores^32^. The total solar irradiance is based on paleoclimate (temperature, coral *δ*^18^O) proxies and model simulation^335^.

The annual index values of the NAO in the last millennium, applied in this study, were reconstructed based on 48 proxy series derived from ice cores, lake sediments, speleothems or tree rings^336^. Global and Northern Hemisphere sulfate injection series were initiated from the sulfate deposition content in Arctic, Greenland and Antarctic ice cores^337^. Arctic annual (normalized) temperature data in Greenland and summer sea ice melting data in Franz Josef Land in the Barents Sea, were derived from ^18^O ratio in ice cores^338,339^. Possible causative atmospheric links and process mechanisms from experimental case studies and climate simulations are discussed in Supplementary Data and Supplementary Information section 5.

Data on the number of volcanic eruptions are derived from the Smithsonian Institution GVP database^21^, and were divided into Icelandic eruptions (E-Ic), northern hemispheric extratropical eruptions excluding Icelandic (E-etr) and tropical eruptions (E-tr). The VEI, representing the magnitude and intensity of eruptions, is based on the Smithsonian Institution GVP database^21^ including its references that use a range of information such as lake sediments, ice cores and documentary evidence (Supplementary Data 6). This database includes small and large eruptions, whereas key publications and related databases^20,337,340^ discuss only the largest eruptions. The atmospheric impact of eruptions through volcanic radiative forcing and sulfate injection has been evidenced in the literature^20,22–24,27–31^.

Historical archives clearly document eruptions of Hekla and probably also other Icelandic volcanoes, such as the Eyjafjallajökull, around 1341 (Supplementary Information section 5). However, it remains difficult to assess whether these eruptions injected material into the stratosphere or whether aerosols were mostly tropospheric with limited impacts on climate. The 2010 Eyjafjallajökull eruption provides a useful modern analogue. One way to further test the proposed hypothesis would be to investigate sulfur isotope (Δ^33^S) measurements in ice-core records for the 1341 event, to discriminate between stratospheric and tropospheric sulfate deposition^28,341^.

Although the climate response and the floods of winter 1341–1342 show strong similarities to those of winter 1783–1784 following the Laki eruption^342^, an important difference is the decrease in Arctic sea ice in the late 1330s to early 1340s, which was absent in the early 1780s and which may also have contributed, among other factors, to the considerably wetter-than-usual summers of 1342 and 1343.

To further strengthen the interpretation, it may be worthwhile to conduct specific climate simulations using current aerosol optical depth reconstructions for the 1341 event^340,343,344^ to assess the likelihood of enhanced precipitation or flood-favourable circulation patterns. In addition, it would be useful to examine existing paleo-reanalyses, such as the Global 2000-Year Paleo Hydrodynamics Data Assimilation product^343^, which provide estimates of large-scale modes of variability.

Causative data, climate comparisons and global climate indices in 1341–1343 are available in Supplementary Information section 5, and are based on refs. ^345–395^.

### Estimation of Arctic Oscillation and NAO

NAO reconstructions^336,396–398^ sometimes show significant discrepancies, leading to a somewhat conflicting view of annual and winter NAO. Furthermore, there are no summer NAO reconstructions that extend back to the 1340s^399^. Thus, we analysed the documentary and natural proxy data of the seasonal and annual weather in 1342 and 1343 (Supplementary Data 2a,b), using the NAO–weather relationship presented by the United Kingdom Met Office^400^, to identify the seasonal signs of the NAO. The severe cold outbreaks in mid-latitudes and the unusually high temperatures observed in some high-latitude areas^401–403^ suggest prevailing negative winter Arctic Oscillation and NAO values in 1342 and 1343, which is consistent with a previous annual reconstruction^336^. Comparisons with contemporary data and modern simulations indicate that summer patterns probably exhibited prevailing positive NAO mode during both years because of the anomalously wet and cool conditions in the Mediterranean and the prevailing dry and warm patterns in the North Atlantic (Supplementary Data 2a,b).

For linking European weather in 1341–1343 to global climate processes, we also examined reconstructions of other Northern, Southern Hemispheric and global climate mode indices. For example, the combination of negative Arctic Oscillation and NAO, presence of the East Atlantic pattern and positive ENSO along with a positive Indian Ocean Dipole could be important contributing factors to the unusually severe and wet winter in 1342 (Supplementary Information section 5.2).

## Online content

Any methods, additional references, Nature Portfolio reporting summaries, source data, extended data, supplementary information, acknowledgements, peer review information; details of author contributions and competing interests; and statements of data and code availability are available at 10.1038/s41586-026-10888-8.

## Supplementary information

Supplementary Information This Supplementary Information file contains the following sections: (1) Examples for return period estimations. (2) Infrastructural changes and flood prevention measures after the 1342–1343 European floods. (3) High prices in Europe, food shortage, dearth and famine in 1342–1344. (4) Event descriptions and modern parallels. (5) Volcanic eruptions and Arctic sea ice melting: potential atmospheric and weather-related signatures, and global climate indices.

Supplementary Data 1 Flood data and source information and uncertainty evaluation.

Supplementary Data 2 Weather data and source information and uncertainty evaluation.

Supplementary Data 3 Impact data and source information.

Supplementary Data 4 700-year European flood index data.

Supplementary Data 5 Data series of possible extra- and intra-terrestrial causes of European extreme weather and floods.

Supplementary Data 6 Locations and explanations of the reconstructed 16 flood events.

Supplementary Video 1 Conceptual reconstruction of the 1342–1343 floods in Europe.

## Figures and Tables

**Extended Data Table 1:**
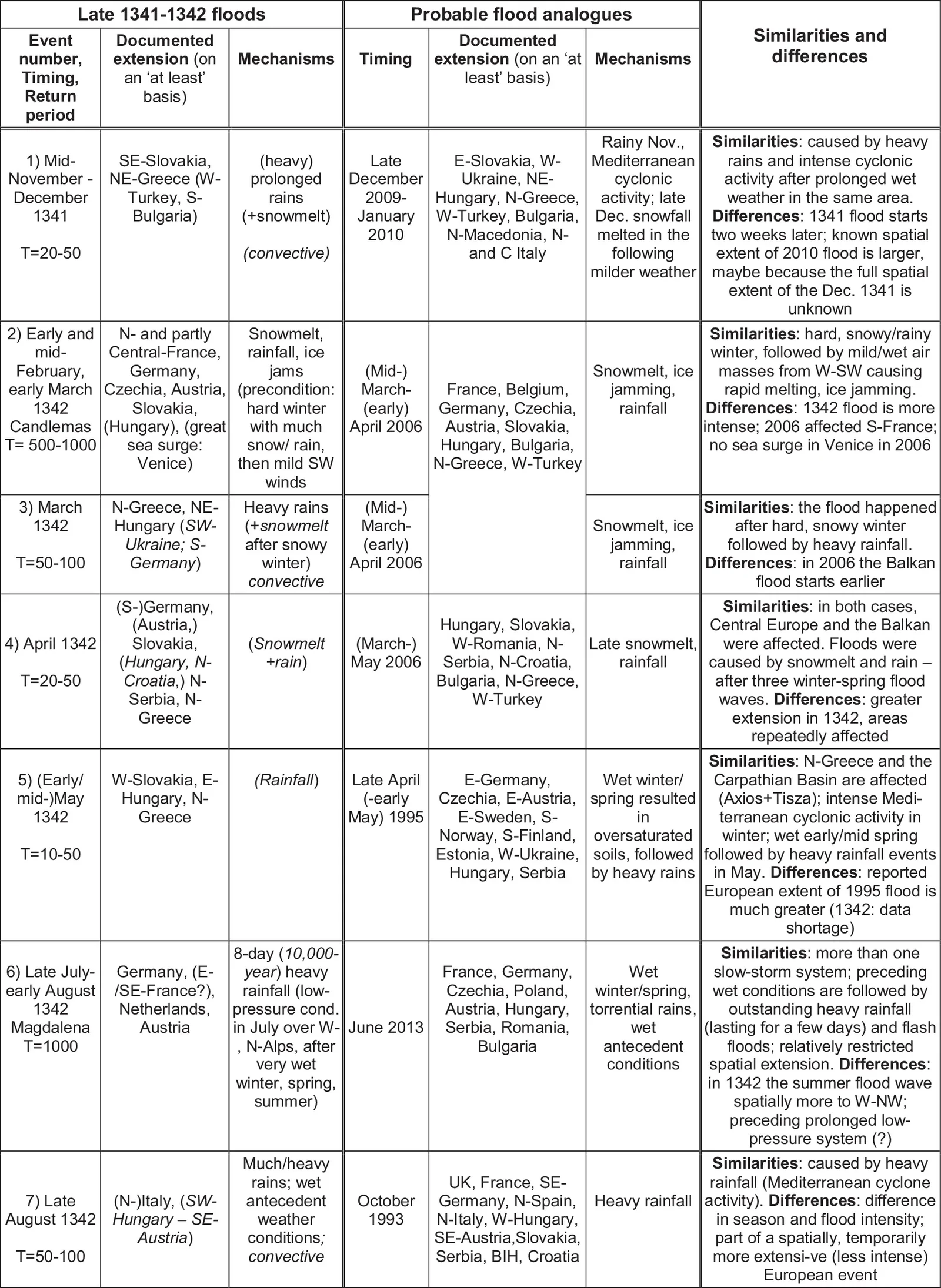
Overview of major floods and main causes between late 1341 and late 1342 compared to similar modern flood events in the period 1960–2020 (events 1–7)

Brackets indicate presumed (but not directly mentioned) information based on the sources. T is the estimated return period (yrs). Italics indicates higher uncertainty.

**Extended Data Table 2:**
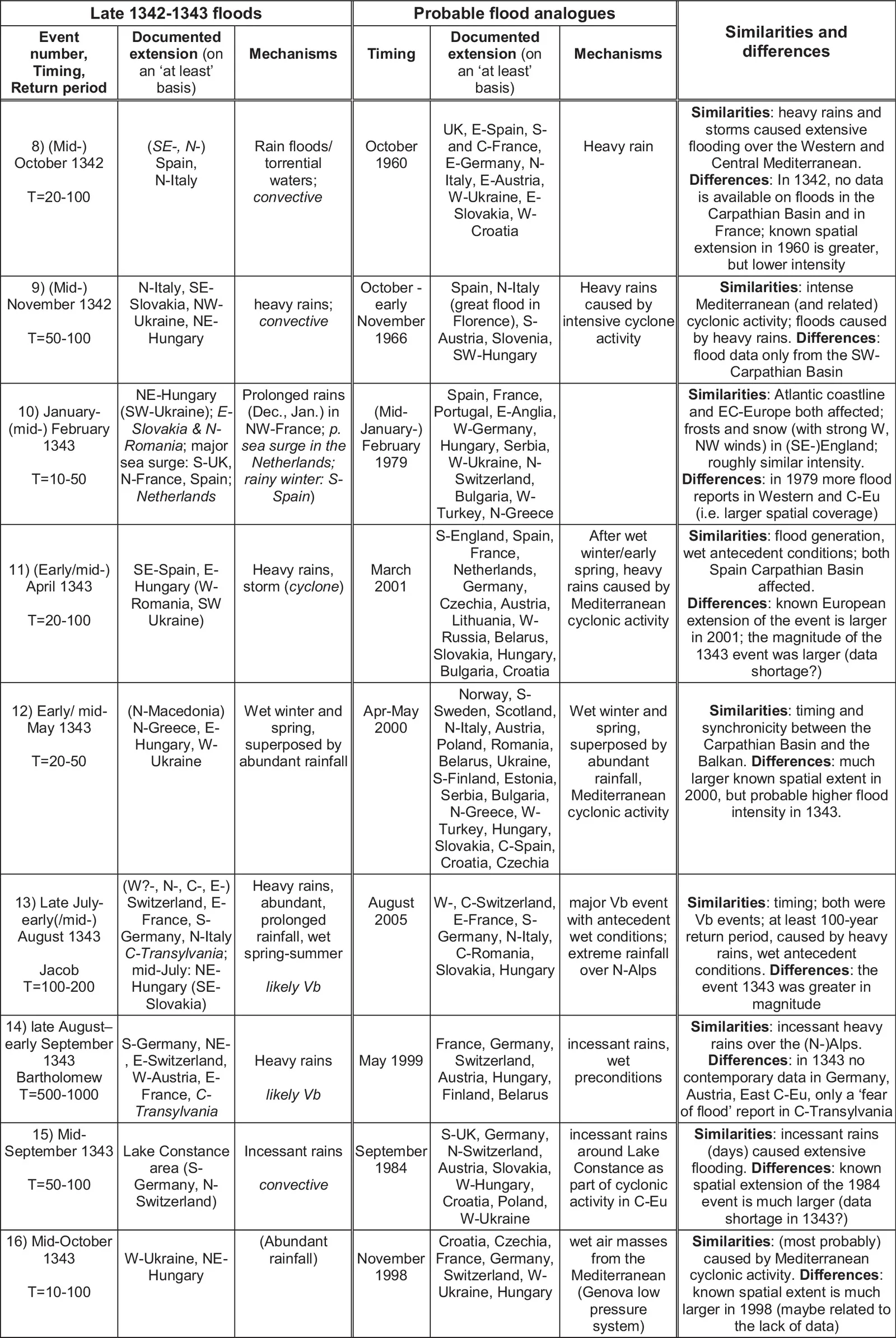
Overview of major floods and main causes between late 1342 and late 1343 compared to similar modern flood events in the period 1960–2020 (events 8–16)

Brackets indicate presumed (but not directly mentioned) information based on the sources. T is the estimated return period (yrs). Italics indicates higher uncertainty. Vb indicates cyclones originating near the western Mediterranean or Gulf of Genoa and moving northeastward across northern Italy toward central Europe, turning counterclockwise^159^.

**Extended Data Table 3:**
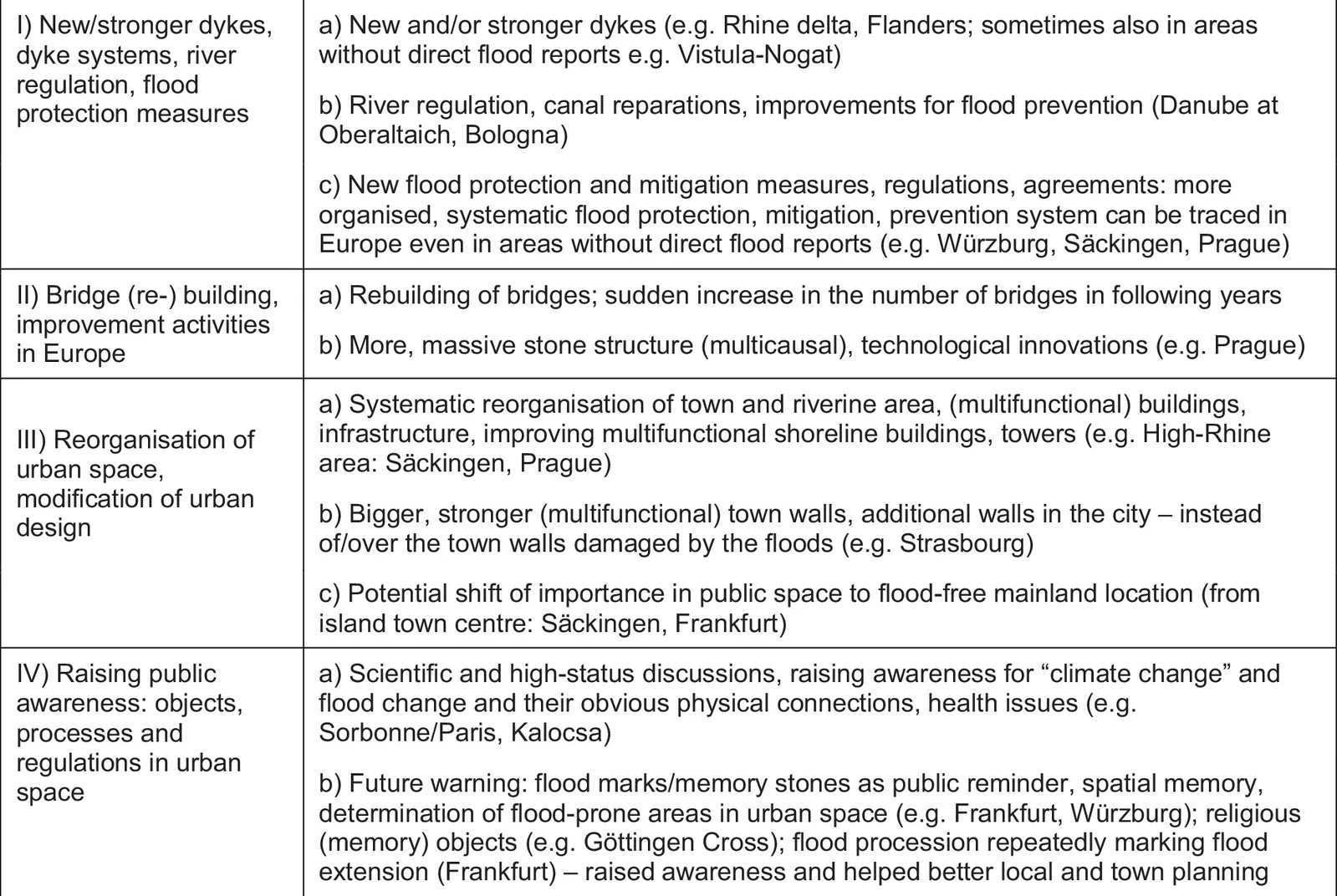
Long-term changes caused by the 1342–2343 floods (infrastructural innovations, strategies and new flood protection and mitigation design after the 1342–1343 events)

References; see Supplementary Data 3; an extended version of the table with original source text quotations on damages is available in Supplementary Information 2.

**Extended Data Table 4:**
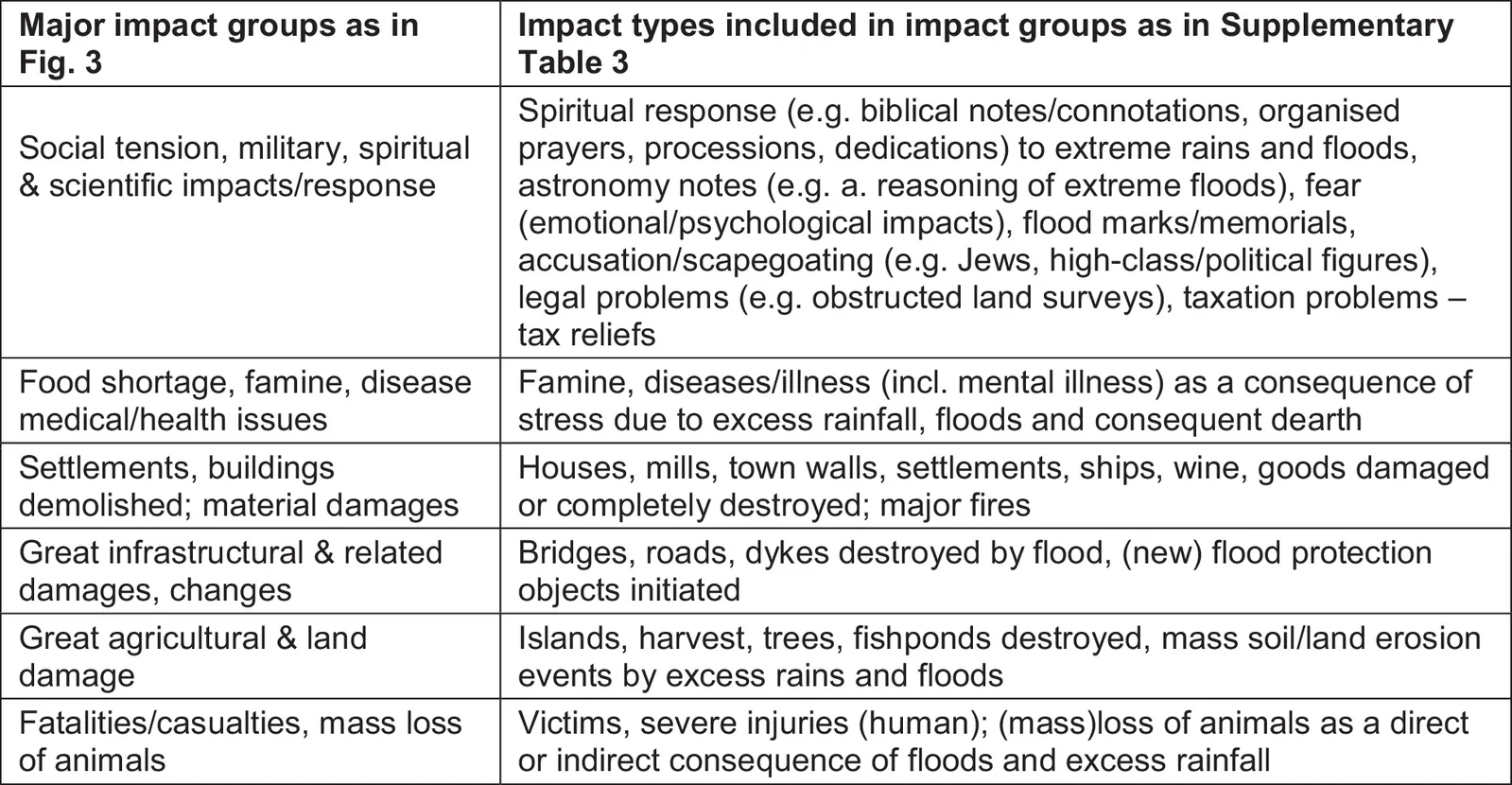
Socio-economic impact groups of the great weather and flood anomalies of 1342–1343

References see Supplementary Data 3.

## Data Availability

All flood- and weather-related documentary source data are available as Supplementary data.

## Code availability

No code was used in the data analysis.
