# Supplementary material for: Cascading continental-scale floods across Europe in 1342–1343
Source: Nature. 2026 Aug 12;656(8128):638–45. doi: 10.1038/s41586-026-10888-8 (PMC13489955; doi:10.1038/s41586-026-10888-8)
Supplement: Supplementary file 1 [file 41586_2026_10888_MOESM1_ESM.pdf]

nature portfolio

https://doi.org/10.1038/s41586-026-10888-8

Supplementary information

Cascading continental-scale floods across Europe in 1342–1343

In the format provided by the authors and unedited

Supplementary Information

to the paper “Kiss et al. Cascading continental-scale floods across Europe in 1342 – 1343” in *Nature* (2026)

Table of Contents

1 Examples for return period estimations 3

1.1 Event 2) February(-March) 1342: Candlemas Flood 3

1.1.1 The Vltava Flood in Prague (and other settlements) 3

1.1.2 Elbe flood at Dresden (and other places) 7

1.1.3 (Upper-)Danube flood 7

1.1.4 Seine flood at Rouen 8

1.1.5 Eure (Seine tributary) flood at Chartres 11

1.1.6 Loir flood in Vendôme 13

1.2 Event 6) Magdalena Flood: summer/July 1342 16

1.2.1 Main flood (ca. 1000y return period) 16

1.2.2 Rhine flood (ca. 100y return period) 19

1.2.3 Weser (Werra, Fulda) flood in Hannoversch Münden 20

1.3 Event 13) late July–early August 1343 floods 21

1.3.1 Flood of the Upper Rhine at Strasbourg 21

1.3.2 Flood of the High Rhine between Basel and Lake Constance 22

1.3.3 Flood of Lake Constance 24

1.3.4 The flood of the Limmat at Zürich – ca. 200-500 years 26

1.3.5 Flood of the Reuss and the Vierwaldstättersee az Luzern 28

1.3.6 Flood of the Töss at Winterthur 29

1.4 Event 14) Lake Constance, High- and Upper-Rhine 30

1.4.1 High-/Upper-Rhine flood 30

1.4.2 Flood of Lake Constance 30

2 Infrastructural changes and flood prevention measures after the 1342-1343 floods 35

2.1 Overview of flood damages and prevention measures 35

2.2 Flood damages in built-up area and riverine infrastructure: Source texts 36

3 High prices in Europe, food shortage, dearth and famine in 1342-1344 46

3.1 Subsistence crisis intensity & grain (beverages) prices in Europe, data sources and key price-related information in 1342-1343 46

3.2 Example for indirect economic consequences: Salt production problems and their relation to weather and floods in Italy in 1342-1343 ... 49
4 Event descriptions and modern parallels ... 52
4.1 Event 1) Nov-Dec. 1341 – closest parallel: December 2009–January 2010 ... 52
4.2 Event 2) Candlemas Flood: February 1342 ... 54
4.3 Event 3) March 1342 ... 57
4.4 Event 4) April 1342 ... 59
4.5 Event 5) early/mid-May 1342 ... 60
4.6 Event 6) Magdalena Flood: Late July-early August 1342: ... 63
4.6.1 Preconditions ... 63
4.6.2 General summer preconditions and the early summer rainy period ... 63
4.6.3 Weather of July 1342 and the (Maria) Magdalena Flood ... 64
4.6.4 Potential modern parallel(s) ... 66
4.6.5 Flood generation of the July 1342 (Maria) Magdalena Flood ... 67
4.7 Event 7) Late August (–early September) 1342 ... 72
4.8 Event 8) (Mid-)October 1342 ... 74
4.9 Event 9) Mid-November 1342 ... 76
4.10 Case 10) February 1343 ... 78
4.11 Case 11) April 1343 ... 80
4.12 Case 12) May 1343 ... 83
4.13 Case 13) Jacob Flood: July–early August 1343 ... 85
4.14 Event 14) Bartholomew Flood: Late August–early September 1343 ... 88
4.15 Event 15) Mid-September 1343 ... 91
4.16 Event 16) October 1343 ... 93
5 Volcanic eruptions and Arctic Sea Ice melting. Potential atmospheric and weather-related signatures, and global climate indices ... 96
5.1 Documentary sources and databases of volcanic eruptions (1340-1343) ... 96
5.2 Global climate and its response to volcanic eruptions and Arctic sea ice decrease ... 98

1 Examples for return period estimations

1.1 Event 2) February(-March) 1342: Candlemas Flood

1.1.1 The Vltava Flood in Prague (and other settlements)

Regarding the extreme flood of February 1342 on the Vltava River – caused by rapid snowmelt (of large amount of snow), ice break-up and blocking as well as rain – contemporary evidence suggests extreme destruction. In Prague, the legendary Judith Bridge, a stone bridge built in the mid-12th century, was mostly destroyed and swept away, and only ca. one-third or one-fourth remained. Transportation along and across the Vltava/Moldava was blocked for long also after the flood, Prague riverine districts, buildings, woods/trees, mills, entire villages along the Vltava were destroyed, with an enormous loss of people and animals (e.g., Franciscus Pragensis:[58] see Supplementary Data 1, 3; Supplementary Information 3). The described level of destruction and shock is only comparable to the greatest and most destructive floods in Prague and its surroundings (e.g., 1501, 1784, 2002). Thus, based merely on the magnitude and spatial extent of destruction and the exceptionally great attention and unusually detailed descriptions from contemporary medieval Czech authors, the flood had to be particularly outstanding in magnitude and in its consequences. As mentioned above, apart from snowmelt and rainfall, the Vltava flood was strongly influenced by ice jamming, and the large quantity of (wooden and other) debris (also mentioned by contemporary source) could also cause an even greater destruction (as also noted by the contemporary description).

Although no contemporary flood mark or maximum water level signs (i.e., quantitative information) remained that would directly help the quantification of the magnitude of this extraordinary event, a medieval reference, dated to 1481, suggests that the flood of February 1342 reached the "nose of the Bradáč (head carved into the stone wall at the Prague Old Town side of the Vltava River embankment near the Charles Bridge),[64] serving for centuries as a "natural" flood gauge.[65] In modern conditions, the "nose of Bradáč" would mean, a moderate, 3.4 m flood level, while more recent great devastating floods reach much higher levels, such as the famously destructive August 2002 flood with its 6.4 m or the February 1784 flood (that shows strong similarities in flood generation to the February 1342 event) with its ca. 5.8 m maximum height, and even the great August 1501 flood reached over 5 m flood level in modern conditions.[65] This great difference (especially when considering the huge devastation contemporary sources in 1342 describe) raises the question that this water level cannot be understood in modern environment, and we need to place it into a long-term environmental prospect. While trying to understand the potential difference between a 14th and the much later (e.g., 1501, 1784 or 2002) extreme floods, apart from other conditions, we have to take into consideration the information available regarding historical alluviation processes.

The city of Prague,[64] in the valley of the Vltava River built on fluvial terraces developed in the Pliocene and after; the stone structures in the old town area (incl. fortification) near the bridge were mainly built in the 13th century.[66,67] Sedimentary evidence regarding the alluviation of abandoned Vltava branches, covered by water only during floods, revealed that close to the banks of the old town the rise of the floodplain was around 4-6 m between the 11th and the 20th century.[68] There is a roughly 2.5 m difference between the ground level area of building complexes and garden initiated in the mid-17th-century.[69] While the earlier general, low-

floodplain alluviation related information suggests a roughly 0.4-0.6 m average ground/channel bottom area rise per century, the latter, high-floodplain or flood-free area related evidence suggests an average ground-level difference (compared to present-day terrain) of 0.7 m per century.

Other archaeobotanical investigation in a 9th-century riverbed, silted up and covered by cultural layers in the 10th century, was carried out in the area of the Lesser Town, revealing a 371 cm core with at least two significant flood layers in the 14th century, dated approximately from the second half of the 13th to 15th century. Although in this case the analysis is concentrated only on the late medieval period, the thickness of the cultural and alluviation layers – deposited within a time span less than 300 years (i.e., well over 1 m per century) – is rather significant;[70] this high sedimentation intensity, at least partly, is related to the construction of the nearby weir in the 13th century.

Further data come from the (geo)archaeological excavations carried out in the town and the floodplain area, where archaeological profiles, especially the ones consisting sedimentary layers, originated from historical Vltava floods, provide evidence on ground level evolution.[71] Four more case studies can be related to our case: one at Prague Klementinum in the Old Town (lowest Old Town terrace), the second, third and fourth at Klárov, Hellicova and Mišeňska Street in the Lesser Town:[70-73]

- In the first case east of the Charles Bridge over the high medieval settlement a 14th-century fluvial (flood-related) sedimentary layer was found in a depth of 3.5 m covered by stone waste – this suggests a roughly 0.5 m/century increase of ground/surface level in the area between the major 14th-century flood layer (possibly related to the greatest flood of the century, the February 1342 flood).
- In the second case (Kánov, near Mánes Bridge), the late medieval settlement layer on a former island of the Vltava, covered by thick early modern flood layer, was located around 2.5-3 m under the present surface, similarly suggesting a roughly 0.5 m/century surface increase.
- In the third case, at Hellichova, at the (upper) edge of floodplain under the post-medieval deposits 14th and then 15th-16th century alluvial (flood) layers were found. Here the 14th-century layer was around 2.5-3 m under the 19th-20th century ground levels, which again suggests an around 0.5 m ground level increase on average.
- Finally, at the Mišeňska Street excavations (close to the Malá Strana at the Charles Bridge), in a low-lying uninhabited medieval floodplain area the alluvial flood layers were dated with ceramics to the 14th-15th century. Here, the 14th-century layers are located roughly 3-3,5 m under the present surface, again giving a roughly 0.5 m/century average ground-level increase.

Thus, similar to the previous general conclusion, case studies support an around 0.5 m surface elevation increase per century in the flood affected areas and the narrow floodplain/riverbed as well. Although it is a very rough estimate, it provides some general (heavily averaged), but roughly coinciding information about the sedimentation rates and the dimension of changes in historical times. These data also clearly suggest that the maximum water-level values of extreme historical floods gained by using such flood marks as the Bradáč in Prague may need further evaluation, and it is necessary to incorporate the changing environmental conditions (esp. riverbed alluviation) during the centuries into statistical estimations (i.e., maximum water level, discharge).

Even if we count with a somewhat increased sedimentation and (urban and other) deposits in the later period due to population growth, the intensification of land use and the potentially higher flood activities broadly experienced in Central Europe partly already from the 14th but even more intensively from the 16th century, the archaeological evidence both in

the former riverbed (i.e. island), floodplain area and at the edge of the floodplain, the surface level difference between the (mid-)14th and the 20th–early 21st century suggested roughly 3-3,5 m (ca. 0.5 m/century) on average. In comparison, along the Middle-Danube (with extensive floodplain areas at least on one side of the river), the 0.5 m surface elevation increase happened during a much longer period (during ca. 200 years: between ca. the mid-13th and the mid-/late 15th century).[74,75]

Adding around 3.2 m (i.e., 3-3.5 m) to the 3.4 m suggested height (i.e., the "nose of Bradač") of the February 1342 flood, we receive a flood level roughly around (or slightly above) the maximum flood level of the devastating "millennium" flood of August 2002 – if we consider the 15th-century information about the water-level of the 1342 flood. While one has to be careful while using this late-medieval information because of the considerable time-lag, taking into consideration the geoarchaeological information, it fairly well agrees with the information concerning flood impacts, derived from contemporary (local) sources. Two floods are known to be notably above this maximum water-level value in Prague: the 1118 and the 1432 floods.[76,77]

Our approach to some extent differs from the discharge calculation results of Elleder,[77] in which analysis – though also listed among the greatest floods of the last millennium – the estimated peak discharge of the 1342 is not listed amongst the top 7-8 floods. An important reason for this could be that 1) In case of ice jam floods, the discharge values show significantly lower values than the real maximum water level, influenced by the blocked ice, would alone in itself suggest. The February 1342 flood was reportedly influenced by ice, and therefore it is not surprising that the potential level of "discharge" was not extreme high. 2) Elleder[77] accounted with a negligible (minor) change of the floodplain in the last half millennium (i.e., after 1500), but geoarchaeological research suggested rather notable alluviation and elevation changes that could have a significant influence also on flood levels (see more details above).

A somewhat more indirect, but potentially useful evidence on the possible maximum water level of the February 1342 flood in Prague is related to the old (Judith) and the new (Charles) stone bridge and the elevation changes along the river and the riverside area (with special emphasis on the area near the bridge and the road leading to and along the river) of Prague downtown, in the decades following the devastating flood. The Judith Bridge, as one of the most famous and largest stone brides of Europe, was completed in the 1170s following the style of such infamous bridges as Regensburg or Avignon. When the Judith Bridge was built in the 12th century, the natural terrain was lower and the flood resistance was therefore at a somewhat different level than in the 14th century. Prague went through significant changes in the 13th century with the building of the new walls, the elevation increase of the city area and the construction of the weir system in the 13th century that increased the water depth with 1-2 meters around the Bridge.[66] This latter change influenced the sedimentation, the speed and flow of the river, and could also have an impact on the bridge foundations.

With the destruction of the Judith Bridge in 1342, a temporary wooden bridge was erected, and only in 1357 the multidecadal building procedure of the Charles Bridge started.[66,78] Moreover, archaeological research identified a several meters thick artificial embankment in front of the Charles Bridge, which was built significantly larger as its predecessor. The roadway of Judith Bridge was situated 6.1 to 7.5 m above the water in the standard arches, and the first surviving arch had a roadway only about 5 to 5.5 m above the water.[78] In general, compared to the Charles Bridge, the Judith Bridge was about 3–4 m lower (and about 3 m narrower). It is probable that the greatest floods threatened the Judith Bridge with overflow. This is true at least for the arches at the bank where the roadway elevation is below the Q100 level. Charles Bridge had icebreakers that were meant to lift and break the ice, but it is not known that the piers of Judith Bridge would have also had such a protection.

Furthermore, after the 1342 flood, the areas along the river and close to the bridge were significantly elevated. Based on archaeological excavation findings, the original height of the road, leading to the Judith's Bridge from the river shore, was localized to 185.55 m above sea level,[79] while the width of the arches varied and probably increased towards the middle, similarly to the bridge in Regensburg. The embankment-related raised road, leading to the Charles Bridge, in Křížovnická Street is at a height of about 193-194 m a.s.l. and the bridge tower passage is at a height of about 195 m a.s.l.[80] This is important, because it suggest that sometime after the devastating flood (or as a consequence of the 1342 and following floods) in the downtown area around the (new) bridge and along the river the ground surface was raised with several (sometimes almost 10!) meters, and the surface level of the Charles Bridge as in level with the elevated road and riverine downtown area. The significant increase of the elevation near the bridge and the road (probably also serving as part of the embankment) suggests that, at least partly, the reorganisation of the down town area in the years, decades after the devastating flood tried to follow or even exceed the probably (maximum) flood level. It also indirectly suggests that the 1342 flood could well exceed the road that led to the Judith Bridge (and probably also overflown the Bridge itself), which again refers to back and indirectly supports the argumentation above concerning the extreme magnitude of the February 1342 compared to other outstanding floods of the last millennium in Prague.

While the Judith Bridge in Prague suffered severe destruction (from the flood, ice and debris) in February 1342, based on the contemporary (eye-witness) report of Franciscus Pragensis,[58] the Roudnice Bridge – a medieval stone bridge completed in 1340, leading over the Labe and located only 50 km downstream from Prague – withstood the pressure of the water and ice (”*Pons quoque fortissimus a venerabili patre domino Johanne quarto, Pragensi episcopo vicesimo septimo in Ruduicz efficaciter, firmiter, decenterque constructus inviolatus permansit, licet maior ibi concursus exstitit aquarum et grandior de glacie massarum impulsus.*”).[58] Roudnice is located at the Labe (Upper-Elbe) after the confluence of the Vltava to the Labe, and therefore the riverbed is already broader than that of the Vltava alone but, due to the fact that the Labe has a considerably smaller discharge than that of the Vltava before the confluence, the Vltava has a primary influence on the water-level conditions of the Labe.

Moreover, the Judith Bridge existed practically in the same form since the 12th century, and therefore – despite renovations – its structure could get somewhat weaker at the foundations (also because of the weirs that were erected long after the bridge was built, and created artificially high water-level that was intensively utilized by the milling industry), whereas the Roudnice Bridge was a newly built, strong stone structure, much shorter (less than half the length) than the Judith Bridge.[81] In addition, the Roudnice Bridge and the town of Roudnice are after/in a larger bend of the river, and at Roudnice the Labe/Elbe River has a relatively extensive floodplain north to the river (while the town is situated south to the river). This means that the flood, ice, the debris (and sediments) could extend and get deposited in the floodplain without difficulties, and the water, ice and debris did not hit directly and in its entirety the bridge like in Prague where the river flows in a relatively narrow valley (and the bridge might have even blocked the ice and debris – and with this increased the water level). In fact, no sources mention that any of the later, major historical floods – even the greatest ones – would have caused a notable damage in the Roudnice Bridge.[82] Flattening of the flood wave was also mentioned along the section after Mělník (i.e., Vltava Labe/Elbe confluence) in connection with the August 2002 flood.[76] These altogether might have been responsible for the great differences in destruction.

In conclusion, regarding the magnitude of the February 1342, the mainly impact- and destruction-based contemporary documentation speaks about extreme flood that happens once in multiple centuries. Based on the medieval, but non-contemporary quantitative (descriptive

flood mark) evidence – if we also consider alluviation over time – the flood could reach (or exceed) the maximum height of the "millennium" flood of August 2002. As the flood was clearly influenced by ice (thus, apart from the water that had flown through the Prague river-channel, also the blocking of ice and debris played a crucial role), comparison with the reconstructed discharges of other historical flood events is difficult, and only water levels can be compared. Regarding water levels, at least two floods likely exceeded the level of the 2002 flood in the last millennium (1118 and 1432).

1.1.2 Elbe flood at Dresden (and other places)

Contemporary documentary evidence suggests outstanding flood of the Elbe (caused by snowmelt, rain and ice jamming) in February 1342, fuelled by the outstanding floods of several Elbe tributaries: Apart from the Vltava, other tributaries (outside of the Czech Basin) also heavily flooded. Furthermore, the devastating flood in the Elbe estuary and Ditmarschen in the north, recorded under the year 1342, could also be connected to this major flood event. As a consequence of the outstanding flood (presumably also influenced by ice jamming), the great stone bridge of Dresden was damaged (interpretable in its late medieval environment[64,84]): Important contemporary reports (e.g., Beneš Minorite; Annales Veterocellenses: "*1342. Infra octavam purificationis corruerunt pontes in Dresden, in Praga*…."; see Supplementary Data 1, 3) connect this major destruction to the February 1342 flood. This statement differs from the reconstruction of Herget[83] who suggested that out of the two major floods of 1342, the outstanding (i.e., the greater) flood occurred in July, and the February flood would have been only great in magnitude (see Supplementary Data 1, 3).

To date, no geoarchaeological or sedimentary reconstruction is available that would suggest notable alluvial development of the Elbe floodplain in the Dresden area, and therefore it is not possible yet to calculate the difference between the modern and the 14th-century river channel and floodplain elevation. It is only known that, based on the changes in the water level and discharge curves of the Elbe gauge in Dresden due to modifications of the flood channel,[83,85] the water level and the corresponding discharge would be less than 10% lower today than before the 19th-century river modifications. Based on the available evidence and the analysis provided by Herget,[10983] we can only assume that the Elbe flood (i.e., the larger one in 1342: ca. 820 cm) might have reached or exceeded the level of a great (min. ca. Q50-year, maybe up to Q100-year) flood. We have to emphasize, however, that this is a rather relative estimation (without applicable sedimentary data that could suggest more precise information on river channel evolution and sedimentation rate between the 1342 and modern conditions).

1.1.3 (Upper-)Danube flood

The contemporary author, Johann von Winterthur, suggests (based on the contemporary report of others) that an outstanding flood of the Danube, extending roughly 2x1 miles from the riverbed, happened around the same time when Prague was devastated by flood (in 1342: "… *Danubius fluvius … in tantum excrevit, quod in longitudine duorum miliarium et in latitudine unius miliaris per sui alvei effluenciam nimiam in quibusdam partibus res cunctas et fructus universos destruxit, ut fertur, et quod de hominibus VI milia absorbuit.*"[56] See Supplementary Data 1, 3). Apart from the fact that "everything" (e.g., moveable properties) and all the harvest was destroyed by the flood, as was told, around 6000 people also died, which description – together with the extreme extension – suggests a flood of outstanding magnitude. Here the

applied terminology may also provide further information concerning the magnitude: such strong expressions contemporary chronicles usually used no more than once or twice in a century. Furthermore, in the same region (caused by the bad weather and floods) there was dearth/famine in the following 2-3 years (see Supplementary Data 3, Supplementary Information 3.1). All these information altogether approximately (with higher uncertainty) suggest an extraordinary flood event of around (50 to) 100-year return period or over.

Furthermore, the somewhat strange way of providing both "longitude" and "altitude" of the flood in the text may increase the possibility that, similar to some other major Central European rivers, this flood (apart from snowmelt and rain) was probably also influenced by ice. As Winterthur did not see and experienced the event with his own eyes, there is a possibility that the numerical information somewhat differs from the real numbers; however, it is clear from the description that the event was particularly outstanding in magnitude, and caused extraordinary devastation. Winterthur himself suggests that he participated the General meeting of the Franciscan Order held in Marseille in mid-1343, where he had the opportunity to collect recent news, even eye-witness information (including the Danube flood evidence), from other Franciscan brothers coming from different regions of Europe and beyond. Furthermore, based on his chronicle, he paid regular visits to the neighbouring regions (i.e., in the broader neighbourhood of Lake Constance), including Allgäu and Regensburg. Although Winterthur did not mention exact locations while describing the extraordinary Danube flood in February 1342, because of his general main interest areas, he quite certainly referred to the Upper Danube.

1.1.4 Seine flood at Rouen

A great flood was documented in contemporary sources on the Seine between 14 February–4 March (JC: 6–24 February (other source: 22 February in GC) at Rouen and Martainville (today a district of Rouen), in Northwest-France (see Supplementary Data 1, 3). According to the contemporary Annales S. Catharinae de Monte Rotomagensis[86] (see Supplementary Data 1, 3), the Seine flooded around Rouen, the water inundated the country road and reached the gate of the bridge ("*tantum inundavit fluvius Sequanae circa Rotomagum, quod aqua superavit calceyam de Martainville, et quod gentes desuper navigio transfretabant usque prope portam de ponte Hunfredi* …." i.e., the medieval stone bridge of Rouen). The country road called "the *Chaussée de Martainville*" (already depicted on 16th-17th-century panoramic views of the town; see e.g., *Figs. 1.1.4.1-2*),[87-89] was located east to the medieval town and the "low city" of Rouen. Nowadays, a street named "*Rue de Martainville*" is located in the city near the Seine right bank: This was the main road to get to Paris in the 14th century,[90] when the riverbank in Rouen had low elevation and some districts of the city were situated near the water, prone to floods.[87-88] This riverside part of the town and the road was elevated since then, the ground level was raised by several meters.

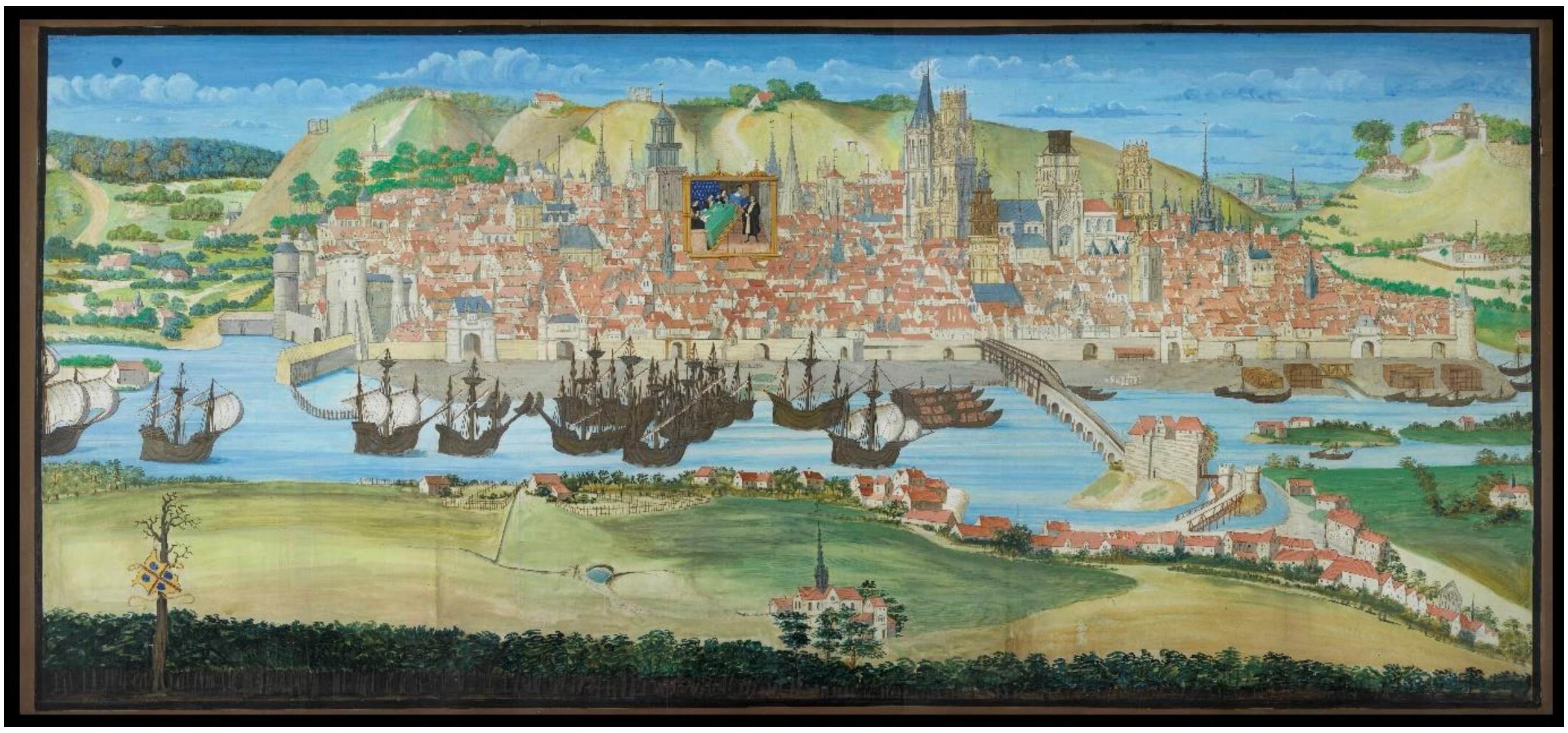

Fig. 1.1.4.1 Miniature painting of Rouen and its surroundings in 1525.[88] Note the medieval bridge in the right frontal part of the painting, and the elevation conditions around the town

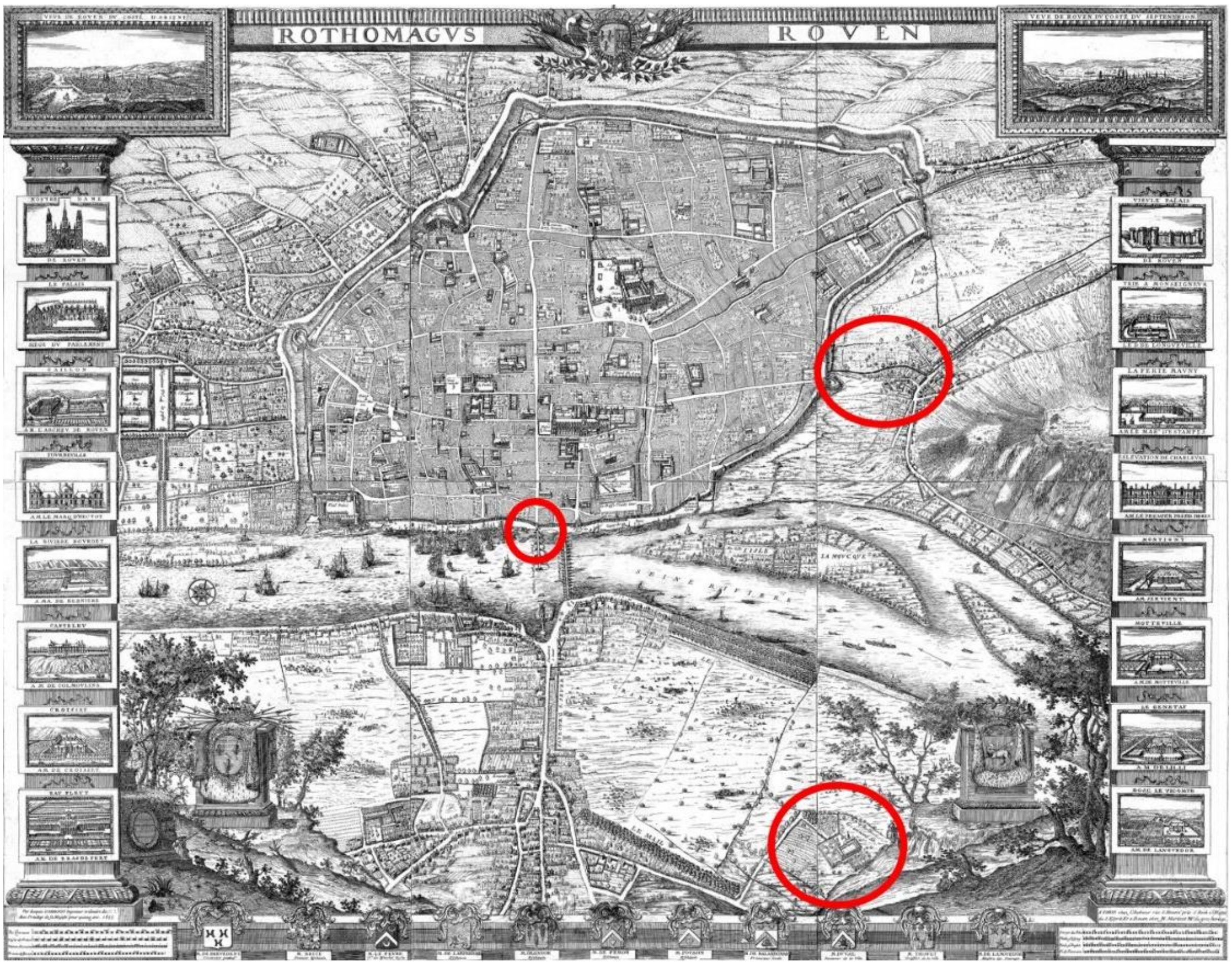

Fig. 1.1.4.2 Map of Rouen and the neighborhood in the mid-17th century.[89] The three locations mentioned to be heavily flooded in February-March 1342 are marked by red circles

More detailed description about this flood remained in the Nota Rotomagensis (presumed to be contemporary; quoted/embeded in a 16th-century work: Supplementary Data 1, 3),[86] suggesting that the flood took four arches of the bridge, many people and good perished. The source also mentions that floodwaters entered the Notre-Dame-du-Parc (Chapelle de Grandmont: founded between 1157 and 1180; raised to the level of priory with 14 monks in 1317, today the location of Parc Grammont), located outside of Rouen, on the left bank of the Seine, just upstream of the city (see *Fig. 1.1.4.2*: in the bottom right corner). The flood suddenly rose there to half foot ("half leg") in the monastery (in the cloister, refectory and other places) of the priory of "Blessed Marie de Parco", and then over that. Moreover, the water reached such heights that it was possible to enter with large planks and boats, and a boat weighing six tons entered and exited at the same time through the archbishop's gate. The mentioning of the archbishop's gate may refer to the gate at the burial place of Geoffrey, the Archbishop of York (died and buried in the monastery in 1212). The abbey/monastery itself are known to be also damaged by the floods of 1372, 1496, 1571, 1658 and 1740.[91]

The flood lasted for almost three weeks; this can be explained by a latency (response time) between the early rains and the peak discharge of the river during the flood: The city of Rouen is located at the beginning of the Seine estuary.[92] At Rouen, the river is directly influenced by tides of the English Channel and the medium water level (excluding flood periods) of the Seine varies from 5 to 8 m depending on the time of day and tide times (meaning that the official water levels for Rouen are given in relation with the tidal datum settled at –4.48 m based on French National Levelling Network, in this case 4 m for the river bank). Nowadays, during flood periods, water levels can reach over 10 m. Because Rouen is located downstream of the large Seine watershed (about 78,600 km²), just before its estuary, in France it is considered as a port city. The flood can last several days, or even weeks, before the catchment of this size is drained. The impact of tides is also important for the flood draining, and there is a possibility that the February 1342 flood occurred during the annual strong tides. Besides, the river water level was also naturally higher in the middle of the 14th century; the Seine riverbed became shallower because of an increased sedimentation rate.[93,94] Recent embankments and river flow regulation projects on its upper course, carried out in the 20th century, caused the narrowing and deepening of the riverbed (as there is no dam on the Seine from Rouen to the English Channel).[95]

Because of alluviation processes on the one side, and a significant tidal erosion on the other, active sedimentation rate in the Lower Seine river-channel was rather little in the last two millennia (did not exceed 1 meter). While in the period ca. 900 to 1200 the continental input on sedimentary patterns was stronger, between 1200 and 2003 tidal and open marine hydrodynamics controlled primarily the sedimentary evolution in the Seine estuary, suggesting a higher frequency (and intensity) of Atlantic sea-storms,[96] also influencing sedimentation conditions of the Seine at Rouen. This also means that, taking into account that, when the Notre-Dame-du-Parc (and the bridge of Rouen) was built in the 12th century, the influence of high tidal floods could be overall less frequent and pronounced than in the 14th century.

As the bridge of Rouen was largely destroyed by the flood, several human lives and an immense quantity of goods were lost, the flood lasted for weeks, the main country road was blocked that caused immense difficulties both for locals and long-distance travelers and the river even flooded caused damages in the cathedral, the flood had to be at least great in magnitude (meaning an around Q50 year or larger event). The mere fact that contemporary sources dedicated a long passage in their text to this flood in the 14th century also indirectly suggests its high magnitude, importance, destruction and relatively rare occurrence (meaning: high return period flood). Nevertheless, as the flood is not mentioned, for example, in Paris (probably meaning that the flood magnitude was lower there), and considering also the fact that

– apart from the immense damages and casualties in (probably the lower-lying part of) the city – not the entire bridge was damaged and the cathedral, although flooded, suffered not great damages, the flood magnitude was presumably not over 100-year return period (i.e. its discharge probably was under / did not exceed the 100-year return period 1910 design flood level of 10.5 m and/or 2600 m^3/s).

Today the mentioned country road is located on an elevation between 13-17 above sea level,[97] but the area of the road and the road itself was heavily filled up, meaning several meters of ground level raise. The Notre-Dam-du-Parc is situated around 7 m above sea level. Since the Seine average water level is 1-3 m above sea level in this area, but the water was at least (1-)1,5 m above the ground level in the Priory, the maximum water level elevation difference could be roughly 5.5-7 m, probably suggesting a flood of at least great (i.e., ca. Q50y or over) in magnitude.

1.1.5 Eure (Seine tributary) flood at Chartres

In the same region, probably following the intense late winter or early spring rains, in 1342 the Eure River, a tributary of the Seine, flooded heavily and caused great damages to the convent and church of the Abbey Saint-Père-en-Vallée in the city of Chartres. The lower city was also flooded and destroyed (only year is provided in contemporary source: Supplementary Data 1, 3).[98-101]

The entire city was enclosed, surrounded by town wall, by the 1340s, and the fortifications were not changed until the late 16th century (only a barbican was constructed in front of the city gates in the mid-15th cent.);[102] therefore, the 16th-century map of Braun[103] (*Fig. 1.1.5.1a,b*), regarding the town walls, and probably also the medieval church of the abbey of Saint-Père-en-Vallée as well as the affected town area presumably shows a situation rather similar to that of the 1340s. As the water caused great damages even in the church of the abbey, the water rather likely exceeded over (and/or damaged) the town wall situated close the monastic fields (see *Figs. 1.1.5.1–2*: the town wall is depicted in front of the Abbey). The great (or outstanding) magnitude is also supported by the fact that, according to the contemporary description, the medieval lower town was also flooded.

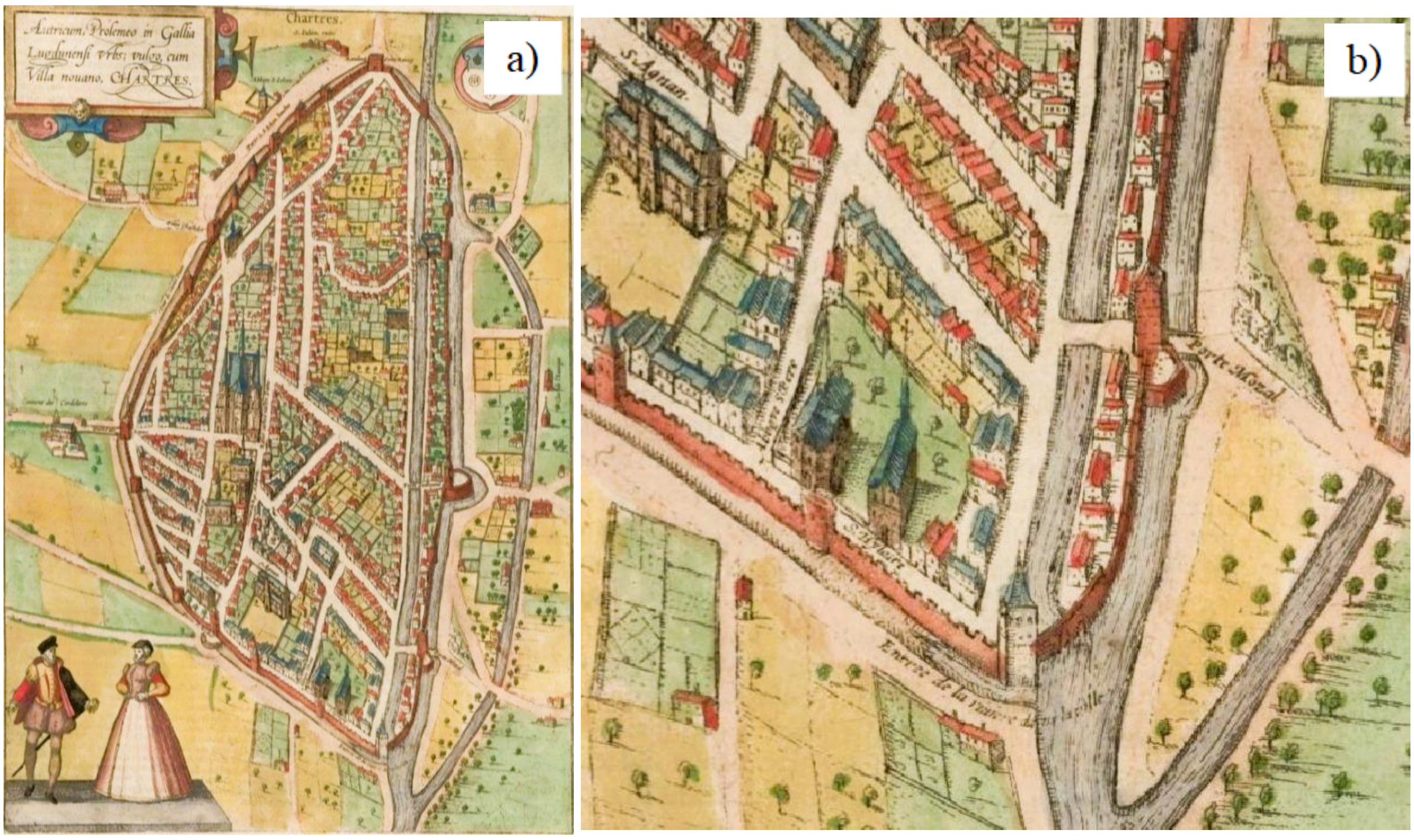

Fig. 1.1.5.1 a) Chartres in the late 16th century;[103] b) detail with the the Abbey St. Pierre (centre)

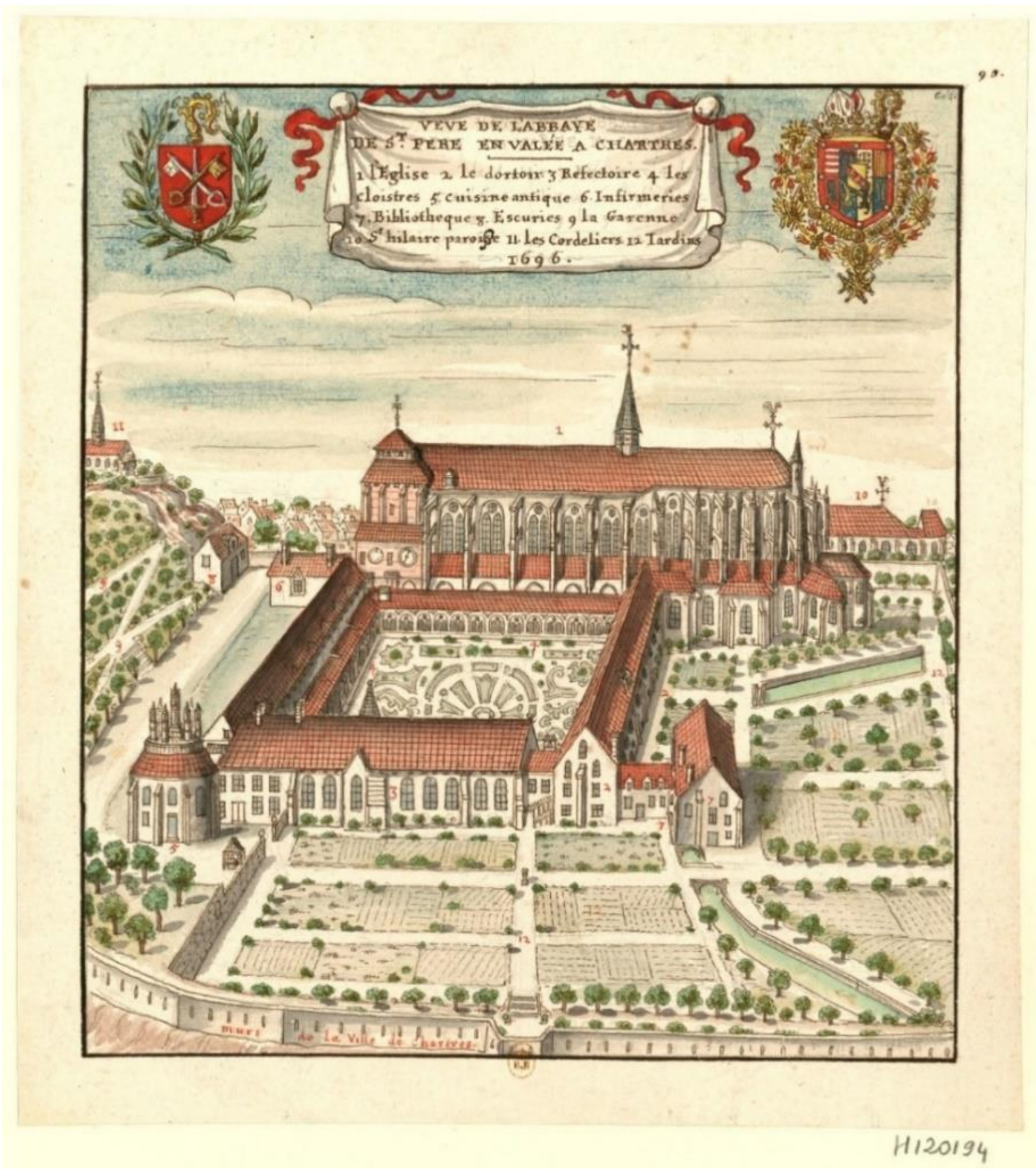

Fig. 1.1.5.2 The Abbey of St. Pierre in 1696 (view from the town moat, with the town wall – the river is situated right/east to the Abbey)[104]

Although little is known about the riverbed changes and alluviation processes of the area in the Middle Ages and after, based on modern conditions,[105] and accounting with some uncertainties in relation with historical alluviation, tectonic changes and well as human modifications, the River Eure water levels could increase with around 8-10 (or up to 12 – which means in modern conditions the water level when the floor of the entire church is covered by water) m to mostly or entirely flood the church area (not only the monastery/convent buildings). However, the river could also cause considerable damages merely by undermining the ground under parts of the building. Thus, in theory, somewhat lower maximum water level (if the flood lasted for weeks, as was reported along the Seine, in Rouen) could already cause significant damages. Based on the fact that the flood was great enough to be mentioned by a contemporary narrative, and based on mentioned extension and the approximate level of destruction, the flood had to be at least great (i.e., ca. Q20-50y or over that) in magnitude.

1.1.6 Loir flood in Vendôme

In the week of 19-25 February (JC: 11-17 February), 1342, the Loir was also in great flood at Vendôme (West-France): According to the (contemporary) local annals of the Holy Trinity Abbey, the main church/cathedral in the heart of the city, the water was so great that in the abbey it reportedly reached the height of 5 feet (1.52 m or 1.6m, depending on different feet calculations) "*Hoc p. anno* (1342)*, eddomada quinquagesime*, *fuerunt aquarum inundaciones apud Vindocium tam magne quod in abbacia omnes dicebant aquas esse magnas estimacione quinque pedum in alto*" (see Supplementary Data 1).[106]

The Loir River, an indirect tributary of the Loire, is originated from the same area (Eure-et-Loire) as the Eure: Thus, its flood primarily reflects on the precipitation and weather conditions of the same region. The modern average water level is artificial, mainly caused by the presence of numerous (113) dams along the Loir River. Founded in the 11th century, the Gothic cathedral of the Abbey is located close to the Loir riverbanks; in fact, the Abbey and the medieval city is located on a major island of the Loir. Today the average water level of the Loir is on ca. 83 m above sea level, while the pavement of the cathedral is on 86 m. As only low-resolution sedimentary investigations have been carried out so far regarding the last 1000 years in the Loir River valley,[107] we only have approximate information about the river channel conditions in the 14th century and their differences compared to modern conditions.

Sedimentary research generally suggests rapid alluviation in the Loire floodplain in Roman times and during the (high-/late) medieval period due to slope erosion, primarily related to the intensification of human activities.[108] Based on the (La Fontaine de) Montorson sedimentary profile, located along the Loir close (ca. 15-18 km upstream) to Vendôme, where the highest, 0.5-0.55 m layer was related to the period 1697-1917 (radiocarbon dating), while the next sedimentary layer 105-110 cm depth was dated to 405-208 BC,[108] meaning that the sedimentation speed was relatively low before the 18th century, and alluviation (and with this, the increase of riverbed bottom) probably did not cause a significant difference in the Loir water-level conditions between the mid-14th and mid-17th centuries.

Based on the analysis of contemporary sources, the multi-centennial Loir flood data suggest that this flood (taking into consideration the quantitative evidence of the last, almost 1000 years) was of outstanding magnitude, and regarding the maximum height the water level reached, it was clearly one of the greatest floods of the Middle Ages, if not the greatest.[109] When the flood happened in 1342, Abbot Jean de Buffa had just begun the construction of the

Gothic arches of the nave (see *Fig. 1.1.6.1–2*): During the flood, the pavement was still at the same level as the original paving of the Romanesque church, but it was raised significantly after the flood,[110,111] and the Gothic pavement became ca. 0.6-0.8 m higher than the Romanesque pavement.[109] Accordingly, the 1.5-1.6 m high maximum water level of the flood in February 1342 was measured in comparison with a notably lower pavement, which this means that the water was around 0.7-1 m high, in comparison with the later, Gothic pavement. Later, the maximum water levels in the Trinity Abbey were mentioned in two cases when comparative data are available: In 1961, when the maximum water level reached at least 0.3 m in the choir, and in 1665 – the greatest flood known in historical times along the Loir –, when the flood (based on descriptions and flood mark) reached around 1.1 m above the 1961 flood in Vendôme.

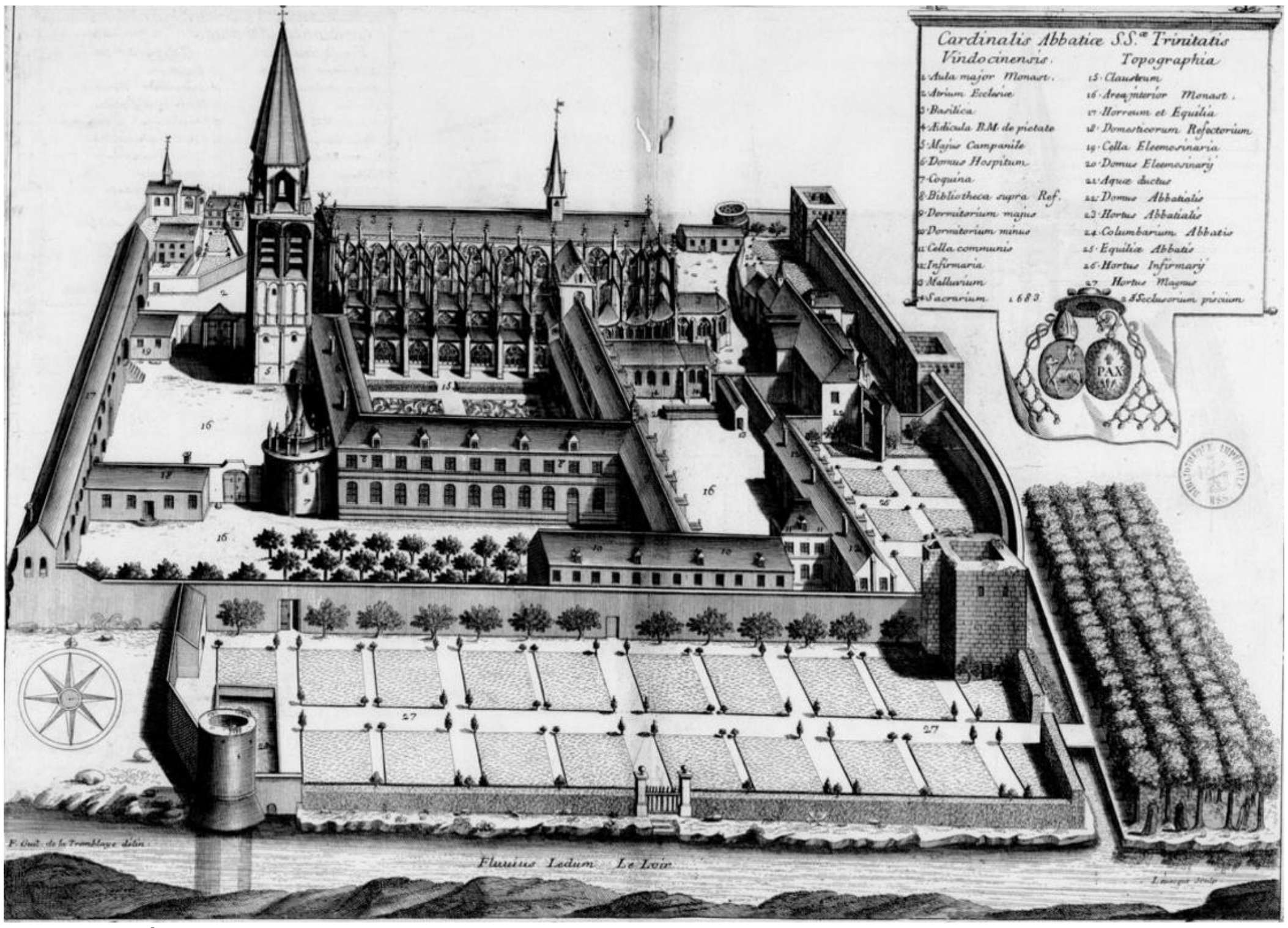

Fig. 1.1.6.1 17th-century representation of the Holy Trinity Abbey in Vendôme.[112] Note the River Loir in front

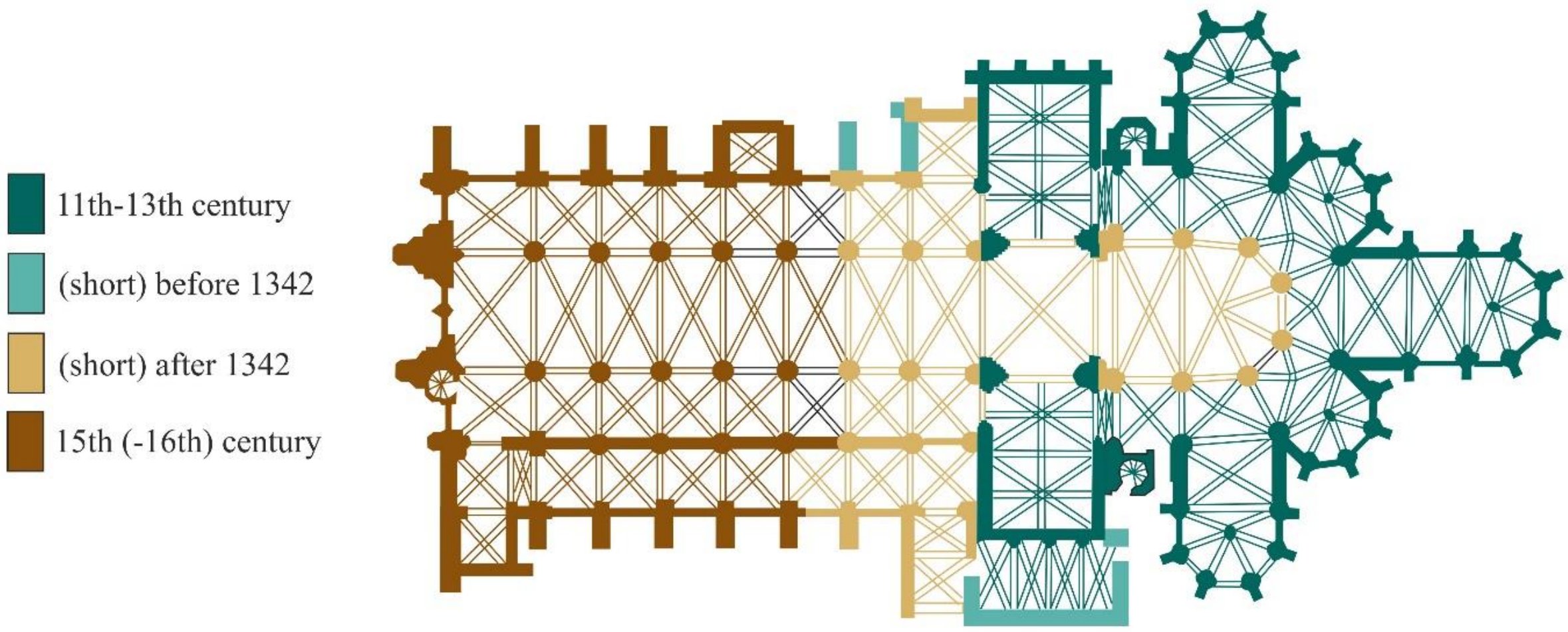

Fig. 1.1.6.2 The ground plan and building periods of the Holy Trinity Abbey in Vendôme.[113] The new, 14th-century Gothic sections of the church were under construction in 1342

Without taking into account the possible (most probably anyway) minimal differences related to alluviation and/or any unknown changes of the pavement or the building (e.g. sinking) after raising the pavement of the Trinity Abbey in the mid-14th century (after the flood), the maximum water level of the 1342 flood would have been higher by (at least) 0.4-0.7 m than the 1961 flood and ca. 0.1-0.4 m lower than the famous 1665 flood.[109] As the water levels of the 1665 flood is still today usually compared by scientific literature to the 1961 flood, and the time interval between the 1342 and the 1665 floods is roughly the same (with presumably lower sedimentation/alluviation rates), we can suggest that the 1342 flood was the second largest in the known ca. 900-year history of Loir floods (documented floods of the last ca. 900 years).[109]

Taking the 1665 flood as the millennial flood, it still means that the 1342 flood was a multi-centennial return period flood event (i.e., over Q100-200y, but lower than Q1000). As between 1342 and 1665 the river-alluviation related river-channel elevation change was most probably more positive than negative (as usually it was the case in areas situated nearby a flat terrain/floodplain, with minimal tectonic changes, and no tidal erosion influence) at Vendôme, and also considering the relatively small difference between the maximum water levels of the 1342 and 1665 floods, we cannot even rule out the possibility that the 1342 could be of higher magnitude than the 1665 event.

In the current text, we do not discuss some other cases where either less or uncertain (or no) quantitative (only qualitative) information is available regarding the floods or if the flood was largely or entirely influenced by the sea (i.e., high tide, storm/surge).

1.2 Event 6) Magdalena Flood: summer/July 1342

1.2.1 Main flood (ca. 1000y return period)

For the Main flood at Würzburg (*Fig. 1.2.1.1*) and Mainz (*Fig. 1.2.1.2*) in July 1342, in peak-discharge estimation, the empirical Manning equation for mean flow velocity was applied by Herget and his colleagues,[7,83] using the data of cross-section area and hydraulic radius of the maximum flood level, the slope and hydraulic roughness coefficient. The highest discharge of five individual elements of the cross-section area was calculated and then summed up to one value. As they assumed no effect on velocity, the authors excluded the flooded city area from the calculations.[83,114]

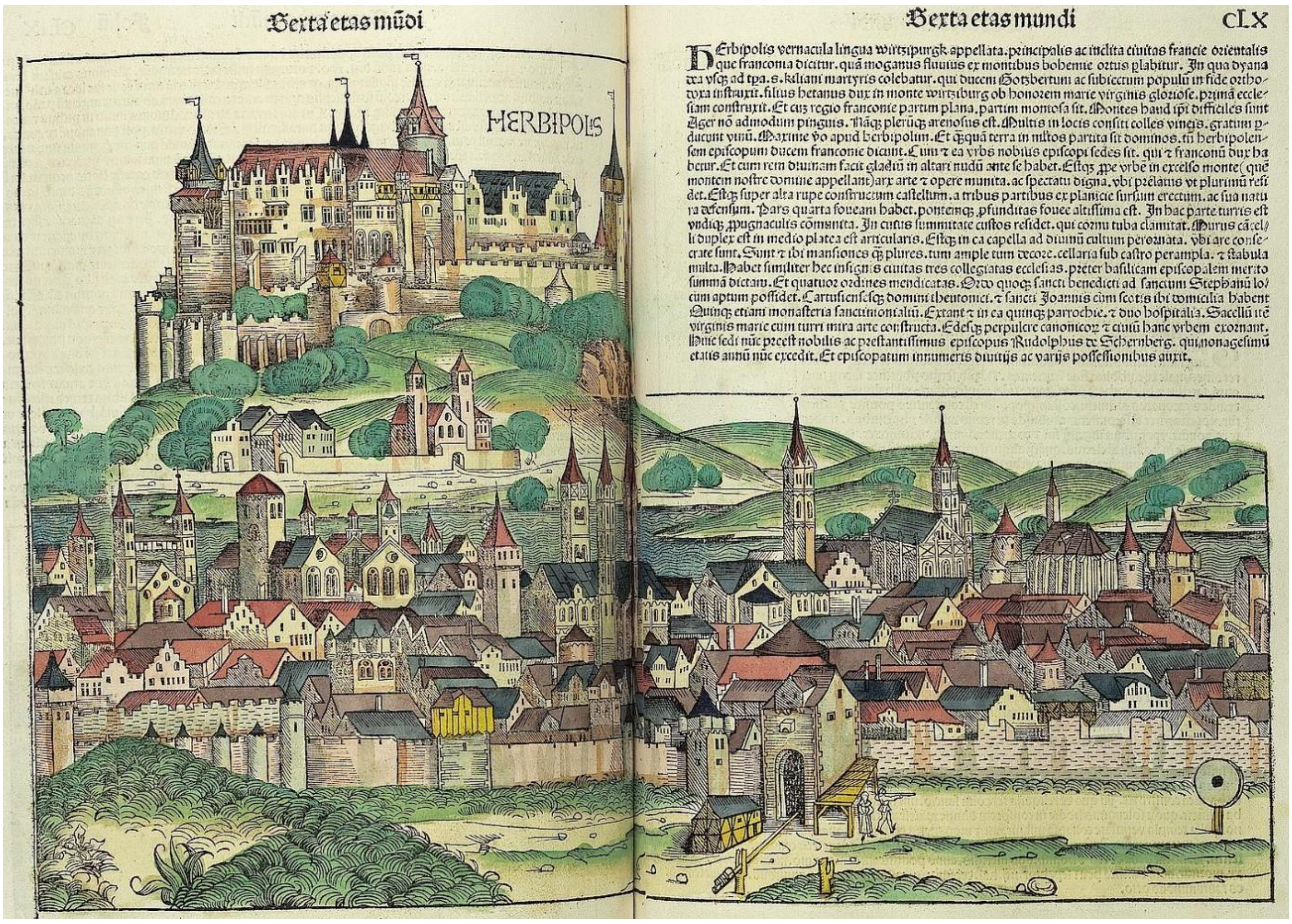

Fig. 1.2.1.1 Würzburg in 1493.[115] The River Main is presented in the central part of the drawing

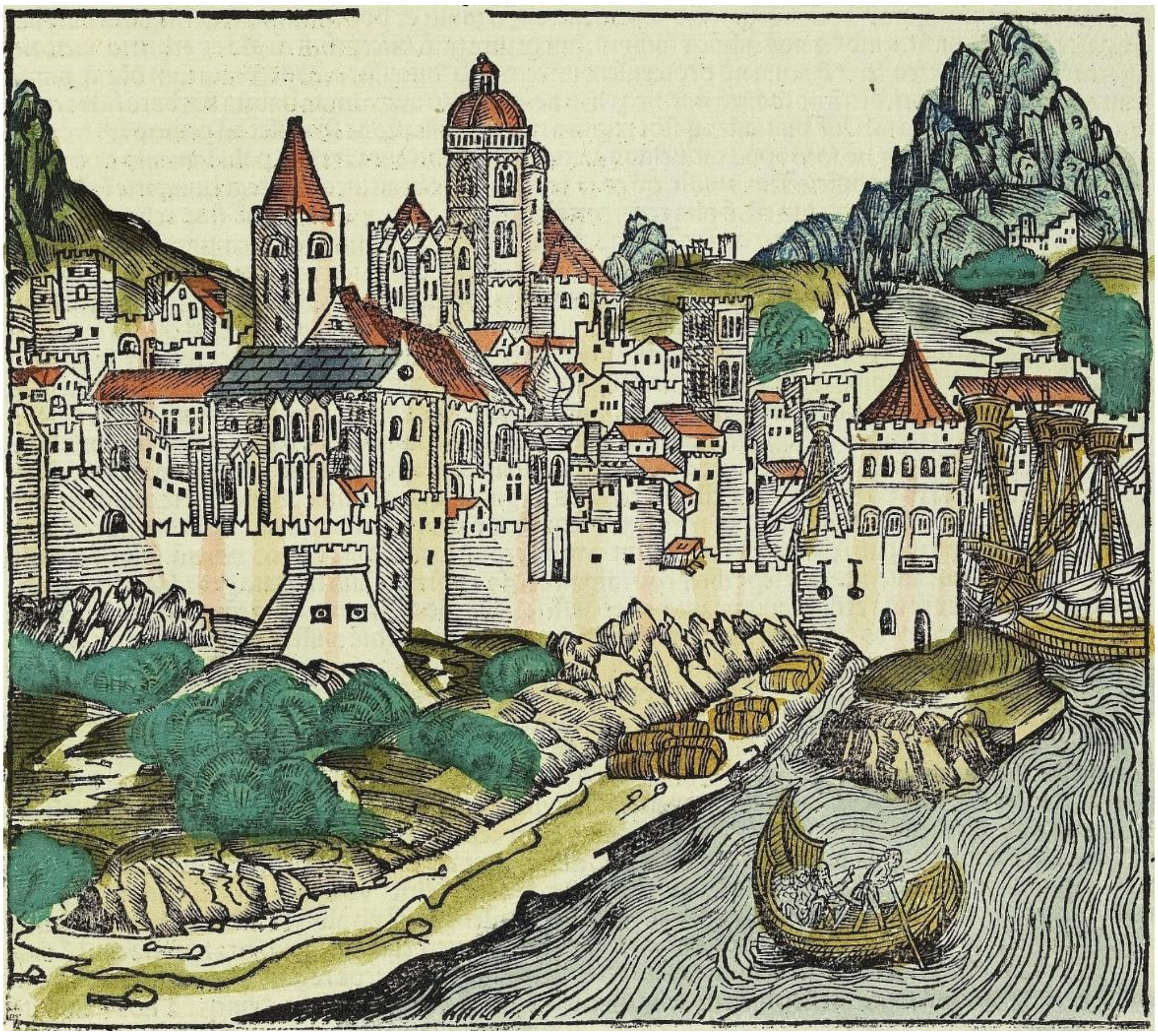

Fig. 1.2.1.2 Mainz, depicted in 1493[115]

As until the 19th century no significant river regulations works were carried out (therefore, mainly floods influenced the change of the river cross section), it was possible to utilize the historical (18th-, 19th-century) cross section profiles and maps (with clear channel patterns).[83] Regarding the Main flood in Frankfurt, similar to the peak discharge reconstructions for Würzburg, Mainz and Cologne, Herget and his colleagues[7] utilized the historical flood mark at the Eiserner Steg (98.82 m a.s.l.), and applied the riverbed and slope data available from the CMH.[116] The suggested peak discharge in late July 1342 was approximately 4300 m3/s (3700 m3/s b Q b 5100 m3/s; see *Fig. 1.2.1.1*), and with this, suggesting a higher peak discharge value than Tetzlaff and his colleagues[5] (3500 m3/s) who calculated with a simplified cross section 70 km upstream to Frankfurt (Kleinheubach) that, however, is located closer to Würzburg and therefore provides a closer estimate for the possible Würzburg discharge to that of Frankfurt.[7]

Contemporary documentary source evidence suggested the destruction of the Frankfurt Bridge and also that the water entered the church of St. Bartholomew through the Red Gate[117] (Supplementary Data 1, 3). Furthermore, one annal or chronicle (contemporary, based on the critical edition) also provided detailed description that supports the water-level information about the extraordinary height of the July 1342 flood (Supplementary Data 3): "*On the third day before Mary Magdalene's feast day, the Main was so great that the water flowed completely around Sachsenhausen* (i.e. opposite to Frankfurt; see e.g. on the Braun et al. bird's-eye-view from 1572)[87] *and in Frankfurt into all the churches and streets, except for the St. Bartholomew's Church* (note that two other contemporary chronicles suggest that the water even entered the St. Bartholomew Church), *and the churchyard around midnight that is St. Michael's, our*

Frauenberg and the Thöngesgasse, the Krämergasse (the later Markt) *from St. Bartholomew's Churchyard to the Schuchhaus, the Drechlergasse* (the later Kantengießergaß) *to the Oppenheimer house* (the later Domplatz 4) *and to the schoolmaster's house* (the later Domplatz 8)*, from our Frauwenverg to the Bornheymer gate, from the Giesbrunnen to the Ziegelgasse, from the Schnurgasse to the Gelnheusergasse, the Steingaß, Krauchengaß (now Graubenhasse) divided, up to the Beutelkisten* (the later Kränn 30) *and to our dear Drauenberg, up to the Sandhof* (the later little Sandgasse 4)*, the Sandgasses up to the Leußborn* (the Leußborn or Luseborn was in the Weißadlergasse in front of the corner house with the little Kormarkt) - *all full of water; the churches all full of water as follows: St. Nicholas church 6 feet high, St. Jacob 3 feet, the Barfüsser church 4 feet, St. Elßbet in Sachsenhaußen, to the Holy Spirit, and St. Anna, the German Lords' church, Our Lady's brothers, the church of the White Lady, each 6 feet, the Preacher's church 9 feet, to the Three Wise King 12 feet, from the so-called St. Jörg to the St. Lenhart up to under the lowest arches. .... This water had made a trench in Sachsenhausen 100 feet long, 20 feet deep, and 50 feet wide, as well as many trees and stones, and had also broken up the stone path*."[117] The last sentence is suggesting that, apart from the extraordinary high water-levels, the river also formed a new branch in the town. Note that much later, 16th-century chronicles provided different maximum height information.[117]

As for the Main at Würzburg, Herget and his colleagues[7,83] used the water-level information from four source reports taken from Bork and Kranz[118] (and the Chronik der Stadt Schweinfurt: none of them contemporary): a) the water was as high as the lithic statue above the stairs at the entrance of the Dome (174.85m a.s.l.); b) the water was only near the Dome (lowest point of the Dome 174.3 m a.s.l.); c) the water reached the porch in front of the Dome (174.8 m a.s.l.), d) the stone bridge of Würzburg (built around 1133) was destroyed by the flood (taken from contemporary sources – see also Supplementary Data 3: this reflects on the exceptionally high water levels and velocity, but also a potential additional blocking in the cross section). Furthermore, a (later, non-contemporary) flood mark on the wall of the town hall suggests the maximum water level of the July 1342 to 174.05 m a.s.l., which approximately corresponds to the elevation suggested by report c) above).

A contemporary local source (Michaelis de Leone Canonici Herbipolensis; see Supplementary Data 1, 3 – also applied in the water-level and discharge estimation by Herget and his colleagues[7,114] – describes the event in the following way: "*Of the great and previously unheard (flood) of Mogus* (i.e. Moenus: Main). */ Then also in the year of our Lord M.ccc.xl.ii., xii. kal. Augusti (21 July) on the day of blessed Praxedes, namely Sunday, or then on the vigil of Saint Mary Magdalene, from sunrise and before noon in Herbipolis (Würzburg) the river Mogus rose so much by flooding that the precious stone bridge of Herbipolis, with its towers and walls and the city itself, and also many stone houses there and around, suddenly collapsed. And this kind of flood of the Mogus waters flowed excessively over the steps of the portico of the church of Herbipolis near the first stone statues. All the wooden and stone bridges of Mogo above and below also collapsed and were broken. And then also immense damages and destruction of urban and rural occurred along (or by) the Mogus. And also in other parts of the world around that time there were unprecedented floods of water. …*"[119]

In addition, according to Herget and his colleagues,[7] the (contemporary) cultural (archaeological) layer/debris near the Dome also supported the maximum flood level estimations described above; however, the elevation of this layer contradicted to the maximum water level reconstruction of Schiller,[120] the latter suggesting almost half meter lower maximum water levels. Taken into the uncertainties, Herget and his colleagues[7] also calculated two different peak discharges, depending on whether or not the bridge (arches) caused obstruction in the river profile: Without considerable obstruction/change in the river profile they suggested a peak discharge range between 4300 m3/s (max. water level elevation 174.85 m a.s.l.) and

3800 m^3/s (174.05 m a.s.l.), or with the obstruction discharge ranging from 2800 m3/s (174.85 m a.s.l.) to 2400 m^3/s (174.05 m a.s.l.).

In comparison, Schiller[120] calculated the peak discharge value for 3300 m^3/s (±10%), while Tetzlaff and his colleagues[5] of 3500 m^3/s for Kleinheubach can also be compared with the values for Würzburg. Finally, because of the uncertainties, Herget and his colleagues[7] suggested for the peak discharge an interval of 2600–4700 m3/s. Thus, based on the reconstructed cross-section information, estimated roughness, differences in sedimentation and the changes in riverbed profiles compared to recent conditions, Herget and his colleagues[7,83] estimated the Main flood for a 1000-year return period event. Compared to the historical data applied by the latest and most comprehensive water level and discharge reconstruction,[7] we also know from contemporary sources that, on the day of Maria Magdalena, the floodwater flow over the town walls both in Reinburg and Würzburg (*Chronici Saxonici Continuatio Erfordensis*: see Supplementary Data 1, 3).

At Mainz, where the Main enters the Middle-Rhine, Herget and his colleagues[7] used two sources, one (the 15th-century *Eberbacher Chronik der Mainzer Erzbischöfe*) suggesting that the water was man's belt high in the Dome[121] while another, non-contemporary (early modern, but continuously written by Carthusians since the 14th-century?)[122] chronicle suggested 3-feet deep water in the same building, meaning in both cases a roughly 1 m deep water (elevation: ca. 88 m a.s.l.).

Based on all known estimations and discharge calculations, the July 1342 flood of the Main reached the highest known water levels in all the three locations where quantitative information was available since 1200 onwards. Related scientific studies[4,5,7,83,114,118] all suggested that the July 1342 Main flood was an approximately 1000-year return period event. In addition, taking into the uncertainties in the descriptions and the potential influence of blocking in the riverbed, Herget[83] and then Herget and his colleagues[7] suggested lower and upper thresholds to their discharge estimations, which also define the lower and upper uncertainty thresholds of the estimated return period.

1.2.2 Rhine flood (ca. 100y return period)

The maximum water level and discharge estimation of the Middle-Rhine flood in July 1342 was carried out using the same methodology as in case of the Main (at Frankfurt, Würzburg and Mainz), especially utilizing the data available in Cologne (and Mainz) where reconstructed historical river channel cross-section is also available.[7,114,123] Based on the available documentary evidence and the long-term flood reconstruction and analysis of Middle-Rhine floods at Cologne since 800, the July 1342 flood on the Middle-Rhine at Cologne was estimated to a 100-year return period event.[7,114] Here one has to mention, however, that no contemporary, only later source evidence is available that provides more definite, quantitative maximum water-level information about the flood in Cologne, and therefore the uncertainty of maximum flood level is also higher than in case of the Main.[7]

As significant human intervention in the river profile occurred only after the late 19th century, in historical times relatively little change of the river cross-section occurred on the Middle-Rhine at Cologne,[114,123] even if outstanding flood events could cause some change in the riverbed profiles (e.g., in 1342, 1374, 1497 and 1595).[114] The flood with the estimated greatest discharge in the last almost 700 years occurred in 1374[7,114] – since the estimated cross-section profile in 1342 was presumably not significantly different from that of 1374 (i.e., the estimated difference between 1374 and 1895 is also rather small), roughly the same riverbed

parameters could be employed in the water-level and discharge reconstruction regarding the 1342 as the 1374 floods. Flood frequency curves (with 95% confidence intervals) and reconstructed return periods of more recent historical discharge investigations[123,124] show no significant difference compared to the reconstruction of Herget and his colleagues[7,114] The extreme high, 1000-year flood levels suggested by previous literature[7,125] was erroneously dated to 1342 (the estimated discharge is close to the highest present-day flood discharge of 11,200 m3/s) instead of 1374 (estimated peak discharge: ca. 23,000 m3/s) – the latter flood reached the highest water levels of the last millennium, even if the 1342 flood could be also of extraordinary magnitude on the Middle Rhine.[114]

1.2.3 Weser (Werra, Fulda) flood in Hannoversch Münden

At the confluence of the Fulda and the Werra, three flood marks are known that mark the maximum water level (or "wet mark") of the July 1342 flood: The first one is clearly a much later mark (at the Packhof),[126] while another, one of the earliest known fixed flood marks of the July 1342 event (carving in one stone of the wall) is available in H. Münden (Germany), still standing in the town (probably in its original location/position), marking the approximate maximum level of the outstanding flood of the Weser and Fulda Rivers: the stone in the wall of the St. Blasius Church is located on 2.1 to 2.5 m (compared to the contemporary surface/pavement.[126] The building was renovated in the late 15th century: It is unknown whether or not there was any change in the location of the stone (cannot be verified) but, taking into consideration its community functions, there is a good chance that the stone is still in its original position).[127,128] In the late 18th century another July 1342 flood mark at the Tanzwerder Gate, still visible (and mentioned) in 1790, was described by a contemporary author, suggesting a 5 ell (ell could be between 0.5 m to 0.8 m – most frequent in Germany: 0.54-0.57 m) probably meaning a ca. 2.70 to 2.80 m height (compared to the contemporary surface).

Based on the current conditions, this would mean an around 8 m rise of the water level. While it is possible to know the elevation of the medieval ground level and that of the mentioned buildings, little is known about the 14th-century river channel conditions and elevation,[126] historical sedimentation (e.g., increased floodplain elevation?). River regulation works since the 19th century (e.g., moderate deepening?) could influence the riverbed of the Fulda, Werra and the Weser itself in the area of Hann. Münden (interpretable in its late-medieval, early-modern historical river environments[64]). However, as the Werra bridge, built in the 1220s (as well as the medieval town wall) still exists today, to some extent, it is also a reference to the medieval water level and riverbed conditions. As the historical/collective memory preserved the 1342 flood as the greatest known (with also marking the height of later floods), it is rather possible that the return period of this flood could reach or exceed 500-600 years.

In other cases, the quantitative information is too limited or only qualitative information is available, therefore we can only provide index-based threshold (i.e., 2=great or 3=outstanding) information regarding flood magnitude.

1.3 Event 13) late July–early August 1343 floods

1.3.1 Flood of the Upper Rhine at Strasbourg

Amongst others, the Strassburger Chronik of (the contemporary chronicler) Fritsche Closener describes[57] the great flood of the Rhine in 1343 (also copied by the near-contemporary local Jacob Koenigshofen[129] Supplementary Data 1), in Strasbourg: the flood was so great as had never been seen or heard before ("*Do man zalte 1343 jor, do wart (der) Rin also groß und ging also sere us, daz nieman do zemol lebete der üt gedohte oder ie hette gehoret sagen, daz er so groß würde*").[57] There were, in fact, two floods: The first great flood started four days before St. Jacob's and ended 4 days after the feast (JC: 21-29 July; the current Event 13). It caused significant damages, amongst others, in the town wall and the nunnery. The second, even greater flood occurred a month later, during the week around 24 August (21-28 August in JC; see Event 14).

Fritsche Closener[57] (see Supplementary Data 1, 3) describes the flood in the following way: "*Das große waßer. / Do man zalte 1343 jor, do wart (der) Rin also groß und ging also sere us, daz nieman do zemol lebete der üt gedohte oder ie hette gehoret sagen, daz er so groß würde. Dazselbe waßer det ouch vil schaden an den ringmuren zů Strosburg, und erschraht ouch die klosterfrowen die (p. 133) ußewendig der stat in den clo(e)stern worent, daz sü nüt truwetent zů iren frunden untze daz daz waßer wider ingefiel. dis waz umbe sant Jocobes tag* (25 July) *in der ernen, vier tage dervor und 4 dernoch. do dis waßer verlief, in demselben summer, vier tage vor sant Bartholomeus dage* (24 Aug.) *3 dernoch, do kam aber ein also große waßer, alse mans ie gesehen hette zů Strosburg, one daz neheste dovor. wan daz vorder waßer was wol zweier schůhe hoher oder anderhalbes schůhes wan dis hünderste. donoch maht man die ußer ringmure von Utengaße untz an den turn und den turn dermitte, also do vor geschriben stot, do man zalt 1346 jor.*"[57] Accordingly, the flood of the Rhine in late July was so great that "*no one who had ever lived before or ever heard*" it happen: It damaged the city wall, and flooded the area so much that the nuns outside the city (most probably referring to the Convent of St. Mary Magdalene) had to escape to another convent. This flood lasted for at least 9 days, and was 1.5-2 feet (=46-61 cm) lower than the second, even greater flood that happened roughly a month later (Bartholomew: Event 14). Thus, apart from the quantitative information regarding the difference between the late July and late August floods, no direct quantitative (quantifiable) data is provided in the source regarding the maximum flood level.

The fact that this flood was mentioned in such detail by contemporary authors, the town wall was damaged and also that the sisters had to escape from the St. Mary Magdalene convent as it was not suitable for habitation for several days, already suggest that the flood was at least great in magnitude. The 13th-14th-century Mary Magdalene convent, in the modern Place de la République, was located in a relatively low-lying area, close to the former river branch transformed into the medieval town moat.[105,130]

In the historical iconographic representations and maps of Strasbourg the river flowing into the city center is the Ill River (e.g., in 1572).[87] Active branches of the river inside the town are not those of the Rhine but those of the Ill, running across Strasbourg from north to the south (some lateral branches have been filled up over the centuries). The branches of the Ill entering the town were in direct connection with the Rhine; moreover, Rhine water also directly entered the moats around the town.[87] The 1343 flood in Strasbourg is reportedly linked to (only) the Rhine, which river was clearly (and only) blamed by the contemporary and near-contemporary sources for causing the flood. Although the River Rhine has always flowed outside the city, it had to be much closer in the 14th century to the eastern walls of the town than it is today (*Fig.*

1.3.1.2). The current ground level elevation of the city is around 135 to 141 m above sea level (a.s.l. – according to NGF), while the former elevation of the riverbanks is around 139 m a.s.l. that corresponds to the Q100 flood event (maximum flood level of the 1882, 1999 floods), while the Q1000 level corresponds to 139.6 m a.s.l.[131,132]

Given that, based on the remaining sections of the high-medieval town walls followed the usual standards (i.e., they were at least 6-7 meters heigh over the medieval terrain, with embankment/notched walls),[133] the maximum flood level had to reach a notable height also along the wall, meaning an at-least 3-4 m increase of the water level compared to average conditions. Based on long-term, Holocene (and pre-regulation) sedimentary analysis, the magnitude of sedimentation in the area was low (i.e., under 1 m; also due to the floodplain conditions) between the 14th and 20th centuries. This makes medieval topography and flood conditions generally comparable in the broader area of Strasbourg,[134] even if there is clearly a difference between the medieval and modern ground level conditions because of the cultural layers exceed medieval town level.[133] This, however, does not seem to have major effect in the area of the Mary Magdalene Church (the area of the 14th-century convent). These data altogether suggest that generally the flood could be close, reach or exceed the Q100 level; this is also supported by the communal memory information (on never-seen or heard flood),[62] also approximately suggesting that this flood magnitude was not observed (at least) for ca. 100 years.

1.3.2 Flood of the High Rhine between Basel and Lake Constance

The same flood heavily hit Basel and the High Rhine in late July: Heinrich von Diessenhofen (1302-1376),[59,135] a clergyman living in Konstanz around that time, mentioned that on 25 July (JC) in 1343 all bridges between Rheinau and Breisach were destroyed by the devastating flood on the Rhine (Supplementary Data 1), indirectly also referring to an extraordinary, destructive flood at Basel. Johann von Winterthur gives partly similar information, but even more details: the flood was caused by excess rains, and the flood was so great that it destroyed all bridges over the Rhine between Schaffhausen and Strasbourg (“*Praeterea eodem anno citra festum sancti Jacobi propter inundaciones aquarum exortas et causatas propter ymbrium supra proxime memoratorum excessum a Schafhausa usque ad civitatem Argentinensem omnes pontes Reni fluvii propter ipsius inswetam magnitudinem et inpetum furiosum dirupti et abstracti sunt cum hominum non parva desolacione.*”).[56] Thus, his description rather agrees with that of Heinrich von Diessenhofen; this also means that Basel

(and its bridge), even if it was not mentioned separate, according to both contemporary reports, had to be heavily affected by the great 1343 flood event (Supplementary Data 1, 3).

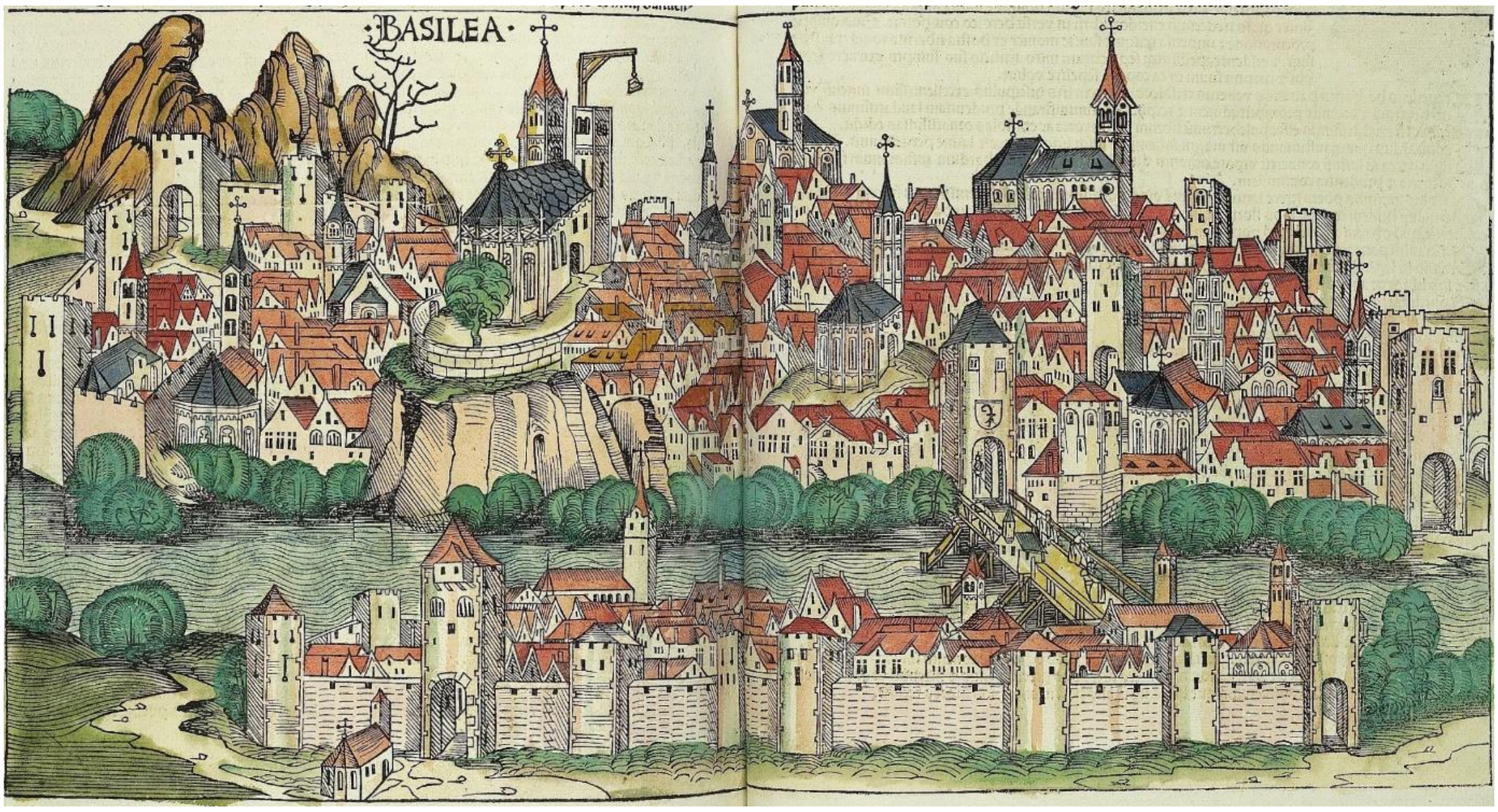

Fig. 1.3.2.1 Graphical representation of Basel in 1493.[115] Note the bridge in the right (central) part of the drawing

In Basel, 15th-century (medieval, but non-contemporary) sources, namely the greater and the smaller Basel chronicles both mention the destructive flood; however, only the Schnitt manuscript version of the greater Basel chronicle mention it under 1343; the other two dates the event to 1339 and 1340.[137] All these descriptions generally mention the destruction of bridges (and buildings, villages) in the area by the Rhine (the smaller Basel chronicle does mention also Basel separately), meaning that also the – at that time wooden – bridge of Basel was damaged/swept away (see e.g., *Fig. 1.3.2.1*).[115,136] Based on this (and other High-Rhine flood-related) information and the historical-environmental background, in the 800-year water-level and discharge reconstruction of the greatest floods on the High Rhine at Basel, although erroneously under 1342, based on historical, archaeological, sedimentary data and the medieval town structure and particular buildings including the medieval Gate Tower and the wooden bridge, Wetter and his colleagues[61] listed this event among the greatest floods of the last millennium, even if with higher uncertainty regarding the interval of the possibly highest water level of the flood (see *Fig. 1.3.2.2a,b*). As the High-Rhine flood related sources mention only one flood and only referring to the year, based on this information it is not possible to detect whether this information reflected on the July or the August flood (or both).

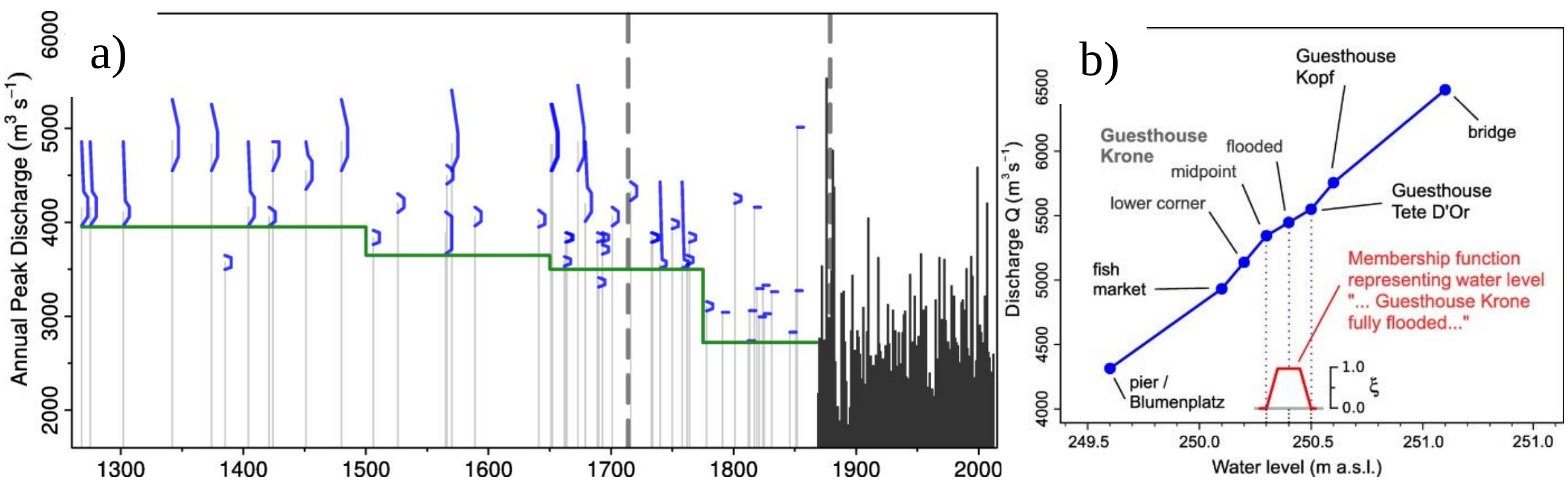

Fig. 1.3.2.2 a) Discharge reconstruction of Wetter and his colleagues[61] regarding the historical floods of the Rhine in 1256–1867, at Basel. Blue colour indicates membership functions, black discharge values in the systematic measurements period.[55] Green line: perception threshold, vertical dashed grey lines: river regulation/diversion. b) Rating curve (blue line) at Basel, cross-section Rheinbrücke.[55,61]

Similar type of information is available on the High Rhine regarding several other settlements whose bridges and buildings were destroyed, people and goods swept away. Out of these settlements, regarding magnitude information, the example of Säckingen[136] should be also mentioned, as charters issued in the next years about great (e.g., the Hungarian widow queen's) donations for the significant rebuilding and the restructuring of the town, after the devastating flood, which information indirectly support the extreme magnitude of the flood(s). Regarding Säckingen, Johann Winterthur mentioned that the water flowed over the town wall.[56] As the town walls were significantly rebuilt or transformed into a stronger defence line – also adding the Gallusturm to enforce the most easterly point of the town directly facing the strength of water during flood – after the 1343 floods,[138] no direct information is available about the height of the medieval town wall, built in the (mid-)13th century. However, Central European parallels (and the information about the Konstanz town wall: see Event 14) suggest an approximately 7 m (plus/minus a couple of meters) height with most probably notched walls, meaning that the water could, in theory, enter ca. 6-6.5 m over the ground level. As the sources do not provide details on the timing of the maximum height, this information most probably refers to the higher, late August 1343 flood event (which was both in Strasbourg and Konstanz higher than the late July flood: Event 14).

1.3.3 Flood of Lake Constance

In the description about the extreme late July flood of Lake Constance, an educated and also internationally well-connected Franciscan friar living in the Lindau Franciscan Friary (*Fig. 1.3.3*), Johann von Winterthur,[56] as an eye-witness mentioned rather important details: The flood that arose in late July was so outstanding that outside of the town the lake flooded vineyards, fields (i.e., arable), pastures and ditches. Inside of the town houses, squares and streets, entire quarters were flooded ("*... lacus Potannicus tantum excrevit, quod extra oppidum Lindaugense vineas, agros et prata et fossata, intra vero domos et plateas et vicos occupavit.'*"). Moreover, the water entered the residences, the garden/yard and the workshops of the Franciscan monastery ("*Anbitus quoque fratrum Minorum illic residencium et viridarium et officinas subintrans fedavit*."), and even entered the Franciscan church ("*Nichilominus lacus, quod ex oblivione fere transilivi, fratrum prefatorum ecclesiam subintrans*.").[56]

Furthermore, the water did not merely enter the church, but it also sprang up from the pavement, causing the “feeling of loathing” to pray in the church, in their usual places (“*Nichilominuslacus, quod ex oblivion fere transilivi, fratrum prefatorum ecclesiam subintrans et a pavimento erumpens hominibus horridum et swalidum locum et habitaculum oracionis dedit.*”).[56] The water “boiling” secretly in the basilica generated a stench that was deservedly to be feared, which they distanced themselves from, as has been said, lest they inhale it by breathing and infect their hearts. Because of that, the brothers first went to the *porticum* (i.e. entrance area outside the church) and then moved to the *podium*, the upper church where the holy mass is usually celebrated/provided (“*Unde nauseam inde orandi ibidem concipientes ad exteriora, scilicet ad porticum, et ad superiora, videlicet super podium ecclesie, quod in vulgari superior ecclesia dici conswevit, se contulerunt, ...*”).[56] Generally, the water reached further than ever imagined before.

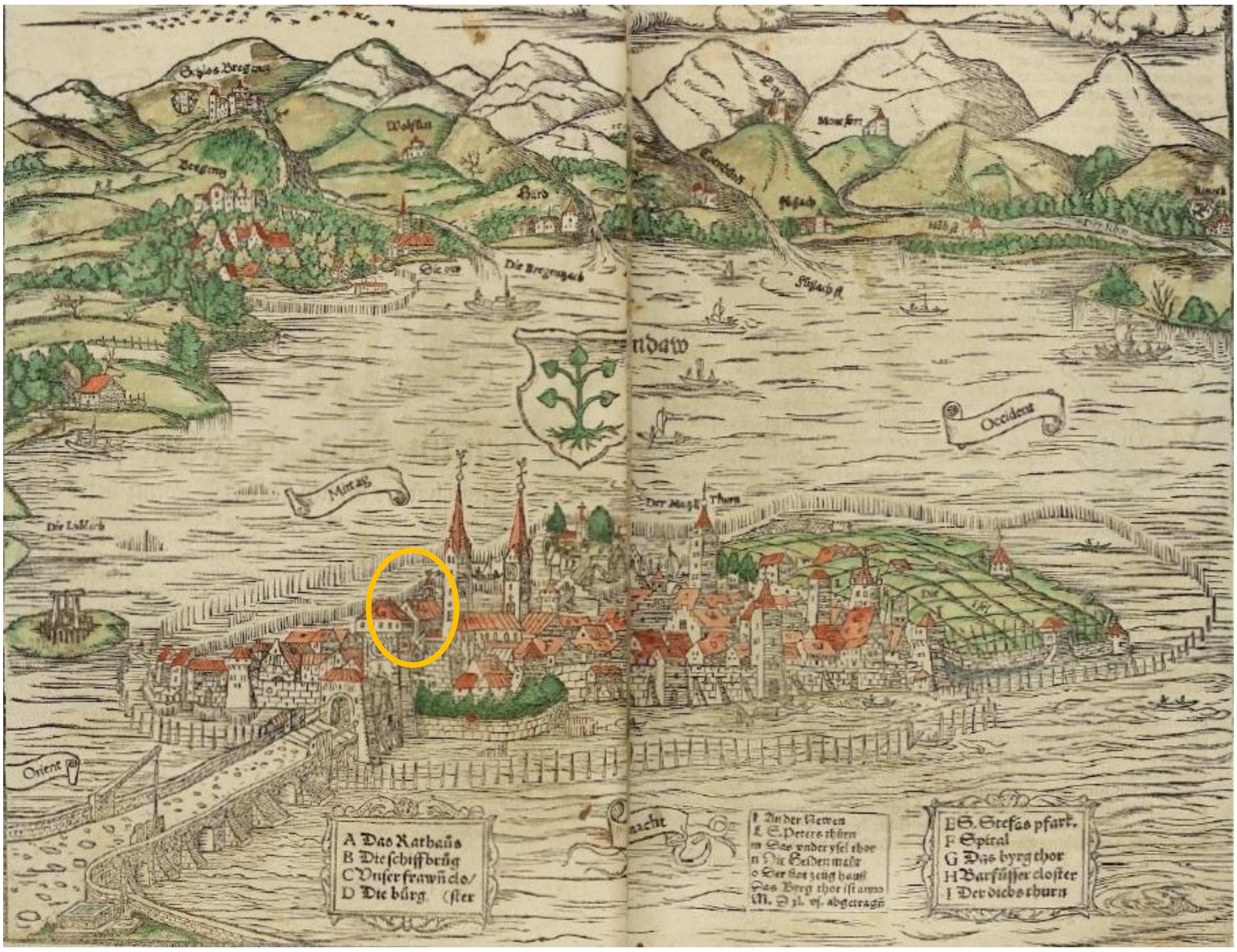

Fig. 1.3.3 Panorama view of Lindau and the eastern basin of Lake Konstanz, from the north, before 1550 (Barfüsserkirche marked by orange ellipsis).[139] Note the original Alpenrhine, Bregenzer Ach and Fußach inflows, the Lindau town walls (comparable to those of Konstanz), and the agricultural fields on the smaller, western island of the town that, similar to the town, was surrounded by stone walls

These sentences help us identifying an approximate maximum water level: as we could see, the water flooded agricultural areas (even vineyards and arable lands) outside of the town, and streets, squares and houses inside the town. The water did not only flood the Franciscan friary buildings,[140,141] but also entered the church and extended up to the stage where the mass was celebrated. Whereas the first set of information (flooded agricultural lands, streets and squares, buildings of Franciscans) already suggest that in hydrological sense an extreme flood event (beyond the level of a Q100 flood) happened (for comparison, see *Fig. 1.3.3.1*), the more

specific information on the maximum heights of the water level in the Franciscan church may provide some evidence on an even greater event, discussed later in more detail.

Based on the modern HQ100 and HQextreme estimations,[142] in the south-eastern part of the town and specifically the area of the Franciscan Friary, today the Q100 flood would not reach the area of the Friary, while the Qextreme, roughly fitting the maximum extension of the 1817 (the "greatest known ever") flood. In the latter case, part of the area of the Friary would have been already covered by water, but not yet the close area and not most of the church itself – however, this is typically a situation when the ground under the church could be already so highly saturated that it reached the surface layers and (especially if there were burials under the church pavement, as was often the case in the Middle Ages), after a while (some days or weeks), even if the floodwater has not yet physically entered the church itself, could cause the filthy, unhealthy smells Winterthur wrote about. At the end of the description, Winterthur finally mentioned that the water also entered the church: as based on the text this could happen the last, after the period of smells, rather possibly it happened when the lake water level again considerably increased. As we only know about one more case when this happened, namely in late August, the direct flooding of the church (when the friars could only use the upper church) could happen during the second, late August flood.

Qextreme[142] do not cover the church area and, based on the comparison of historical and modern pictorial evidence, the church area did not go through any significant change, or great (i.e. over 1 m) ground level/pavement rise (and also considering the lake sedimentation-related changes over time: see Event 14). Therefore, we can suggest that, while the late July flood probably reached the level of the 1817 flood (with the living quarters and working areas already flooded at that time, and with near-flooding situation of the church), the late August flood reached beyond that, and probably extended to a level we can call is Q1000. Q1000 is realistic, because an even greater (e.g., Q10000) could happen only when the street north to the church would have been mentioned as flooded. As that street and the terrain over that are naturally located notably higher on the slope (increasing in elevation towards the centre of the island), the silence about the further flooding of that area gives no reason to think that the floodwater even reached those areas (especially, because the height difference between a Q100 and Q1000 event is hardly over half a meter: i.e., 58 cm between the 1999 and the 1818 flood).

1.3.4 The flood of the Limmat at Zürich – ca. 200-500 years

Johann von Winterthur mentions that around the same time when the extraordinary floods occurred on the Töss, the Reuss, the Rhine and Lake Constance, there was severe flood on the Limmat river at Zürich ("*In Thurego fluvius Lindimacus supra modum eciam tunc temporis excrescens mangna pericula et nocumenta et angustias hominibus intulit*.").[56] Since the river has its outflow at Zürich (e.g., *Fig. 1.3.4*),[139] the lake itself had to be in flood at that time, too. About the great flood in Zürich on 25 July, the annals of Zürich (the part between 1336 and 1364, written by Eberhard Müller) gives even more information: the water was so great in Zürich that it flew over the bridges, and over the Sihlfeld (i.e. the Sihl was probably also affected; see e.g. the 1642 Merian-view of the town[145]), the bridges (with people?) were damaged, everything was in water ("*ûf sant Jacobstag, dô wärent diu wasser ze Zürich alsô gros, das es über die bruggen gieng und über das Silfend, und muost man die bruggen beschweren mit trotböumen, mit stainen und mit zubern voll wasser*").[141] He also mentions the house of Götfrid Müller at the lower bridge that has been driven by the water onto the bridge and when people tried to take it off, the bridge got also broken and both were taken by the water; and the mills of Zürich were all taken by the water ("*Des selben mauls ran hern Götfrid*

Müllers hûs ûf der nidern brugg ze Zürich in der nacht hin weg, und gestuond an der brugg in dem Hard bi dem durn; und dô man das hûs anfieng schlisen, dô brach diu brugg und gieng das hûs und diu brugg mit ainander enweg. Es runnert ouch des selben mauls die müllinen ze Zürich alle enweg unz an zwô die behûob man mit nôt.“).[143] The 15th-century (ending with 1478) chronicle of Zürich further suggests that man could enter the Frauenmünster by ship (*Figs. 1.3.4*).[144] Apart from the details described above, Winterthur provides some general, additional information: many different and special damages occurred in various places due to this flood event (“*Plura et diversa dampna et discrimina, que in diversis locis contingerant, propter alluviem iam factam essent enarranda. Que quia nimia indigerent prosecucione, hinc ab eis abstineam.*”).[56]

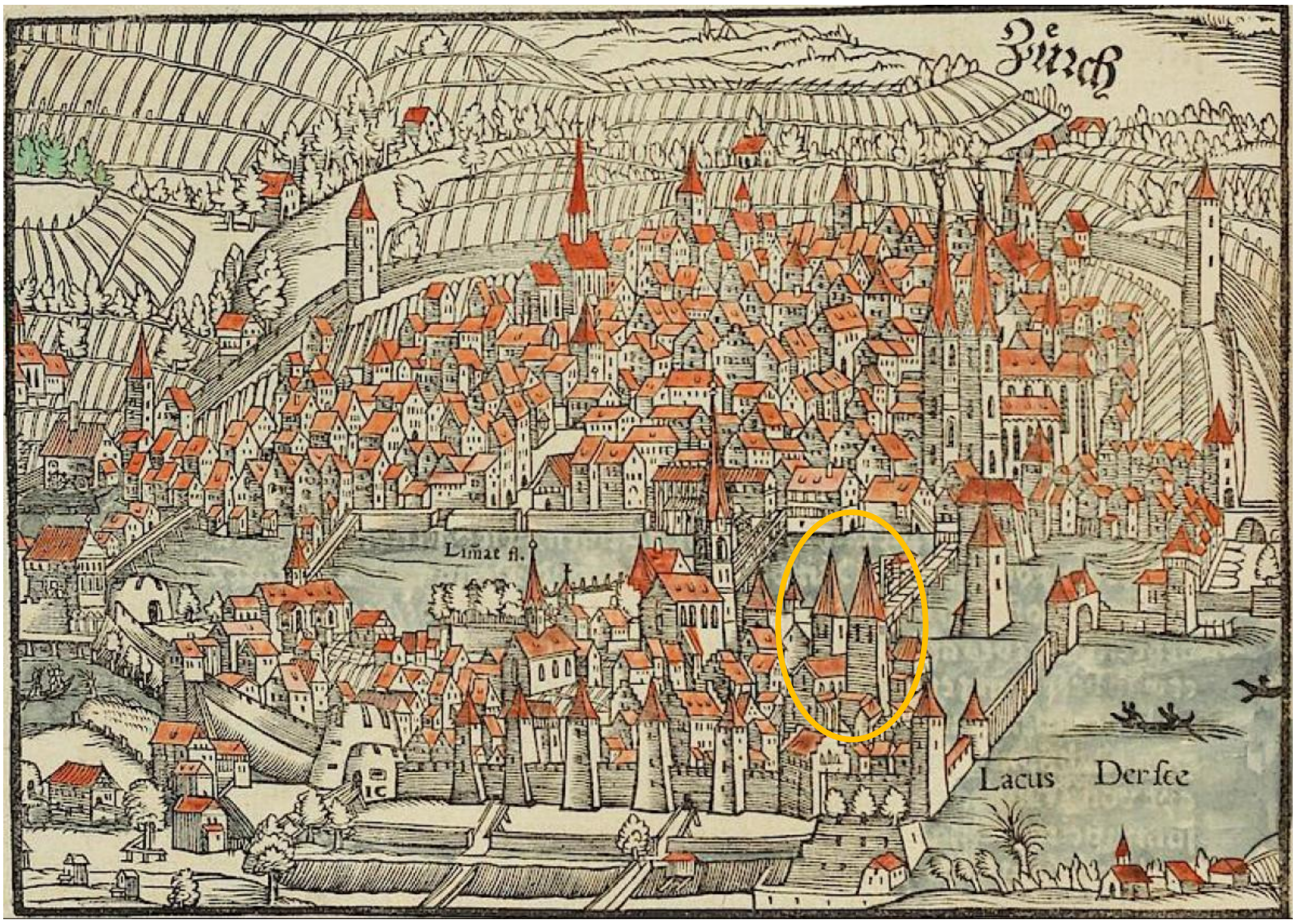

Fig. 1.3.4 Zürich around 1550. Note the wooden bridges over the Limmat, and the Frauenkirche (marked by yellow ellipsis) near the Münsterbrücke, affected by the 1343 flood(s)[139]

Regarding the Limmat flood (Lake Zurich), Wetter and his colleagues[61] suggested that the 1343 flood was amongst the 9 greatest floods of the last ca. 700 years: providing a magnitude threshold, they identified 5 floods this period that belonged to around the same magnitude threshold (1343, 1553, 1579, 1664 and 1720), and only two floods of the last 700 years were suggested to be similar or greater (i.e., with similar or higher water level) than the 1343 flood (1511, 1566).[146,147] Based on the estimated changes of riverbed/channel cross section of the Limmat at Zürich, the return period and corresponding maximum water level data, and the estimated/reconstructed historical maximum water levels (and/or thresholds), the flood of July 1343 (even with the recommended corrections) clearly reached a flood level well exceeding the 300-year return period (and also the EHQ).[147] In general, taking into consideration the available evidence, sedimentation and river-regulation information,[147] the maximum water level of the July 1343 Limmat flood was presumably among the three highest ones in the last over 700 years when quantitative data on the greatest floods are available.

1.3.5 Flood of the Reuss and the Vierwaldstättersee az Luzern

Johann von Winterthur mentioned a great flood, caused by immense rainfalls/downpours ("*tempore estivali in una excrescencia aqurum excessiva propter ymbrium immensitatem.*")[56] on the River Reuss at Luzern (*Fig. 1.3.5*), and on the Töss near his birthplace, Winterthur (Supplementary Data 1). The Reuss (leading away the waters of the Vierwaldstättersee) at Luzern was so heavily flooding that the water reached the great altar in the Franciscan church. Winterthur admitted that such a strong flood has never(!) been seen or heard there ("*...fluvius Ru(i)sa oppidum Lucernense preterfluens tantum excrevit, quod in choro monasterii fratrum Minorum illic residencium altari maiori apropinquavit, quod a retroactis annis forte nunquam visum vel auditum fuit.*").[56] And the water was so high and vehemently flowing towards the altar that it could have taken a weighted ship(!). The Franciscan church in Luzern (built in the 1270s-1280s) is located on app. 436 m a.s.l., while the medium water level of the river today is on approximately 430 m a.s.l. The water had to be deep and strong enough to carry a weighted ship/boat, but the altar stayed still visible. This means that, just calculating very roughly, the water level in the church had to be at least a meter deep, but probably deeper (see e.g., the case of Rouen). This would mean a minimum 7 m increase of the water level compared to the medium level, which would show parallels to the flood of Lake Constance at Konstanz in late August 1343.

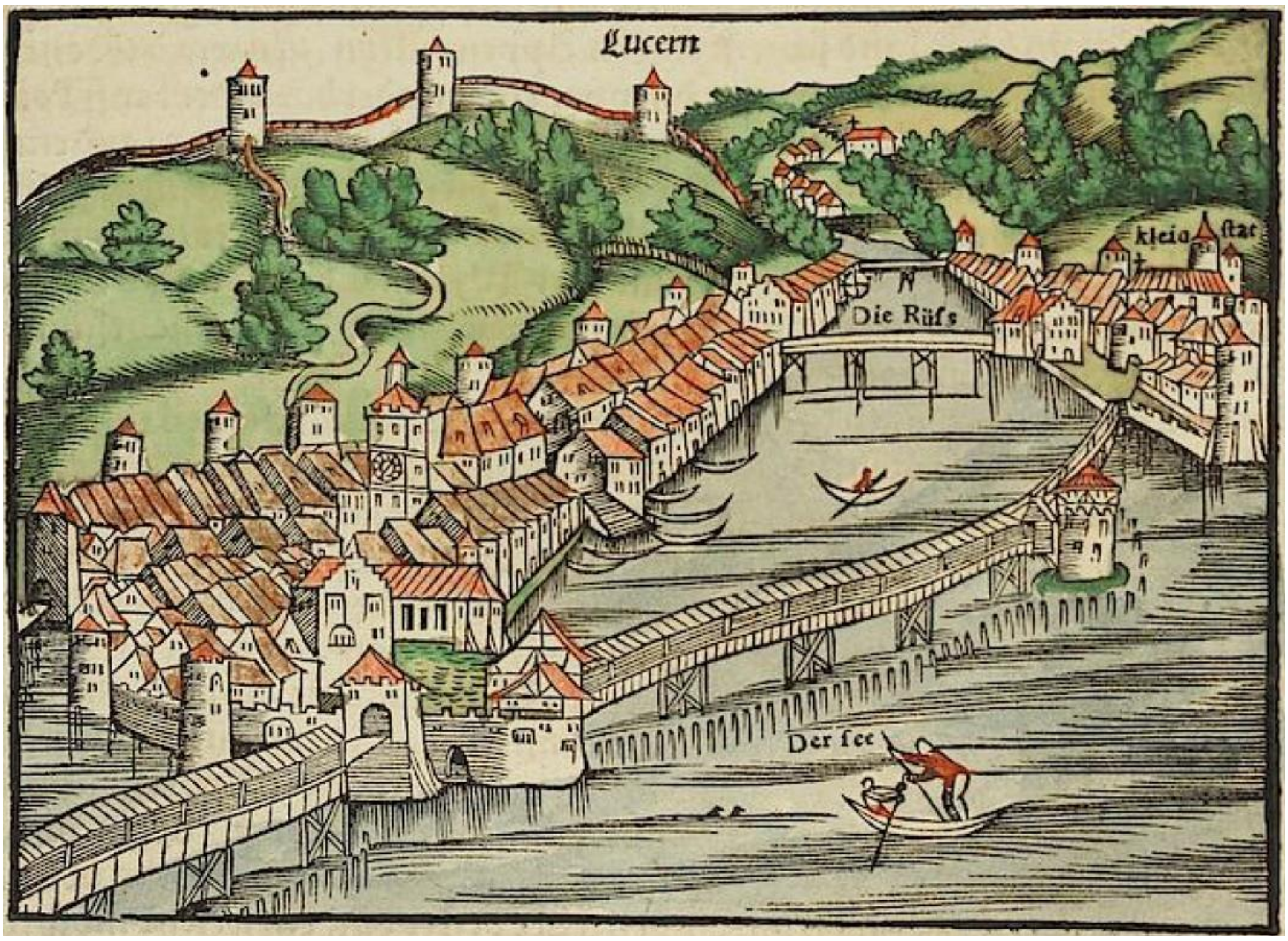

Fig. 1.3.5 Luzern in the early/mid-16th century[139]

Sedimentary evidence in Central Switzerland, dated to ca. the mid-14th century, in several cases were connected to the 1343 event (Supplementary Data 1b). A thick sandy flood layer – immediately under the layer associated with the 1356 earthquake – was identified in the sedimentary profiles of Urnersee (northern part of Lake Luzern/Vierwaldstättersee), and

similarly in the Baldeggersee (located between Luzern and Zürich) and Lake Lauerz (south to Lake Zürich) all located in Central-Switzerland, in the area, based on Winterthur, most hit by the late July 1343 heavy rainfall and outstanding floods (see Supplementary Data 1b). In all of these cases the sedimentary layer, associated with the 1343 flood, is among the most important flood-related layers in the sedimentary profiles.

1.3.6 Flood of the Töss at Winterthur

The Töss, described by Johann von Winterthur as a normally small unnavigable small river, extended so much that it entered the Dominican nunnery in Winterthur (the birthplace of Johann von Winterthur). The sudden flood resulted in an unexpected high level of damages and frightened the nunnery and its inhabitants: such a great destruction caused by flood has not been remembered in human memory (i.e. since time immemorial) ("...*To(e)sa nuncupatus tantum inundavit, quod unum cenobium monialium coherens sibi cum inpetu pertransiens iacturam maximam intulit, immo quasi per suum augmentum more torrentis rapidissimum cenobio et habitatoribus eius minabatur excidium, a tempore, quo non extat memoria, insperatum.*").[56] The Töss Dominican nunnery at Winterthur was located (and still it is located) right at the bank of the River Töss (detailed representation of the former monastery are available from the 17th-19th century).[148,149] The elevation differences between the medium water level of the Töss and that of the monastery, this means a present water level at least (3-)4 m higher than medium level to threaten the inhabitants of the monastery with major destruction.

In both cases the author (originally coming from the same area) mentioned floods whose magnitude exceeded human memory. According to the investigations of Fanta and his colleagues[43] society keeps the memory of an outstanding event on average for two generations. Two generations is a rather approximate interval that can mean in the Middle Ages around 50-70 years. However, "communicative memory", the form of collective memory that is based on everyday informal communication, "living memory", within a group – as happened in the village/small town communities in the Middle Ages – typically covers a time span of three interactive generations, meaning about 80 years. The "living memory" fades as it is no longer reinforced by direct conversation with those who experienced it. Unlike cultural memory that can keep the memory of events for hundreds of years, it does not rely on monuments, texts, or specialized professionals to be maintained.[62]

1.4 Event 14) Lake Constance, High- and Upper-Rhine

1.4.1 High-/Upper-Rhine flood

After the Jacob Flood, severely affecting Strasbourg, the Strassburger Chronik of Fritsche Closener[57] dates an even greater, extreme Rhine flood event to 21-28 August 1343 (in Julian Calendar): this second flood, ”never-seen by people”, was 1.5(-2) feet higher (“*in demselben summer, vier tage vor sant Bartholomeus dage 3 dernoch, do kam aber ein also große waßer, alse mans ie gesehen hette zů Strosburg, one daz neheste dovor. wan daz vorder waßer was wol zweier schůhe hoher oder anderhalbes schůhes wan dis hünderste.*”); the town was full of water and the water took away the bridges (for the late medieval–early modern townscape and riverscape, see *Fig. 1.3.1.1*). The same contemporary author also added immediately after the description of the extreme floods that the outer (circular) walls around the town were built after this flood, in 1346.[150] The timing and the textual contexts suggest that likely the additional circular wall could partly or mainly serve (amongst other defence functions) as an additional protection against floods.

Even if under 1342, Wetter and his colleagues[61] lists this High-/Upper-Rhine flood at Basel among the greatest floods of the last millennium, but – applying a wider maximum water level/discharge threshold – also points on the higher uncertainty of the (and other medieval) available quantitative data. This is also due to the fact that only very limited amount and less detailed contemporary or near-contemporary information is available about this flood. This also means that regarding the High-/Upper Rhine flood levels during the 1343 floods, we have information from its Bodensee outflow (Constance), from most of the settlements along the High-Rhine and the Upper-Rhine downstream to Strasbourg and in Strasbourg itself.

1.4.2 Flood of Lake Constance

On 27 August, three days after the feast of Saint Bartholomew, the clergyman Heinrich von Diessenhofen[59] living in the town of Konstanz, mentioned the (repeated) great rise of the water level of Lake Constance/Bodensee: the water increased over (i.e. overflown) the town walls near the Fish Bridge (the small bridge leading to the island of the Dominican Friary). He also noted that the water level of the Lake had not been seen or reported to be so high before (“*Item tercia die post festum beati Bartholomei anni predicti lacus ascendit murum civitatis Constantiensis iuxta pontem piscium quo itur ad Predicatores, quod antea non est visum, ut antiquiores tunc temporis referebant.*”).[59]

Based on Diessenhofen's report[59] (and to some extent to the “even-greater” water levels of the Rhine in Strasbourg) the late August flood could be even higher than the late July flood described by Winterthur. However, Winterthur does not provide much information about this flood event: he briefly mentions the ”flood of rains” that “has been briefly previously listed above,” were renewed (occurred again) and caused great destruction. In his work,[59] Heinrich von Diessenhofen in Konstanz only talks about one extreme, “never-seen” flood of the Bodensee, and he dates it to 27 August (and not late July). The fact that in Strasbourg there was an extreme flood in late July, nevertheless, allows and may even support the idea that another flood peak could be observed in late August on the Bodensee (i.e.: here also 2 flood peaks would have occurred). A further problem is that Heinrich von Diessenhofen describes the late August Bodensee flood as the “greatest ever”.[59] While the first information regarding the overflown town wall provides quantitative information about the (maximum) water level of the

flood, the second data on the memory of (minimum) return period provides descriptive (but with some uncertainty quantifiable) information.

Presented in Event 13 in more detail, collective memory usually provides information for approximately 3 generations, covering a time span of ca. 80(-100) years. Thus, based merely on this information we can suggest that roughly from the middle of the 13th century, no such a great flood happened in Konstanz. However, as the author also referred to earlier times (i.e., the "ancients" or "olders") probably meaning the times before collective memory (e.g., written reports), he also extended his time-spam "estimation" to the level of cultural memory that can already mean even hundreds of years. Naturally, this only means that neither in "living" (i.e., collective) memory nor in any sort of documented memory he/they found information about this flood (i.e., subjective estimation/statement, strongly dependent on the available resources). This altogether may pinpoint on an event that exceeds a time-spam over a century, at least (but can as well be several centuries or more) – however, this information is much more uncertain and subjective than the minimum time estimation based on collective memory.

As for the medieval city walls of Konstanz (*Figs. 1.4.2.1–3*), the town walls, overflown by water in August 1343, were built in the second half of the 13th century, and in remained the protector of the town without significant transformations/changes until the early modern/modern period, when the town walls were either destroyed (and the stones utilized) or built into houses and still today serve as house walls – some sections of the town wall preserved in an intact form until today. In 2023, because of planned construction works archaeologists monitored and excavated an over 28-meter section of the medieval wall that remained intact in later house walls (btw. Bodenstraße and Hussanstraße, near the late 14th-century Schnetzthor):[151] The wall foundation was surveyed to the depth of 396.50 m above sea level (a.s.l.), where the bottom edge of the wall has not yet been reached. The wall rises to a height of up to 7.5 m above ground level (consistent with the typical height of high/late medieval town walls in other German-speaking areas and North-/Central Italy), with the section above the walled-up battlements visible in the western and eastern parts consisting of modern additions. The highest point of the wall is situated at approximately 407 m a.s.l.,[151] while the ground level is therefore at 399.5 m.

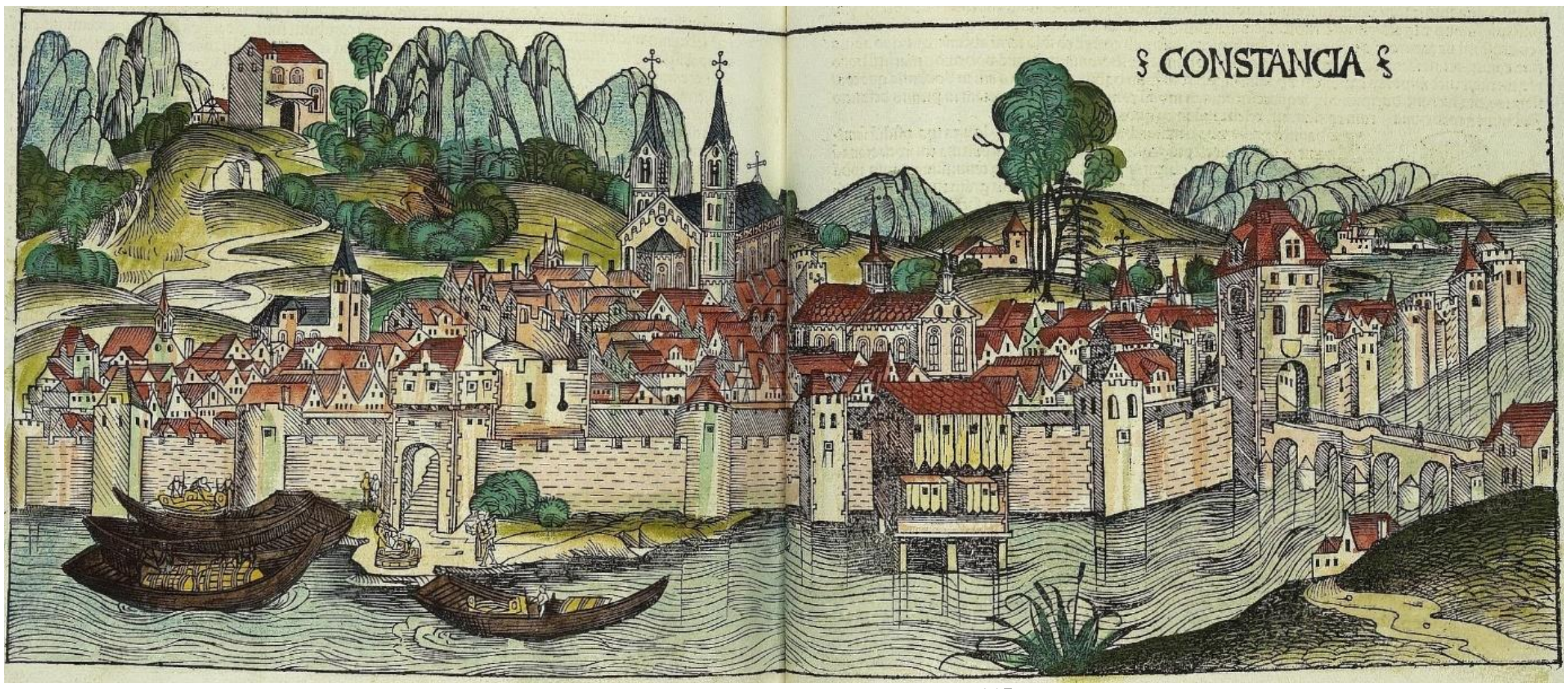

Fig. 1.4.2.1 Simplified panoramic view of Konstanz in 1493.[115] The Dominican Friary (where the floodwater exceeded the town wall in 1343) erroneously presented as a wooden house over the water

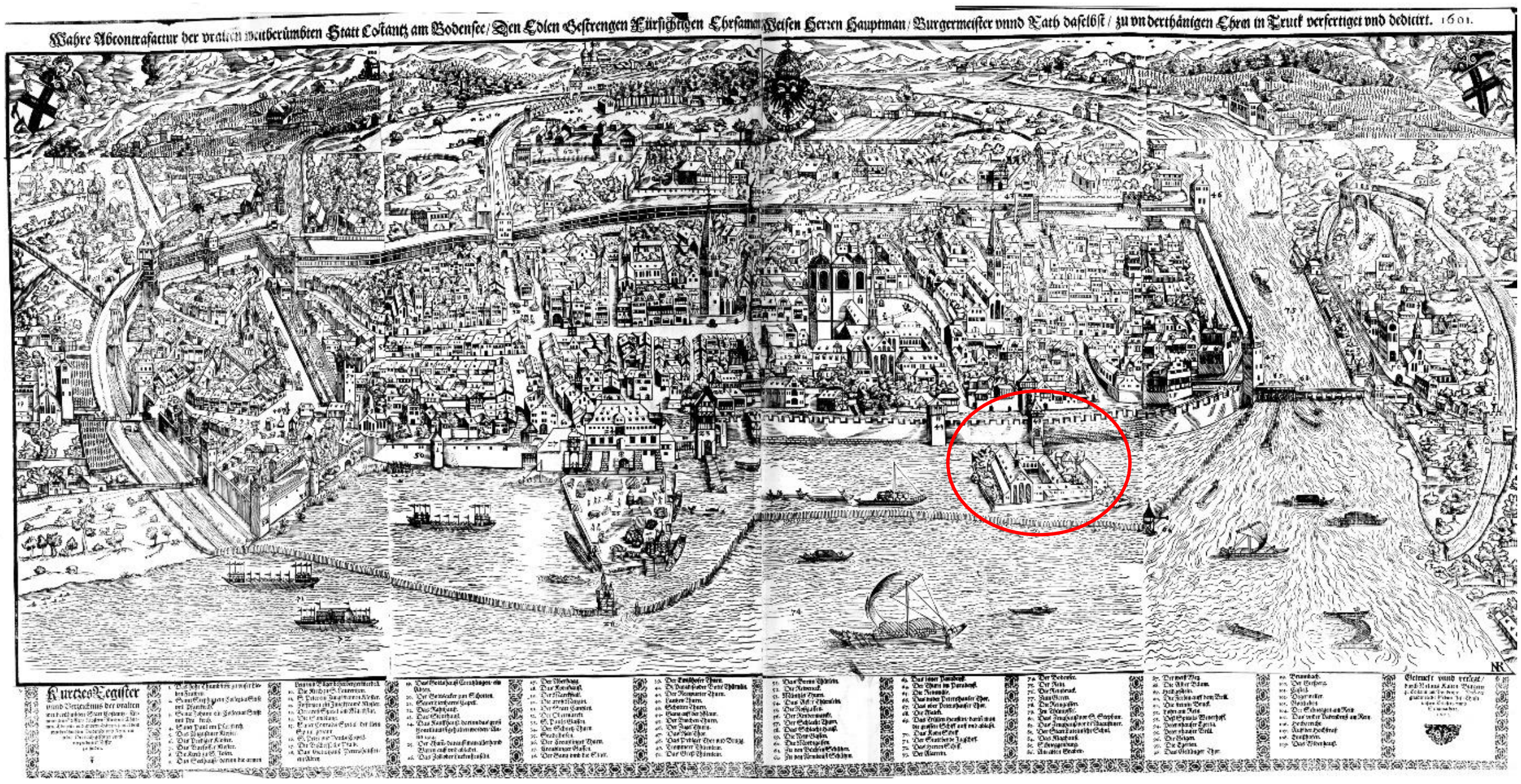

Fig. 1.4.2.2 Detailed bird-eye view representation of Konstanz and its immediate surroundings around 1600.[152] Red ellipse highlights the Dominican Friary of Konstanz. Note the notched/battlemented town wall (similar to 1493) running along the water and around the town (the recently-monitored/excavated wall section is located at the left-central, upper part of the image)

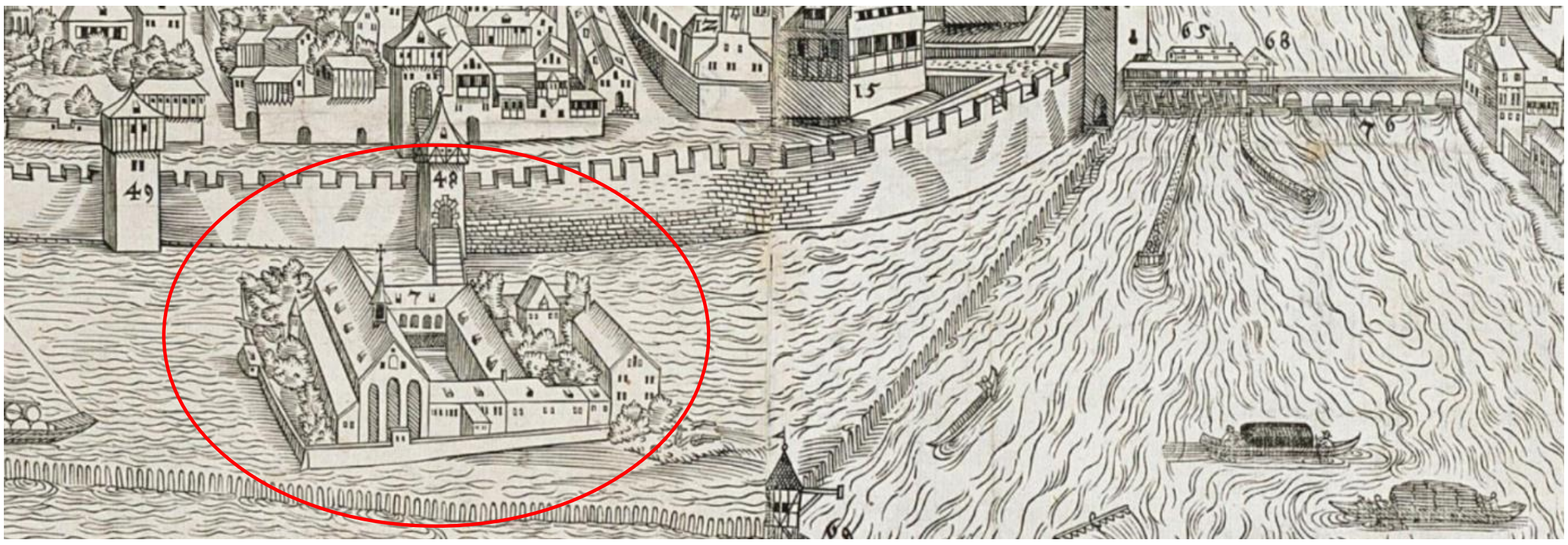

Fig. 1.4.2.3 Detail of the bird-eye view representation of Konstanz from 1600[152]: the medieval Dominican Friary (marked by red ellipse) of Konstanz in the Untersee, the Old Rhine Bridge with the Seerhein, and the town wall section where the water overflowed in August 1343

Regarding the August 1343 flood at Konstanz, the source also specifies that the water overflowed the wall only at a particular section, namely at the Fish Bridge leading to the (island of the) Dominican Friary (see *Figs. 1.4.2.1–2*). This means that all parts of the town wall, except for the section at the island of the Dominican Friary were higher than the floodwater (including the waves). Even if the town wall was not necessary (much) smaller at the lakeshore than near the Schnetztor, because of the elevation difference, the ground level of the town wall along the Lake near the island of the Dominican Friary could be situated several meters lower than that of the town wall near the Schnetztor. So, probably we are not much mistaken when suggesting that the ground elevation of the shoreline wall was near the lake surface level (i.e., ca. 395 m a.s.l.), and if the height of the (notched) wall was approximately the same as at the Schnetztor, and (as presented on the picture), the maximum elevation of the town wall could be around 402.5 m. Moreover, as presented in the images from 1493 and 1600 (*Figs. 1.4.2.1–3*), in order

to flow over the top of the notched wall, in theory, it could be enough if the water reached a ca. 0.5-1 m (the app. height of the crenel or notch) lower height of the town wall. This means that a maximum flood elevation (in case of a 7.5 m wall) could be 401.5-402 m a.s.l. However, there is also a possibility that the overall height of the wall along the Dominican Friary was somewhat lower than at the Schnetztor.

On the panoramic view of the town from 1600 (*Figs. 1.4.2.2–3*) the maximum height of the town wall, right along the lake (or even reaching the water), seems indeed somewhat lower, even if none of the known historical images of the town suggest a really significant (i.e., meters of) height difference among the different sections of the town wall, but 1-2 m lower wall tops are possible. Accounting for the "worst-case" scenario, this still means that the overflowing water reached an, at least, 399.5-400 m a.s.l. elevation. If we consider that the known highest maximum water-level flood event, the July 1817 flood, reached 6.23 m, which is 398.12 m a.s.l., there is still a rather significant, around 2 m difference. As based on general practices and also the historical images of the town wall it seems unlikely that the wall here would have been as little as half of the size of the wall at Schnetztor, it is rather likely that the maximum water level of the August 1343 flood, at Konstanz, was (notably) over the peak flood level of the greatest known Lake Constance flood, occurred in 1817.

Moreover, this section of the lake, together with the river (Seerhein), likely has somewhat higher maximum water levels than along other wall sections of the lake, as the Dominican Friary and the wall section in question were located close to the place where the river – carrying the waters of the Obersee with naturally higher water levels, especially during floods, than the Untersee – entered the Untersee and thus here both the flux and the water level could be still higher (and was also chance for high waves). Additionally, based on historical images, the island of the Dominican Friary could be smaller and therefore the town walls more exposed in the 14th century, and – as the walls of the Dominican Friary – together with the town wall on the other side could also create a natural channelling effect, with increased flow velocity. Furthermore, if (like in the 15th century) mills operated on the Seerhein at Konstanz,[153] this could also mean a notable water-level increasing effect, particularly during floods.

Recent archaeological investigations in Konstanz revealed that, after the mid-13th century, parts of the lake shoreline likely silted up (as a probable cause, the authors suggest prolonged drought), and during or after that the 12th-century shoreline was then remarked with a row of posts. Sometime after 1300, Lake Constance experienced more frequent (and/or higher magnitude) flooding, it was necessary to raise the terrain in the shoreline area by approximately 1 meter, and the shoreline area was also reinforced by a quay wall and steps[154] Although this did not affect the absolute height of the town wall (and therefore the maximum flood level estimation), in some places it could influence the height of the wall that remained over the ground level.

The Alpine Rhine (Alpenrhein) and other, smaller alpine waterflows carry large amounts of sediment from the Alps into Lake Constance, particularly in the south-eastern part of the lake, periods with higher flood frequency and magnitude and increased human intervention in the Middle Ages could also notably influence the volume of sedimentation. Lake Constance sedimentation rates, and the change of lake basin elevation since the mid-14th century could also influence the comparison of 14th century and modern maximum flood levels. In the Obersee, east to Konstanz, the sedimentary core depth difference was ca. 20 cm between 1890 and 1990, while closer to Lindau it was ca. 46 cm (i.e. over 50% more) between 1850 and 1990 – which gives a roughly 20-30 cm sedimentary layer per a 100 year in modern times,[155] but even in the deeper parts of the (Obersee) lake basin (regarding 20th-century sedimentation) considerable differences (lower or higher sedimentary rate values) were as well identified.[156] Roughly similar values were The Holocene sedimentation rate in the Obersee was altogether

relatively slow, estimated to ca. 1 m in a thousand year; however, post-glacial sedimentary core analysis suggests that sometimes even a short (e.g. extreme flood) event, lasting only days or weeks, could result in a one-meter sedimentary layer in the affected slopes of the lake basin.[157] The decrease of lake depth due to sedimentation, however, does not necessarily mean the increase of water level; it rather means that the same amount of water in the 14th century potentially produced somewhat lower flood levels than in the 20th–21st century.

The Franciscan friar, living in the Lindau Franciscan Friary, Johann von Winterthur also mentions another, "never-seen" flood event of the Bodensee under 25 August (JC; see Supplementary Data 1). With giving the exact day (around St. Bartholomew) he only mentions that, due to the continuous and excessive rains, Lake Constance and the rivers around swelled very much, and the destruction and the desolation of people were extremely great ("*Item eodem anno in kalendis Septembris circiter festum sancti Bartholomei propter pluviarum continuitatem et immoderanciam lacus Potannicus et fluvii circumque tantum excreverunt, limites et terminos suos debitos et conswetos excedendo, quod nocumenta, pericula et discrimina immediate iam et paulo ante in superioribus enarrata cum hominum desolacione sunt permaxima.*").[56] This description in itself does not provide any details about the possible maximum flood level of the lake during this flood, but supports the description of the Konstanz chronicles about the timing and extraordinary magnitude of this (second) flood. It is a very important detail in his text that he mentions it as a continuation of the (rain, flood) events before, which means that – although Winterthur clearly mentions this flood as a separate event from the July flood – he indirectly supports the idea that late July flood of the lake to some extent continued for weeks, maybe even until the new, even higher, late August flood.

In connection with the first, late July flood of Lake Constance, Winterthur provides a more detailed description in another part of his chronicle (see Event 13): In that passage, he describes the flood as a process: first he presents the start (and causes) of the "never-seen" flood, and then continues with the disturbed everyday life and activities of the friars during the flood that seemed to last for a longer period of time, and with an increasing water level. However, as he provides no any further dates (only the above description, in another part of the chronicle), it is not clear whether he still talks about the first flood, or the later increase of the flood level on the island already corresponded to the second, late August flood wave. In the discussion of Event 13, we analyse this text and provide more maximum water-level information that could partly imply to this case (for more details, see Event 13).

2 Infrastructural changes and flood prevention measures after the 1342-1343 floods

2.1 Overview of flood damages and prevention measures

Table 2.1 Flood damage groups and management response examples in 1342-1343 and after (see also Extended Data Tables 3, 4). Damage types with examples of source text quotations, and flood prevention response (numbers: Supplementary Information 2.2; for further cases and references: Supplementary Data 1a, 3)

**Examples for flood destruction:** SI 2.2 No.-s	**Examples for flood prevention response groups (Extended Data Table 2)**
Dyke breaches or destruction: 29, 31, 55, 56<br>Lands flooded, agricultural damages: 3, 6, 12, 16, 20, 40, 41, 52<br>Loss/depopulation of settlements: 3, 4, 25, 28, 37, 46	Ia) New or repaired dykes: Dyke repairs and/or new dykes in e.g. Flanders, the Rhine delta and Frisia where sea surges damaged or destroyed dykes (and islands) in 1342/1343; canal (incl. sluice) reparations: e.g., Bologna 1343.[161]<br>Ib) River regulation for flood prevention: For example, in 1343-1345, the first known major Danube regulation at Oberaltaich, Bavaria (diverting the river into a new riverbed; building flood protection dikes – after the 1342-1343 Danube floods): 56.<br>Ic) New flood protection measures, agreements: For example, important new flood protection dyke agreement on maintenance regulations along the Nogat, primarily against ice jam floods (major branch of the Vistula; today N-Poland) where dyke ownership and maintenance responsibilities were fixed between landowners (1343).[162]
Bridge damages: 1, 2, 45, 7-9, 13, 16-19, 21-27, 32, 33, 38, 41-43, 45, 46, 48	IIa) Rebuilding bridges, more bridges built in following years: Many bridges were quickly rebuilt or temporarily replaced by wooden bridges. Many new bridges (or stone replaced wood) in the Holy Roman Empire in the 1340s-1350s.[8,163]<br>IIb) more, massive bridge(s) structure, technological innovations: Example: after the 12th-centruy Judith Bridge was destroyed in 1342, new Charles Bridge (built from late 1350s; temporary wooden bridge was used for crossing in between) much higher, broader, with improved technical solutions (e.g., stronger protection, pillar shape and design against ice, broader arches increase discharge capacity: see also Supplementary Information 1.1.1).[187164] Stone bridge in Dresden: presumed to be quickly rebuilt; technological innovations such as the large blocks of square stones joined at the bottom by pillars with iron clamps and lead were suggested to be initiated after the 1342-1343 floods.[165]<br>(Partly) successful bridge protection attempts during the 1343 flood in Zürich: 46.<br>In general, technological improvements of previous decades could be built into the new constructions, and also other floods of the mid-/late 14th century could influence technological innovations. Flood prevention improvements indirectly can also be deduced from later evidence.[8]
Destruction of urban buildings: 4, 16-18, 21, 23, 25, 40, 44-46, 49, 50, 52<br>Town walls (overflown, damaged): 17, 21-23, 25, 27, 33-35, 44, 47, 51<br>Riverine industry, infrastructure, roads: 4-6, 17, 21, 22, 48	IIIa) Reorganisation of urban space: Systematic reorganisation of town and riverine area, (multifunctional) buildings, also serving the purpose of flood prevention: For example, in Säckingen charter and local history evidence suggest the restructuring of the flood-damaged (down)town (on an island), and especially the built-up area along the shoreline, with inserting more stone walls in the town, and shoreline towers (e.g. Gallus) with flood-prevention function (early warning, diverting the main stream of the floodwater).[138,166] In Prague, stone buildings were propagated in the Old Town, and in the New Town (built in the 1350s along the river) only stone buildings were allowed (fire and possibly flood prevention purposes).[164,167,168]<br>IIIb) Reparation of (more and stronger) town walls (at least partly serving flood prevention purposes): For example, in Strasbourg, the new town wall section was built after the 1343 flood: 47 (also Supplementary Information 1.1.3). In Säckingen, new town wall system was built (incl. the Gallus Tower) against the floods of the Rhine, in and after 1343[138] (see also Supplementary Information 1.3.2).<br>IIIc) Shift of importance in public space to flood-free mainland location: For example, in Säckingen from 1343 the settlement in the (higher situated) dryland (Obersäckingen) received extensive right, suggesting an increase in its importance after the floods.[138,166]
Psychological impact (fear, distress): 40, 45, 53, 57<br>"Whole world" hit: 1, 16<br>Dearth, illness: 3, 45<br>Mortality/victims: 3. 4, 12, 16, 36, 37, 48, 54	IVa) raising (scientific, high-status) awareness: For example, as an explanation of too extensive flooding and extreme weather, Paris (Sorbonne) astronomers suggested a 500-year return period astronomic constellation that gained significant scientific attention: 58. Raising awareness of the pope for health issues, in Kalocsa (Hungary); [16] raising awareness of the great dearth and illness following the rains and floods, for example, in Allgäu and Bavaria (Johann von Winterthur).[56]<br>Vb) floodmark, (religious, urban) procession, public memory: For example, the flood mark on the Weißfrauenkirche in Frankfurt, the memorial stone and mark on the Blasius church wall in Hann. Münden (see Supplementary Information 1.2). In Frankfurt, an annual Magdalena procession took place in the later centuries, visiting the areas of the town hit by the great flood. With this, raised awareness of the possible maximum height and the maximum extension of water, keeping its memory in the urban space for centuries[8,12,169] (see also Supplementary Information 1.2). Religious response: thanksgiving offerings e.g., Göttingen Cross: 57, religious prayers, processions: 40.<br>Preventing health issues caused by bad food: Würzburg: food regulations (e.g., prohibiting to sell the meat of drown animals, stepping up against food (e.g., bread) quality decrease and forgery.[8]

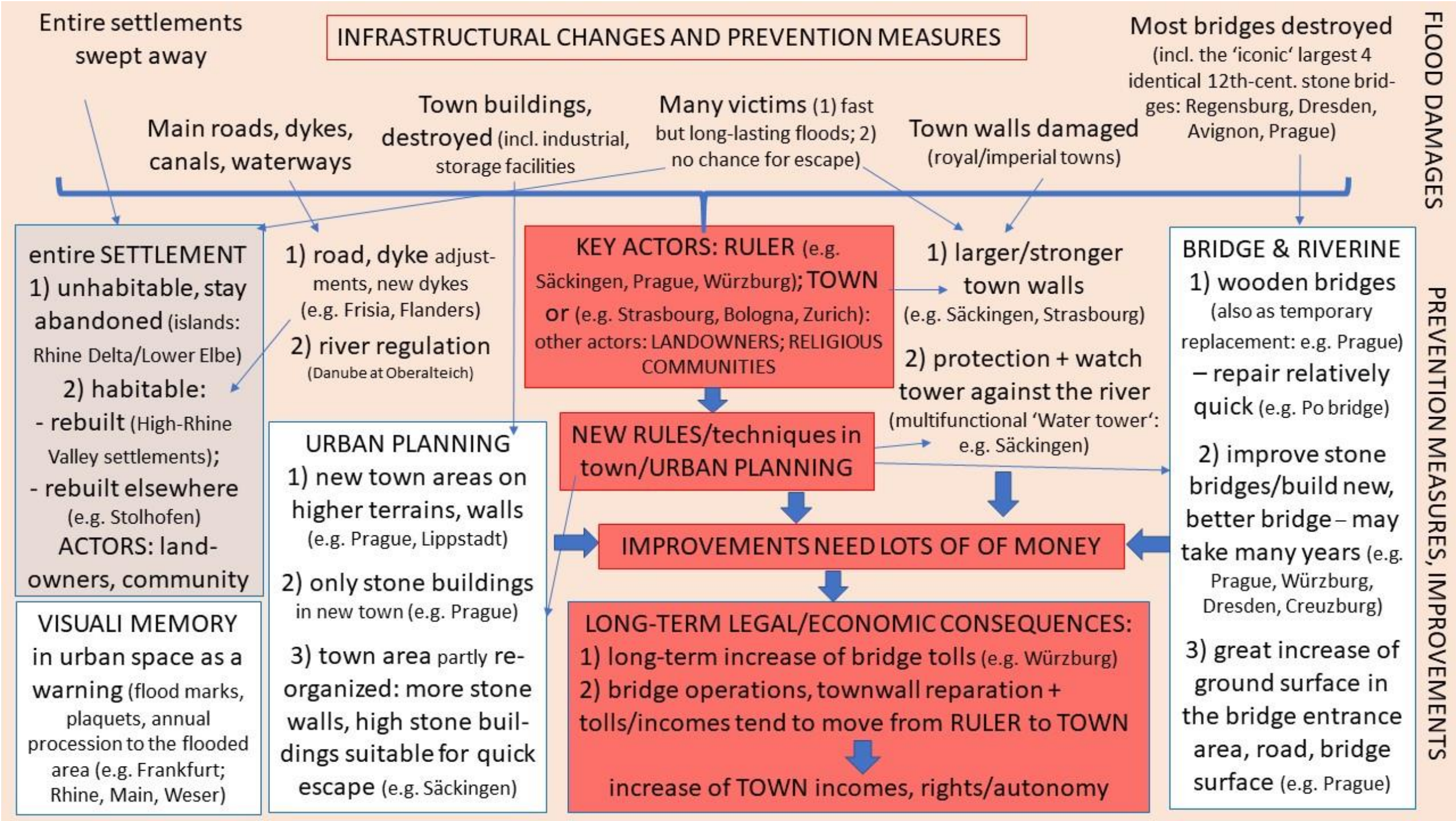

Fig. 2.1 An overview of examples for flood management response in written contemporary (Supplementary Data 3) and medieval historical and archaeological data[8,163,164,169,170] (Supplementary Information 1)

2.2 Flood damages in built-up area and riverine infrastructure: Source texts
(for further text references, see: Supplementary Data 3; hereafter SD3):

1342

Floods in the 'whole world' / 'many other places' (i.e., in large part of Europe – viewing it from Prague or Dresden or Münster): "

1) Franciscus Pragensis p. 569[58] (Feb. 1342; SD3: line 8): outstanding floods in the 'whole world'; destroyed both the stone and wooden bridges): "*In toto quoque orbe terrarum tunc temporis fuerunt maximae aquarum inundationes, ita quod et pontes lapidei et lignei per aquas nimias sunt destructi.*"

2) Annales Veterocellenses p. 43 (Feb.; SD3: line 13): "*1342. Infra octavam purificationis corruerunt pontes in Dresden, in Praga et aliis multis locis*."

3) Chronica episcoporum Monasteriensium p. 48 (famine, illness, mass mortality of people and animals, many towns, lands, castles and villages/settlements submerged; SD3: line 63): "*…maxima subitanea mortalitas in fluxu sanguinis est subsecuta et inaudita validissima fames in universa terra, ita quod homines diversis herbis sicut bestie vescebantur. Et in hac fame homines et iumenta communiter perierunt. Et plurime civitates terre, castra et ville de inundancia aquarum sunt submerse.*"

Prague and Vltava Valley (Feb. 1342)

4) Franciscus Pragensis p. 568-570[58] (Judith Bridge /one of the greatest and most famous stone bridges of contemporary Europe/ damaged several places – only ca. 1/3 remained, transportation along and across the Vltava/Moldava blocked, Prague riverine districts, buildings, wood/trees, mills, entire villages along the Vltava destroyed, waterways blocked, huge number of victims, loss of animals etc. in Feb.; SD3: line 8): "…*ruptus est pons Pragensis in pluribus locis, quod vix tertia pars de ipso remansit, verumtamen aquarum et glaciali impetu concussa et debilitata. Et omnia molendina et obstacula sunt destructa, pluresque villae circa litora sitae cum hominibus et ceteris animalibus sunt absorptae et suffocatae. … Et in civitate Pragensi aquae longe lateque dampna plurima intulerunt. Cives quoque strues magnas et multas lignorum ad aedificia varia dispositorum impetu aquarum perdiderunt; et quia inopinate et repente factum est diluvium, visae sunt domus cum hominibus, molendina cum suppellectilibus, infantes in cunabulis natantes, quibus matres anxiae succurrere non valuerunt. Visa sunt quoque animalia diversa domestica et varia utensilia deferri. Porro illa quassatione miserabili mitigate multa corpora hominum et iumentorum submersorum sunt reperta. Et primo homines intellexerunt, quod tam grande et arduum bonum perdiderunt, nam cum res necessaria perditur, primo eius pretium advertitur. Nam quasi corona regni occidit, cum ille pons famosus corruit et fit labor magnus et periculum in navigando, moerorque pauperum naulo carendo. Pons quoque fortissimus a venerabili patre domino Johanne quarto, Pragensi episcopo vicesimo septimo in Ruduicz efficaciter, firmiter, decenterque constructus inviolatus permansit, licet maior ibi concursus exstitit aquarum et grandior de glacie massarum impulsus. Tunc temporis circa minorem civitatem Pragensem propter multitudinem arenae, quae impetu aquarum exstitit aggregata, fuit obstructus aquae meatus et amplius molendina ibidem haberi non potuerunt. / Surgit rea mira, grandis validaque ruina / Pontis Pragensis per rivum Multaviensis, / Insultus cuius mala terrae parturit huius, / Planctus prorupit, ingens dolor intima rupit, / Villae nam multae perierunt optime coltae, / Domus natabant, homines in eis ululabant, / Et glacies gentes nudas dixut rugientes.*"

5) Beneš Minorita p. 492 (3/4 of Judith Bridge destroyed, all mills, weirs, many settlements with people and animals, great obstruction of navigability; SD3: line 8): "… *multitudo hominum in pluribus terris fuit extincta, factum est grande diluvium per impetum aque nivalis et pluvialis, et propter ingentem molem glaciei et spissitudinem ruptus est pons lapideus Pragensis in pluribus locis, et ita, ut vix quarta pars remaneret. Omnia molendina eorum et obstacula destruct sunt, et plures villa in littore cum hominibus et animalibus perierunt. Et quia inopinate et repente factum est diluvium, vise sunt domus cum hominibus molendina cum suppellectilibus, infantes cum cunabulis natantes, quibus nullus succurrere poterat. Et facta sunt dampna irrecuperabilia ex inundacione huiusmodi, et precipue propter rupturam pontis Pragensis. Tunc enim primo homines intellexerunt, quod tam grande et arduum bonum perdiderunt. Fit quoque labor magnus et periculum in navigando, et meror pauperum naulo carendo.*"

Deta (Hungary; Feb. 1342)

6) Hungarian National Archives DL 75835 (perambulation charter dated to 13.02.1342: perambulation/land survey could not be carried out because of great flood; SD3: line 11): "…. primo accessissent ad faciem Possessionis Déta praenotata et eandem praesentibus aliquibus Nobilibus Comprovincialibus, qui per ipsos inibi convocari potuissent, per suas veras metas, et antiquas reambulassent, reambulatamque quia ipsi ob nimiam aquarum inundationem dictam Possessionem Déta regali mensura mensurare nequivissent, ides ipsi duntaxat visu considerando eandem totalem Possessionem Déta ad quatuor aratra juxta Consvetudinem Regni sufficere debere perspexissent, ac considerassent, ipseque Consideratione peracta, rectam dimidietatem ejusdem cum suis Utilitatibus quibuslibet, et pertinentiis, eidem Johanni, filio Petri actori, sine praejudicio Juris alieni, prout sibi pertinere dinosceretur, nullo Contradictore apparente, statuissent perpetuo possidendam, …."

Dresden (Germany; Feb. 1342)

7) Chronica Brevis … Missne et Thuringie Sp. 55 (the bridge in Dresden destroyed; SD3: line 13): "*Item 42. inundacio aquarum pontem in Dresden destruxit.*"; Chronicon parvum vernaculum Sp. 350: „*In dem XLII. Jare vyl dye brucke eyn in Dreseden.*"

8) Beneš Minorita p. 492 (Feb.: stone bridge of Dresden damaged; SD3: line 8): "*Corruit autem ipse pons ipsi anno in die sancti Blasii in ipsa nocte, et pons lapideus in Drzezden super eodem flumine corruit.*"

9) Annales Veterocellenses p. 43 (SD3: line 8): "*1342. Infra octavam purificationis corruerunt pontes in Dresden, in Praga ….*"

Venice and other towns along North-Adriatic sea shore; Italy (Feb. 1342)

10) Franciscus Pragensis p. 569[58] (Aqua Alta in Venice and other towns in the broader area of the Venetian lagoon, all water tanks/reservoirs destroyed/flooded; SD3: line 17): "*Mare quoque fuit in altum elevatum, quod omnes cisternas Venetiis et in aliis civitatibus mari adiacentibus totaliter suffocavit et plurima dampna causavit*."

11) Johann von Winterthur p. 189 (buildings in the 'entire town' flooded, great damages; SD3: line 17):[56] "*Tanta eciam inundancia eodem tempore maris circa Venecias facta fuit, quod totam civitatem absorbere quodamodo visum fuit; nam eam longe lateque occupans et in singulis domibus alte ascendens in reidicbus et speciebus aromaticis et aliis rebus dampna inestimabilia civibus intulit.*"

Danube Valley (Feb. 1342)

12) Johann von Winterthur p. 189 (extensive flood with thousands of victims; ultimate destruction of crops; SD3: line 76):[56] "*… Danubius fluvius … in tantum excrevit, quod in longitudine duorum miliarium et in latitudine unius miliaris per sui alvei effluenciam nimiam in quibusdam partibus res cunctas et fructus universos destruxit, ut fertur, et quod de hominibus VI milia absorbuit.*"

Rouen (Seine; Feb.-March 1342)

13) Pierre Cochon p. 331 (the great stone bridge of Rouen largely destroyed; many victims, great destruction of goods, extensively flooded the cathedral, with damages; SD3: line 12): "*Anno Domini millesimo trecentesimo secundo, magna inundatio aquarum fuit super terram, et incepit octavo idus februarii, duravit que usque ad sexto Kl. marcii, quarum inundatione et habundacione quatuor arche pontis Rothomagi penitus repleverunt, et multe gentes, tam magne quam parve, in maximum dammum cum multis aliis bonis perierunt, certissime credentes quod predicte aque subito per dimidium pedem et amplius in monasterio, in clausiro, in refectorio istius prioratus Beate Marie de Parco, ordinis Grandimontensis (Notre-Dame-du-Parc, près de Rouen), apparuerunt taliter quod, in predictis locis et per alia loca inferiora predicti prioratus, oportebat intus exterius intrare et exire periculose cum planchis maximis et batellis; et per portam vocatam archiepiscopi batellus ponderis sex tonneliorum intrabat pariter et exibat….*"

14) Annales S.Catharinae de Monte Rotomagensis p. 32 (Seine flood overflow town wall; SD3: line 12): "*…tantum inundavit fluvius Sequanae circa Rotomagum, quod aqua superavit calceyam de Martainville, et quod gentes desuper navigio transfretabant usque prope portam de ponte Hunfredi.*"

Mainz (Main, Rhine; July 1342)

15) Johann Kungstein p. 83 (SD3: line 32): "*Post m c triplum fit xl et ii duplicatum / Diluvium vehemens dampna multa ferens Praxedis festo, cuius facti memo resto.*"

River Main, Rhine: Cologne, Mainz, Frankfurt – and many provinces of the "world" (July 1342)

16) Cronica de episcopis Maguntinis p. 144 (15th century source; bridges damaged; SD3: line 32) "…*per plurimas provincias et maxime circa Renum et Mogonum fluvios ac alibi omniaque in frugibus, fructibus, feno, edificiis, pecoribus et heu pluribus hominibus multiformiter et miserabiliter devastavit. Insuper maxima pars civitatum, precipue Colonia, Maguncia, Francfurt apud domum Theutonicorum cum parte pontis diruit penitus et evertit, preterea plurima horribilia et dampnosa evenerunt in variis mundi provinciis.*"

Erfurt (and 'all lands'), Reinsburg, Würzburg (Gera, Main; July 1342)

17) Chronici Saxonici Continuatio Erfordensis p. 483 (the Main went over the townwalls, taken away bridges (incl. Erfurt stone bridge), houses, great towers; SD3: lines 39-41): "*Des selbin jares umme sente Marien Magdalenen tag da was vil groz gewißere in allen landen unde ouch zů Erforte, also daz nimant mochte mit eime roße in den gaßen gerite zu Erforte. Daz selbe vurte hinweg die brucken zů Reinsborg unde zů Wirzebůrg mit eime grozen turme, unde. unde der Moyn ging ubir die můren, unde man vůr mit schiffen ubir die dach, unde tet vil anderen großen schaden.*"

Ertfurt (Gera; July 1342)

18) Cronicae S. Petri Erford p. 385, 388 (SD3: line 39): "*Anno milleno tria C quadraginta secundo / Torrens excrevit, pontes domosque delevit. … Eodem anno fuit tanta inundacio aquarum in Erfordia in die Praxedis virginis, ut nemo in runcino in plateis equitare posset, et destruxit longas temetas Erfordie nec non pontem apud Sanctum Nicolaum*."

Erfurt, Würzburg, Bamberg, Frankfurt, Dresden,Wetzendorf, Regensburg (Gera; Main, Danube, Elbe, Unstrut; July 1342)

19) Cronicae S. Petri Erford p. 375 (bridges in all the listed towns damaged – in Dresden again?; SD3: line 39): "…*Fuit tanta inundacio aquarum in Erphordia in die Braxedis virginis, ut nemo in runcino equitare posset, et destruxit longas temetes Erphordie, pontem ante valvam Sancti Augustini, Sancti Iohannis ante Krampfentore, pontem in Herbipoli cum magna turri, pontem in Ratispona in Dreseden, in Frankenfordia, in Wezindorf, in Babinberg et multa alia dampna peregit*."

Ensdorf (and 'all lands', July 1342), Bavaria

20) Annales Ensdorfenses p. 7 (SD3: line 58): "*1342. Hoc anno tanta inundacio pluvie facta est, ita ut omnes fructus terre devastaret in vigilia Braxedis.*"

Thüringia and other areas; Kreuzburg, Isenach, Erfurt, Vacha (Sale, Werra; July 1342)

21) Düringische Chronik p. 574 (first half of 15th-century source; bridges, mills, weirs and others – e.g. the stone bridge in Vacha, all bridges is Eisenach, both the long bridge and the stone bridge in Erfurt – destroyed, and the water overflown the town wall in Kreuzburg, and the monastery ruined; SD3: lines 42-43): "*Zu den gezeiten alsso man schreib noch Cristus gebort 1342 jar aud sente Marien Magdalenen (in die Praxedis virginis) tagk do wart alsso gross wasser von regen in Doryngen unde ouch ynd andirn landen (das die Sael unde ouch die Werra enwegk furten husser unde hussrat, brucken unde stege boyme unde zune unde was dor bey gelegen was unde ted grossen schadin an den moeln unde an den weerin. Zu Vache zu brach is die steynen brucken. zu Krutzburgk gyngk is obir die statmuwirn, unde die clostirfrawen musten mit alle yrem gesynde das clostir rumen. zu Isenache do bleib keyn stekg*

noch brucke) zu Erfforte kunde nymant yn den gassen gereiten noch gewandirn. Die lange brucke unde die steynen bruck bey sente Nicolause zu brach is. …"

Erfurt, Kreuzburg; Werra, Gera, Sala, Rhein, Mosel, Danube, Elbe, Werra, Weser, other rivers (July 1342)

22) Chronicon Ecclesiasticum Nicolai de Siegen p. 385-386 (many bridges and houses along the Werra, Gera, Sale; SD3: lines 42-43): "… *nam Werra, Gera, Sala plures pontes edificia domus sepes molendina deportarunt. Plura molendina confracta sunt, Erfordie pene omnes pontes destructi; in Cruzeburg Werra ultra muros civitatem intravit, et hec aquarum habundancia pene ubique terrarum fuit, in Reno, Mosella, Danubio, Albea, Werra, Wisera, etc*."

'entire Teutonia'; Frankfurt, Sachsenhausen (Main; July 1342)

23) Frankfurter Chroniken p. 1, 2 (the great bridge – with buildings and towers to Sachsenhausen destroyed, villages in the area destroyed; SD3: line 46): 1: "…*quasi per omnes partes Theutonie, et longe major quam alia inundatio fuerit: ita quod Mogus predictus in ecclesiam sancti Bartholomei prefatam fluebat per portam rubeam que vulgariter dicitur die rode důre, ac pons dicti opidi cum multis pilaribus suis et turris magna fortis et sublimis ejusdem pontis versus Sasinhůsen cum capella lapidea desuper ibidem sita, pro tunc feria quarta (24 July) sequenti propter aquarum predictarum magnitudinem ceciderunt et funditus destruebantur. et quasi per omnes vicos ipsius opidi Frankenfordensis Mogus fluxit et transivit, ita quod ex eo circumcirca ipsum opidum dicta aqua in fussato ad magnam profunditatem similiter ibidem profluebat*."

24) Deutsche Annalen von 1306-1343, p. 5 (bridge to Sachsenhausen destroyed; SD3: line 46): "*Hernach an sanct Jacobs abend umb 1 Uhr hatt das waßer die brück und den thurn gegen Sachsenhaußen, de pfeiler mit der hübschen capellen in grund hinweggeführt, ausgenommen 6 schwibbögen gegen Frankfurt zu,…*"

Würzburg, (entire) Main Valley (July 1342)

25) Michaelis de Leone Canonici Herbipolensis p. 469 (Würzburg walls and towers, many stone houses and church destroyed; all stone and wooden bridges in Würzburg, and also upstream and downstream were destroyed; SD3: line 41): "… *pons Herbipolensis lapideus et pretiosus cum turribus et muris suis et ipsius civitatis, ac etiam multis domibus lapideis ibidem circumquaque, subito corruerunt. Et huiusmodi Mogi aquarum inundacio super gradibus porticus ecclesie Herbipolensis prope primas statuas lapideas excessive affluxit. Omnes quoque pontes lignei et lapidei ipsius Mogi supra et infra corruerunt confracti. Et tunc etiam immense dampnositates perdiorum urbanorum et rusticorum iuxta Mogum per totum lamentabiliter evenerunt*."

Bamberg (Main; July 1342)

26) Versus Babenbergenses p. 639 (bridge destroyed; SD3: line 47): "*Multos submersit, turrim cum ponte depressit*."

Minden (Weser; July 1342)

27) Chronicon Henrici de Hervordia/Hermann von Lerbeck p. 72 (the main church, town gate, old bridge destroyed; SD3: line 45): "*Item aquae Wiserae tantum excreverunt, quod civitatem Mindam intrantes per maiorem ecclesiam ad forum usque pervenerunt et ostium portae civitatis quatuor vel quinque pedibus minus ad superius totum repleverunt et super illum altum pontem, quem funditus destruxit, elevatae sunt a. D. 1342*."

Rhine, Main Valleys (July 1342)

28) Gesta Baldewini de Luczenburch p. 256 (entire settlements diminished along the Rhine and Main; SD3: line 58): "*Postea in vigilia Jacobi maxima erat aquarum per totum Rhanum, non a Rheno sed a fluvio Mogos dicto, quod tamen contra suam est naturam, in flagellum causata; ita quod integrae villae disruptae et defluxae cernebantur*."

Rhine Delta (summer 1342)

29) Annales Tielenses p. 26 (dykes damaged, large islands submerged; SD3: line 61): "…*in vigilia noctisque diei beati Iacobi apostoli tanta fuit inundacio aquarum, ut per confractionem aggerum utraque Batua tam superior quam inferior et eciam insula Tylensis usque Diefdyk preter Tyell, Drompt submersa est*."

Limburg (Lahn; July 1342)

30) Tilemann Ehlen von Wolfhagen: Limburger Chronik p. 28 (erroneously under the year 1344; SD3: line 60): "*9. Item in der selben zit unde jare uf sente Jacobes dag des heiligen apostolen gelegen in dem erne da was große flut unde waßer uf erden, daz großer unsegelicher jamer unde schaide geschach von der flut. Unde hatte nit sere geregent oder waßer gefallen zu der zit, also daz ez von wunderlicher godesgewalt was unde quam, daz di Schoppen, daz man mit nachen allenthalben darober fur. Unde ist di erste waßerflut di den alden luden indenklich ist.*"

Esslingen (Neckar; July 1342)

31) Johann von Winterthur p. 191 (great landslide in Esslingen; SD3: line 49):[56] "*Multe quoque civitates et eorum cenobia, sicut patuit in Eslingen, propter aquarum inpetum et effluenciam importunam in magna parte dirupta sunt cum maxima hominum displicencia, desolucione et tremore*."

Frankfurt (Main; July 1342)

32) Johann von Winterthur p. 191 (many bridges, e.g., the one in Frankfurt, destroyed; SD3: line 33):[56] "*Preterea eodem anno in estate in partibus Alemanie tanta fuit facta inundancia ymbrium et excrescencia aquarum, quod multi pontes firmissimi, quorum unus in Frankenfurt fuit, discerpti et dissipati sunt*."

Cologne (Rhine; July 1342)

33) Prima Vita Clementis VI. p. 242 (bridges and towers destroyed, ships could pass over the town wall; SD3: line 33): "…*pontesque et turres diruerent, et quod est mirum dictu, in civitate Coloniensi navibus ad muros scanderetur,...*"

Avignon (Rhône, Durance; early? summer 1342)

34) Prima Vita Clementis VI. p. 242 (Avignon town flooded; SD3: line 68): "…*et intra civitatem Avinionensem navigio transiretur*."

35) Achard, Archiv. Avignon (SD3: line 68): "*Forte inondation occasionnée par de grandes pluies qui réunirent dans les rues d'Avignon la Durance et la Rhône*."

Magdeburg (Elbe) – also in general sense (1342)

36) Gesta archiepiscoporum Magdeburgensium p. 435 (in general, people barely escaped in the uppest parts of houses; but many people and animals died, as they could not – had no time – run to the hills; SD3: line 38): "…*diluvium aquarum tam magnum, quod in plerisque locis*

homines in superioribus domorum solariis se vix poterant conservare; unde homines et iumenta plurima perierunt, que non poterant confugere ad montes."

Settlements in Frisia, Eiderstedt, Ditmarschen regions (Eider, Elbe; 1342)

37) Chronologia Rerum Danicarum p. 305 (all agricultural lands devastated/depopulated/ because of the flood; many people died; SD3: line 90): "*In Frisia, Eydorensi et vicina Dithmarsia, Eydora et Albis facta inundatione omnes agros depopulantur, et haud exiguum hominumqve numerum opprimunt*."

Straubing (Danube; 1342)

38) Annales Straubingenses p. 694 (the "deluge" over flown the bridge; SD3: line 95): "1342. *Diluvium erat tantum, quod transiit ultra pontem in Strubinga*." Same information: Annales Windbergenses p. 566; Compilatio Chronologica p. 342.

Padua, entire Lombardia (Po and tributaries; Nov. 1342)

39) Johann von Winterthur p. 195 (major disruption of travel routes, foreign pilgrims and merchants with goods obstructed to travel; SD3: line 74):[56] "…, *aput civitatem Paduanam et aliis partibus Longobardie …, quod per multos dies viatoribus vie obsite et obstructe facte fuerunt et ob hoc mercatores peregrini plures illic contra voluntatem suam cum gravibus sumptibus suis detenti fuerunt, non valentes cum mercibus suis ad terras nativas repedare*."

1343

Cawood (England; August 1343)

40) Letter of the Archbishop of York to the official of the Court of York (incessant rains and inclement weather destroy harvests, cause sterility – because of the sins/ungratefulness of people; prayers and processions ordered for peaceful weather; SD3: line 140): "….*Quas et quantas aeris intemperies imbriumque stillatio prodiga incommoditates ingerant his diebus, fructus perdentes, ad usus producti humanos, intuentibus non absque (stupore ostendunt, dum fructuum ipsorum sperata fertilitas ad inopinatam sterilitatem, proh dolor! tali turbine redigetur, nisi benignius plebi Suae de excelsis prospiciat sancta dementia Creatoris: sed hoc quippe efficiunt peccata hominum, qui arridentes prosperis, tanquam ingrati, fiunt immemores Largitoris. Restat igitur ad Ipsum solum recurrere, Cujus misericordia excedit judicium, et Qui de conversione gaudet piissime peccatorum, orationibus insistendo humiliter et precibus, ut Ipse, mitis et mansuetus Dominus Deus, iram Suam avertat a populo, Suo praetioso sanguine quem redemit. Discretioni itaque vestrae injungimus et mandamus quatenus faciatis, quam citius poteritis, publicari, quod, tam in nostro cathedrali, quam in aliis collegiatis et conventualibus, ac singulis parochialibus ecclesiis nostrae dioecesios, omni quarta et sexta feria, devote processions fiant cum decantatione letaniae solemni, et specialis oratio dicatur in missis pro serenitate aeris omni die similiter, et pro domino nostro rege et statu regni totiusque populi Anglicani; ut frequentem pulsationem Salvator exaudiens creaturae subveniat, quam ad Suam creavit imaginem ipse Deus. Haec in singulis archidiaconatibus dictae nostrae dioecesios per locorum archidiaconos, eorumque officiales, faciatis celeri executioni mandari. …* "

High-Rhine from Rheinau to Brisack (July/August 1343)

41) Heinrich Diessenhofen p. 38-39[59] (all bridges destroyed between Rheinau and Brisack; SD3: lines 128-129): "… *festum sancti Iacobi factum fuit tantum diluvium, quod omnes pontes*

Reni ab oppido Rinaugensi exclusive dyoc. Const. usque ad oppidum Brisaugiense eiusdem dyocesis destructe sunt, et multa prata agri et vinee totaliter eversa et devasta sunt."

High-/Upper Rhine Schaffhausen to Strasbourg (July/August 1343)

42) Johann von Winterthur p. 205 (great damages; SD3: line 130):[56] "*Praeterea eodem anno citra festum sancti Jacobi propter inundaciones aquarum exortas et causatas propter ymbrium supra proxime memoratorum excessum a Schafhausa usque ad civitatem Argentinensem omnes pontes Reni fluvii propter ipsius inswetam magnitudinem et inpetum furiosum dirupti et abstracti sunt cum hominum non parva desolacione*."

Laufenburg (July/August 1343)

43) Johann von Winterthur p. 215 (12 houses and the bridge taken by the flood; SD3: line 131):[56] "*Nam Renus ultra modum incrementa suscipiens de Lo(e)fenburg oppido fere XII domos cum pontis abstractione abduxit*."

Säckingen (July/August 1343)

44) Johann von Winterthur p. 215 (flood exceeded the town walls of Säckingen, destroyed houses, took people, and forced the citizens to seek refuge; SD3: line 132):[56] "*Insuper ultra muros oppidi Sekkingen influens rapidissime habitatores universos fere eius exclusit et effugavit cum pluribus eciam domibus, que miserabiliter dissipando et a locis suis avellendo abduxit*."

Rheinfelden (July/August 1343)

45) Johann von Winterthur p. 215 (Rheinfelden bridge destroyed with its stone piles – reflecting on extreme magnitude/force of flood, many houses and other buildings destroyed, uprooted stone statues; it also hit the castle near the bridge; SD3: line 133):[56] "*Nichilominus pontem oppidi Rinvelden constructum ex pilis lapideis totaliter dirupit. Nam inpetuose plures domos et propungnacula et alia diversi generis edificia deducens et cum eis in statuaria lapidea et firmissima impingens ipsa funditus de suis locis evulsit. Habitatores quoque castri iuxta pontem in medio sui quasi siti seu fundati deprehensos famis miserrimis angustiis et submersionis metui et aliis calamitatibus variis et sevissimis implicavit*."

Basel (1343)

46) Die Grössern Basel Chroniken nach Schnitts Handschrift p. 251 (major general destruction of bridges, buildings and the villages along the Rhine; SD3: line 134): „*Anno 1343 ward der Rein also grosz uff sant Panthelions tag, das er alle brucken am Rein hinweg fu(e)rt; desz glichen thett er grossen schaden an den thurnen und do(e)rfferen*."

Strasbourg (July/August 1343)

47) Fritsche Closener p. 132[57] (July 1343: flood caused great damages in the townwalls; then the August flood was even greater; SD3: line 135): „*Dazselbe waßer det ouch vil schaden an den ringmuren zů Strosburg, …, vier tage vor sant Bartholomeus dage 3 der-noch, do kam aber ein also große waßer, alse mans ie gesehen hette zů Strosburg, one daz neheste dovor*. ... donoch maht man die ußer ringmure von Utengaße untz an den turn und den turn dermitte, also do vor geschriben stot, do man zalt 1346 jor."

Zürich (July/August 1343)

48) Eberhard Müller (in Ettmüller p. 70;[143] see also: Klingenberger p. 62; bridges damaged but largely saved; a major bridge swept away; SD3: line 136): „*ûf sant Jacobstag, dô wärent diu wasser ze Zürich alsô gros, das es über die bruggen gieng und über das Silfend, und muost man die bruggen beschweren mit trotböumen, mit stainen und mit zubern voll wasser. Des selben mauls ran hern Götfrid Müllers hûs ûf der nidern brugg ze Zürich in der nacht hin weg, und gestuond an der brugg in dem Hard bi dem durn; und dô man das hûs anfieng schlisen, dô brach die brugg und gieng das hûs und diu brugg mit ainander enweg. Es runnert ouch des selben mauls die müllinen ze Zürich alle enweg unz an zwô die behûob man mit nôt*."

Luzern (July/August 1343)

49) Johann von Winterthur p. 213 (river broke into the Franciscan monastery and reached never-seen height; SD3: line 137): [56] "*Item eodem anno, quod suo pretermisi loco non volens set nolens, quia nondum mihi tunc innotuerat, tempore estivali in una excrescencia aquarum excessiva propter ymbrium immensitatem fluvius Rusa oppidum Lucernense preterfluens tantum excrevit, quod in choro monasterii fratrum Minorum illic residencium altari maiori apropinquavit, quod a retroactis annis forte nunquam visum vel auditum fuit. Tanta eciam altitudine contra altare fluendo processit aqua, quod onuste navi ad portandum eam suffecisset*."

Winterthur (July/August 1343)

50) Johann von Winterthur p. 213 (river broke into the nunnery; SD3: line 138):[56] "*Parvus quoque fluvius innavigabilis aput oppidum Wintertur fluens To(e)sa nuncupatus tantum inundavit, quod unum cenobium monialium coherens sibi cum inpetu pertransiens iacturam maximam intulit, immo quasi per suum augmentum more torrentis rapidissimum cenobio et habitatoribus eius minabatur excidium, a tempore, quo non extat memoria, insperatum*."

Konstanz (July/August 1343)

51) Heinrich Diessenhofen p. 39 (town wall damaged/overflown; SD3: line 144):[59] "*Item tercia die post festum beati Bartholomei anni predicti lacus ascendit murum civitatis Constantiensis iuxta pontem piscium quo itur ad Predicatores, quod antea non est visum, ut antiquiores tunc temporis referebant*."

Lindau (July/August/September 1343)

52) Johann von Winterthur p. 203 (July 1343; SD3: line 139):[56] "….*extra oppidum Lindaugense vineas, agros et prata et fossata, intra vero domos et plateas et vicos occupavit. Anbitus quoque fratrum Minorum illic residencium et viridarium et officinas subintrans fedavit*."

53) Johann von Winterthur p. 206 (August 1343; SD3: line 139):[56] "…*nocumenta, pericula et discrimina immediate iam et paulo ante in superioribus enarrata cum hominum desolacione sunt permaxima*…."

54) Johann von Winterthur p. 207 (early/mid-September 1343; SD3: line 148):[56] "*Preterea eodem anno proxima die ante vigiliam nativitivatis beate virginis paulo ante crepusculum tanta fulgure et tonitrua usque ad medium noctis durancia cum pluviis excessivis et vehementibus, cum lapidibus quoque grandinis graves hominibus suo descensu lesiones inferentibus facta sunt, quanta a multis retroactis temporibus nulla hominum quamodo meminit antiquitas. Propter enim tanto tempore pluvias ex insperato supervenientes et breviter perdurantes fluvii repentinam et insolitam excrescenciam et augmentum stupendum circa lacum Potannicum, presertim circa Lindaudiam susceperunt*."

Later/general

Frisia (1342/1343)

55) dyke account books, Holland (with reference to dyke breach/breaches – and the cost of repair – in ca. 1342–early 1343 in Hamaker p. 318; SD3: line 169): "*Item van der Goyer veen, dat si nywes ghebroken heben, Claes Spodier, 6 lb 10sc*."

Danube at Oberalteich (1344)

56) Chronica de ducibus Bavariae (Danube regulation in 1344 because of dangerous riverbed after 1342-1343 floods; SD3: line 182): "*ad a. 1344: Anno Domini MCCCXLIIII abbas et conventus monasterii sancti Petri in Obernaltach ordinis sancti Benedicti Ratisponensis diocesis inceperunt magnum et arduum opus multis inredibile comparari. Magnus enim fluvius ille et nominatus Danubius, iuxta quem claustrum illorum fundatum est, per longa tempora fundo illorum sic appropinquavit, quod ab eo distans ad unius sagitte spacium crebra litorum sulcacione minabatur ipsum cenobium cum suis silvis, pratis, agris, pascuis funditus suvertendum. Unde ipsius Danubii fluvium ab antiquo suo decursu rectractum per novam, quam in magna a loco suo distancia vallem foderant, a suo cenobio abduxerunt*."

Göttingen Cross (July? 1342)

57) Inschriftenkatalog: Stadt Göttingen (No. 5: St. Albanikirche; dedicated to Iesus for saving the Goldsmith in the great flood of St. Margaret; SD3: line 44): "*i*(esus) *n*(azarenus) *r*(ex) *i*(udaeorum) / *an*(no) *m° ccc° xl ii do ver drank hermen goltsmet in der groten vlot to sentemargret*(en) *dage.*"

Paris, Sorbonne (about the year 1342)

58) Johann von Winterthur, p. 215 (astronomers of Paris investigated the cause of the extraordinary flooding in Europe, with applying astronomical tables they concluded that such a constellation occurred only 500 years before, and will not happen for another 500 years; SD3: line 158): „*Audientes autem astronomi Parisius constituti equoris inswetam diminuciones et in quibusdam regionibus insolitum aquarum excessum et causam horum rimari et indagare satagentes repererunt, ut fertur, forte in astronomie tabulis quandam stellam excellenter rutilanten aquas nimis haurientem ac elevantem et post refundentem regnare. Quam ante D annos rengnasse et usque ad illum annum ultra non et ab illo anno usque post iterum totidem annos ultra non regnaturum asserebant*."

3 High prices in Europe, food shortage, dearth and famine in 1342-1344

3.1 Subsistence crisis intensity & grain (beverages) prices in Europe, data sources and key price-related information in 1342-1343

Following the bad harvest and harvest losses, a significant dearth and starvation affected large part of Europe in 1342-1344, West-Central Europe in particular. Combined with political stress and war conditions, areas outside of Central Europe, less (or not) known to be hit by bad or lost harvests, were also affected in a varying extent (*Fig. 3.1.1*). A significant price rise is detectable in the Western and West-Central European grain prices series, presented in *Fig. 3.1.2* – in some areas price rise in 1342-1343 was greater than during and after the Black Death.

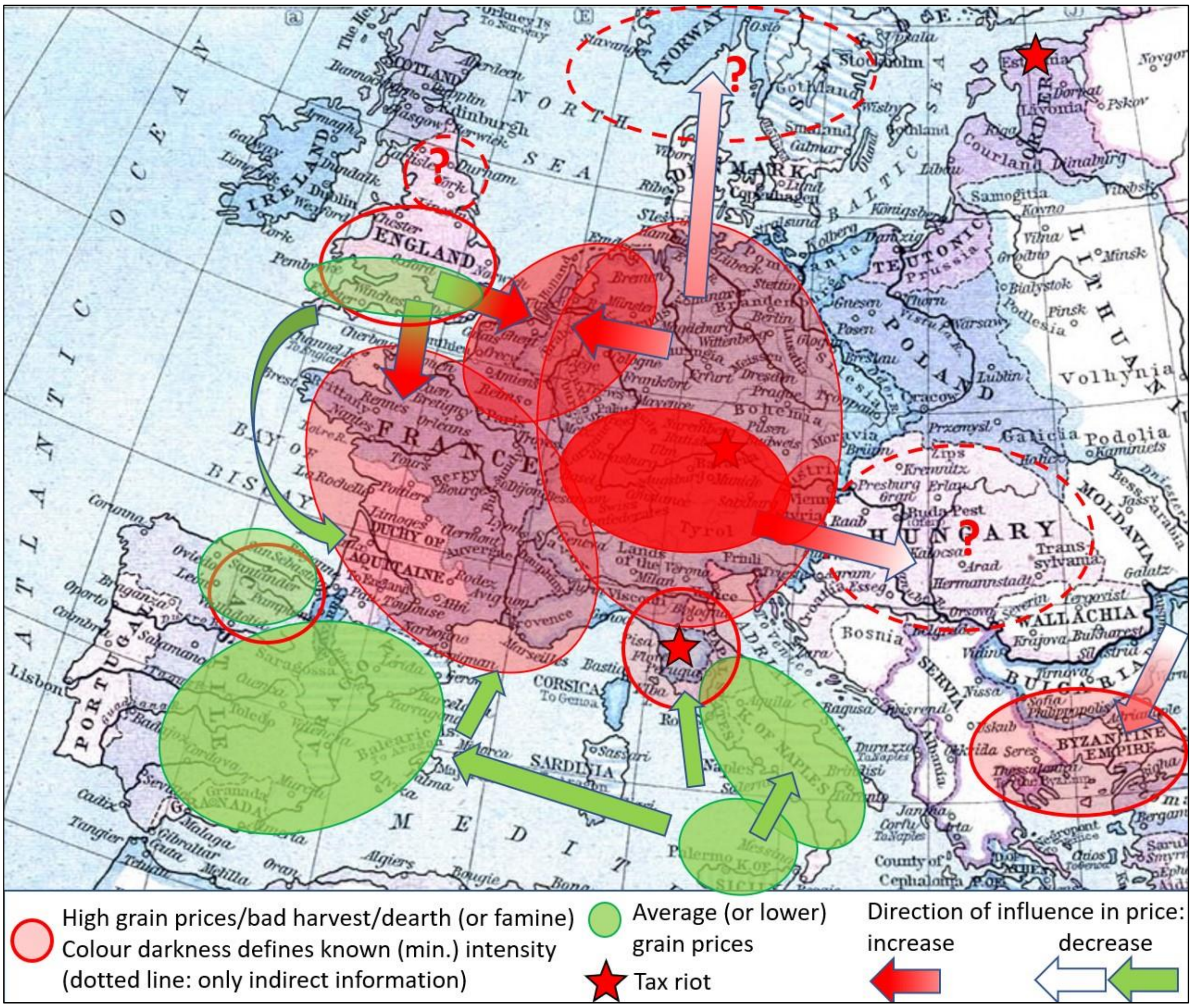

Fig. 3.1.1 Subsistence crisis intensity & grain (beverages) prices in Europe. Normal (green) and high (red) prices and the potential price increasing and decreasing influences of the different countries/regions in Europe (base map;[171] data sources: Supplementary Information 3.1, Supplementary Data 3)

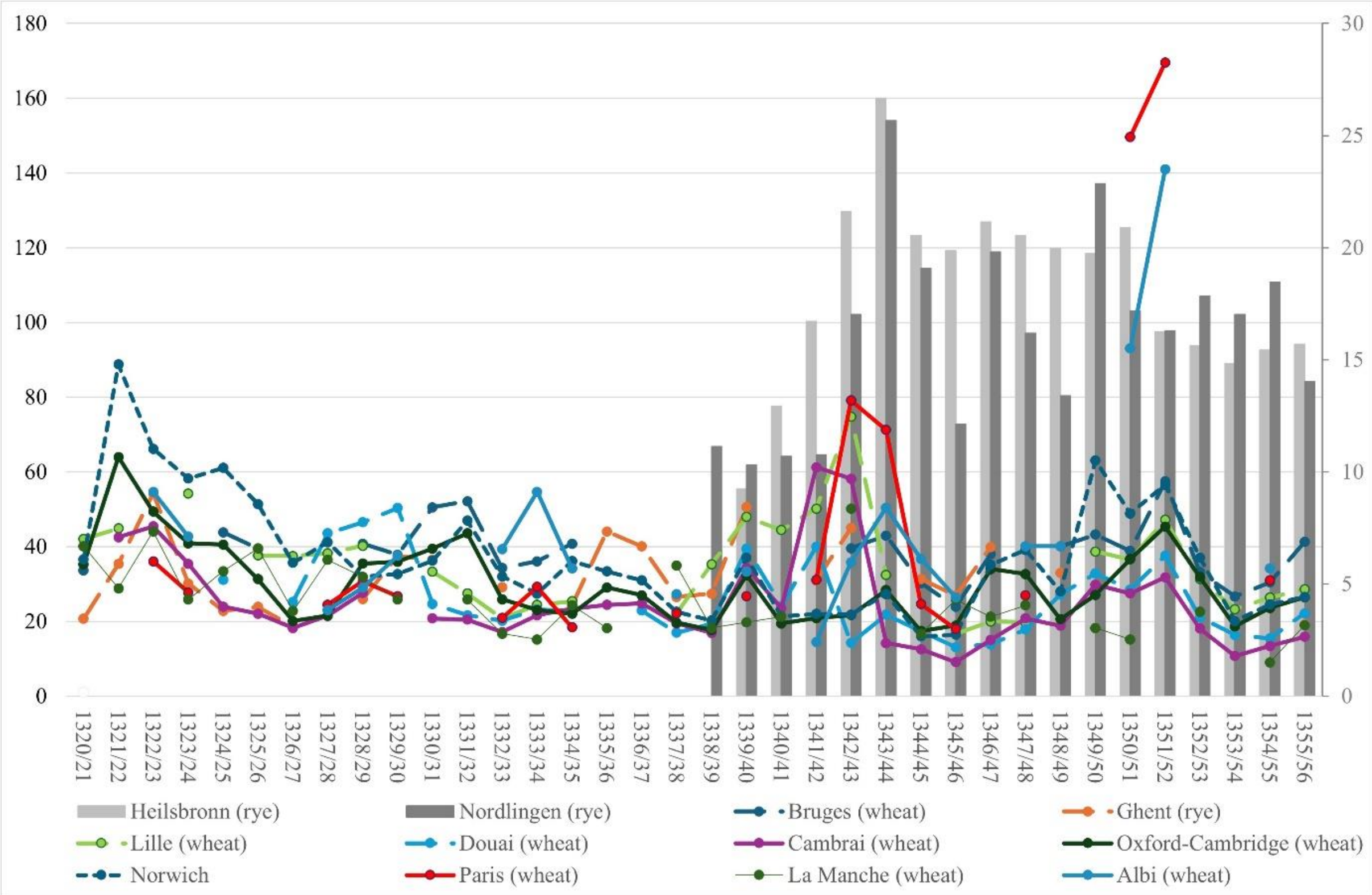

Fig. 3.1.2 Wheat and rye prices in Western and Central Europe (gr Ag/hl). Sources of price series: Paris–France,[172] Albi–France,[173] Heilsbronn, Nordlingen–German kingdom,[174] Norwich–England,[175] La Manche, Normandy–France,[176] Bruges, Ghent–Flanders and Lille, Cambrai–France,[177] and Oxford/Cambridge–England.[178,179] The German (Nürnberg area: Heilsbronn, Nordlingen) series data are presented as columns (with a secondary axis)

Below, a short European overview of food availability, shortage and price information is provided according to countries and regions, with special emphasis on grain prices and shortage (if available):

1) Norway(-Sweden): In 1343, alleviation of customs for German grain – indicator of shortage of (exported) grain in (West-)Norway(-Sweden?) - more German merchants in Scandinavia. No price lists or direct contemporary evidence, but legal act suggests grain shortage (shortage of imported grain) in 1343 (at least).[180]

2) German-speaking areas/Holy Roman Empire: Bavaria, Swabia & Austria (including today's Switzerland or large parts of it): high prices, (great) dearth (bad harvests and great loss of harvests in both years) starting from 1342, continuing in 1343 and 1344. The rest of the German kingdom also suffers from harvest loss and related subsistence crisis (references: see Supplementary Data 3). Price series in the broader Nürnberg (countryside) area suggest 2-3 times more grain prices,[174] dearth and famine hit Bavaria and Allgäu according to Johann von Winterthur,[56] and the Annals of Stift Neuberg clearly suggests dearth/very high prices in Austria,[181] while other, indirect evidence on loss of/weak grain harvest is also available in other parts of the Empire).[8]

3) Low Countries: grain price crisis (largely missing grain export from the German areas + Hundred Year War related; although the Netherlands and Brabant were exempt from the English embargo).[180,182]

4) England: around normal grain (salt, cattle) prices in south of England (e.g., Exeter, London, Lowland England) in 1342 and higher in 1343 (esp. wheat). However, the nominal value of wages was lower in 1342-1343 than in the previous and following years (until the Black Death). In the north of England (York area) rain-related grain harvest problems (see also Supplementary Information 2.2: 40) might have increased prices (no price data available in the north).[183,184] Low cheese and salt prices, medium (to higher) grain yields were typical in England.[185] The available datasets from south of England suggest average to good grain harvests in 1342 and 1343.[186]

5) Baltic: In Estonia, a great uprise of Estonians took place in 1343 because of Danish overtaxation.[187]

6) In Poland, Lithuania, Russian principalities: no price or food shortage data are available.

7) Hungarian kingdom: no price (or harvest-related) information available for these years. 14th-century parallels, the presumable price increase effect of the great dearth in Austria, and the extraordinary floods in both major river catchments (i.e. Danube, Tisza) may assume some price rise, at least.[16,188] The hurried coronation of the new Hungarian king in July 1342 (only 5 days after his father's death) and the higher number of (partly food and fodder related) violent attacks in these years – detectable in the available charter evidence in 1342 and 1343, in an otherwise politically stable period – may indirectly also support the idea that the unusual weather, the outstanding number of floods and the Central European subsistence crisis might have had some cumulative effect on the availability and prices of base food and fodder in Hungary (*Fig. 3.1.1*).[189]

8) Dalmatia: Dubrovnik wine prices in 1342 show normal/average values.[190]

9) Byzantium: relative high price of wheat in 1343 in Constantinople.[191]

10) Italy: in Venice, dearth and famine from the late 1330s continued in the early 1340s: continued and intensified in 1342.[192,193] In Tuscany and South-Italy, after the famine and epidemics of 1339-1341, somewhat better conditions in 1342-1343; food shortage and riots in Florence in 1343 (but it is largely also due to the political turmoil).[194-196]

11) France: great price increase, dearth both in the Paris region, Northwest-France, and the south of France in 1342-1343 and/or 1343-1344. Bad harvests in 1343-1344 in the Toulouse region (Occitania) and France in general. In Occitania and some other parts of France, dearth was eased by (English) grain shipped to Bordeaux (to the areas which were not under English grain export ban), and perhaps also by Mediterranean grain.[172,197]

12) Iberia/Spain (e.g. Toledo, Catalonia): no particular grain price fluctuation (close to normal conditions) in 1342, 1343;[198,199] Navarre 1343: high wheat (and hog) prices, but overall low-average commodity prices.[200]

After the dearth and famine of 1339-1340(-1341-) in the Central and Eastern part of Southern Europe (S-France, Italian states, Byzantium), 1342 and 1343 were generally better years in most of the Mediterranean, with grain price decrease. Note that, because of the Hundred Year War, price rise in western Europe started earlier, and to some extent the war also influenced prices in Northwest-Europe in 1342-1343, while the Byzantine civil war and the loss of Tana (and the Black Sea control of Italians) could also have a negative effect on grain price levels in Constantinople in 1342-1343. Some of the survival strategies and societal responses to the dearth and subsistence crisis were also included in contemporary source evidence in 1342-1344, and are presented in *Fig. 3.1.3*.

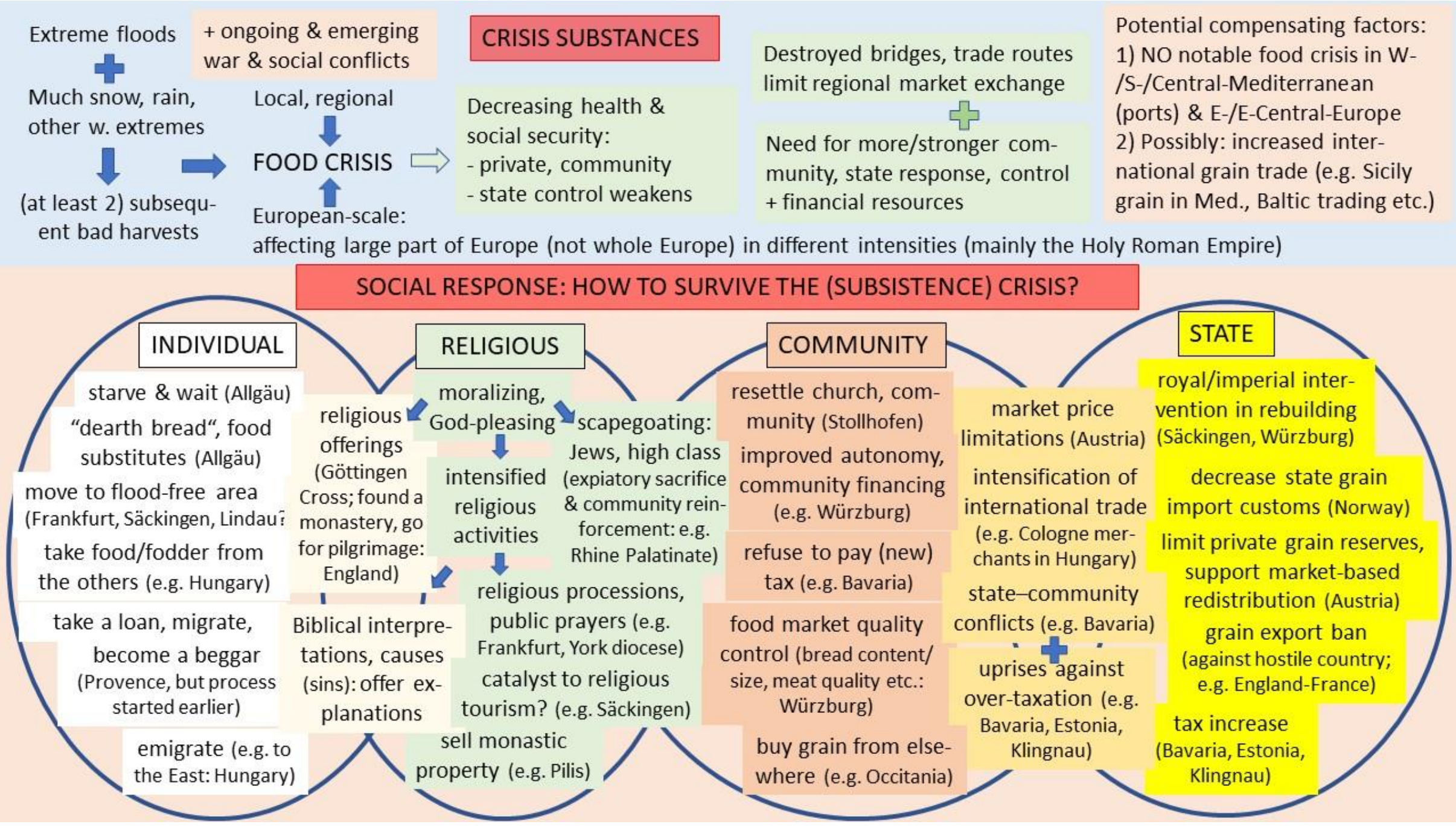

Fig. 3.1.3 Societal response, survival strategies during the subsistence crisis of 1342-1344: an overview (sources: Supplementary Information 2.2, 3.1, Supplementary Data 3). Examples: Allgäu, Bavaria, Rhine Palatine, Lindau, Klingau,[56] Göttingen Cross (SI 2.2: 57), York diocese (SI 2.2: 40), Säckingen,[56,138,166] Würzburg,[8] Frankfurt,[8,169] Stollhofen,[201] Austria,[181] Hungary, Pilis,[188,189,202] Denmark-Estonia,[187] Norway,[180] England,[203,204] England-France, Provence and Occitania,[197] intensified religious activities).[8,138,170] Note that the direct connection in most cases was not mentioned in contemporary sources, but the causal relationship based on local- and large-scale processes, contemporary socio-economic conditions and the consequences of anomalous weather and floods is either obvious or very likely. While many of the responses are considered as typical medieval societal reaction in times of environmental stress or crisis, some of the responses are new or even unique, not known from previous documentation

3.2 Example for indirect economic consequences: Salt production problems and their relation to weather and floods in Italy in 1342-1343

On 5 August (Julian Calendar) 1342, in Venice, officials notified that, due to the weather conditions, very modest quantities of salt had been produced "everywhere" ("*cum isto anno [1342] propter condicionem temporis sal factus sit in modica quantitate in omni parte.*"[205] probably meaning the seas around Italy, especially the Adriatic Sea with its seawater salines mainly under Venetian control. The Venetian source does not provide more precise information about the particular weather conditions that resulted in the bad salt "harvest" "everywhere", but leaves no doubt that anomalous or extreme weather event(s) caused this problem, and that the weather-related problems were spatially extensive enough, affecting large areas of the northern Mediterranean for a prolonged period of time.

Although, depending on the context, the Latin term *tempus*/*-oris* can mean both "weather" and "times" (in general), combining it with the *propter conditionem* makes it quite clear from a linguistic point of view that *tempus* here means the weather (environmental/climatic conditions) that was unfavourable for salt production. Moreover, in a political/social context the "everywhere" would be rather difficult to interpret in mid-14th-century Italy if the source combines it with "times" (given the fact that the political and social conditions of 1342 are outstandingly well documented and there is no political or social reason

in this year that could have so strongly impacted salt production on a spatially large scale), while – taking into consideration the ample source evidence in 1342 referring to the weather-related problems in Italy (i.e. sea surge, unusual cold, floods etc.) – the significant problems of salt production and the low produced quantities perfectly fit and complement the information collected from other contemporary narratives.

Further evidence of salt availability problems is known from the contemporary urban administrative documentation of the town of Bologna. On 23 December 1342, Taddeo Pepoli (the Signore of Bologna) imported an unknown quantity of salt (for 1974 Bolognese pounds) at his own expense.[206] On 4 January 1343, the city of Bologna imported salt from Cyprus via Venice and paid 1200 Bolognese pounds.[207] On 26 February 1343, Bologna imported again 1480 corbe of salt from Cyprus and Sardinia for 24 soldi per corba.[207]

In March 1343, salt was transferred forcefully from the countryside to the city of Bologna.[207] On 31 March, 5 and 20 April Bologna imported 647 corbe of salt from Venice, and again ordered the transfer of unspecified amounts of salt from the countryside to the city.[207] On 17 and 31 May 1343 37 and 100 corbe of salt were taken from the countryside to the city and sold for 25 and 29 soldi per corba, respectively. Then, on 6 June 1343, 32 corbe of salt were taken from the countryside to the city again and sold for 25 solidi per corba.[207]

On 2 July 1343, Bologna imported 627 corbe of salt from the countryside worth 200 Bolognese pounds and sold it (in the town) for 30 solidi per corba; the town also imported 197 corbe of salt from Cyprus and sold it for 29 solidi per corba. On 6 July 1343, 1181 corbe of salt were imported from Venice and sold for 28 solidi per corba, and 197 corbe of salt were imported from Cyprus and sold for 29 solidi per corba. On 10 July 1343, a messenger acting in the name of Taddeo Pepoli was paid for his 13-day stay in Venice, where he had to look for salt from Cyprus, but also from the region, for example, from Cervia and Chioggia.[207]

On 5 September 1343, Bologna imported further 880 corbe of salt from the countryside, and sold this salt for 28 solidi per corba.[207] On 10 November 1343, the town imported 1652 corbe of salt and, finally, on 30 November 1343, Bologna again imported 1768 corbe of salt from the region, sold for 28 solidi per corba.[207]

The note about Bologna importing large quantities of salt from a distant location such as Cyprus becomes particularly significant when considered in its historical context. In this period, Bologna mainly bought salt from Venice, as in Italy in the 14th century the largest salt production centers were located in the Venetian lagoons. When, however, these local/regional markets could not supply sufficient salt, Bologna – mainly through Venetian merchants – typically sourced it from the Adriatic (i.e., the western or eastern Adriatic). The fact that they also had to import salt from Cyprus (partly themselves, partly by Venetian merchants) suggests a broader regional shortage – one likely affecting extensive parts of the northern/central Mediterranean – where salt was not available in the required quantity or at a reasonable price.

At the time, due to the papal embargo on Muslim trade and goods, Cyprus acted as a key intermediary: Muslim goods were transferred there, sold to Christian merchants, and thereby rendered tradable in the Christian world. Although the pope lifted this embargo in 1342 – likely in part because internal Christian trade could not meet demand, and maritime transport could "help out" in times of local-regional shortages and/or remained cheaper than overland routes – Cyprus retained its strategic role in international trade for several more years, though with decreasing importance.

Thus, the mention of Cypriot salt likely indicates that the salt purchased by Bologna had originally been produced in the Muslim world and shipped from the eastern Mediterranean over a considerable distance. This implies that salt production or availability was significantly

reduced – not only in Bologna, Venice or the Papal State (to which Bologna belonged at that time), but likely across much of Italy and the central Mediterranean, including parts of modern-day Greece.

Political disruptions – such as the Byzantine Civil War (in these years mainly affecting the area of today's eastern Greece), the loss of Tana in the Black Sea to the Golden Horde (which disrupted Italian Black Sea trade), and the 1342 conflict between Venice and the Hungarian crown over Dalmatian towns – may have contributed to supply issues. However, these conflicts were not directly impacting Bologna (or the trade in the Papal State) and the Venetian lagoons to an extent that would fully explain such a widespread salt shortage. Even the internal conflicts in nearby Florence had limited impact on broader trade networks.

More significantly, extreme weather conditions – persistent rain, storms, convective systems, and the floods in 1342-1343, which severely affected the warm season – likely played a major role on a large spatial scale in disrupting salt production (e.g., through the severe flooding of salt pans in the Venetian lagoons at least twice in these two years) and distribution, making meteorological factors a strong candidate for the root cause of the shortage.

4 Event descriptions and modern parallels

4.1 Event 1) Nov-Dec. 1341 – closest parallel: December 2009–January 2010

Floods: NE-Carpathian Basin, SW-Balkan (heavy rainfall, presumably also snowmelt)

Cause: heavy rainfall (possibly also snowmelt) – mild-wet S/SW air masses

In late 1341, flood reports are available in the Northeast-Carpathian Basin (River Rima; tributary of the River Sajó/Slaná) and the Central Balkan, on the River Evros (Supplementary Data 1; historical analyses).[212] Moreover, as the perambulation affected areas close to the inflow of the Rima into the Sajó/Slaná, and also because the discharge of the Rima is not notably smaller than that of the Sajó/Slaná, presumably the Sajó/Slaná was also in flood.[213] In late November 1341 a great loss of trees, caused by the heavy snow, was described in Constantinople (İstambul-Tr). After that, a severe cold winter-spell caused great loss of pack-animals during a military campaign in Thrakia (Edirne-Tr). This was followed by the heavy rainfall that reportedly caused the flood of the Evros in December (Supplementary Data 1, 2; in accordance with typical flood timing).[214] The flood was preceded by frosts and great quantity of snow at the Bosporus and in Thrakia (NE-Greece–W-Turkey) at the end of November/beginning of December and, therefore, the Evros flood was probably the result of snowmelt and heavy rainfall. After an autumn with unusually great temperature variability, the Merle weather diary[158] in the south-east of England mentioned rains almost every day, sometimes with strong winds or storm (even if, as Merle stated, the rains penetrated the soil, and no flood reports is known from the British Isles). In contemporary sources, the early part of the winter was generally described as severe, with much snow (Supplementary Data 2). Although without specification on the exact timing (or flood type), flood was also mentioned in south of England in winter 1342 (Supplementary Data 1).

The occurrence of great floods in both regions within a couple of weeks is relatively uncommon but not unusual in the 20th-21st century, and requires specific hydroclimatic conditions. The closest modern parallel, with notable flooding on the Sajó/Slaná (and its tributaries: snowmelt and sudden heavy rainfall caused flash flood) and the Evros, is the mid-December 2009–early January 2010 European flood (*Fig. 4.1.1*). If we also consider nearby river catchments (e.g. in E-Slovakia), considerable floods already started in mid-November.[215-217]

In 2009, October and November were rich in precipitation in East-Central and South-east Europe. After that several days of frosts with snowfall arrived in mid-/second half of December, then rapid melting and mild-wet weather conditions occurred, followed by predominantly frosty January with multiple snowfalls and mild interruptions.[218,219] In 1341, one month earlier than in 2009, rather similar weather conditions could prevail in East-Central and South-east Europe, with generally higher (West-)Central European mean temperatures in December 2009–January 2010. In January 2010 higher than usual pressure conditions prevailed in Eastern Europe,[220] influenced by the southwestern expansion of the Siberian high, while the lower than usual pressure conditions prevailed in the Mediterranean – altogether responsible for the prevailing frosty conditions with snowfall and also the frequent mild and wet interruptions particularly in East-Central and South-east Europe. As the precipitation totals in

January 2010 show,[221] not necessary the monthly quantity but often rather the temporal distribution of the rainfall, combined with the changeable temperature conditions,[219] with rapid snowmelt in the (lower) mountain areas, were responsible for the floods.

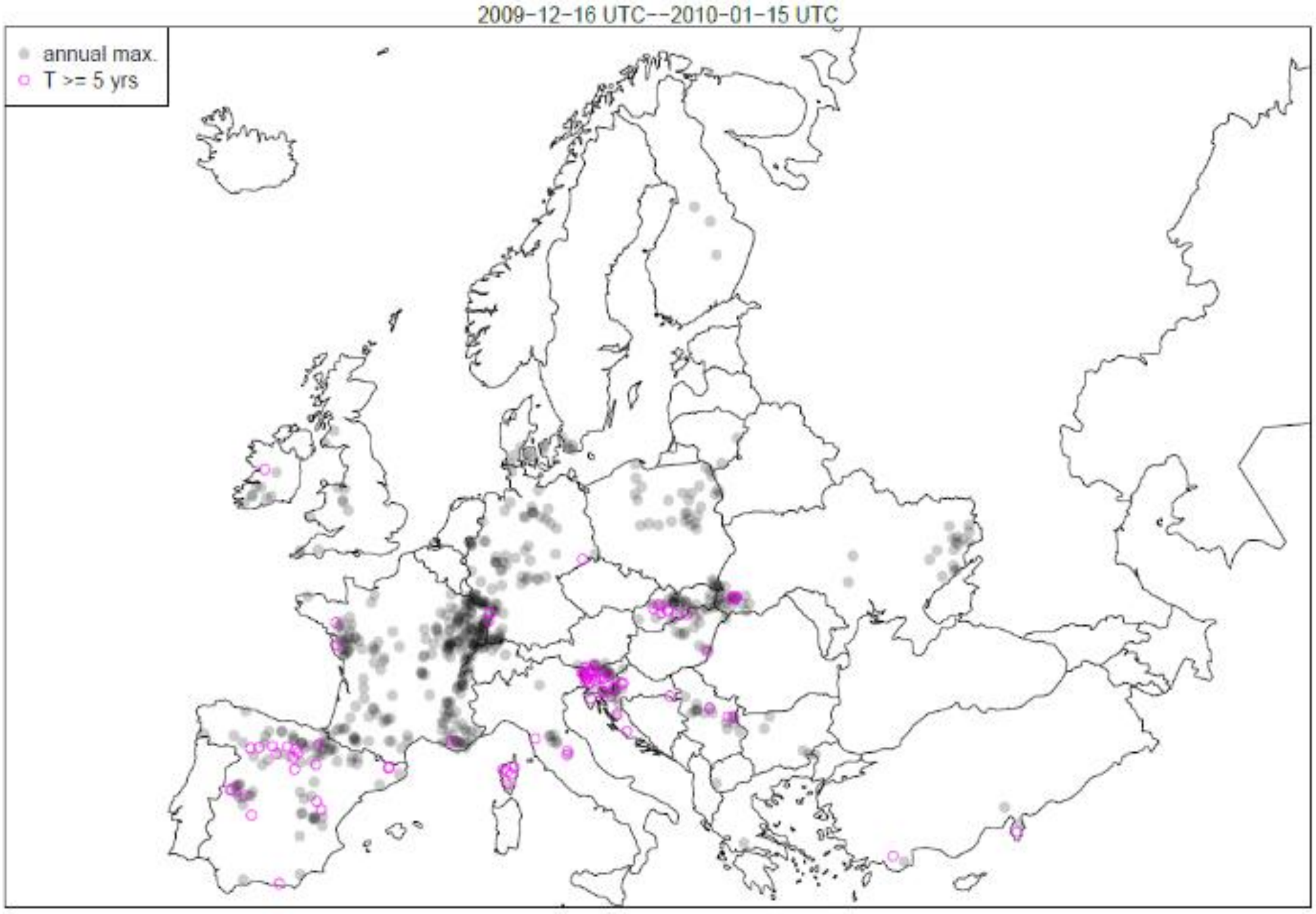

Figure 4.1.1. Floods exceeding the 5-year return period limit and/or with annual maximum reached between mid-December 2009 and mid-January 2010 in Europe, based on the pan-European flood database[1]

Based on the Pan-European Flood Database[1] and other, national water-level/flood databases in early/mid-winter 2010 there were two, large-scale flood waves: The first one took place at the end of November and first half of December, while the other, more intensive and spatially extensive wave occurred from late December to January.[1,214,222-225] Only the second large-scale flood wave (Dec. 2009–Jan. 2010) resulted in floods in both the NE-Carpathian Basin and the Balkan that could be comparable to the November–December 1341 flood events. Like in other cases (e.g., Dec. 2014, Nov. 2019, Jan. 2021) showing some similarities (but also considerable differences) in the flooded rivers, causes, seasonality and (esp. Balkan and Central) European weather conditions, from late December 2009 there was a spatially extensive flooding in Europe, affecting Western, Central, South-eastern and some parts of Eastern Europe, where mainly the (smaller) tributaries and less the major rivers were in flood (*Fig. 4.1.1*). Even if the areas affected by floods in late 2009–early 2010 were spatially extensive in Europe, the more considerable magnitude floods were concentrated to the N-Balkan, the Carpathians, parts of the Western Alps and Spain.

In most areas in late 2009–early 2010 the floods were rather moderate and generally less intensive than the floods reported at the end of 1341 (in magnitude the latter were more comparable to the May-June 2010 Central-European floods). As in late 1341 the two floods were specifically reported in areas that are rather prone to the occurrence of cyclones following the Vb path, and the European late autumn–early winter weather (and large-scale climate mode: NAO-, AO-) patterns also supported the development of Vb events, there is a possibility that

the floods in the NE-Carpathian Basin and the Balkan were related to a Vb event. In general, the reconstructed synoptic patterns[220] also show considerable similarity to the large-scale (NAO-) patterns suggested by the current paper (in accordance with the NAO reconstruction).[73]

Despite the somewhat limited spatial overlap (e.g., there is no data for the Balkan in 1784), the weather and floods in late 1341 show some parallels to the December 1783–January 1784 floods[79] that affected the British Isles, today's North-France and Belgium, and the eastern, north-eastern part of the Carpathian Basin. Just like in late December 1783 early–January 1784, after a severe early winter with much snow, primarily the eastern part of the Carpathian Basin was hit by floods in late November 1341. Merle in S-/SE-England also mentioned[158] that in 1341 snowfall was followed by rain, strong winds and thunderstorm in late November, then strong western wind in early December and west/south-west winds in mid-December (see Supplementary Data 2).

4.2 Event 2) Candlemas Flood: February 1342

Floods: W- and Central Europe

Causes: snowmelt, sudden ice break-up and jamming, rainfall (western/south-western mild-wet air masses arriving from the southwest)

An extensive, outstanding European flood, affecting Western and Central (including East-Central) Europe, started in early/mid-February 1342. The winter was severe with much precipitation in large part of Europe, with much rain in the west, in England, especially in its later parts. In Central Europe, the winter until February was described as extremely hard and snowy; in Bohemia, for example, lots of people in many places died in the severe coldness (see Supplementary Data 2). In his weather diary, William Merle reports[158] on a sharp change in weather conditions in the last days of January. As amongst others Francis of Prague also described,[58] on 1 (Gregorian Calendar – hereafter GC: 9) February warm southern winds arrived, followed by spring-like rain ("*calido vento australi praeambulo, quem pluvia quasi vernalis fuit subsecuta*"). Exceptionally strong wind was documented in the Villani chronicle for 2 (GC: 10) February 1342 in Florence, Italy (Supplementary Data 2).

Due to very mild and humid air masses, sudden snowmelt, mild rains and the firm ice cover on the rivers, great flood started on 1 or 2 (GC: 9-10) February on the Vltava/Moldau river ("*factum est grande diluvium per impetum aquae nivialis et pluvialis*"). Francis of Prague also mentioned that in those times very great floods occurred not only in Prague, but in the 'entire world' ("*In toto quoque orbe terrarum tunc temporis fuerunt maximae aquarum inundationes*").[58] The extreme flood and the immense amount of ice pieces ("*et propter ingentem molem glaciei*") in Prague and along the Vltava caused horrible damages: two-third of Prague's iconic Judith Bridge, one of the largest stone bridges of Europe at that time (built in 1170), many houses and all mills were destroyed, people in villages along the river suffocated and many animals died. Along with the ones in the 1108 and the 1432, in Prague this flood was reported to be the most destructive flood event of the Middle Ages (Supplementary Data 1–3).[65,76]

Similarly, the great Elbe flood destroyed the stone bridge in Dresden on 2 or 3 February (GC: 10, 11 February), while another source dated it for a week later. The flood also damaged

the bridges in many other places ("*in aliis multis locis*") along the River Elbe. Although with only annual dating, possibly the major flood that caused great devastation and loss of settlements and islands in the Ditmarschen at the Elbe estuary could be also related to this major late-winter Elbe flood. Winterthur also mentions an outstanding Danube flood (probably on the upper sections?) around 2 (GC: 10) February: accordingly, the flood extended two miles in length and one mile in width, causing extraordinary destruction in an unusually large area. Winterthur also heard that the water in the flooded area devastated all sowings, fruits (practically everything), and killed 6000 people. In the western German territories, the February floods on the Rhine and Main were reported somewhat later, in the second half of February, but possibly it was still part of the same major European February flood event (for references, see Supplementary Data 1). Other, potentially related case is available in (present-day) N-Poland, where an important embankment/dyke was built in 1343 along the Nogat (a major branch of the River Vistula at its lower sections) in order to defend the area from floods. Although no flood is directly mentioned in this case, but the timing of the significant improvement of flood prevention infrastructure is rather thought-provoking (see Supplementary Data 3).

Great flood of waters obstructed the legal process (perambulation) around 9–11 February (GC: 17–19) in NE-Hungary (Déta and Korh villages) near the Rivers Hejő, Szinva and Sajó, close to their inflow into the River Tisza (probably also referring to the influence of Tisza high waters or flood). The water might have decreased by the next day, because – according to the charter – on 12 (GC: 20) February one part of the field survey could already be carried out. Around the same time (11-17 February; GC: 19-25) the Loir was in great flood at Vendôme (W-France). A (sudden) great flood was also documented on the Seine between 6 and 24 (GC: 14 February–4 March) February (other dating: 22 February) at Rouen and Martainville, in NW-France. The Eure flood at Chartres (only dated by year) might have also related to the same February (and March) flooding. According to the *Nota Rotomagensis* (quoted/embedded in a later work), half leg or higher water suddenly appeared in the monastery, the cloister and the refectory of Notre-Dame-du-Parc (Rouen). The flood destroyed four pillars of the bridge in Rouen, killed many people and caused very great damages. As suggested by Francis of Prague,[58] floods of more rivers also occurred in Europe, but remained unreported (for further references, see Supplementary Data 1).

Parallel to the great early February flood wave, Johann von Winterthur also mentioned the great sea flood (*Aqua Alta*) in Venice ('around the same time' – i.e. early February in JC). A description about the highly destructive great storm surge in Venice can be also found at Francis of Prague,[58] while Italian sources consequently dated it to 25 February when the water rose in the canals much more than usual, and the accumulation of water destroyed many goods. Around the same time exceptional snow was also reported in Bologna. In the Villani chronicle, there was a windstorm reported in Florence on 2 February. Moreover, important river-ice related damage was reported in the Rhone delta in February at Arles, only some km-s from the Mediterranean Sea, suggesting massive ice pieces/ice flow on the river (Supplementary Data 1-3). Based on the analysis of Pichard and his colleagues,[226] in the second half the 14th century (no analysis available for the first half of the century) ice in such quantity was only documented in 5 years on the river, and only in 7 years in the 15th century. The frequency of mentioning (significant) ice on the river greatly increases from the mid-17th century, but in the 14th century it is still a rather rare phenomenon.

The great European flood, its weather background, causes, extension and development as well as magnitude show strong similarities to the winter of 1783-1784, as a consequence of the outstanding Laki eruption: in this case at least 3 extensive European floods were reported: one in December that affected England and the Carpathian Basin, especially its eastern parts. The second, greatest flood started (just like in 1342) in early February and affected the today's

areas of N-France, Belgium, Germany, Czechia, Austria and Hungary, covering almost the same areas as the February 1342 floods. A third European flood happened in April 1784. [342]

In the period between 1960 and 2020 winter floods show no such close similarities to the February 1342 event as, for example, in 1784; a relatively close modern (20th-21st century) parallel of this major European flood event (yet mostly without the ice jam floods), caused by snowmelt and rainfall (after a snow-rich winter), is probably the spatially quite extensive March-April European event in 2006, primarily affecting Western and Central Europe (although without the Venice flood; see *Fig. 4.2.1*).[227-232]

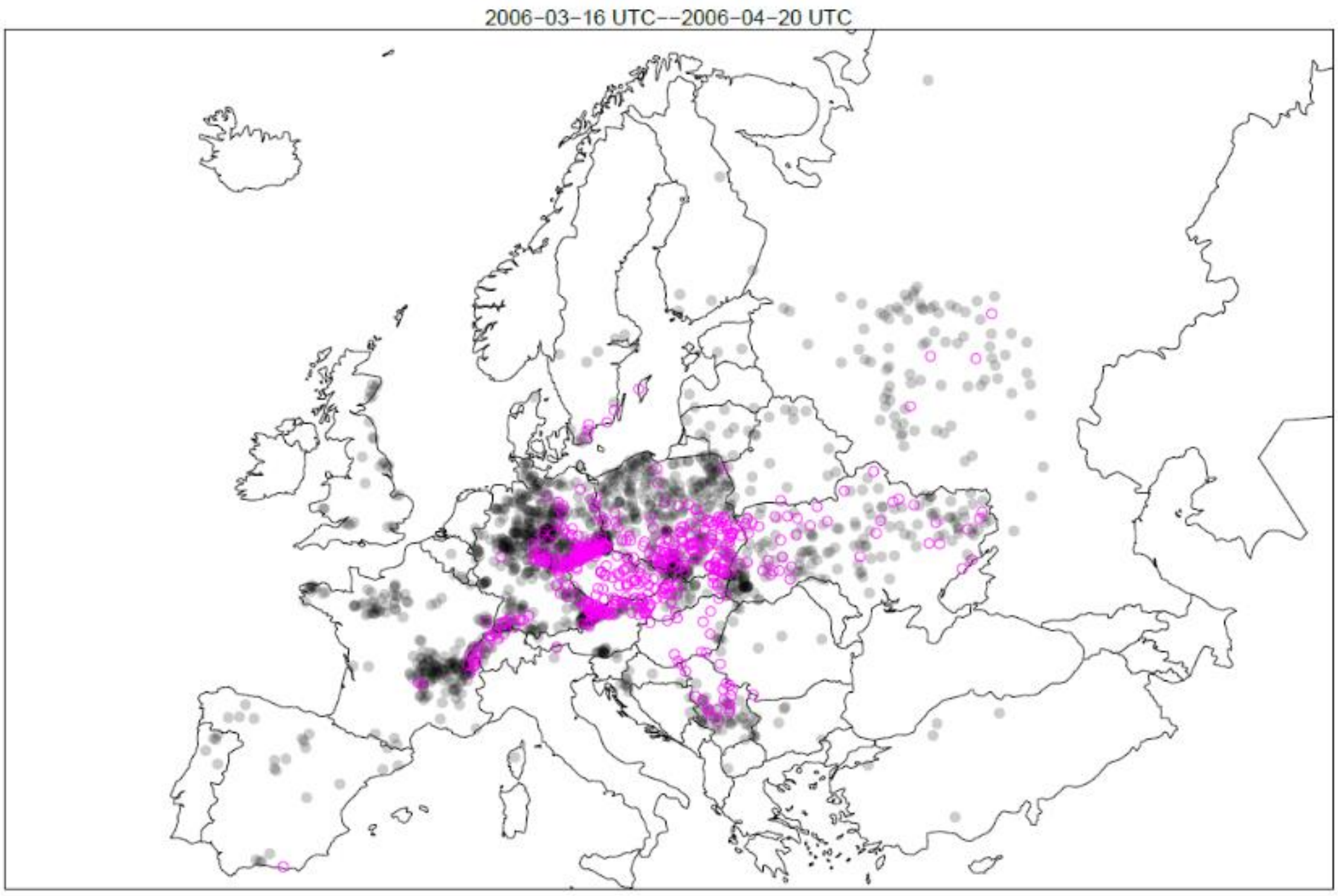

Figure 4.2.1 Floods exceeding the 5-year return period limit and/or with annual maximum reached between mid-March 2006 and mid-April 2006 in Europe, based on the Pan-European Flood Database[1]

Other European floods, showing some similarities to the February 1342 event occurred for example in (late January–)February 1967 when the Upper Danube, the Vltava, the Elbe and the Oder were in flood (with a changeable winter, occasional harder frosts and snowfall, and mild interruptions); the mid-March floods in 1963 (after a rather cold winter). Or in February 1966 when the hard and snowy winter was followed by milder February and then cold March (just like in 1342 when late winter–early could be again colder), but in this case there was only small flood on the River Vltava (which could have been perhaps the case also in 1342 if there were no ice jamming), but there were considerable floods on other Central European rivers. However, the 1960s floods usually did not affect Western Europe (i.e., were spatially less extensive).

In conclusion, the long-term cause of the extraordinary magnitude and spatially extensive European flood in February was the severe, snowy winter prior to late January 1342 and the immediate cause was the sudden strong mild and wet air masses, arriving from the south, south-west. Depending on the location and river, the floods of early/mid-February (lasting for weeks) were related to snowmelt, rainfall and river ice jamming. The flood wave

affected most of Europe from Western/North-western, Central, East-Central and parts of Southern Europe.

4.3 Event 3) March 1342

Floods: East-Central Europe (Upper-Tisza), Balkan (Axios)

Possible causes: snowmelt, rainfall (not directly mentioned in source)

Outstanding magnitude floods were reported on the Upper-Tisza in the Carpathian Basin and on the Axios (Vardar) in the Balkan in (mid-)March; and the (February) Seine flood continued in early March. The Eure flood at Chartres (only dated by year) might have also related to this and/or the February flooding (Supplementary Data 1).[212] The early part of March was cold in Southeast-England and frosty with snow in northern and north-Central Italy. The river navigation was still obstructed in March (until ca. early April) on the lower section of the Rhône between Arles and Avignon (in February it was the river ice that damaged the cargo ship), in Southern France, where problematic (harsh/cold?) weather conditions continued until around early April (Supplementary Data 2). Similar to the late November–December 1341 floods, the co-occurrence of River Tisza and Axios floods, especially very great/outstanding floods are uncommon but not unusual,[233,234] and in all such cases in the Pan European Flood Database[1] it is related to extensive flooding in Central and Southeast Europe, typically with multiple great or outstanding floods/inundations lasting for weeks.

In the period 1960–2020, the closest match is the March(-April) 2006 floods events that affected Bulgaria and also N-Macedonia. In 2006, River Tisza had notable and great floods in March and April: the Upper-Tisza and its tributaries flooded already in March and floods continued into May.[235,236] The mid-March flood in 2006 affected the northern and central parts of the Balkan, the Sava in Croatia and Slovenia; caused extensive flooding in France, the Low Countries and W-Germany and to some extent in eastern, SE-Germany. Floods also affected N- and N-Central Italy, in some parts of Spain and the UK (see *Fig. 4.3.1*). As for further, more historical parallels, another close match from the 20th century is March 1940 when, after a cold and snowy winter in Central and Eastern Europe, floods affected N-Greece, Southwest-/West-/Central/Northeast-Hungary, East-Austria, Southwest-Ukraine and the Czech Republic.

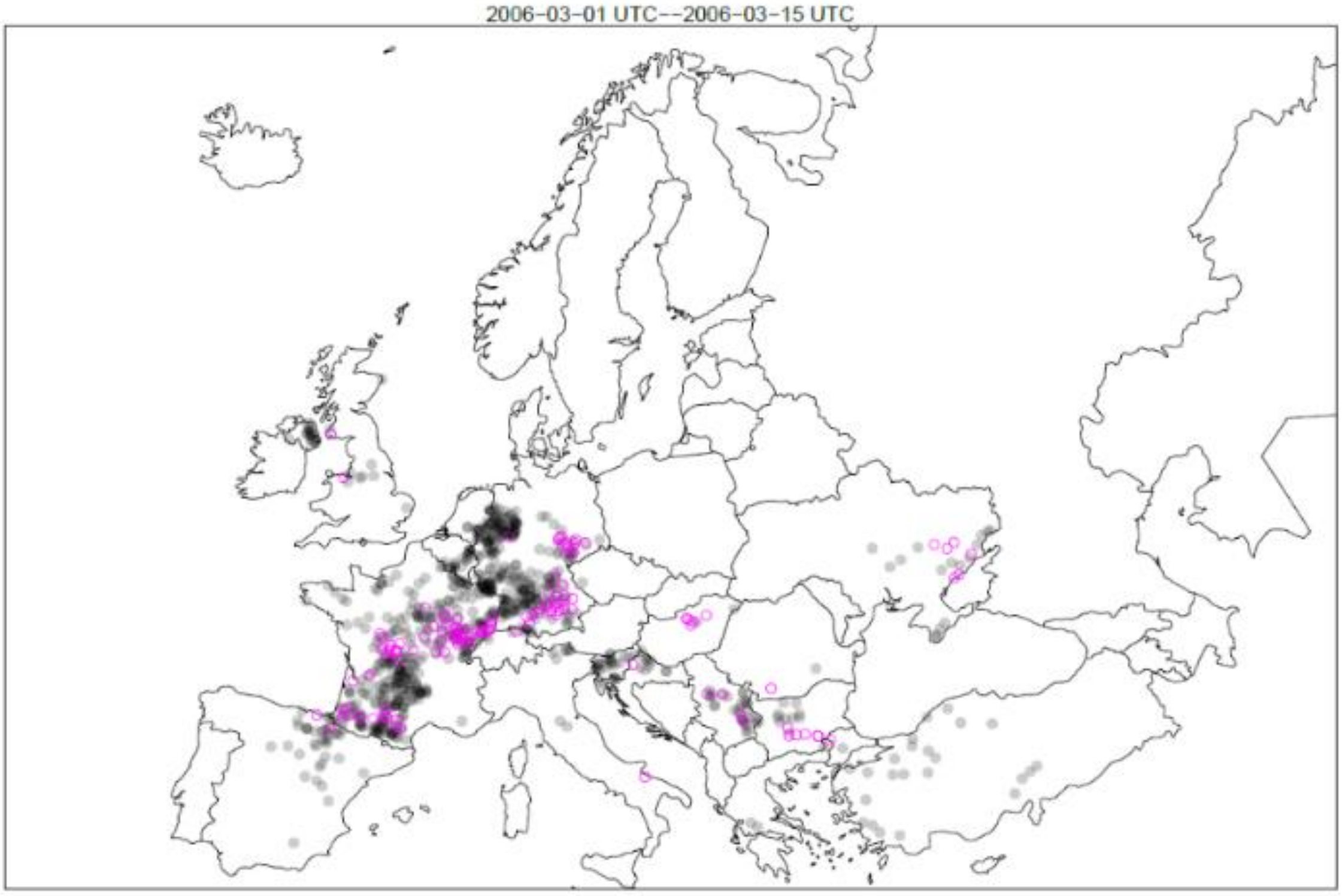

Figure 4.3.1 Floods exceeding the 5-year return period limit and/or with annual maximum reached in early/mid-March 2006 in Europe, based on the Pan-European Flood Database[1]

Although the cause of flooding was not mentioned in contemporary sources, based on the preceding weather conditions (i.e., preceding snowy, hard winter conditions with rapid mild interruptions), just like in 2006, the high-magnitude flood wave in Central-Europe and the Balkan could be primary caused by rapid snowmelt after a particularly snowy winter, also influenced by rainfall. Taking into consideration the early March cold, frost and snow reports in the northern part of the Central Mediterranean (and simultaneous report in SE-England), possibly the cold and wet intrusion in (Central) Europe (and N-Mediterranean) was followed by the arrival of mild air masses. The fact, however, that no particular weather change was described by Merle until the second half March may suggest that the sudden thawing did not affect (at least) the most westerly parts of Europe. Concerning the Balkan flood(s), based on the 2006 case, there is a possibility that, for example, the Evros was also in flood; however, as the only documentary evidence available from the region is primarily related to army operations at the lower sections of the Axios, whatever happened in the Evros area at that time, remained unrecorded.

The March 2006 daily (and hourly) precipitation totals[237] and the timing of the following flood in the Tisza catchment may show interesting further parallels: in 2006, a great peak in daily (and hourly) precipitation totals occurred on 5 March (preceded and followed by rainy weeks), especially in the Transcarpathian Region (today's W-Ukraine).[238] Although no weather data are available in 1342 from this area, a contemporary charter reports about a flood of extraordinary magnitude on 14 March (and the following days) on the Upper Tisza, collecting the waters of roughly the same area of the highest precipitation totals of March 2006.

The case in Arles and Avignon may also provide some interesting, additional information: Based on the flood situation in March(-April) 2006, and also based on the European and local, Rhône-catchment related weather and flood information available from

1342, it is rather possible that the ice pieces that were strong enough to severely damage a cargo ship in Arles did not develop in the Rhône delta near (already under the influence of) the Mediterranean Sea, but were rather originated from the upper sections of the Rhône. During the durable and hard winter, massive ice cover might have developed on the river on its slower but more northerly sections, which were broken up by the intermittent mild/wet episodes in (late) winter and early spring. As the weather conditions even in early March were colder than usual also along the northern coast of the (Central) Mediterranean, it is possible that – as the papal accounts also refer to the unfavourable weather as a contributing factor to the unnavigability of the river – the ice pieces that arrived from the north stayed in the Rhône delta for even weeks and melted only slowly, with this obstructing the waterway until April. It is also possible that the Rhône was in flood (at least in February); however, no available contemporary source is available to support it.

4.4 Event 4) April 1342

Floods: Central Europe, Balkan/SE-Europe

Cause: thaw/snowmelt (and rain)

In the second half of April 1342, an unusually great and extensive flooding was reported at the lower section of the Middle Danube in today's Northern Serbia that, in combination with a Carinthian reference, suggest that at least some of the major tributaries of the lower Middle Danube (especially the Drava/Drau) were also in flood in April. Based on the location and magnitude of the reported event, to some extent, this flood also had to affect the Lower Danube. Primarily caused by rapid thaw, based on the same Carinthian reference, around (early) mid-/April presumably there were floods also on the major rivers in today's Germany and Austria (e.g., Rhine, Danube), and the March flood continued on the Axios/Vardar in the Balkan (Supplementary Data 1, 2).

Apart from the reference on the hard winter and the following thaw and flood in April, and the overall unfavourable March (for river navigation) in Southern France, no weather-related information is available from the flooded areas on a local-regional level; however, Johann von Winterthur mentions huge amount (and "inundation") of rains around Easter (JC: 13 April; GC: 21 April: see Supplementary Data 2). Additionally, in the weather diary of William Merle[158] warm weather is mentioned more often in the second half of March, and the same source suggests predominant warm and dry conditions (even drought) regarding the south-east of England in April (Supplementary Data 2). In contrast, the early May floods that affected large part of the Carpathian Basin in the north and east may indirectly suggest that April was predominantly wet (and/or great amount snow melted in the Carpathians).

Based on the timing, the affected areas and the reported flood type/cause, in the 1960-2020 period,[1] the case shows similarities, for example, to the late March–early/mid-April 2006 floods and, to some extent, to a number of other spring flood waves (e.g., in spring 1995, 2000 or 2001) when (as also reported in April 1342) snow and ice melting occurred rather rapidly in the hilly areas in early April. This flood was, in the most affected regions, larger than the floods in early March or in May 2006. In Central Europe, the April 2006 floods heavily hit the Middle and Lower Danube; besides, most rivers in Germany (including the Upper Danube) and large part of the Balkan were also hit by flood (see also Events 2 and 3).[230,240]

The cold March (following a snowy winter-early spring) and the sudden, great late-March temperature increase in large part of Europe in 2006 (*Fig. 4.4.1*), followed by intensified flood waves, shows parallels to the 1342 case. Moreover, April 2006 precipitation patterns[241] suggest that precipitation totals were also high particularly in Central Europe and the Northern Adriatic. In the Balkan, the spring 2006 floods primarily took place in the northern and central regions, including parts of North-Macedonia, but the highest magnitude floods rather occurred north to River Axios/Vardar catchment.

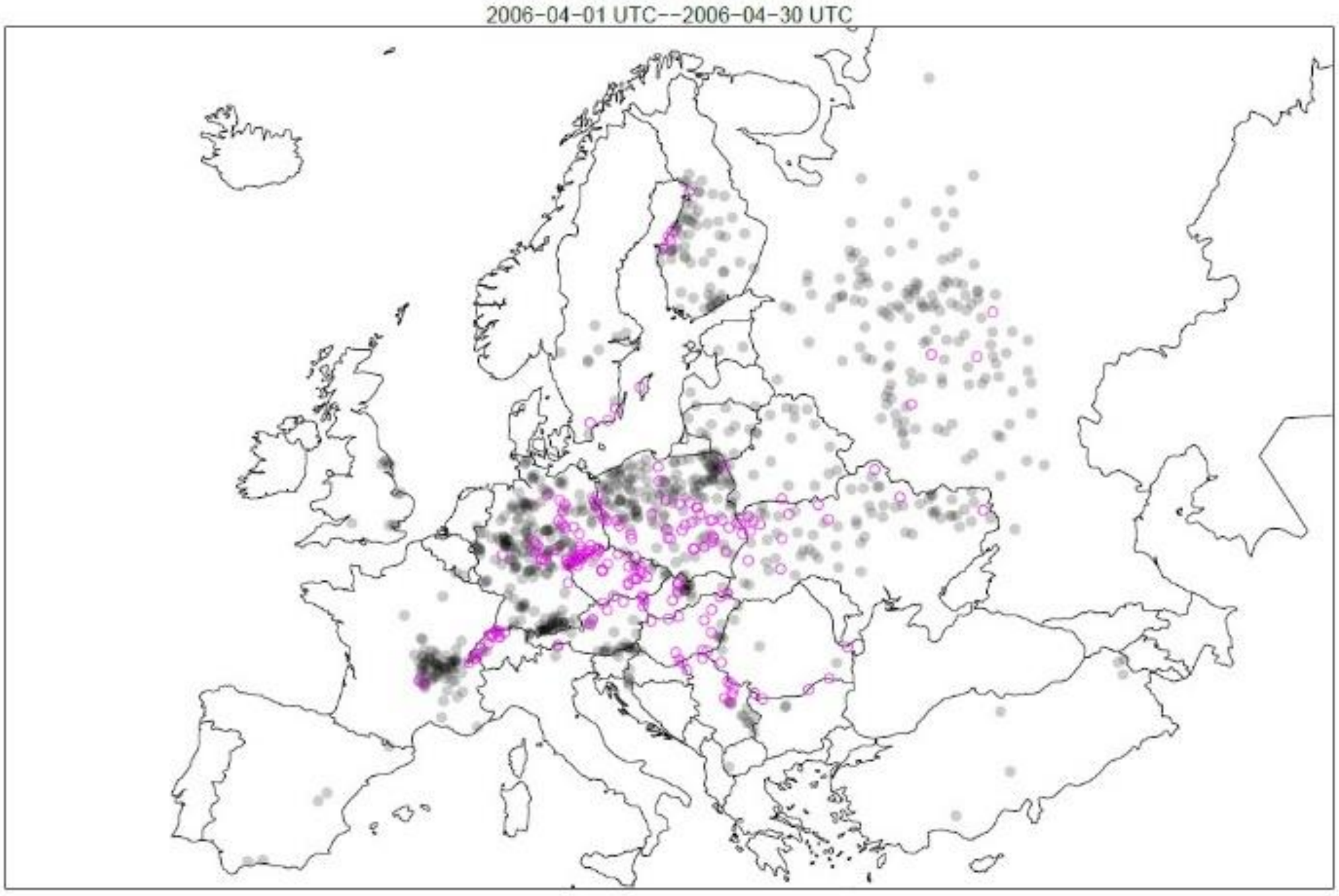

Figure 4.4.1 Floods exceeding the 5-year return period limit and/or with annual maximum reached in early/mid-March 2006 in Europe, based on the Pan-European Flood Database[1]

Although the April 1342 flood also shows parallels to the late March–early April floods in 1784 (as a consequence of the great Laki eruptions) – affecting the area of today's Hungary, Slovakia, Western Romania, Northern Serbia, Northern Croatia and even parts of Eastern Europe[342] (it is interesting that only about the flood on the Danube (and Central Europe) there is available contemporary evidence, while no flood data is presented from the Balkan in 1783-1784, an area heavily hit by the 1342 and 1343 floods.

4.5 Event 5) early/mid-May 1342

Floods: East Central and South-east Europe (Žitava, W-Carpathians; Middle-Tisza wetlands influenced by Tisza and Körös, E-Carpathian Basin; Axios/Vardar, Balkan)

Causes: rainfall

In early and mid-May 1342, floods were reported on the River Žitava in the Western Carpathians (W-Slovakia) and in the Sárrét wetland area supplied by the Middle-Tisza, the River Körös and Berettyó in Eastern Hungary. Flooding continued on the Axios (Vardar) in N-Greece (N-Macedonia; see Supplementary Data 1a.[158] While the large extension of waters in the Sárrét wetlands reflect on prolonged wet conditions and flood of the north-eastern (W-Ukrainian, E-Slovakian) and the N-/Central-Transylvanian catchment areas of the River Tisza in (winter-)spring, in agreement with the late spring-early summer precipitation reconstruction, the Central Balkan and N-Greece were wetter than usual in late spring(-early summer) 1342 (see Supplementary Data 2b). The sources mostly do not provide information on the main cause of the floods, apart from the general rainfall reference related to the Axios flooding), but in early May there was damaging thunderstorm in Western Germany and the April and early May had a generally drier and mild character in the south-east of England (Supplementary Data 2a) – these patterns are in agreement with the unusually southerly (Mediterranean) position of the North Atlantic Jet Stream in this year. These patterns increase the likelihood and could potentially be related to a Mediterranean cyclone that affected parts of the Balkans and East-Central Europe along a Vb (or Vc or d) path.

Taking into weather and flood reports, in the 1960-2020 period,[1] the most similarities can be identified with the period mid-/late April–early May 1995. This was a mediocre magnitude flood event based on the Pan-European Flood Database, but with strong flood clustering, affecting within a few weeks both Central Europe and especially the Carpathian Basin (including the Transylvanian Basin and the NE-Carpathian Basin) and also the Central Balkan with Northern Greece (the Axios catchment). Moreover, this intensive flood clustering in Central Europe and the Balkan can also be detected taking a more extensive time window (i.e., April-May 1995) that would fit well the longer-term spring conditions of 1342 (see *Figs. 4.5.1–2*).

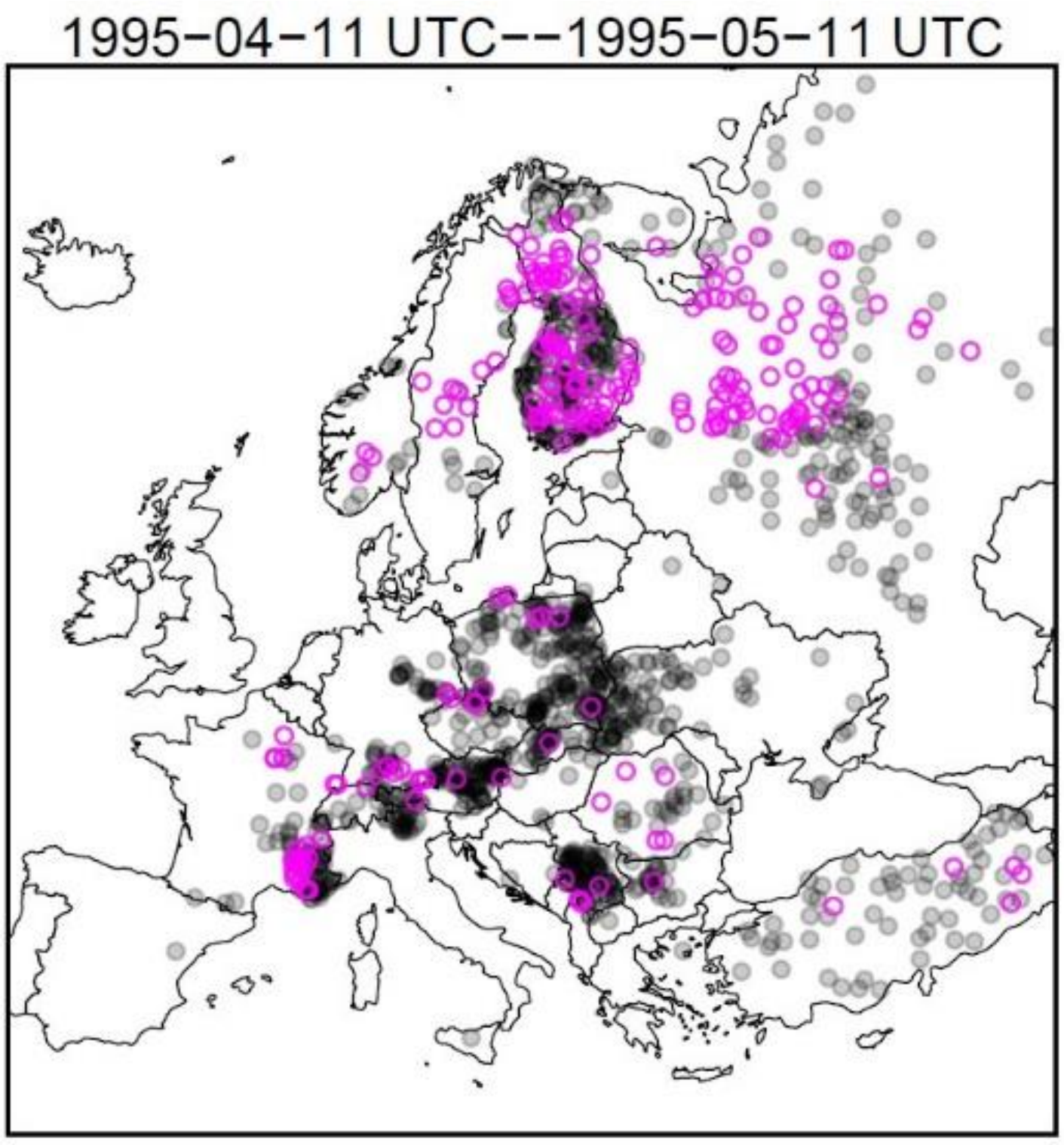

Figure 4.5.1 Floods exceeding the 5-year return period limit and/or with annual maximum reached between early/mid-April and early May 1995 in Europe, based on the Pan-European Flood Database[1]

Based on the Pan-European Flood Database and national databases,[1,235,236] in a lesser extent also the April 1972 (also with Axios flood), April-May 1987 (also with Axios flood, but not simultaneous flooding), the May 2006 and the (May-)June 2010 floods show notable similarities,[242] the latter ones without the Balkan/Axios flooding (in this sense, the March and the April 2006 flood waves still show somewhat more similarities to the May 1342 events than the May 2006 floods). The (mid-) May 2014 floods also show similarities in spatial extent,[242-245] seasonality and general weather conditions – with affecting the (W-Central) Balkan, the Carpathians, and the E-Alps – but without a particular flood on the Tisza and its tributaries.

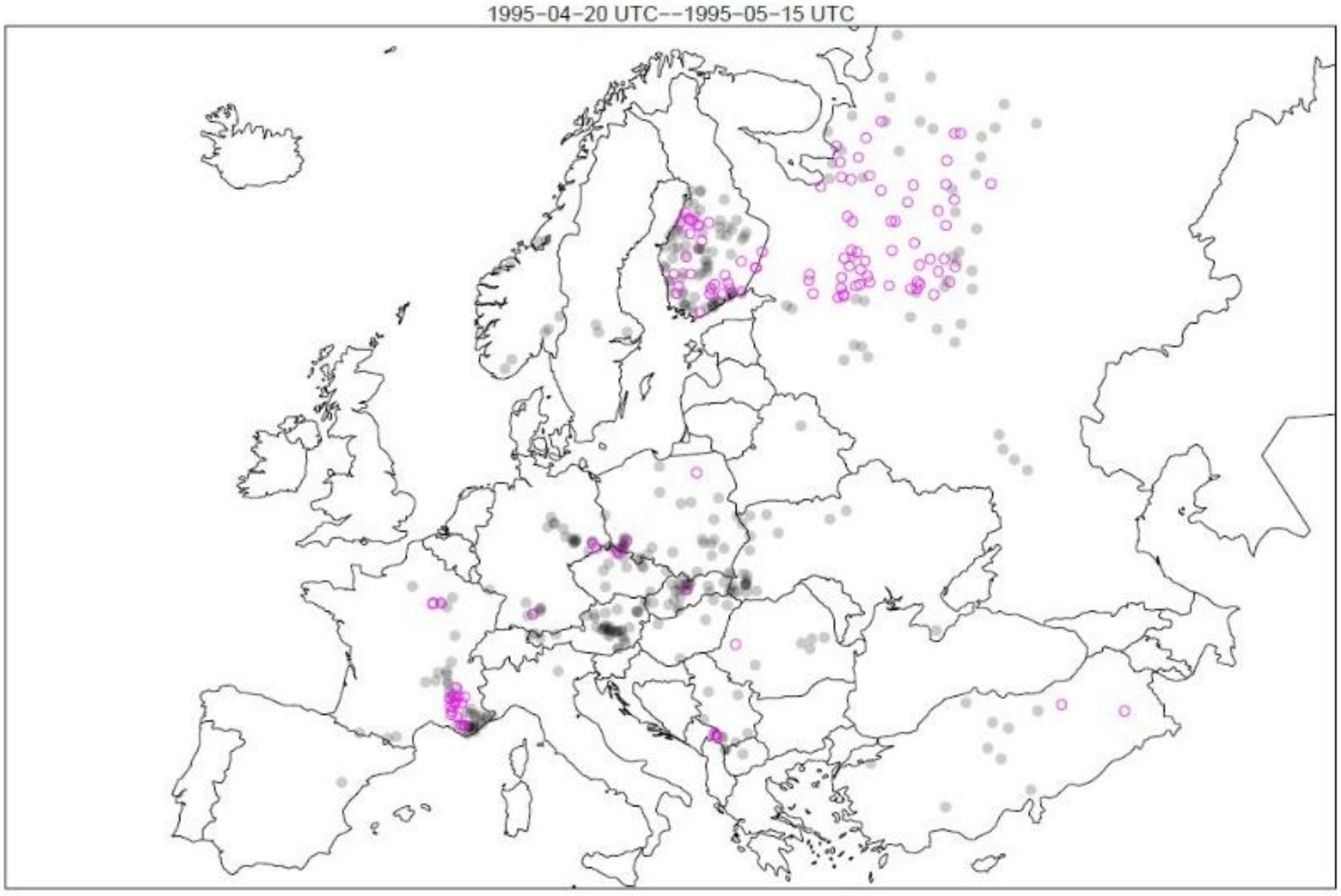

Figure 4.5.2 Floods exceeding the 5-year return period limit and/or with annual maximum reached between late-April and mid-May 1995 in Europe, based on the Pan-European Flood Database[1]

As for the preceding precipitation conditions, compared to the previous periods (i.e., March and early April alone; see Events 2, 3), when a local/regional precipitation centre developed, amongst others in the north-eastern part of the Carpathian Basin and the Carpathians, in April 2006 (see *Fig. 4.5.1*) an area with increased precipitation totals arose in the north-western Carpathians, and over Transylvania, the latter primarily affecting the catchments of the Körös and Maros. These patterns show interesting similarities to the locations of flood reports in early May 1342, along the Žitava, and in the wetlands supplied by the Körös, Berettyó and the Middle Tisza.

4.6 Event 6) Magdalena Flood: Late July-early August 1342:

Floods: Central Europe (Main, Rhine, Elbe, Danube etc.), parts of Western Europe

Cause (not directly mentioned in source): (preceding wet period latest since winter +) multiple days of torrential rains

4.6.1 Preconditions

The North Atlantic Jet Stream was positioned (annually) at an unusually low latitude (41-42°), generally suggesting that the main "action area" of the Atlantic storm tracks/westerlies in 1342 were in the Mediterranean, significantly increasing the probability of occurrence of Mediterranean cyclones and the cyclones following the Vb track.[6] In summer 1342, the Old World Drought Atlas[246] and CMIP6 hydroclimate simulations suggest dry conditions along the Atlantic coast of North America, combined with wetter than average conditions over the North Atlantic. Tree-ring based multidecadal reconstruction suggests high (spring-summer) groundwater levels for many years prior to 1342.[247] After an unusually wet and long winter with (great) floods in much of Western and Central Europe both in late winter and spring, and a prolonged rainy period in early summer, soils could be rather saturated by summer 1342. Although the Mediterranean Sea surface temperature was not necessarily higher than usual (in the north central Mediterranean rather lower, after a wet spring), for the development of the greatest Vb events in the Mediterranean the wind speed is the most important factor, more important than sea surface temperature.[248]

4.6.2 General summer preconditions and the early summer rainy period

The summer was generally cooler[249] in large part of Europe, with positive(-like) summer NAO (partly as a consequence of Arctic ice melt) and strong westerlies, particularly in the Mediterranean, also related to the dominant annual southerly position of the NAJ at 41°N in 1342[336] (partly or mostly caused by the Icelandic eruption in 1341). The strong westerlies predominantly affected the south, carrying moisture-rich air from the Atlantic Ocean to the Atlantic coast and the Mediterranean (see Fig. 6 in the paper). Warm and dry weather (blocking high) weather prevailed over Scandinavia and in south of England (see Supplementary Data 2a); the high sea surface and sea surface temperatures in summer over the Atlantic east/north-east as well as the Old World Drought Atlas hydroclimate reconstruction suggest drier than usual conditions in (spring-)summer, over Eastern Europe and South-east Europe (also the Middle East), but overall wet (spring-)summer conditions in Europe (Supplementary Data 2b).[38,246] In summer, generally warmer and drier prevailed in north-west Scotland; similar but changeable weather dominated in the south of England (Supplementary Data 2a).[250]

Early summer in West Central Europe (esp. NW-Alps) was wet; prolonged rainy weather was reported in Mainz and the "mountain areas" (possibly meaning the Alps) from at least the second half of June, potentially causing already some flooding in (late) June; however, only the multi-week rainy conditions are detectable in contemporary documentation (see Supplementary Data 2a). Although a 17th-century source explicitly mentions two distinct flooding periods in summer in West-Central Europe, one in late June and the other in late July (the latter being the Magdalena Flood), contemporary sources only speak about a prolonged

rainy period and then the “deluge” of rain in the days before the (Maria) Magdalena Flood (Supplementary Data 2a).

Caused by heavy rainfall, in summer 1342 there was flood also on the Rhône (and Durance) that reportedly arrived from the mountains (probably meaning the upper, alpine river sections). Significant (summer) flooding was also detected in the lake sedimentary profile in the Upper-Rhône alpine catchment (Supplementary Data 1b). One contemporary source generally dated the Rhône flood in Avignon to the early part of summer, another (Winterthur) mentioned a highly lethal flood (with ca. 200 victims) in connection with the new pope who was elected in May (many church officials/priests, hoping for promotion, gathered in Avignon and stayed there probably for months, meaning that the flood could happen any time from May and in summer). Additionally, a third contemporary source dated this flood to around the same time as the (Maria) Magdalena flood (which may only mean those weeks or sometime in July; Supplementary Data 1a). This dating uncertainty of the Rhône flood could be potentially also related to a prolonged flood or the overall impact of the Magdalena Flood in the author’s memory (i.e., the author organised his notes/memories, perhaps years after the events). There is also a possibility that the Rhône flood was related to the larger-scale flood event mentioned by the 17th century source (and other non-contemporary sources). As the information about the probable late June flood was only preserved in much later sources, and the summer dating of the Rhône flood is rather imprecise, we did not account with the June rainfall and flood references as a separate European event.

In conclusion, latest from the second half of June, in West-Central Europe (probably centred to the alpine area) there was a prolonged rainy period of weeks in early summer, with perhaps occasional flooding already starting in (late?) June in and around the western/north-western side of the Alps. The extent of this rainy period and potential flooding is unknown, and perhaps it was discussed together with the Magdalena Flood by contemporary and other medieval sources.

4.6.3 Weather of July 1342 and the (Maria) Magdalena Flood

III.1. Incessant rains (8-day rainfall) in Central Europe: While the weeks before the Magdalena Flood were already rainy north/north-west to the Alps, German sources, especially in the north, mentioned the warm, dry summer conditions and the surprise effect of the Magdalena Flood, suggesting a somewhat opposite weather pattern in the north before the Magdalena Flood (Supplementary Data 2a). The torrential rains/heavy rainfall, lasting for eight days according to contemporary sources (estimated to a 1000 to 10,000-year return period event),[5] immediately preceded and was the main cause of the Magdalena Flood, starting from 28 July (GC). Sources do not provide an exact location or area of the rainfall or the extension of its core area; when mentioned, most of them suggest “in the mountains/hills”. The only area where the location of heavy rainfall was also mentioned was in Bavaria, along the Altmühl. Thus, it is not clearly defined how extensive was the area affected by the heaviest (i.e. 1000-10,000-year) rainfall, and which area was “only” affected by less intensive (but still intensive enough to cause great flood) rains.

Thus, based on the available evidence it is also possible that the area of maximum rainfall restricted to relatively smaller areas (i.e., Main catchment and part of Bavaria and the of the Ore Mountains), but generally a perhaps less intensive (but still important) prolonged rainfall, also capable of causing even great floods, could affect much larger areas. In the areas

already affected by the rainy weather from June (and perhaps to some extent already in flood), even a not-so-extraordinary rainfall period of some days could cause significant flooding.

No additional weather information is available in East-Central and Southern Europe; however, the daily weather reports in South-east England, presented by William Merle,[158] provides some interesting details particularly regarding wind conditions prior to and during the Magdalena Flood. Accordingly, in the south-eastern part of England (observed at the latitude ca. 52°N), on 10-13 July (in Julian Calendar; 18-21 in GC) there was strong westerly wind that brought only occasional, very light precipitation. After 3 days with varying precipitation (sometimes light, sometimes more rain), the heavy rain arrived on 17 (GC: 25) July brought by strong south-western wind. This was followed by two days of rain, with thunders on 19 (GC: 27) July. The days after were only with occasional light rain.[158] There is a chance that these changes also reached and affected West-Central Europe some days later and contributed to the development of the incessant rains.

III.2. Spatio-temporal extent and intensity distribution of the Magdalena Flood: Based on the contemporary reports, the most intensive flooding developed on the Main tributaries (both northern and southern tributaries of the river were mentioned), perhaps also in (some parts of) the Neckar basin, and also the western tributaries of the Elbe were heavily hit by flood. While the heavy rainfall and floods extended to the south, into the Franconian Alps and the Upper-Danube catchment in Bavaria, no flood report is available from Bohemia (which does not exclude the possibility of some moderate flooding). Only the fact of flooding but no unambiguous magnitude information is available regarding major rivers such as the Elbe, the (Upper) Danube (Supplementary Data 1a). In some other cases (e.g. Lahn), the description is not clear enough to decide whether the flood was the result of local flooding (on the specific river) or, being a Rhine tributary, the water level increase was significantly influenced by the great flood of the Rhine.

The earliest flood reports come from the west, from 28 July (in GC) on the Leine, Weser, Fulda, Werra, Main), followed by the Leina, Mulde, Gera, Regnitz, Oker, Neckar, Danube, Unstrut, Saale, Werra and Elbe on 29 July, and the flood reports extend towards the east in the next two days (with further flood references also in the west; Supplementary Data 1a). Flood damages were recorded 4-5 days later in the Rhine delta compared to the Middle-Rhine: this approximately fits the usual flood travelling time on the Rhine. The flood locations appear relatively mixed in time, so that it is difficult to trace any clear change in spatial pattern or the potential 'move' of, for example, a possible low-pressure system.

Based on the original source evidence, floods (of varying intensity and duration) affected an unusually large area: the greatest damages and flood levels were documented along the Main River and its tributaries (both sides), but both the eastern and the western parts of the Main catchment were affected by the floods. The other area of heavy precipitation was situated east to the Main catchment, and affected the southwestern tributaries of the Elbe and the western part of the Bavarian upper Danube catchment. In the contemporary documentation, there is a couple of days delay between the heavy rain and first flood reports, spanning between ca. 20 July to 25 July (Supplementary Data 1, 2). The spatial extension of the areas, affected by the Magdalena Flood, makes it possible that a low-pressure system or systems reached over the Main catchment and parts of (today's) Bavaria, and partly extended to the north-east, to the north-western side of the Ore Mountains.

While in some cases there is clear evidence on the extreme magnitude of flooding on specific rivers (especially the Main and Upper-Main catchment and the south-western tributaries of the Elbe), in other cases only the great flood certain rivers were mentioned without specification, and indirect circumstances may suggest that the flood, even if great in magnitude,

was perhaps not record-breaking. Based on a Braunschweig source and also the short and less detailed flood mentions towards the east that specifically refer to the Magdalena Flood with exact dating, it seems that the largest magnitude flooding primarily hit the more westerly rivers (i.e., west to Braunschweig), while the magnitude of flooding was less severe on the large rivers of the east. For example, the Elbe and the Danube were also in flood, but – unlike in February 1342 when there were clearly outstanding floods on these two rivers – regarding magnitude the sources generally do not elaborate further (see Supplementary Data 1a). The "deluge" mention in Bavaria (along the Altmühl, also close to the Danube) perhaps refers more to the magnitude of the rainfall (i.e., it was a deluge, just like exactly a year later) than the flood itself. Although it is clear that there were heavy floods on some of the major western tributaries of the Elbe, the Czech chroniclers mention no flood (on the Vltava or Labe) in this summer in Bohemia, which – together with the surprisingly short flood mentions of the Elbe itself – indirectly suggests that the upper section of the Elbe and its key tributary was probable not flooding (heavily).

In comparison with the spatial distribution of the area with the outstanding heavy precipitation, provided by Herget and his colleagues[7] there is an important difference: We locate the area of heavy precipitation more to the south, centred around and Main catchment, the areas south to Main catchment (but not covering/reaching the Northern Alps) and to the middle hills north-west to the Czech basin. Although flood reports about great and extraordinary floods are also available from the areas north of the Main catchment, these floods either could come from the north and therefore local great precipitation was not necessary (and no contemporary report suggests there was any) or the descriptions about the floods are (spatially) general and/or refer to the (outstanding) floods of other areas (e.g., Braunschweig).

4.6.4 Potential modern parallel(s)

Compared to the major Central European flood events in 1960-2020 that affected most of the areas known to be hit by the Magdalena Flood, the May-June 2013 European floods may show the most (seasonal, spatial extension) similarities, with the difference that the core area, just like in summer 2002 and 2006, was located much more to the east in 2013 (or in 2002, 2006)[251] and was notably smaller compared to July-August 1342. Similar to the 2013 floods,[252] the Magdalena flood in late July 1342 was a trans-basin flood; the heavy rainfall (primarily caused by a major Vb event) affected the Upper-Danube catchment, Bohemia and the Ore Mountains roughly in the same time, and although rather moderate, there was a notable (but not great) flood on the River Main, too. Typical overlapping characteristics between the July 1342, 2002, 2006 and 2013 floods were the particularly wet initial catchment conditions, and also the following very heavy precipitation in the days immediately preceding flood development.

Moreover, in 2013 there were two notable floods on the Rhône (in May and in early July). In general, in the 1960-2020 period the more important floods mostly took place on the Rhône in June (e.g., 1969, 1970, 1974, 1985, 1987, 1991, 1995, 2003, 2010, 2012, 2016: Pan European Flood Database)[1]; but only in a few cases (e.g., 2002, 2006 and 2013) the Rhône floods shortly preceded or coincided with important Central European floods. Although the core (heavy-rainfall) area of these floods was relatively small, the area that was affected by this flood event within Central Europe could be rather large: Therefore, in the (hypothetic) reconstruction we also included the Danube area in East Central Europe, because the Danube had to be in flood from its upper through the middle to the lower sections.

More problematic is the situation when we try to find particular parallels to large-scale Central European flooding with flood maxima concentrated to the Main catchment (and the

hilly area east, north-east to the Main), as the largest Main floods usually happen in winter (rarely early spring or late autumn), while only smaller floods occur occasionally in summer. As for great Main floods, the flood happened in 1925 or the one in 1970 show some, notable spatial similarities; however, both happened in winter not in July and, apart from the rain, snow(melt) played a rather significant part[18] (also based on the 500y European Historical Flood Database),[11] which means a basic difference from the flood generation of the July 1342 floods.

Although with a different (smaller) spatial extension, but similarly with a core area of heavy rainfall and flood reaching 10,000-year return period, the July 2021 flood – together with the floods of 2002, 2006 and 2013 – may provide further useful information, as there are some, potentially important details that show interesting parallels to the July 1342 event and can also help in the better understanding of the meteorological patterns leading to the outstanding floods in July 1342. The 2021 flood event, just like most of the other modern parallels (e.g., the 2002 and 2013 floods) was the consequence of a combination of patterns, and not only a "simple" Westlage or Vb event.[253]

Any modern comparison is further complicated by the fact that the spatial distribution and spatial extent of heavy precipitation documented in July 1342 flood, does not show more significant similarities to the top eight (spatial) precipitation patterns that were observed between 1951 and 2021 in Germany,[254] even if the overall spatial extent (but with different locations) of extreme precipitation totals during some of these events were close to that of the Magdalena Flood.

4.6.5 Flood generation of the July 1342 (Maria) Magdalena Flood

Previous scientific claims on flood generation: While all studies agree that the extraordinary flooding was the result of convective event, regarding the causative mechanisms there are two, partly contrasting views:

1) Part of the preceding research suggest that the Magdalena Flood was caused by a cyclone following the Vb path.[118,255,256] The opinion is based on mixed contemporary and non-contemporary data, suggesting a larger spatial distribution of floods that also affected the northern part of the Alps, and it is also based on the theory of a temporarily very concentrated rainfall-related mass soil erosion and also on some of the weather and flood descriptions (i.e. multiday incessant rains preceding the floods, high-intensity flash-flooding, the event was largely unexpected based on prevailing weather conditions, especially towards the north).

2) Another investigation, based on a selection of contemporary and non-contemporary sources,[7] suggests that the (Maria) Magdalena Flood was the result of a major convective event that hit a restricted area, mainly located north of the Main River, and it was not Vb, but some kind of convective event. The authors presented and analysed summer (August) weather patterns cases resulting heavy precipitation in the 20th century, in the area affected by the July 1342 floods in Germany: They found that the 14 studied cases (between 1936 and 1992) were related to "West, cyclonic", "Southwest, cyclonic" and "Western European trough" circulation patterns.[7] However, their reconstruction provides a partly different spatial distribution of floods and spatial reconstruction of the potential core areas primarily affected by the outstanding rainfall compared to our current database, and the heavy precipitation in their reconstruction was mostly restricted to large part of the Main catchment and to some areas located somewhat north to the Main catchment. According to this approach, the area of flood generation concentrated to a roughly rectangular area between Nürnberg, Frankfurt/Limburg, South-

Braunschweig and extended to the Zwickau area in the east. The denial of a Vb event was also reasoned by the fact that no flood mention is available in summer 1342 in Bohemia.

Despite the critical survey and detailed analysis of the latter investigations regarding the potential weather background of the July 1342 floods, a common characteristic in both approaches is that the analysis of floods, climate and weather was not preceded by systematic new, large-scale historical source and data collection and (historical) critical source evaluation, even if in the second approach some source selection (primarily geography/hydroclimate-based) criteria were applied. In both cases the events were discussed in a local/regional context, predominantly covering the present-day southern and south-central parts of Germany. Both opinions agree that the incessant rains were most probably the result of major convective event(s).

Flood generation based on contemporary sources and modern parallels: In our current analysis we conducted a systematic survey, revisited and critically evaluated all previously applied sources that referred to the Magdalena Flood. Moreover, we carried out an additional survey of published contemporary sources and also made pilot surveys and found unpublished original, contemporary sources – primarily referring to weather events and partly to floods – never applied before in the analysis of the July 1342 flood event. Thus, we developed a systematic dataset based on contemporary sources, and only in one-one cases we additionally (to statements already available in contemporary source) applied near-contemporary (i.e., late 14th–early 15th-century) source evidence (with clearly marking them and giving less weight in the analysis). Once we only consider the contemporary (and connected near-contemporary) medieval sources, and also separate the evidence that refer to the (heavy rainfall/incessant rains and then) flood of specific areas and to those that more generally refer to the flood with only providing a list of affected rivers and/or areas (in which case the magnitude of flooding is not known), we see a broad flooding concentrated to the (south-)central part of today’s Germany that started within 5-6 days.

According to our findings, the hilly areas from the Franconian Heights and Franconian Alps in the west (and maybe beyond, in the Meuse catchment) through the Thuringian Forest, Thuringian Highlands, to the northern, north-western part of the (Western) Saxon Ore Mountains were probably hit by the incessant rains and floods. Although the great flood in the Neckar Valley and the deluge mention near the Altmühl and the Upper Danube could as well be (but not necessarily) related to the northern Alps, no direct information is available in contemporary sources that the heavy rainfall and the following floods would have (heavily) affected the northern alpine area. Here, we agree with Herget and his colleagues[7] suggesting that no historical report directly suggest significant amount of precipitation directly along the Middle-Rhine (i.e., Cologne), and that the (ca. 100-year return-period) Middle-Rhine flood was primarily caused by the extraordinary (ca. 1000-year return-period) flood of the Main, meaning that the upper Rhine sections (i.e., Upper-Rhine) were not in (significant) flood in July 1342. Actually, the contemporary report of the *Gesta Baldewini de Luczenburch* in Trier[257] concerning the flood of the Rhine has special importance: the author emphasised that the great flood of the Rhine in Cologne was not coming from the Upper Rhine (i.e., from Basel or Strasbourg), but it was (mainly) the consequence of the heavy flooding on the Main.

This is, to some extent, a difference compared to most of the previous opinions that put an emphasis on the alpine areas with suggesting a Vb origin of the Magdalena Flood (i.e., investigation group 1 above). It has to be mentioned, however, that based on the available contemporary sources we cannot explicitly state either that the most northerly parts of the alpine foothills remained unaffected by rainfall and flooding. However, if there was a flooding at all in that area that either stayed unrecorded (i.e., as in Bohemia) or it was not mentioned in sources because its magnitude (and destruction level) did not reach the level worth recording. The

floods were, according to our current database, spatially more extensive to the west, south-west and east, south-east than estimated by Herget and his colleagues[7] (i.e., investigation group 2 – see above). Great or outstanding flooding also affected the southern tributaries of the Main, the Neckar and the Regnitz-Altmühl-(Upper-)Danube catchments. There was an at least notable flood on the Meuse, too (but sources do not elaborate on the magnitude or the magnitude information refer to the Delta area and not only for the river itself; see Supplementary Data 1a).

When mentioned, contemporary sources suggest that the origin of the floods was the extreme rainfall in the "hills" or "mountains" (i.e., the flood came from far upstream) and sometimes also mentioned as if the water would have "come from the ground" (excess groundwater?) – probably reflecting on the very wet antecedent conditions and that many of the areas hit by (sudden) extreme flooding had not experienced the excessive rainfall in the days/week(s) before. Thus, as in July 1342 sources in most cases do not clearly define the core area of the heaviest rainfall, it is only possible to deduce based on the extension of river (upper) catchments heavily affected by flooding. However, high groundwater and soil moisture levels can cause (great) flood even after relatively modest rains,[258] which means that the area affected by the heaviest incessant rains were not necessarily so extensive as all the upper catchments of the rivers hit by great or outstanding floods. Similar conclusions were drawn, for example, concerning the July 2021 flood by Mohr and his colleagues;[252] additionally, infrastructural damages and the spatial scale of July 2021 destruction were much lower and more localised than the spatially more extensive May-June 2013 floods.[254]

Magdalena and the Vb: why it is a possibility (as a contributing pattern) and why the only-Vb flood generation hypothesis does not work: Based on the areas hit by the incessant rains in mid-/late July, and also based on the prolonged (weeks of) rainy period in (late) June (reported e.g., in Trier), it is difficult to suggest one type of (either e.g. Westlage or Vb) climatic pattern as an ultimate cause of the (Maria) Magdalena Flood. The prevailing very southerly position of the North Atlantic Jet Stream in this year generally supported the development of high intensity cyclones in the Mediterranean, and this statement likely also implies for the cyclones of Mediterranean origin following the Vb path. Nevertheless, major River Main floods usually do not happen in summer, and are not caused by a Vb event. Moreover, the unusually large spatial extension of the areas affected by incessant rains do not support the simple (usually spatially restricted rainfall area) Vb-origin either. The fact that no flood reference is available in Bohemia also weakens the (simple) Vb-related argumentation; however, the lack of flood report does not necessarily mean that there was no any kind of flooding in Bohemia – for example, if the flood was not outstanding in magnitude (i.e., did not cause much destruction; especially after the catastrophic flood of February 1342) it was not necessarily mentioned in chronicles.

On the other hand, the fact that both (parts of) Bavaria (i.e., at least part of the Upper-Danube catchment), the Neckar and the south-western tributaries of the Elbe were heavily affected by the incessant rains roughly in the same time in July – together with the very southerly position of the North Atlantic Jet Stream in this year – may enhance the possibility that, at least partly, Mediterranean cyclone activity could as well contributed to the development of the extreme rainfall and floods in Central Europe.

In general, a clear characteristic of a Vb event is that the heavy precipitation events occur over the Alps and its northern foothills, while there is typically no precipitation in the southern part of the alpine area at that time.[259] In this sense, Johann von Winterthur's remark on the fact that in the time of the Magdalena Flood there was no flood in Lombardy (i.e., the other, southern side of the Alps; Supplementary Data 2a) is also interesting as it could indirectly reflect on flooding in the northern side of the Alps – however, this rather indirect information (or the general rainfall and flood references in the Neckar and Upper-Danube catchments)

cannot be used as a supportive proof for the northern alpine flooding, and in July 1342 no contemporary evidence directly suggests (or denies) large-scale rainfall and flood activity in the (northern part of the) Alps.

Magdalena, modern flood parallels and potential (other) weather patterns: Another possibility, more likely considering the River Main catchment, would be the causative hypothesis that relates the weather conditions causing the Magdalena Flood to western or north-western low pressure system activity, namely that the rainfall originated from western, north- (or south-)western moist air masses, directly arriving from the Atlantic Ocean, the North- and/or the Baltic Sea. In any of the suggested cases, to the development of such a strong storm/cyclone event, rather strong pressure gradients and low-pressure conditions (and the presence of blocking events) are a necessary precondition.

During summer 1342, dry and warm weather, suggesting high surface pressure conditions, generally prevailed over Scandinavia (and before and during Magdalena also in North-Germany), North-west Scotland and the south of England, and most probably also in large part of the Eastern Europe. However, as we could see before, the weeks and days before the Central Europe July 1342 flood event were on specific days with heavy rains, very strong western and then south-western winds in South-east England (Supplementary Data 2a,b). These conditions, together with the higher-than-usual sea surface temperature (and sea level pressure) conditions over the North-Atlantic (for a detailed 1342 hemispheric/global overview, see Supplementary Information 5.2) may raise the possibility that in one-two weeks before the Magdalena Flood there was a strong westerly (and then south-westerly) flow in the near-surface area that could carry moist air masses and also reached West-Central Europe.

Just like in 2013 or 2021, antecedent wetness conditions can be a key factor in flood generation, as it can determine whether the rainfall event results in major flooding (or not).[251,252] Particularly in connection with the Magdalena Flood, contemporary authors emphasised the importance of the water that “broke out of the ground”, presumably reflecting on the high soil moisture and groundwater levels (this idea is also supported by tree-ring based reconstruction).[247] As we could see before, in (late) winter and spring and there was at least one more rainy period (maybe with some flooding) that lasted for weeks in late June–early July. Thus, the soils of the areas affected by the July 1342 rainfalls could be already highly saturated with very low infiltration capacity immediately before and during the Flood.

Taking into consideration the changing wind directions in South-east England (maybe in connection with the southward lengthening/expansion of the Rossby waves), and the unusual spatial extension of the area hit by incessant rains, there is a possibility that the Magdalena Flood, like some of the most destructive Central European floods of the 20th-21st century (as, for example, the summer floods in 2002, 2013 and 2021), was caused by a more complex weather pattern and course/coincidence of events, influenced by multiple slow-moving low (and high) pressure systems.[232,251] Although from the period 1960-2020 period we chose 2013 as an event with the overall most similarities (spatial extension, magnitude, suddenness), it has to be emphasised that the Magdalena Flood, its location and at least partly also flood generation, show notable differences from any of the relatively similar extreme flood events. Moreover, other highly destructive outstanding floods such as the July 2021 flood also show some thought-provoking potential parallels that should be considered.

The disastrous July 2021 Ahr Valley flood can also provide a potentially useful parallel: In July 2021, the combination of antecedent low infiltration capacity of soils (combined with the land use and weakened prevention measures), blocking in North-east Europe, slow moving Atlantic air masses (Bernd storm/cyclone), enriched by moisture-rich Baltic and Mediterranean air masses, combined with the impact of Dana anticyclone/blocking west to the Bernd only days

before the incessant rains and the orography together were responsible for the flood disaster that could be even significantly larger (both in magnitude and spatial extent) if occurred only a couple of ten km-s to the east.[260,261]

Another group of possibilities is related to, for example, the 2002 flood event: As we could see, in the week(s) preceding the Magdalena flood, for days strong westerly, then some days later strong south-westerly winds occurred. If these winds continued towards Europe, there is even a possibility that in July 1342 a *Westlage* type of cyclone was followed by a *Südwestlage* (based on the rainfall pattern in the eastern part of the area most hit by the incessant rains) with only a few days difference. In 2002, for example, the flood disaster in Central Europe was caused by two cyclones arriving with only some days difference, following almost the same Vb track.[262]

The known spatial extension of great and outstanding floods in July 1342, does not show direct similarities to any of the *Westlage* or *Südwestlage* (including Vb) flood generation patterns that occurred in the period 1960-2020. However, if the July 1342 could also be the combination of multiple patterns including more than one slow moving (south-)westerly cyclone/storm systems, with potential water intake both from the Mediterranean and the (North and the) Baltic Sea as happened in July 2021, it would be possible to explain the most important elements of the rainfall-related weather patterns and large spatial extension of floods that hit West-Central Europe. Based on the currently available flood and weather information, it is not possible to suggest a clear and unambiguous mechanism behind the outstanding magnitude and spatial extent of the Magdalena Flood. However, like the Dana anticyclone in 2021, in July 1342 a blocking pattern/anticyclone could well be responsible for the very slow movement or stationarity of the low-pressure system that resulted in the Magdalena Flood.[252,262]

We also have to consider the important direct influence of additional (short-term) high pressure centres: for example, in late May 2013, the blocking high called Sabine over the sea west of the UK and France split the jet stream, while in July 2021 the Dana anticyclone blocked the westward movement of the low-pressure system over Central Europe, and by doing so became also responsible for the flood disasters: the same could potentially happen in July 1342, too. Interestingly, wind directions of July 2021 in South-east England also show similarities: just like in July 1342, prevailing wind directions were west(-southwest) and then south-west in Hertford (SW-England) days before the start of the incessant rains and flooding in West-Central Europe. Although in early July most days were with little or no precipitation in 2021 (which shows similarities to July 1342) in Hertford, no strong precipitation was recorded in any of these days in early July 2021,[263] which is in contrast to conditions in July 1342 when the days with very strong western and later south-western winds arrived with heavy (daily) precipitation.[158]

Nonetheless, while the July 2021 catastrophic flood (apart from the antecedent conditions) was the result of a less-then-one day rainfall with extreme rainfall totals in the Ahr Valley (more than double of the average monthly total, within 15 hours) and a couple of days intensive (but less intense than in the Ahr catchment) rainfall in the adjacent areas (e.g. Belgium, Luxembourg), July 1342 sources (similar to the 2002, 2006 and 2013 cases) speak about days of incessant rains and then the following flooding in rapid succession. As for the antecedent soil moisture conditions, both in July 2021 and 1342 an intensive (and/or prolonged) rainy period were reported ca. 3 weeks before the catastrophic rainfall and flood events, "*supporting high surface runoff through saturation excess*".[252] Another peculiarity of the July 2021 is that even on the rivers in the adjacent areas – thus, in a rather extensive area including W-Germany, Belgium and Luxemburg – flood magnitudes often reached or exceeded the 100-year return period levels. A further similarity of all these high-intensity floods including the July 1342 is the high velocity and hydromorphological activity of the flood, or the high erosion

rates (e.g., Frankfurt: see Supplementary Data 2b, 3). Similar to July 2021 (or to 2002 or 2013), hillslope denudation and large-scale landslides – high volume of sediments and debris increased water level and the level of destruction (compared to the possible discharge magnitude).

In general, the combination of factors, nonlinearity, interdependence/feedbacks and non-stationarity can as well influence a pattern resulting in a rainfall event and following catastrophic flood of unprecedented/surprise extension, timing and magnitude. Event perception can also be influenced by cognitive bias.[258] In case of wetter catchment conditions, even smaller precipitation can lead to floods, and flood clustering is stronger in case of less severe floods within a shorter period of time, which might be explained by antecedent wet catchment conditions, catchment memory effect and short- and long-term climate variability.[251] Based on model simulations,[261] in July 2021 a mere 15-25 km more easterly position of the precipitation field would have resulted in an up to 160% increase in peak flows of the Ahr tributaries.

A further thought-provoking point in the July 2021 flood (in comparison with the July 1342 events) is that, while simulating the potential rainfall conditions and the magnitude changes of flooding under future global warming scenarios,[254] suggested that both the magnitude and spatial extension of the Ahr flood will be significantly more elevated in the future, and that the hydrologic catchment response in a warmer climate suggest a non-linear increase in peak flows. Using this example as a parallel, the Magdalena Flood and the 1342-1343 flood events can also act as a potential ”worst case” for potential future scenarios.

4.7 Event 7) Late August (–early September) 1342

Floods: North-Central Italy (Bologna – most probably the River Reno; Florence – River Arno); most probably also River Drava (E-Alps/SW-Carpathian Basin)

Cause: heavy rainfall (reported in Italy)

In late August 1342, in an outstanding flood was reported in Bologna (Reno), North-Central Italy. Similarly in August, the Arno also had a flood of outstanding magnitude – the latter flood possibly occurred around the same time as the Bologna flood event (Supplementary Data 1a). Although an inundation at the River Drava in the Southwest-Carpathian Basin (see Supplementary Data 1) was only reported in (mid-/)late September, the great late August 1342 flood on the Reno, presumably, happened around or was shortly followed by the flood of the River Drava/Drau. This flood likely remained as an inundation for weeks and this was the inundation in the Drava floodplain that was reported some weeks later, in September.[264]

The great late-summer flood event(s) in Italy, reportedly caused by heavy rains, the strong winds in Southeast-Spain (Algeciras, Gibraltar Bay) and the Drava flood, originated from South-Tyrol, mark again a rather special case primarily because of the very unusual/rare summer timing of these great and outstanding floods (probability of flood occurrence in North-Central Italy is the lowest in summer), while July-August floods do happen sometimes on the Drava (see Supplementary Data 1a, 2a). The case shows similarities to modern autumn (October, November) floods that usually affect the Mediterranean and specific parts of Central Europe (e.g., SW-Carpathian Basin). Receiving its floodwaters primarily from snowmelt and then rainfall in the Alps, spring and early summer floods are more common on the River Drava,

and there is also an autumn secondary maximum (which become primary maximum in some years), but August-September is usually a low flood-risk period.[265] And, finally, based on the Pan European Flood Database,[1] great Arno and Reno floods relatively rarely coincide with greater Drava floods.

Based on the Pan European Flood Database, the closest similar event in the 1960-2020 period is the (early and late) October floods in 1993: there were floods in East-France (e.g., Rhône), East-Switzerland as well as in North-Central Italy (including the Arno and Reno, see *Fig. 4.7.1a*) and on the Drava (where flood reached and stayed on the lower Drava also weeks after: see *Fig. 4.7.1b*), even if the magnitude of these events was probably smaller than in August 1342. The flood was caused by the succession of Mediterranean thunderstorms, followed by heavy rainfall in the mountain areas.[266,267]

A considerable difference between the conditions immediately preceding the August 1342 and the October 1993 floods is that in 1993 a couple of weeks before the Arno and Reno floods there were great devastating floods in North-/Northwest-Italy, caused by continuous heavy rains as a result of a cut-off low (with a cyclogenesis around the Bay of Lion.[268] Such a flood would have roughly coincided with the Magdalena Flood in the German areas, but Johann von Winterthur explicitly stated that no flood occurred in Lombardy (i.e. N-Italy) around that time (see Supplementary Data 2a).

Another possible (but somewhat less) matching case could be the famous 1966 flood: in this year in November there was a catastrophic flood on the Arno and the Reno.[268] However, despite the fact that the source area of the river in the Eastern Alps was heavily affected by rainfall also in November,[269] no great flood reached the lower Drava sections in November[1] (see also Supplementary Data 4), although there were great floods on the Drava in other parts of the same year (greatest in August).

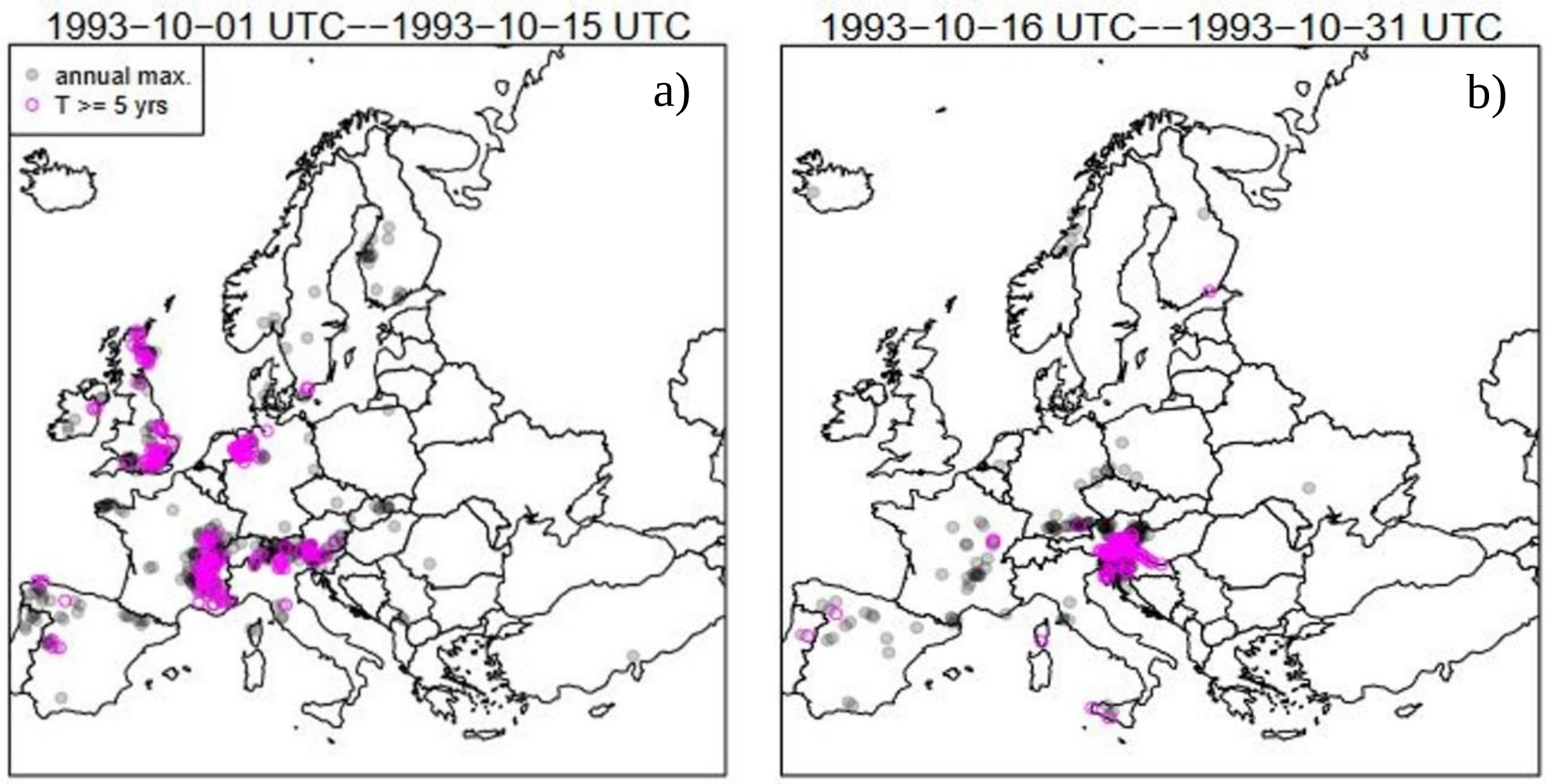

Figure 4.7.1 Floods exceeding the 5-year return period limit (magenta circles) and/or with annual maximum (grey dots) in the first (a) and second (b) half of October 1993 in Europe, based on the Pan-European Flood Database[1]

Other, late summer–early autumn Arno floods (e.g. 1964, 1973, 1976, 1990) that could potentially serve as a modern parallel, as well as the November 1987 floods in Italy, also passed

without a more significant Drava flood.[1,11] Further partial similarities are detectable in September 2014 when (South-)Italy, Southwest-Hungary, Southeast-Austria, Serbia, Croatia, Bosnia and Herzegovina were hit by floods, and August 1965 and July(-August) 1972,[1, 270,271] when major floods occurred on the Drava; however, in none of these cases there were considerable magnitude floods in Italy. Lately, in May 2023 there were floods both in North-Central Italy and on the River Drava: none of these floods represented the annual flood maximum, and the Arno was not even in flood and the Reno only reached minor flood levels.[272] Thus, also compared to the last 60-65 years, the August 1342 floods represent a rare (but not unheard of) case.

4.8 Event 8) (Mid-)October 1342

Floods: N-/N-Central Italy (Po, Serchio), Gibraltar Bay/Algeciras rainflood, Rio de la Miel (S-/SE-Spain)

Cause: prolonged heavy rainfall (documented in both cases)

In mid-October 1342 around the same days, great destructive floods were reported in North- and Central Italy (Lucca, Piacenza: Po, Trebbio and Serchio) and in Southwest-Spain on the Rio de la Miel as well as a rainflood/flashflood in the Algeciras area (Gibraltar Bay), caused by prolonged heavy rains. In South-/Southeast-Spain the event was preceded by weeks of heavy rains, and the flood resulted in prolonged extensive inundations lasting for months. Moreover, sometime in 1342 an outstanding (roughly centennial-scale) flood also happened on the upper sections of the Ebro River in Tudela (Supplementary Data 1, 2).

Based on the Pan-European Flood Database, in the period 1960-2020,[1] there are no flood cases when great floods happened in October practically on the same, previous or following days in North- and Central-Italy and South-, Southeast-Spain. However, it is more frequent in winter or at the beginning of spring, even if usually there is a couple of weeks difference between the floods of the two regions. A case when in South-Spain and North-/North-Central Italy simultaneous flooding was observed in October occurred in 1960: Floods caused by heavy rainfall affected North- and partly Central Italy, and around the same time floods occurred mainly in North-/Northeast- and in a lesser extent in South-/Southwest-Spain. In 1960, heavy rains hit the Algeciras area related to persistent low (sea level) pressure conditions in the last week of October.[273] Similar to the case on South-/Southeast-Spain in 1342, in 1960 most of October was already rich in precipitation and floods affected large part of West-Central and Western/Southwestern Europe (see *Fig. 4.8.1*); however, the October 1342 floods were notably larger in magnitude than the 1960 floods. Other events showing similarities are, for example, the floods in early/mid-October 1993 or (October-)November 2019 when floods (caused by heavy rainfall) took place in East-Spain, North-Italy, South-France, Northeast-Hungary and Southeast-Slovakia.[274] A common characteristics of these 20th-21st-century October (November) floods in South-/Southeast-Spain that they were rarely preceded by such a long, wet period (i.e., several days, weeks), mentioned in 1342 in the area of Algeciras.

On the basis of the synoptic analysis of the (destructive) Spanish Mediterranean floods since 1960,[274] the flood and weather information available in contemporary sources and the reconstructed large-scale climatic patterns available from paleoclimate archives related to 1342, the general synoptic patterns of October 1342 show striking similarities to the type defined by

Gil-Guirado and colleagues,[275] with a peak of occurrence in October when floods mainly hit Northeast- and South-Spain. The prevailing high-pressure conditions over Greenland to (S-)Scandinavia and low-pressure conditions in the Mediterranean in 1342, combined with a relatively rainy September and early October 1342 in Hertfordshire (SE-England; just a couple of days before the Spanish and Italian floods), the prolonged great rains in September and early October in (SE-)Spain with finally the floods in (mid-)October (Supplementary Data 2a,b). This also means that, the October 1342 great and extraordinary floods, although more outstanding than usual, perhaps represent a typical timing and flood (generation) pattern in the Mediterranean. Although based on the contemporary source (urban accounts) only annual dating is possible, based on the historical local/regional environmental/climatic conditions (and also the above-described flood occurrence probability analysis), there is a possibility that the outstanding 1342 flood on the upper section of the Rivers Ebro and Queiles in Tudela (see Supplementary Data 1) also occurred sometime in October.

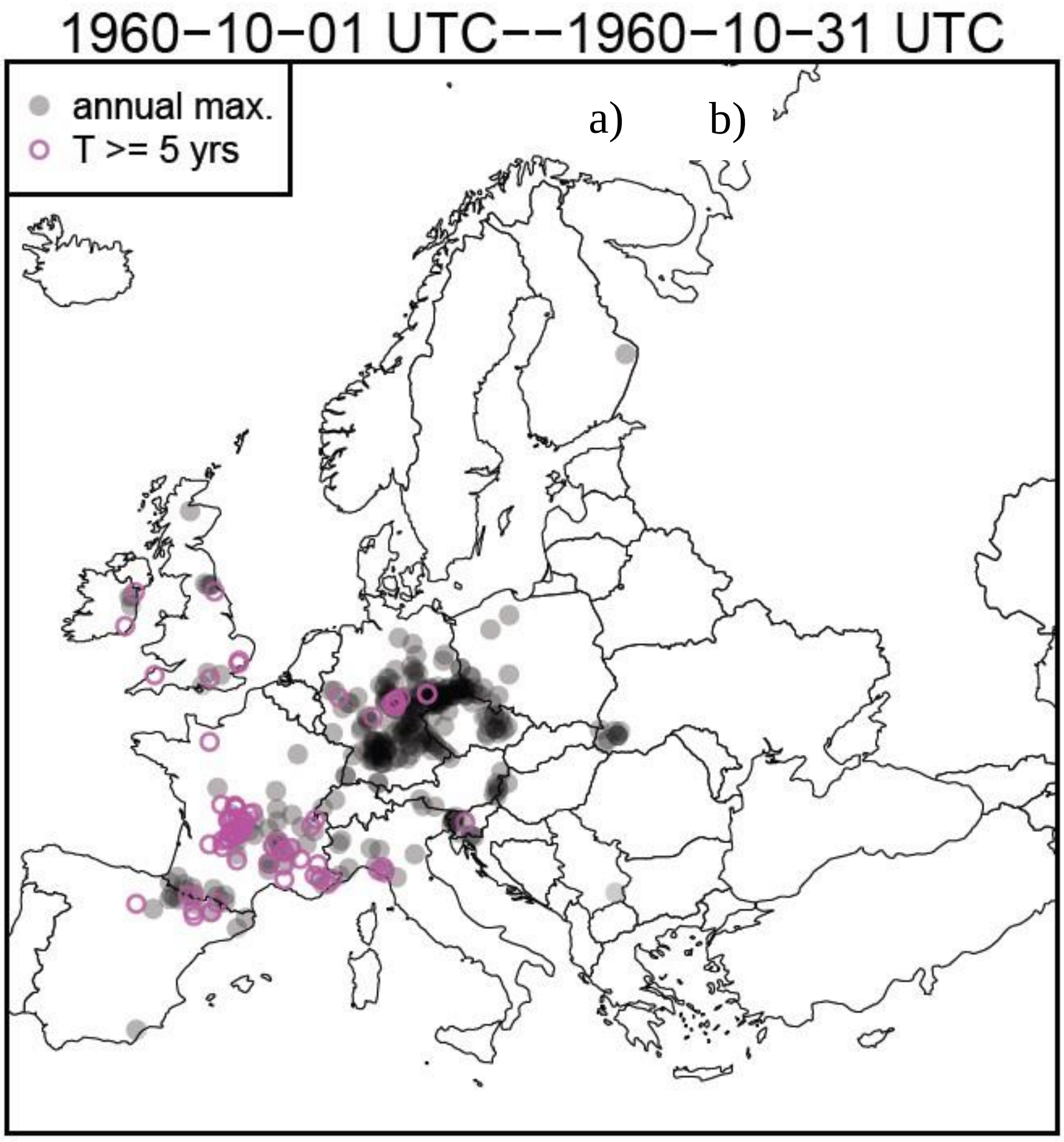

Figure 4.8.1 Floods exceeding the 5-year return period limit (magenta circles) and/or with annual maximum (grey dots) reached in October 1960 in Europe, based on the Pan-European Flood Database[1]

Although it is beyond the time frame of the comparative study period (1960-2020), the recent Mediterranean storm-related floods in October 2024 (or the one that happened in 1957, very similar to the 2024 case) also show some thought-provoking parallels to the October 1342 events: The heavy-rainfall related floods in Central Italy (e.g., Siena, Bologna) occurred on 17, 19-20 October 2024, not far from the location of the 10, 13 October 1342 floods (Lucca, Piacenza). The Valencia flood in 2024 took place 10-12 days later than the floods in Italy, and

(compared to the Valencia case) in Algeciras the storm Dana caused in the harbour more traffic interruption in these days than any (rainfall-related) flood in the broader surroundings of the Gibraltar Bay.[276] The October 2024 flood in Valencia was caused by a "cold drop" event caused by a "cut-off low" blocking pattern[277] – a synoptic pattern that might have been also responsible (supported by the generally wetter preceding conditions in the Mediterranean) for the destructive floods reported in and around the Gibraltar Bay in mid-October 1342.

4.9 Event 9) Mid-November 1342

Floods: Upper-Tisza, (Latorica), Brenta, Po and tributaries (SE-Slovakia, NW-Ukraine, NE-Hungary, N-Italy)

Cause: heavy rains (documented in N-Italy)

In mid-November 1342, great and extraordinary floods of the River Po and its tributaries (and generally, in Lombardy) and the River Brenta (Padua; but also reaching the Venetian Lagoons) occurred in North-Italy, reportedly caused by heavy rains. Completely independent contemporary local source suggests that, on the same day (and several following days), there was an obstructive inundation in an area under the influence of the River Latorica (W-Ukraine, E-Slovakia) and to some extent also the Upper-Tisza (W-Ukraine, NE-Hungary; Supplementary Data 1, 2).[16] The charter context regarding the inundation in the Latorica and Upper-Tisza catchment also allows a somewhat earlier start/occurrence of the floods, meaning that it is also possible that the flood occurred already some days before and, as inundation did not recede enough by the time of the perambulation, even if the perambulation could be mostly carried out, it could not be made in its full extent because of the waters. Furthermore, the charter text is not detailed enough to specify the magnitude and therefore only an at-least notable flood magnitude can be assumed. Nevertheless, it is rather possible that the flood causing the inundation was larger than notable in magnitude and spatial extension.

Based on the Pan European Flood database,[1] the closest match is the infamous catastrophic floods of early November 1966, when Central and Northeast-Italy (including the catastrophic Florence flood on 4 November), Spain, South-France, Southwest-Hungary and North-Croatia were hit by floods (*Fig. 4.9.1*). The catastrophic flooding was caused by a very intensive short-lived cyclone that developed in the western Mediterranean.[278] The 4 November 1966 event reached a particularly high intensity because the Mediterranean low-pressure centre was connected with an even deeper, large-scale cyclone that developed close to Iceland. Through the trajectory between these two cyclones, unusually cold air arrived from the subarctic area to the Mediterranean.[269,270] While a notable difference between the early November 1966 and the November 1342 floods is that no flood report is available from North-Central Italy, the large-scale flooding in North-Italy (even in its slightly more southerly parts) shows clear similarity with the November 1966 event, when the alpine area of Italy (and Austria) was also strongly affected, and floods also extended to the Carpathian Basin.

Even if, usually, there is a clear secondary flood maximum in (autumn-)winter (November-December)[213] on the Upper Tisza and its tributaries, there are only a few cases in the period 1960-2020 when larger floods occurred in the same time in North-Italy and in the Carpathian Basin in November, and in these cases more often the Southwest-Carpathian Basin, and somewhat less frequently the north-eastern parts of the Carpathian Basin (i.e., the Upper-

Tisza catchment) were affected (*Fig. 4.9.1*).[269] Despite the heavy rainfall in late October 1966, there was no significant flood in the Upper-Tisza catchment in early November 1966 and only a couple of tributaries were in flood (see *Fig. 4.9.1*), but this was largely due to the preceding very low water levels on the Upper Tisza and its tributaries that extended almost to the entire year: the low/very low water levels had been typical in the entire catchment since March 1966. In late October–early November, within some days, there was an almost 3.5 m water-level rise on the Upper Tisza,[235] which reached an almost 4.5 m water-level increase on the Middle Tisza,[236, 279] followed by another higher water-level period in the second half of November. Although none of the water-level rise events can be considered as floods, the water-level increase was rather significant (particularly on the Middle Tisza with an already broader riverbed), around the same time in early November 1966, when much of Florence was heavily flooded.

Another, still relatively close match is, for example, October 1998 when, amongst others, there were important floods both in Northeast-Italy in early October, caused by heavy rainfall related to intensified Mediterranean storm activity.[280] There was also a flood on the Upper Tisza (and the tributaries originating in the Northeast-Carpathians, including the Latorica) in late October–early November as a result of enhanced precipitation related to a Mediterranean cyclone. However, there was a considerable, over 3 weeks difference between the two floods and the highest water levels (much higher on the Tisza than in 1966 – as the year was also notably wetter with high average water levels). Later, another flood occurred on the Tisza in mid-/late November,[235,236,281] but no considerable floods are known from Italy in November 1998.

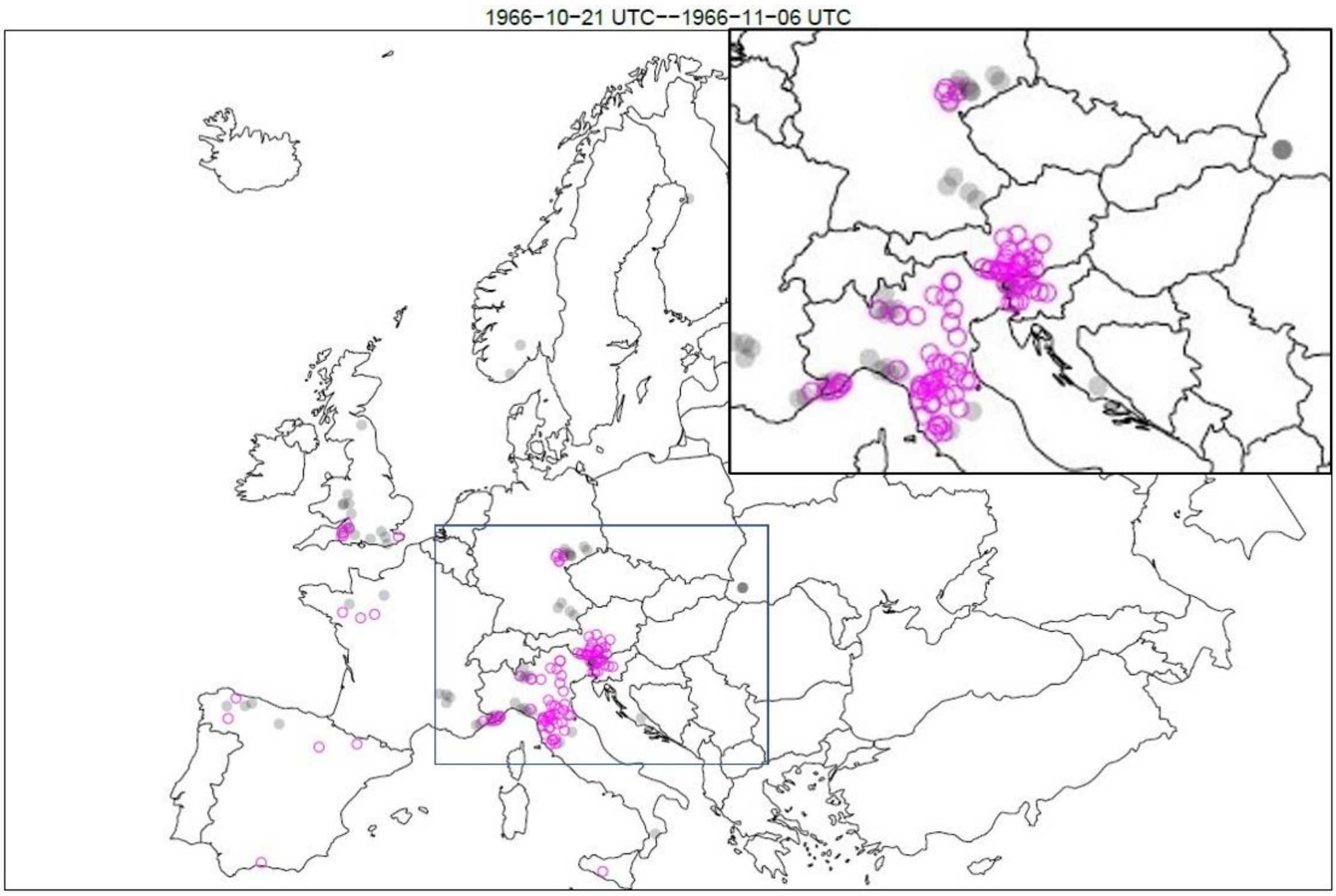

Figure 4.9.1. Floods exceeding the 5-year return period limit (magenta circles) and/or with annual maximum (grey circles) in the first[1]

Similar to, for example, the floods in 1966 and 1998, the mid-November 1342 simultaneous flooding in North-Italy and the Northeast-Carpathian Basin probably was a result of enhanced Mediterranean (and related Central-European) storm/cyclonic activities (in connection with sub-artic cyclone activity). Although we can only estimate the Northeast-Carpathian Basin flood on an at-least basis (it was an at least notable flood, but possibly it was greater in magnitude), the Italian floods were clearly great or outstanding in magnitude. A relatively unusual phenomenon is the practically simultaneous occurrence of the floods that likely refer to prolonged wet conditions of the preceding weeks/months in the Northeast-Carpathian Basin and the Carpathian Mountains as a result of preceding enhanced large-scale (Central-)European cyclonic activity.

4.10 Case 10) February 1343

Floods: pluvial flood(?) in Northwest-France, flood on the Middle Tisza (NE-Hungary, SW-Ukraine – probably also N-Romania)

Cause: excess rainfall, (followed by) sea storm (Tisza: potential causes: snowmelt and rain? Causes of flood are not mentioned by contemporary source)

In January 1343 possibly there was a sea surge in North-France, Netherlands, while in early/mid-January there was much rain in Northwest-France that reportedly stayed over the ground and developed to some kind of inundation by mid-/late January (rainflood or groundwater flood). Flood was reported in mid-/second half of February on the Middle Tisza (Supplementary Data 1a).[16] In February, a great and probably long-lasting sea storm hit the Atlantic coastline between England, Northwest-France and Spain (La Manche, Vizcaya Bay) and the same or another sea surge caused dyke breaches in Frisia in the same year. The Merle diary[158] in South-/Southeast-England suggests a wet (but not particularly frosty) January and colder conditions (with notable frosty periods) in February. There was cold/frost during the winter in North-Greece, while both South-/Southeast-Spain and North-Greece faced much precipitation (Supplementary Data 2a,b).

The 1342 flood reports that come from the eastern part of the Carpathian Basin and the rain-related flooding data from North-west France, seem to be the consequence of a wet (early/)mid-winter. Although the available information comes from a relatively long time-span, they are most probably part (and consequence) of a prolonged wet period. A flood in February on the Tisza is in most cases a consequence of early snowmelt and the arrival of mild/wet air masses. Moreover, during mild (wet) winters high water levels or floods can also happen in January.[198] While the mention of a frosty period in winter in N-Greece may suggest significant cold periods, the Atlantic storms, much rain and the mid-February Tisza flood altogether suggest a winter with cold periods (of unknown length) in the eastern part of Europe, with notable mild interruptions and considerably wet conditions.

The closest parallel event in the period 1960-2020[1] occurred in early/mid-February 1979 (*Fig. 4.10*), when the (Middle) Tisza flood reached over 900 cm at Szolnok: the flood already started in early January, but it reached its maximum height in mid-February; the flood only ended in early March.[236] However, on the Upper Tisza there was flood only from the end of January to early February and the flood reached Szeged in early February.[235,279] There was also flood on the River Maros at the beginning of February, and on the River Werra in East-Germany

in January. In England, there was a great sea storm on 13 February that caused tidal flood and damages in the south of England,[282,283] followed by flood of the Yorkshire Ouse and the Tay in England on 1 March.[283] In the same time there was flood on the Lower Tagus, flood and landslide in Portugal, and there was also flood on the Upper Danube, reaching Hungary only in March. It is also important that a moderate flood already happened in February in North-Greece.[283]

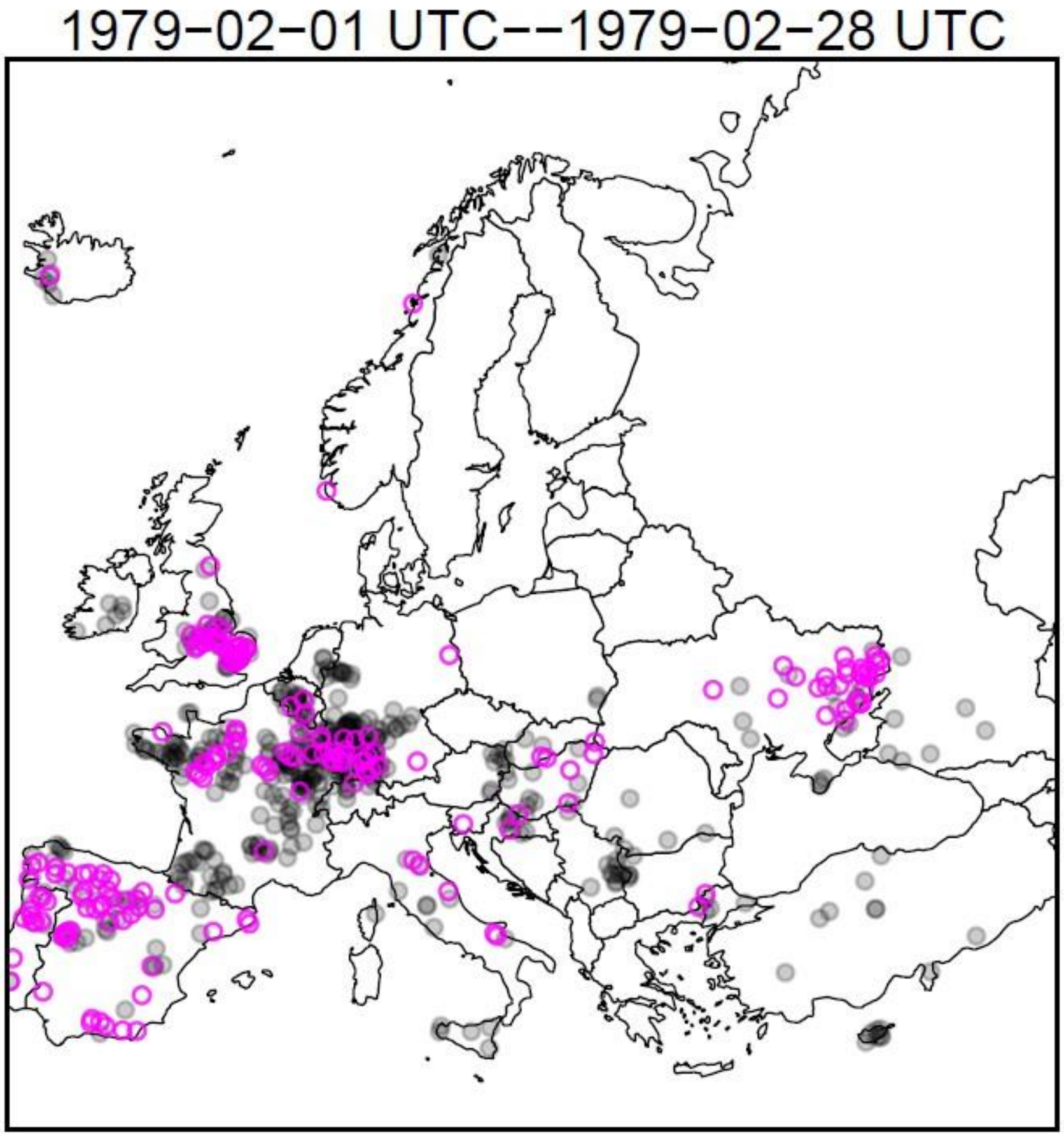

Figure 4.10 Floods exceeding the 5-year return period limit and/or with annual maximum reached in February 1979 in Europe, based on the Pan-European Flood Database[1]

Other cases, showing less somewhat less similarities, are the Tisza floods that happened in February or March between 1960 and 2020 several times (e.g., Feb.: 1966, 1977, March: 1967, 1981, 1999): only in case of 1979 it was accompanied by very wet January in Western Europe, and this was also a year with sea storms in February along the Atlantic coast. Another interesting case occurred in January 1986 when an early-January North-Atlantic Sea storm was followed by a moderate flood on the Middle Tisza in late January–early February, and in March 2008 when, on the other hand, the moderate flood on the Middle Tisza occurred both in mid-March and mid-April. The sea storm generation and general synoptic patterns in both cases showed close similarities to the February 1979 event.[236,284]

Naturally, with the January rainflood in the west, sea storms along the Atlantic coast and a mid-/late February Tisza flood with other flood patterns, not happened in the same form in 1960-2020, could also occur. Nevertheless, based on the studied period the patterns in (January-)February 1979 show rather strong similarities (in flood and weather) to the January-February 1342 case. Another important point is that, mainly on the Upper Tisza (but usually it

also reaches the Middle Tisza), usually there is a secondary flood maximum in winter. This winter secondary flood maximum is predominantly related to Central Mediterranean cyclonic activity (Genova low), and – although river flood reports are not available (yet) from other areas in Europe – it is generally related to larger-scale weather systems causing floods around the same time also on other, Mediterranean (and some Central European) rivers. And this is especially true when combined with high storm activity and much rain (also causing rainflood/groundwater flood related inundation) along the Atlantic coast, as it happened in 1343.

In general, the January and February storms along the North Atlantic coastline, the prolonged rainy weather in January in Northwest-France and the mid-/late February flood on the Middle Tisza altogether suggest a (milder early/mid-)winter that was rather rich in precipitation, combined with enhanced Atlantic storm/cyclone activity. The frosty weather mentioned in the Balkan as well as the frost-rich February in Southeast-England may indicate a rather colder late winter in large parts of Europe and the enhanced activity of the Siberian High at least in some parts of the winter – the latter statement is indirectly also supported by the mid-/late February Tisza flood, with the addition that at least one considerable mild (and wet) interruption had to occur in East Central Europe (latest) in mid-February.

Thus, these data altogether suggest a predominant precipitation-rich winter with changeable temperature conditions over Europe. In accordance with the long-term NAO reconstruction of,[336] these general conclusions on the winter weather patterns suggest that the NAO conditions were less negative than during the previous (1341/1342) winter, and could also refer to the more northerly position of the Atlantic Jet Stream.[32]

4.11 Case 11) April 1343

Floods: sea surge in Algeciras/Gibraltar Bay, River Tisza

Causes: storm (presumably rainfall, maybe with snowmelt – no cause provided by source)

The great flood of the Upper Tisza (with a catchment in modern E-Hungary, W-Romania, SW-Ukraine)[16] in early April and the sea storm and coastal flooding described in Southeast-Spain some days before – combined with the weather reports from Central Europe and south of England – suggest wet conditions in large part of Central Europe and cyclonic activity in the Western, Central Mediterranean. The weather was cold in early April in Hertford, there was strong wind/windstorm in early April in Algeciras, and a generally wet April in Southeast-England (Supplementary Data 1, 2).

Apart from the rainy weather in early April in Central Europe, the violent thunderstorm, reported at the end of March (in today's W-Germany), raises the possibility of sudden warming conditions in Central Europe, followed by a rainy period that could be also responsible for a great flood on the Upper Tisza. While a Mediterranean cyclone usually has local/regional consequences, especially in the Southwest- and/or the Northeast-Carpathian Basin, melting and rainfall-related flood caused by western air masses are usually part of a larger, supra-regional (e.g., W-, Central European) scale flooding.[285]

Water bodies, and rivers in particular, in different parts of the Carpathian Basin are good indicators of the conditions/flood waves in large part of Europe: for example, autumn and winter floods of the Upper/Middle Tisza and the Drava are often the consequence of large-scale weather patterns related to the Genoa low pressure system, forming into cyclones of Mediterranean origin, following the Vb path (special type of Südwest-Wetterlage). Depending on the general European weather patterns, a flood for example on the River Tisza can as well be the consequence of weather patterns following the western path (Westlage) – in this case usually larger part of West- and Central Europe are affected. Common characteristics is that when a more significant flood is reported on the Upper/Middle-Tisza, it practically never means isolated flooding, but – depending on the season and the general European patterns – it is related to large-scale flooding events of varying magnitude (i.e., it can be part of a notable and even spatially extensive but overall not particularly damaging floods in most of the affected areas). In our current case, even if a great flood related information is only available regarding the River Tisza, together with the available European weather data (including preceding precipitation conditions) and based that the possible modern (1960-2020) parallels, we can approximately define an area in Europe that could be affected by some kind flooding.

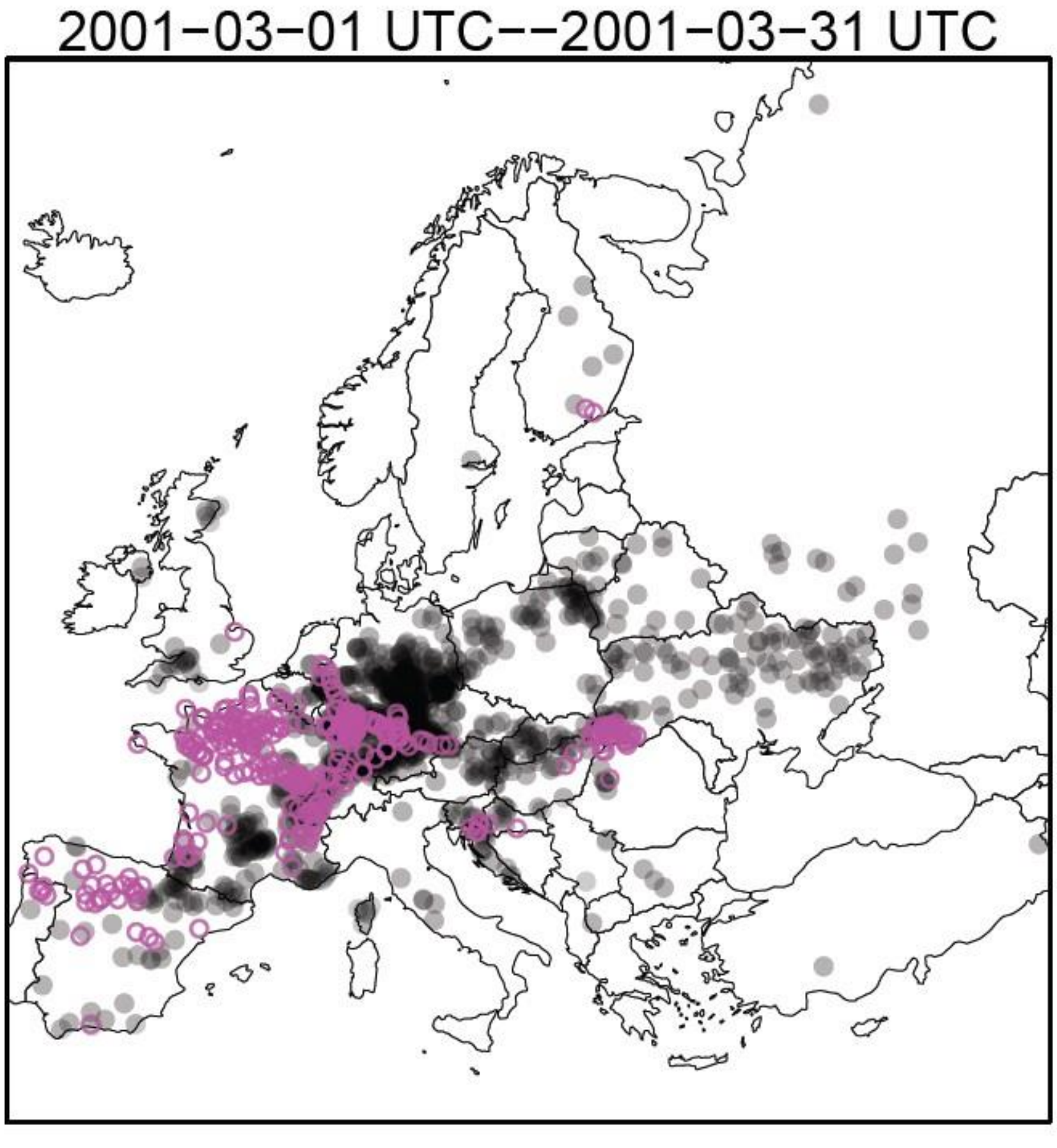

Figure 4.11 Floods exceeding the 5-year return period limit and/or with annual maximum reached in March 2001 in Europe, based on the Pan-European Flood Database[1]

Based on the comparison of flood and weather patterns in Europe, there are several cases in the 1960-2020 period that shows similarities to the April 1343 events, but the March–April 2001 flood(s) show probably the closest modern parallel (*Fig. 4.11*): in this case there was a sea storm in early March,[286] and within some days (in ca. mid-March) a sudden great water-level increase happened on the Upper Tisza. The Tisza flood decreased after a few days and

then was followed by a smaller flood wave.[235,236,285,281] Despite the relatively dry general weather conditions following the April 2000 floods, and the low quantity of snow in winter, the March 2001 event was particularly destructive in the Transcarpathian region of Western Ukraine: the flood was caused by sudden very heavy precipitation. In early March, within a couple of days over 200 mm precipitation fell[285] that – with the melting of the snow (from an important but not outstanding late-February snowfall) and the still largely frozen ground – suddenly caused strong flooding (and dyke breach) in large part of the Upper-Tisza catchment.[285] The outstanding precipitation (in combination with snowmelt and the effect of frozen ground) was caused by a specific macro-synoptic pattern, characterised by a WSW-ENE frontal zone in the middle troposphere, with a two-centred shallow cyclone attached: the first centre was over France and the over (south of the) eastern Alps – the latter moved to the Western Ukraine, causing the outstanding precipitation over the North-east Carpathians.[287]

This latter information might be important also regarding the April 1343 flood case(s), because the fact that the perambulation was rendered (and participants were invited and travelled there) may suggest that the preceding weather (floods) conditions did not give a particular reason not to carry on the perambulation on or around the planned date (days, week). Thus, the fact that the great magnitude of the flood was emphasised in the charter may pinpoint to the relatively unexpected nature/extension/magnitude of the event.

In April 2018 a similar flood wave with a Tisza flood happened that (apart from the Tisza catchment) also affected Austria, Slovenia, Hungary and some parts of Spain but, in this case, there was no sea storm in the south of Spain.[286] Further possible parallel is the flood in late January–early February 1979; however, just like in 2018, the strong wind conditions did not develop to a storm in Algeciras before or during the Tisza flood. The latter case also shows some similarities to the consequences of the Mediterranean storm Zeus in early March 2017, but the Tisza flood was of lower magnitude than in 1343 and the strong winds did not end up in storm in Algeciras.[288] A relatively similar case, with a slightly earlier occurrence, took place in February–early March 1989 with storm in Algeciras in late February[286] and a smaller flood wave on the Tisza in early March.[235]

Additionally, according to some much later, early modern and modern sources, included in the compilation of Curt Weikinn[210] – such as the 16th-century Fabricius,[289] the 17th-century Güth[290] and the 19th-century Klemm[291] –, floods of the Elbe and the Werra would have also occurred in the second half of March, in 1343. Although these Central European floods would be in good agreement with the presented contemporary flood data in East Central and Southeast Europe, without referring to any original contemporary sources, the source quality and credibility of these German data are unclear. Moreover, as these reports partly or mostly originate from the above-mentioned 16th-century source (Fabricius), it is possible that this 16th-century information is based on a transcription mistake of an earlier work. Thus, these flood data were not included in the database, final analysis and reconstruction.

4.12 Case 12) May 1343

Floods: East-/Northeast-Carpathian Basin (Upper- and Middle Tisza), Balkan (Axios)

Causes: heavy ranfall (Axios; no causes provided regarding the Tisza, but most probably great/prolonged rains)

Great flood was reported in early May on the Upper Tisza and mid-May on the Middle Tisza, and a great flood occurred on the Axios (N-Greece) in May 1343.[16,212] The one-week difference between the two perambulation patterns on the upper and the middle sections of the Tisza presumably not only refers to the same major flood wave,[292] but raises the possibility that not only the Upper-Tisza (i.e. NE-Carpathian Basin) but also some of the Middle-Tisza tributaries (i.e. most or the entire E-Carpathian Basin) were in flood. As no information is available when in May (or in the entire May) the flood of the Axios/Vardar started and for how long it lasted, but as the flood on the Tisza lasted (on the different sections) for a longer period of time, most probably there was overlap in the timing of the floods of the two rivers. However, the fact that within a period of weeks (or less) the north-eastern and eastern parts of the Carpathians and parts of the Balkan were affected by floods increases the possibility that flooding of the Axios and the Tisza was part of a larger-scale, European flood event. Mid-/late April was reportedly very rainy in the Bodensee area, and the April was generally rainy in Southeast-England while the summer (presumably also including May) was (unusually) rainy in Iceland (Supplementary Data 2). Similar to March 1342 (Case 3), the flood affected the Southern Balkan and the Upper- (Middle-)Tisza catchment.

Based on the Pan European Flood Database,[1] the (quasi-)simultaneous flood of the Tisza and the Axios is not a frequent phenomenon, it occurs as a consequence of a prolonged wet period and increased atmospheric instability, meaning that such flood occurrences are usually part of a larger-scale, European flood event. The closest parallel in the 1960-2020 period was the great flood in April–early May 2000 (see *Fig. 4.12*). In this year, floods affected both the Axios and the Tisza; moreover, on the Tisza not only the upper-section, but also middle-section tributaries (e.g., the Körös) were hit by heavy flooding. Although the spatially extensive European floods were usually moderate in magnitude, the flood of the Tisza was particularly great, also named as the "flood of the century" (i.e. 21st) in the Upper-/Middle-Tisza region.[293] The April-May 2000 case also fits our 1343 case rather well because also in March 2000 there was an important flood roughly the same time before in the Upper-Tisza catchment[294] as in February 1343 and, because of repeated rainfalls, the water levels remained high throughout most of the spring.[295] Furthermore, due to the very wet spring, just like in 1342, important floods already happened in the Tisza catchment in the previous year, in 1999.[296-298]

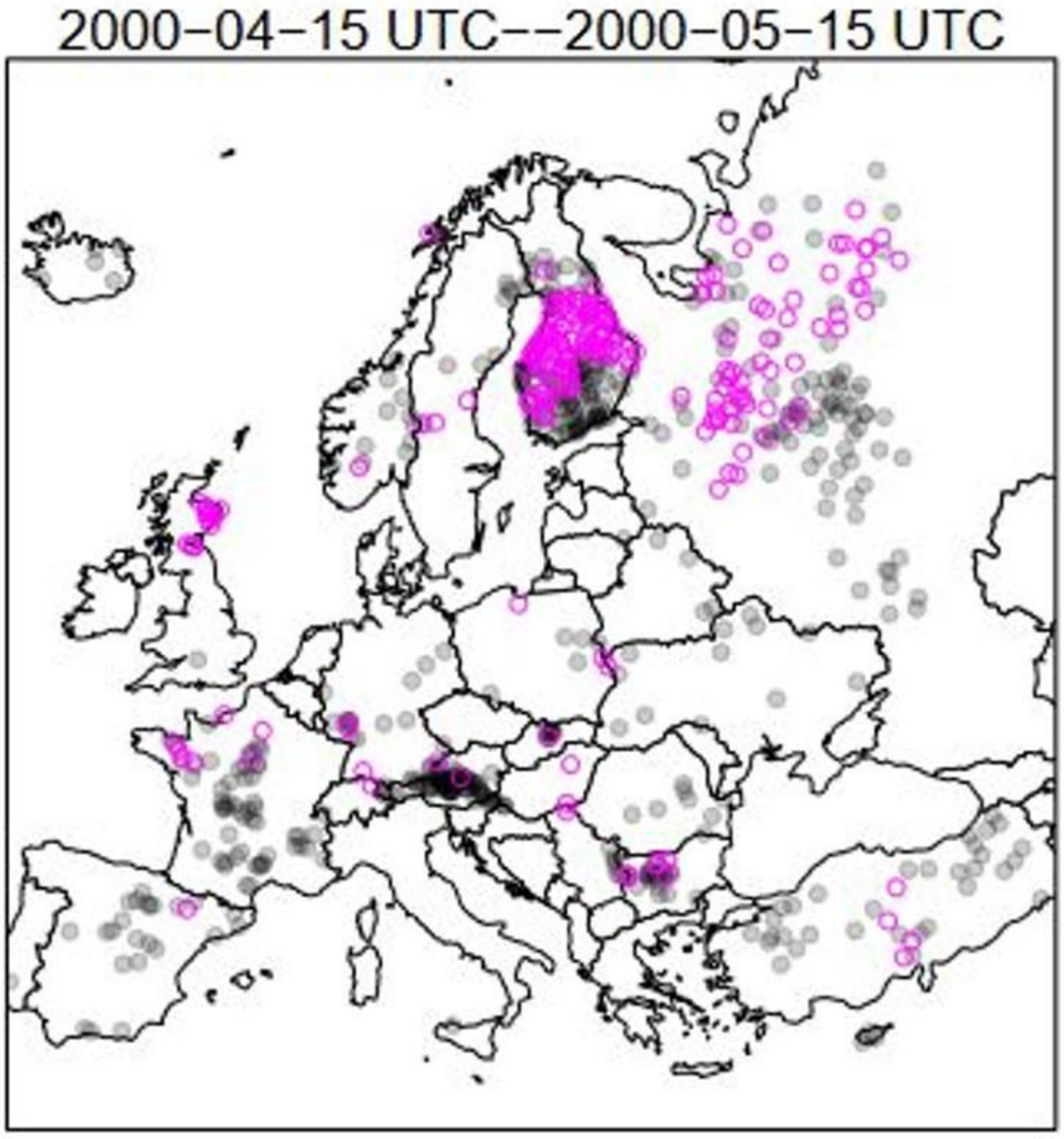

Figure 4.12 Floods exceeding the 5-year return period (magenta circles) limit and/or with annual maximum (grey dots) reached in mid-April to mid-May 2000 in Europe, based on the Pan-European Flood Database[1]

Although in spring 2000 there were strong floods also in Finland and some parts of Scandinavia, and Britain and Eastern Europe were also among the affected areas, these areas were not included in the reconstruction because of their distance from the main flood cluster. Even if prolonged rains were reported from Ireland in July 1343, and from north of England in August 1343 (see Supplementary Data 2), no flood was directly mentioned in these areas in contemporary sources. Another partly similar case occurred in March 2001 when both the Tisza and the Axios flooded. Nevertheless, the 2000 case fits somewhat better as it was a longer flood wave with multiple peaks: in 2000, in addition to the March–April flood waves, there was also a flood peak in May. A further parallel could be (late) May 1991 with the difference that in that year there was only a small flood on the Upper Tisza that clearly did not reach the level of the great flood that occurred in May 1343.

Curt Weikinn[210] in his historical flood data compilation again refers to the same 17th-century source[290] as before, suggesting that, apart from the March flood, there would have been a flood on the River Werra at Meiningen, Thuringia, also in early May in 1343 (allegedly caused by heavy snowfall and thaw). As no contemporary source is available that could support this information, we do not use this data in our analysis and reconstruction. However, the timing of the Meiningen flood reported by the 17th-century source (if it is not a simple misdating instead of, for example, 1342) would coincide with the great flood on the Upper Tisza, discussed above. Moreover, also in April 2000 there was notable flood in Thuringia and Saxony.

4.13 Case 13) Jacob Flood: July–early August 1343

Floods: Northeast-Carpathian Basin (Sajó; and presumably also the upper Middle-Tisza), Switzerland (Töss, Reuss, Limmat, Lake Lucerne, Lake Zürich, High Rhine, Lake Constance), Bavaria/South-Germany (Altmühl and presumably also the Danube)

Causes: very wet antecedent conditions, incessant/heavy rains (presumably Vb event)

In late July–beginning of August there were outstanding floods in (concerning modern borders) Central/East- and North-Switzerland and South-Germany, reportedly on the Rivers Töss, Reuss, Limmat (together with Lake Lucerne and Lake Zürich), the High and Upper-Rhine and Lake Constance. Furthermore, "deluge", similar to the one exactly a year before, was also mentioned in the same time along the Altmühl close to the Danube, presumably referring to both rivers. The Jacob Flood might have consisted multiple flood waves, as the first report about the great flooding is dated already to 29 July (JC: 21 July) in Strasbourg, while floods along the Töss, Limmat, Reuss, High Rhine, Bodensee/Lake Constance and in the Altmühl–Upper-Danube area were primarily reported in connection with the 2 August (JC: 25 July) event. In the Lake Constance area, the flood (mentions) continued on 4, 9 August; the flood on Lake Constance and the High Rhine lasted for weeks (Supplementary Data 1a). Moreover, hardly more than a week before the first Upper-Rhine flood report, another flood was mentioned (on 19 July – and the days around) on the lower section of the River Sajó, close to its inflow to the Middle-Tisza.[16]

Concerning weather conditions in Europe, the summer was mentioned as (particularly) rainy in Iceland, suggesting pronounced low pressure conditions, while tree-ring evidence suggests warmer and sunnier, and therefore predominant high-pressure conditions in Scandinavia and large part of Eastern Europe, while (April–June was extraordinary wet in the Lake Constance area (Supplementary Data 2a,b). The flood was mentioned in the Northeast-Carpathian Basin just a few days before (19 July and the days around), along with devastating thunderstorms in North-/North-Central Italy (22 July). Around the same time the weather changed to a notably rainier character in Ireland. Caused by multiple days of torrential rainfall, the majority of reports date the outstanding flood to 2 August (JC: 25 July: day of Jacob) on the High Rhine, Lake Constance and the lakes and rivers of Central and East-Switzerland (Supplementary 1a, 2a). On the same day, a "deluge" was mentioned exactly a year after the July 1342 "deluge" along the Altmühl near the Danube. Even if the summer was predominantly dry in the south of England (Supplementary Data 2a), 2-3 August (JC: 25-26 July: the timing of the Jacob Flood in Central Europe) was with heavy rain and thunders. In East-England (Lindsay, Lincoln), after 4 hot days in the second half of July, in late July–early August a rainy period started. On 5-8 August (JC: 28-31 July, i.e., still during the Jacob Flood in Central Europe) thunders and rain were reported every day, and there were also thunderstorms on 7 and 8 August (JC: 30-31 July).[158]

As for comparative modern events, the weather and flood patterns of late July–early August 1343 show important similarities both to the August 2005 Central European flood (*Fig. 4.13*)[299,300] that affected Austria, Switzerland, South-Germany (E-France), Romania, Hungary, Slovenia, Poland, Serbia, Bulgaria and Moldova, and to an almost similar extent also to the 'Pentecost Flood' of May-June 1999. In both cases there were outstanding floods in South-Germany, Switzerland, West-Austria, and especially in South-Bavaria and the Lake Constance area, and there was also flood in Northeast-Hungary and other places in East-Central and Southeast-Europe[301,302] (see also *Event 14*). Both the 2005 and the 1999 events were caused by

cyclones of Mediterranean origin showing strong similarities to the classic Vb pattern).[300,302] The 2005 floods were the result of a cut-off low that "*was approximately circular and the flow towards the Alps was from the north sector*", while the 1999 event "*had a zonally elongated cut-off low with advection from the northwest*").[303] This difference in synoptic patterns resulted in a slight difference in the intensity of precipitation, as in 2005 (like in late July 1343) primarily the central part of Switzerland was affected by the first and highest rainfall totals,[303] and then the precipitation centre was moving towards the north-east (as was perhaps the case also in July 1343), while in 1999[303] the highest precipitation totals affected Northeast-Switzerland (which was, based on the available contemporary sources, more typical for the Bartholomew Flood).

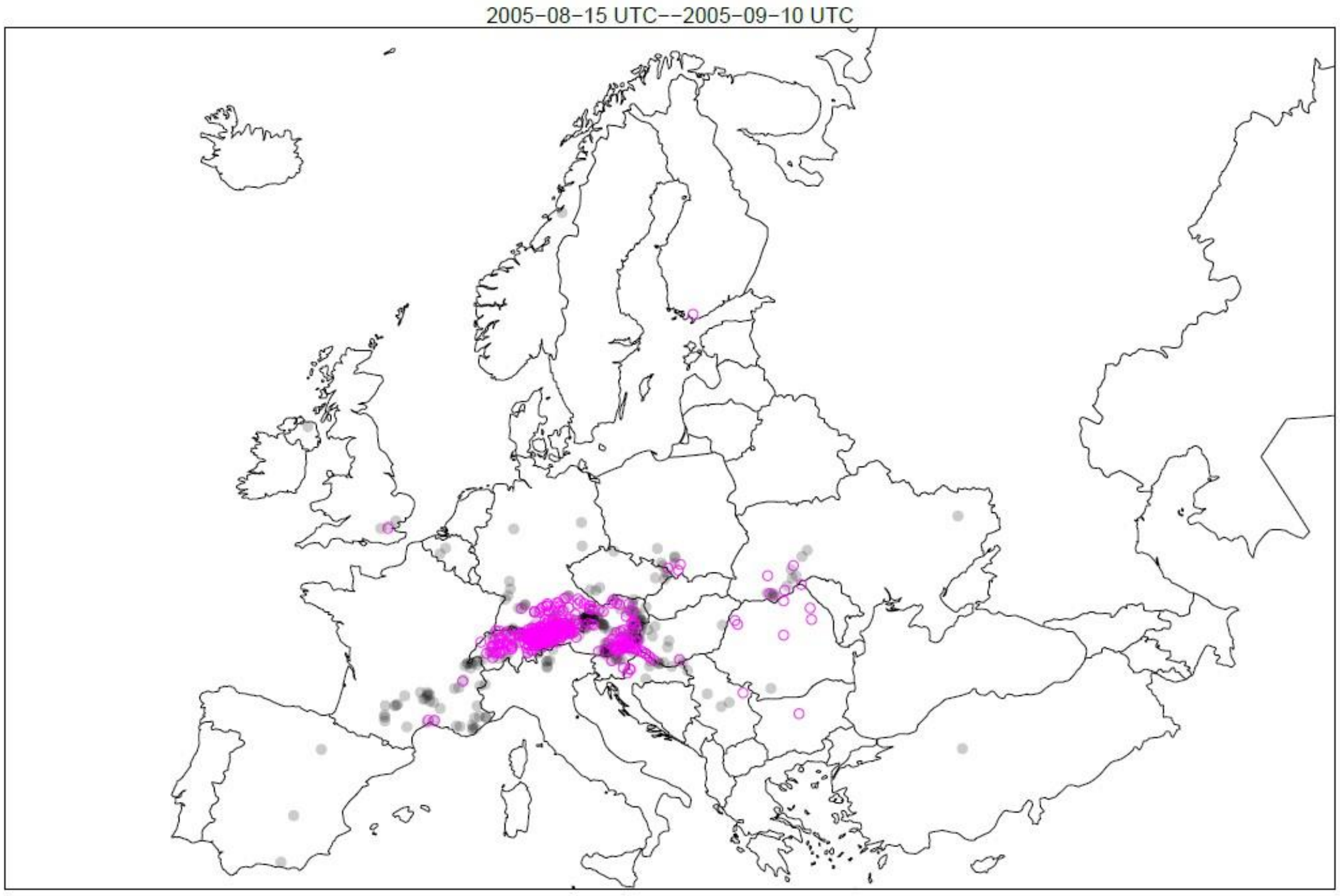

Figure 4.13 Floods exceeding the 5-year return period limit and/or with annual maximum reached in March 2001 in Europe, based on the Pan-European Flood Database[1]

While the 1999 flood occurred in late May–early June, the 2005 flood happened only a couple of weeks later than the Jacob Flood in 1343. Following a wet period when soil became saturated, the August 2005 floods were closely related to the low-pressure/cyclone system "Norbert".[300] In Switzerland the torrential rains and the flood were immediately preceded by high-intensity convective rains in August 2005, which may show some parallels to the late July 1343 conditions when the High Rhine was already in flood at least a couple of days before the main flood wave mentioned on the High Rhine on 2 August. Based on all this information, the August timing, and the lack of a new Sajó and Tisza flood wave in May 1999 (after the March floods: see the Pan-European Flood Database),[1] the (July–)August 1343 floods show somewhat closer similarities to the August 2005 floods.

As we could see before, already the year 1342 was rather wet with many floods in Central Europe. In 1343, at least parts of winter were also wet, and in the areas affected by the floods in July and August most of the spring and the early summer were also unusually wet. These circumstances resulted in unusually wet antecedent conditions, a prolonged period of (over)saturated soils and high lake/river water levels prior to the Jacob Flood. The areas affected by the torrential rains and floods in Central Europe in late July–early August 1343, the preceding flood in the north-eastern part of the Carpathian Basin, the stormy conditions in

North- and North-Central Italy, the rainy summer in Iceland, the intensity, duration of rainfall and floods in Central Europe, the prevailing climatic patterns in this summer (and the southerly position of the North Atlantic Jet Stream this year: see e.g. Supplementary Data 1, 2, 5, Supplementary Information 5.2) show the classic patterns of an outstanding large-scale flood event primarily related to cyclonic activity following the Vb cyclone path.[248,259]

The striking similarities with some of the individual modern flood parallels also support this idea. Apart from the fact that flood-affected areas show a classic Vb cyclonic activity related local/regional flood distribution and intensity, also the information regarding the large-scale European weather patterns suggest important similarities, for example, to the schematic illustration of typical atmospheric blocking situation in warm season, presented by Kautz and his colleagues.[304] Although we have no information about the sea surface conditions of the Mediterranean in this year and general weather information makes high sea surface temperature conditions in the (northern) Mediterranean generally less likely (in 1342 and 1343), the analysis of synoptic conditions of the greatest 19th-21st century Vb-related floods also show that higher wind speed from the Atlantic is a more important factor in the development of really great floods than the higher sea surface temperature conditions of the Mediterranean.[248]

As during Vb-related great/outstanding floods – affecting Central, North-/East-Switzerland and Bavaria – the (Upper) Danube is usually also in flood (as happened in 2005 or 1999), and a contemporary “deluge” mention is available in 1343 only some km-s from the Danube, as well as the fact of Danube regulation works that started only some months later with reference to the consequences of the previous floods (Supplementary Data 3), it is rather possible that the Upper Danube also flooded in in August 1343, even if the magnitude of this flood is unknown. Similarly, in 2005 the outstanding mid-/second half of August flood of especially the Rivers Sajó and Hernád and partly also of other tributaries of the Upper-Tisza caused a notable (but not large) flood wave also on the Middle Tisza.[236] Since the Sajó flood in mid-/second half of July 1343 was reported in an area very close to the inflow of the river to Tisza, the influence of the Tisza water-levels had to be considered: The (at least notable) flood of the Sajó in that particular location, near its inflow to the Tisza,[213] also suggests the flooding of the (upper) Middle Tisza. For other potential historical parallel(s), see also Event 15.

Regarding historical events prior to the 20th century, outstanding floods with rather similar extension and comparable magnitude (heavily affecting both Central and Northeast-Switzerland) to the one in late July–early August happened in 1566 and 1817. These two extreme floods are listed among the most outstanding floods of the last millennium both on Lake Zürich and Lake Constance: in 1817 based merely on maximum water levels and return periods, the Lake Constance flood was somewhat greater than the Lake Zürich flood, while in 1566 the opposite was the case.[305,306]

Apart from the contemporary source-based data, included in Supplementary Data 1a, in the near-contemporary *Grösseren Basler Annalen*, set up in the early 15th century (until 1416), a Rhine flood is also mentioned, but under the year 1340 (“*Im jar darnach /1340/ was der Ryn ouch seer gross. / Anno 1340 Jacobi erat Reni magna inundatio*”).[307] The *Kleiner Basler Annalen* also mentions for the previous year (1339) that the heavy flood (“*magna inundatio*”) of the Birsig river also caused significant damages in Basel. Rather similarly, the Birsig-flood is mentioned for 1339, and the great Rhine flood for 1340. However, in these annals it was also reported that the water took the Rhine bridge in Basel, and then the unknown author(s) listed exactly the same settlements (and their destroyed bridges), namely Laufenburg, Reinfelden, Säckingen and Brisach, what Winterthur had mentioned along with the great late July 1343 (High-/Upper-)Rhine flood event[307] – this list and the great damages well corresponds to the (the contemporary) Diesenhofen's report who (as we could see earlier) mentioned that in late July 1343 all bridges between Rheinau and Breisach were destroyed by the great Rhine flood.

Therefore, we agree with the (modern) editors of both the Grösseren and Kleiner Basel annals wo suggested the flood event under 1343 (instead of 1340, written in the manuscripts). Unlike the Kleiner Basler Annalen and the Grössere Basler Chroniken (that dates it for 1340), the Schnitts version of the (early 15th-century) Grössere Basler Annalen also dated the great Rhine flood that took the bridges and destroyed towers and villages for 27 July 1343.[308] As these later sources do not add significant new information to those provided by contemporary sources, we do not directly utilize them in the 1342-1343 flood database. And while the connection between the 1343 High-/Upper-Rhine flood and the flood mention (under the year 1340) in the Basler Annalen, the date of the Birsig flood remains a question, and therefore we did not include into our 1342-1343 flood database.

As for further, non-contemporary and scientific literature data (without source reference) concerning the Reuss river and the connected lake, Schaller-Donauer[309] refers to a text, taken from an unspecified source, with rather similar content as provided by Winterthur, with dating the flood to 29 June. This later date also appears in the collection of Klemm,[310] providing the weather-related texts found in the annals of the Thann friary.[332311] Furthermore, Schaller-Donauer,[309] without providing source reference, added that the very rainy May and June with the snowmelt caused the great flood, and there was in fact already flood on the Schächen in mid-June which resulted great damages in Bürglen, Altdorf and one part of Schattdorf. However, without historical source references, these data would be problematic to use in the analysis.

4.14 Event 14) Bartholomew Flood: Late August–early September 1343

Floods: South-Germany, Northeast-Switzerland, Eastern France (High and Middle Rhine, Bodensee)

Causes (documented): very wet antecedent conditions, incessant rains/heavy rainfall

Flood on Lake Constance/Bodensee and on the High and Upper Rhine reached its maximum level in late August–early September 1343, while based on the 1343-1344 Danube regulation records and a (general) contemporary report from along the Altmühl, only a few km-s from the Danube, there had to be a flood around on the Danube as well (Supplementary Data 1a). The Bartholomew Flood in the Bodensee/Lake Constance–High-/Upper-Rhine area covered around the same area as the Jacob Flood, as if this flood with its even higher maximum flood levels both on Lake Constance and the Upper Rhine would have superimposed the (prolonged) Jacob flood. Although already the Jacob Flood a month before had extraordinary magnitude and, based on the description of Johann von Winterthur, the water level of the lake stayed very high for many days (maybe even continuously) in August, the maximum water levels of the Bartholomew Flood of Lake Constance and also that of the Upper Rhine were even higher than those of the Jacob Flood.

Another considerable difference from the Jacob Flood is that no flood report is known from the Carpathian Basin or the Upper-Danube catchment (which in itself does not necessarily mean the lack of flooding), which may indirectly suggest that this extraordinary flood was not necessarily the result a Vb event (though it is still possible) but the eight-day heavy rains (Winterthur phrases it as an eight-day "cloudburst": see Supplementary Data 1a) could as well be caused by other weather patterns/convective event(s). An important similarity with the Jacob

Flood is that, just like the days before and during the Jacob Flood, between 22 and 26 August (JC: 14-18) 1343 heavy rains and lightning/thunders were reported every day by Merle in the eastern part of England.[158] On 3 September (in GC), in (North-)East-Anglia a charter referred to a prolonged rainy period (probably started several days or even some weeks before: see Supplementary Data 2a). Both the millennium-scale European summer temperature[249] and (spring-)summer hydroclimate[246,312] reconstructions suggest warmer and drier (i.e., prevailing high pressure, blocking) conditions over Scandinavia and Northeast-Europe in summer 1343 (which could contribute to the development of both outstanding Central-European floods).

From the 1960-2020 period, the event that shows the most similarities with the late August 1343 Lake Constance (and Danube) flood occurred in May–June 1999 (see *Fig. 4.14*), when there was an outstanding ”centennial” flood on Lake Constance and also on the Upper Danube.[301,313] In 1999, the Rhine, Lake Constance and the Danube heavily flooded already in May; the flood lasted long, for several days (weeks) and, similar to 1343, the Lake Constance flood had (at least) two major flood peaks, although the greatest peak was not the last one (and also the Bartholomew Flood was followed within two weeks by another flood in the Lake Constance catchment). However, unlike the 2005 flood, the 1999 flood was primarily not related to a Vb pattern, but was the result of a combination of snowmelt (that was responsible for the antecedent high river/lake water levels) and the heavy rainfalls caused by “*three low-pressure systems in front of the alpine main divide*”: the first heavy-rain period was on 11 to 14 May (this caused the largest water-level increase),[61,314] then on 19–21 May (followed by the largest peak) and, finally, on June 2 and 3.[301]

Another event that shows similarities, but with only a moderate flood peak on Lake Constance, took place in late June 1965 when the Danube also flooded from Germany to Yugoslavia (Serbia) already from the beginning of June. A third, still in some aspects similar flood occurred in July–August 1987 when there were flood(s) on the Danube as well as in Central and Northeast-Switzerland, but again the flood of Lake Constance did not reach an outstanding level. Furthermore, the Bartholomew Flood also shows thought-provoking similarities to the 1817 flood, the “millennium flood” of Lake Constance/Bodensee, with the highest measured historical flood peak on Lake Constance, even higher than the extreme flood of 1566.[315] 1817 was also an interesting flood year, as (just like 1343 that followed the 1341 Hekla eruption – and maybe other eruptions) it happened two years after the infamous Tambora (1815) eruption. The great High-Rhine and Lake Constance floods happened only in 1817 because 1816 summer, the ”year without a summer”, was too cold in the Alps (i.e., no notable snowmelt in the Alps even in summer).[305]

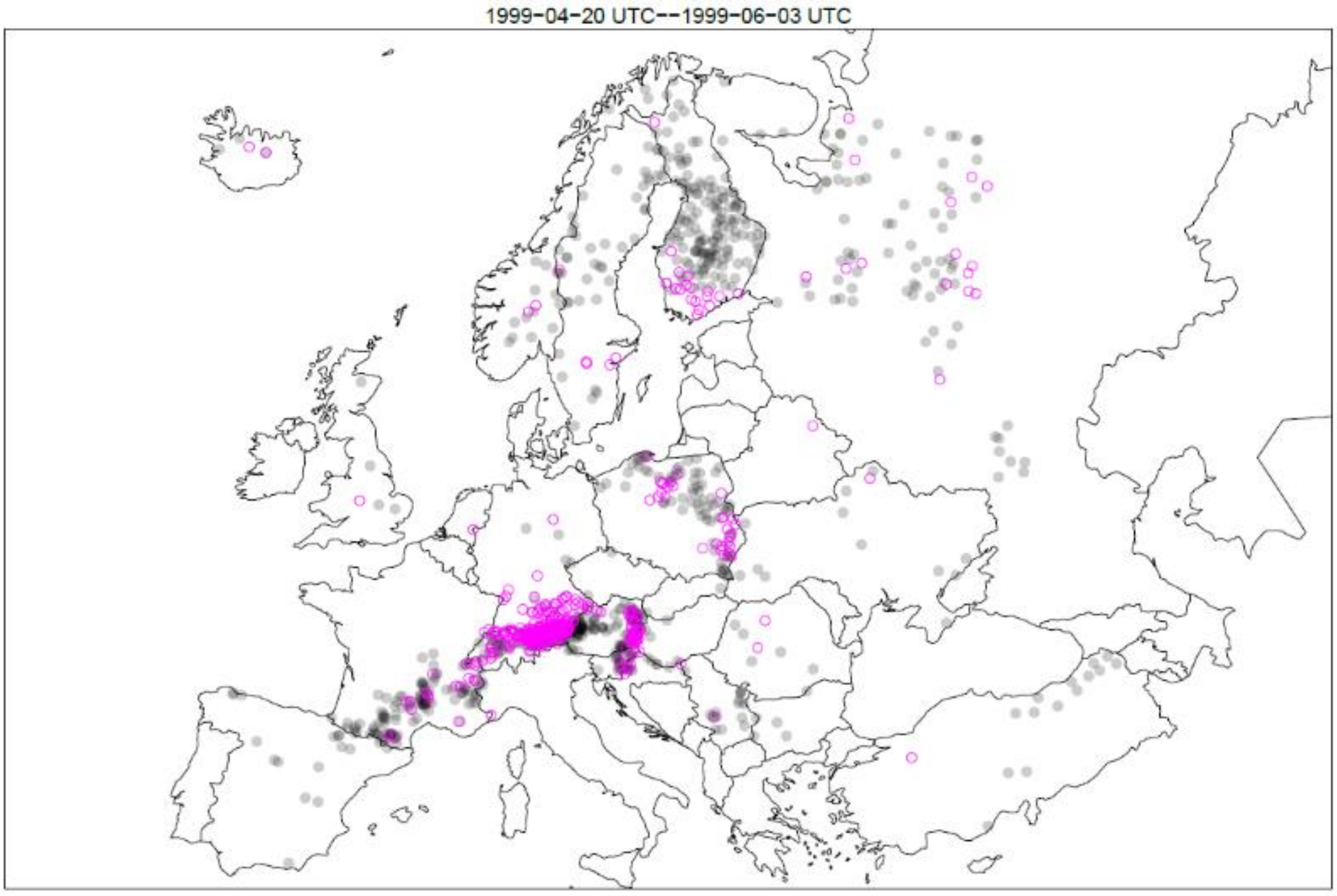

Figure 4.14.1 Floods exceeding the 5-year return period limit and/or with annual maximum reached in May 1999 in Europe, based on the Pan-European flood database.[1] Note that we chose broader time window for display in order to capture also the floods that could be ongoing floods when the major flood event started

Although other years with Lake Constance floods (1975, 1966, 1970 …. 2013, 2019 etc.) also show some partial similarities, 1999 was the case with the greatest (extraordinary) flood magnitude in the 1960-2020 period, and this was also the year when, just like in 1343, preceding high water/flood levels (though due to different reasons) significantly influenced the extraordinary height of the flood peak. The flood lasted rather long (also accounting with the period between Jacob and Bartholomew), for weeks in both cases, with multiple flood waves when the flood peaks were built on each other, as it probably it happened in 1343 when the water stayed for weeks, and it increased gradually even during its extraordinary water levels – Johann von Winterthur provides a rather detailed account of that in Lindau.[56]

As Lake Constance and the High-Rhine floods usually occur between May and August, the late August timing of the Bartholomew Flood (or late July–early August for Jacob Flood) in 1343 was not unusual. Why this flood was truly unusual is the outstanding/extraordinary magnitude and duration (especially if we consider it as a direct continuation of the Jacob Flood). Although the water levels of Lake Constance did not decrease much after Jacob Flood, contemporary sources on the Lake Constance and High-/Upper-Rhine floods consequently refer to them as two (separate/individual) floods, and also the long time-difference (one month) makes it reasonable to discuss the Jacob and the Bartholomew Floods as two separate flood events. While in case of the Jacob Flood both the spatial extension of rainfall and flood reports as well as modern parallels make it possible that the flood was primarily caused by a Vb event, based on the available documentation, the Bartholomew Flood seems to be the result of a major convective event or storm of perhaps more localized nature. However, in neither of these cases we can exclude the possibility of other (Vb, storm, other convective etc.) flood generation types.

4.15 Event 15) Mid-September 1343

Floods: Central Europe (Lake Constance tributaries, probably also Central Transylvania)

Causes (documented): incessant rains/heavy rainfall

Reported in Lindau, the mid-September 1343 great flash flood of Lake Constance tributaries (South-Germany, North-Switzerland, West-Austria) was caused by a week of incessant rains (Supplementary Data 1). A day before, the danger of flooding (i.e., flood-related danger of losing/damage an important charter during travelling) was mentioned in Central Transylvania (probably meaning that either there was already flood or there was a rather high risk for flooding, for example, because of great rains).[16,316] In September, a high intensity storm with westerly, south-westerly winds were also reported in Algeciras (Supplementary Data 2); however, the exact date of this storm is unknown. Based on the available information, the flood or floods could be caused by a storm/convective event (could even be a Vb, too). Together with the Transylvanian charter reference, flood generation and the affected area may show certain similarities to the previous summer floods (*Events 13*, *14*), although possibly with a more limited spatial extent.

As for the weather in East-Anglia, according to Merle,[158] on 10 to 12 (JC: 2-4) September there was serenity and heat, and from 12 onwards for more than a week there was great fog and occasional light rain. Winds only arrived from 28 (JC: 20) September onwards. In general, Merle complained about the scarcity of winds in September. The hot, "silent" and then foggy, windstill weather shows thought-provoking similarities to a phenomenon called "anticyclonic gloom",[317] suggesting a (blocking) high pressure system over (East-)Anglia and/or the North Sea.

In the period 1960-2020, a potential close parallel might be the (mid-/late) September 1984 floods: these floods affected Central and Northeast-Switzerland, South-Germany, South-Poland, the eastern alpine area and parts of the Carpathian Basin, especially its northern, north-western and north-eastern parts (see *Fig. 4.15*). The flood was moderate and magnitude, and only in some areas it reached a higher intensity (based on the Pan-European Flood Database).[1] Because the September 1984 floods were mainly clustered around the southern parts of Central Europe (including the Alps and the northern part of the Carpathian Basin), we chose this area for the comparison with the September 1343 event.

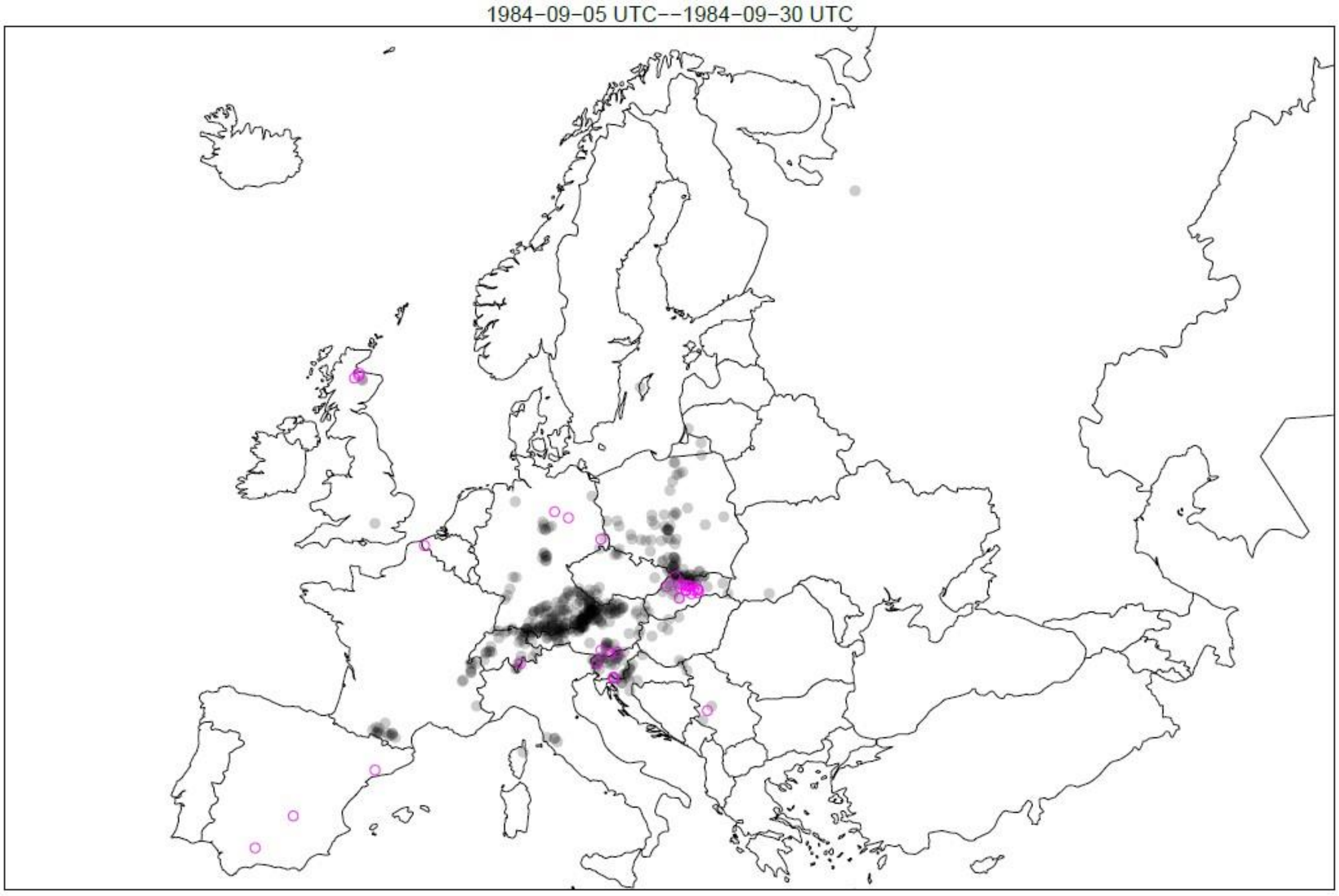

Figure 4.15 Floods exceeding the 5-year return period limit (magenta circle) and/or with annual maximum (grey dot) reached in September 1984 in Europe, based on the Pan-European Flood Database[1]

Even if no important flood was observed in Transylvania in September 1984, the month was rather rich in precipitation: the number of days with notable or great precipitation was double than usual, and most of it was showers/heavy rains and thunderstorms. The year of 1984 itself was also concluded as rainy, but – after a very rainy May, the summer was with normal or slightly less-than-average precipitation; the August was not entirely rainless, but still relatively dry.[318-320] In September 1984, Central European weather conditions were notably influenced by anomalous high sea surface temperatures (with high pressure conditions) over the eastern North Atlantic (to some extent also including the British Isles and the parts of the North Sea), while similarly higher (near-)surface temperature and high-pressure conditions in southern part of Eastern Europe, extending to the Carpathians (but not beyond).[321] Regarding spatial extent and flood generation based on the Pan-European Flood Database,[1] other events, such as the early June 1965 flood or the late August 2005 floods, may also show some relevant parallels.

4.16 Event 16) October 1343

Floods: Guadarranque (SE-Spain), Uh/Už/Ung (NE-Carpathian Basin)

Causes: heavy rainfall (without documentation, same can be presumed regarding the Uh/Už/Ung flood)

Floods of the Guadarranque in Southeast-Spain (6 October) and the Uh/Už/Ung River (14 October: Supplementary Data 1a), an important tributary of the Upper Tisza in the Northeast Carpathian Basin (today W-Ukraine and E-Slovakia)[16] in early/mid-October 1343. As the information available only suggest an "at least" magnitude in both cases, the flood events could as well be greater (than index 1) in magnitude. In the meantime, significant (sea) storm was reported in South-/Southeast Spain (8 October; suggesting low pressure conditions in the area), and dry weather conditions prevailed in Southeast-England (possibly related to predominant high-pressure conditions).

Based on the Pan-European Flood Database,[1] the flood patterns resulting in almost simultaneous flooding in the Gibraltar Bay and the Northeast-Carpathian Basin (and the Carpathians) usually happen in winter or spring (e.g., March 2006, December 2009–January 2010). In the period 1960-2020, the flood showing the most similarities also in seasonality happened in November 1998, when a medium (to high) intensity event occurred covering most of Central Europe, parts of Western Europe and also parts of the Northern Mediterranean (*Fig. 4.16*).[322] The flood on the River Uh/Už in early November developed very quickly, and it was great in magnitude,[323] while in the Transcarpathian Region of Ukraine, where – after a wet summer and September (with heavy precipitation in early and mid-September), a late October heavy rainfall (and a minor flood wave) – heavy precipitation in early November caused simultaneous floods of the Tisza tributaries; these floods were of disastrous magnitude.[324,325]

In November 1998 not only the Uh/Už (and the Bodrog) but other northern tributaries of the Upper-Tisza and also the Upper-Tisza were in flood, and the great Tisza flood was in fact primarily caused by these tributaries.[326,327] Using the November 1998 flood case (and other) parallel(s), and also accounting with the fact that the area affected by the flood/inundation was located between the Uh/Už and the Tisza, it is rather possible that in October 1343 the Tisza was also in flood (as it is only a possibility, we did not include the Tisza in the flood analysis). An important difference in 1998 compared to October 1343 is that in the Iberian Peninsula significant floods developed only in Northern Spain.

On the basis of the Pan-European Flood Database,[1] other relatively similar case occurred in October 1990: this time the magnitude of the Uh/Už flood was notably smaller than it could be in 1343 (or it was in November 1998), while the autumn (including October) was rather stormy with small floods in the Gulf of Gibraltar and the Guadarranque catchment.[328] In October 1990 mostly notable floods were reported in Europe, and more important floods only occurred in the northern part of the British Isles and the south-eastern Alps. A third possibility is 1960 when, in October, annual maxima floods generally occurred in the Northeast Carpathian Basin as well as in Southeast-Spain; however, in this case the flood magnitudes were probably much lower and only the broader areas (and not exactly the same rivers) were affected by notable floods.

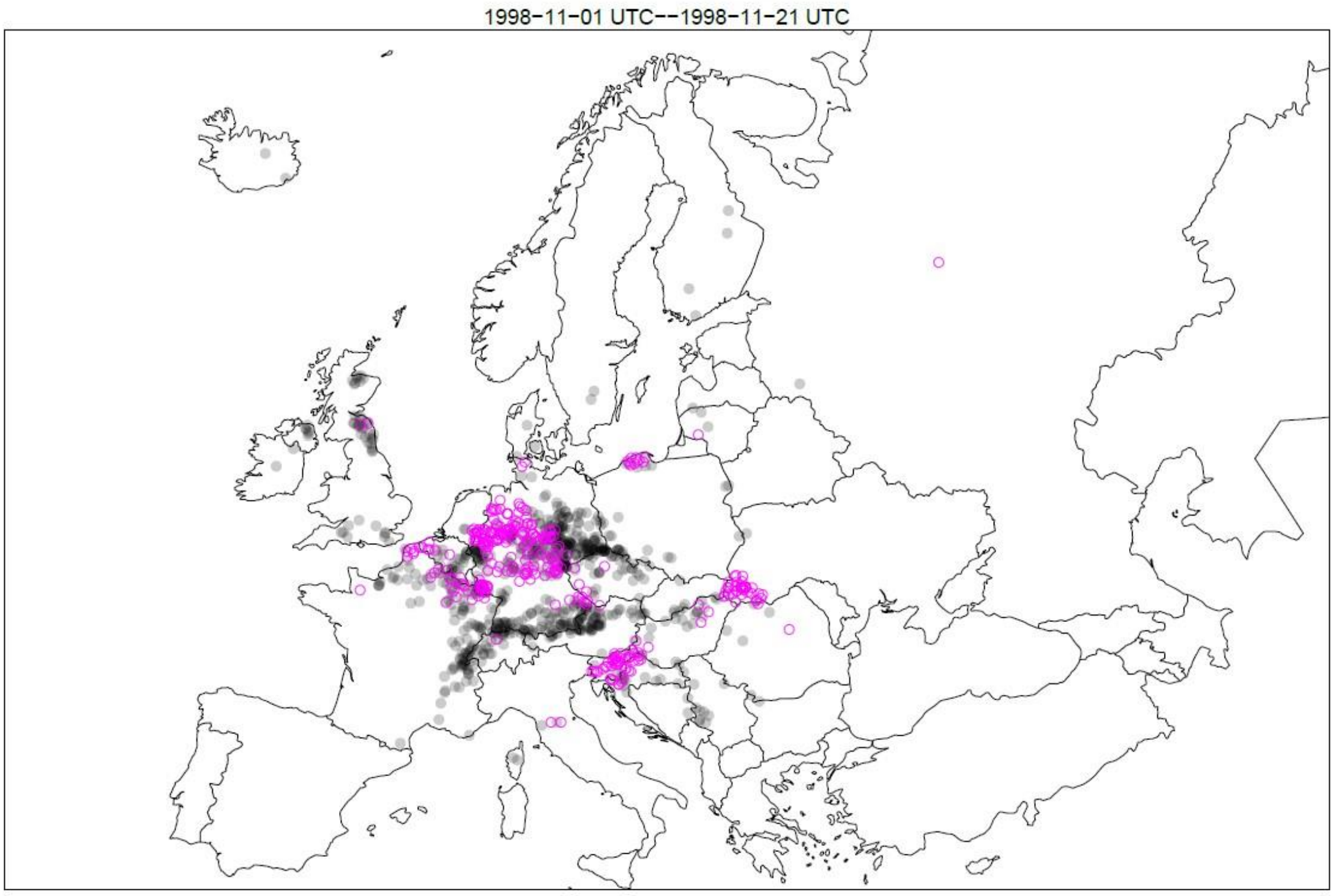

Figure 4.16. Floods exceeding the 5-year return period limit (magenta dots) and/or with annual maximum (grey dots) reached between November 1998 in Europe, based on the Pan-European Flood Database[1]

In November 1998, the floods affected Western and Central Europe from the British Isles to Ukraine and North-Italy. In Spain, more important regional flooding is only detectable in the north (e.g., Upper-Ebro catchment), while in the Gibraltar Bay a minor event (and a gust with rainfall) occurred in November (for daily descriptive weather observations/reports, precipitation and wind observations/records).[329] In terms of near-surface and sea-surface temperatures, a rather unique situation developed in November 1998 that potentially could also show similarities to the 1343 situation (i.e., high sea-surface temperature over the North-Atlantic, warmer-than-usual temperatures in Greenland). Except for a narrow corridor along north-western coast of Africa, over practically the entire North-Atlantic anomalous high (i.e., compared to 1991-2020 means) sea surface (and also near-surface air) temperatures developed extending to Greenland and even beyond in the north. Notably warmer than usually sea-surface and near-surface air temperature conditions prevailed in the Middle East and over the Black Sea. On the other hand, anomalous low air and sea-surface temperatures were observed in most of the Mediterranean.[330] These conditions altogether might have supported the development of stronger westerlies with excess precipitation over most of the Mediterranean and also in Central Europe. In early November, the cold front of the cyclone – arriving from the British Isles – extended from the Ukrainian Carpathians to the (northern part of the) Iberian Peninsula, and wet air masses from the west-southwest (Westlage) caused excessive rainfall, mainly in the hilly/mountainous areas.[331,332]

Based on parallel examples from 1960-2020, the Algeciras case in October 1343 could be part of a more extensive European flood event and/or to some extent could be also related to enhanced Mediterranean cyclonic activities. Based on modern parallels, it would be difficult to find a direct connection between the two floods as, for example, the November 1998 floods affected different part of the Iberian Peninsula. However, one of the (related) possibilities for

flood generation in South-/Southwest Spain is that the flood could be related to, for example, a stationary cut-off low pressure system along the south-eastern coast of the Iberian Peninsula as happened, for example, in Valencia in October 2024[333] (yet in that year without any floods in the Carpathian Basin), causing local rainfall and flood extremes. Such a pattern would fit the general October 1343 picture, as the Guadarranque is also a short, smaller-sized river that was mainly hit also, for example, in October 2024.

5 Volcanic eruptions and Arctic Sea Ice melting. Potential atmospheric and weather-related signatures, and global climate indices

5.1 Documentary sources and databases of volcanic eruptions (1340-1343)

Medieval source text quotations (contemporary; *non-contemporary*), mentioning Icelandic eruptions in (1340-)1341:

(1) Skálholtsannáll (p. 209):[345] "(1341) … Elldz vpp quama inn seta í **Heklu** felli einni nótt eptir festum Dunstani. Með sra miklu myrkri af ȯsku fallinu at í sumum stȯðum fra dagmalum til nóns sa ekki skrím vti helldr enn menn væri blindir. ok var þ mikit myrkr allan daginn ok marga aðra siðan tok askan í aukla undir **Eyia fiȯllum**. ok fylgði naut fellir micill. Annarr elldr var vppi í **Hnappar vallar iȯkli**. hinn priði í **Herði breið yfir Fliotzdals** [Fljótsdalur] heraði ok voru allir jafnsnemma vuppi. …"

(2) Annálsbrot frá skálholti (p. 222):[345] "(1341) … Ellz upp kuama i **Heklu** felli med myklu sanndfalli ok sua storum brestum at biorgum laust saman i elldinum at naliga heydri um allt land sua uar ok dimt medan sanndfallid stod mest yfir. at eigi uar bok liost i kirkium þeim er næst stodu uppkuamu ellzins. hallæri mikid. mikill fiarfellir bædi sauda ok nauta sua at midil fardaga ok Peturs messo fiell at eins fyrir Skalhollti lxxx nauta. …"

(3) Konungsannáll (p. 155):[345] "(1341) … Kom upp elldr i **Heklufelli** med oari oc osku falli oc eyddwz margar bygdir. Myrkr svo mikit um daga, sem um naetr a vetr. …"

(4) Lögmansannál (p. 273):[345] "(1341) … Þa kom vpp elldr j **Heklufelle** med sua miklu sannfalle at fenadr do af vm varit. ok saudfe ok nautfenadr do mest vm Rangar vollu. ok eydde naligha .v. reppa. ok vidara annarstadar do naut manna af sandinum. …"

(5) Gottskállskannál (pp. 351–352):[345] "*(1341) ... Ellds vpp kuoma jn vj j Heklu felli med suo storum dy[n]kium at landit skalf allt suo at j fiarlægum [stodvm og] hierudum hristis skiar æ husvm sem fyrir vindi hvossvm vm langa tima og var þa kyrt vedur. Myrkr var svo micit af oskv falli j nalægum hiervdum at lios brann j hvsvm vm daga. Þetta var fyrir Vrbanus messo vj nottvm. Af þessv osku falli do micill hludi naut fiar fyrir svnnan land svo at marger men vrdu snavder at kuikfie og gengu vr busvm fra eignvm og jordum [sijnum]. eyddvst marger bæer vm Skalhollts sueit og Rang ar vollu og nockrer austar. ...*"

(6) Oddsson:[346] "*Anno 1340 et duobus conseqventibus sexto [rather: secto? - i.e. in the meaning of 'secuto'] coepit ardere mons **Hekla** horrendo fremitus. Item mons **Lomagnúpur**; simul et alter qvidam mons **Trolladingia** exoneravit se usqve ad mare apud provinciam maritimam, qvae appellatur **Selvogur**. Item promontorium **Reykianes** plus dimidio igne consumptum, cujus vestigia in aperto mari etiamnum conspiciuntur, excelsi scopuli exinde dicti **Elldeyiar** sive ut veteres volunt **Driftar** steirn. Item **Geirfugla** skier, ubi lapides plurimi adhuc conspiciuntur exusti. Eodem tempore exarsit etiam mons meridionalis Islandiaa **Sijdu Jokull et multi alij montes** integras provincias devastantes, aliqvando etiam in mari ignis et incendium fuit con spicuum, et montes exusti a continente in mare corruerunt. Tantae mutationes tunc temporis visae sunt in Islandia incomparabile detrimentum insulae adferentes, qvantae nunqvam antea posthac nunqvam resarciendae.*"

Table 5.1 Volcanic eruptions in 1340-1341(-1345). Eruption shading: blue=identified in ice cores (with sulfur load); green=based on contemporary written sources; brown=eruptions with higher uncertainties. Text colours: black=Northern Hemispheric extratropical; grey=tropical eruptions; brown=early 17th century source. Note that, because of the multidecadal dating uncertainties, several other eruptions, listed by the Smithsonian Global Volcanism Program,[21] could potentially also happen in 1340, 1341 or 1342 (e.g. El Chichón, VEI 5: 1360±100y – Smithsonian 10608; or the S. Guadeloupe, VEI 4: 1370±150y – Smithsonian 12358).[21] However, no tropical eruption is noted.[20,340]

**Eruptions**	**VEI** (Smith-sonian)	**Dating** (uncertainty)	**Atmospheric & other consequences reported**	**Documentary source** (contem-porary, *non-cont.)* / **References**
**(Undefined) NH extratropical**	3 (explosive)	1341 (±1-5y?; ice core, dendrocl. data)	Ice core sulfur load: NH: 7.8 kg/km^2 (1.78 Tg)	1341[20,340]
**(Undefined) tropical** (and/or SH)	4 (explosive)	1345 (±1-5y; i.c.+t.) 1343 (ice c.+tree-r.) 1341 (ice c.+tree-r.)	(1) Ice core sulfur load: NH: 27.9, SH: 19.1 kg/km^2; (2) st.sulf. aero-sol inj.: NH:5.62 Tg, SH:25.52 Tg	(1) 1344 or 1345;[20] 1343[24] (2) 1341[337]
**Asosan** (Japan)	2	1340 (3, 10, 25 Feb; doc. source dating)	Only eruption dates mentioned in historical source	[347] Smithsonian 282110[21]
**Hekla** (Iceland)	3 or 4 (explosive)	From 27 (Gregorian Calendar) May 1341 (end unknown, but lasted at least some for days; mentioned by 4 contemporary and 2 non-contemporary sources)	Loud bang & crashes, great storm (or: thunderbolts), *houses shook even in distant places*; mountains destroyed by fire; stone fall. Half-day full darkness, then many days of winter-night like darkness; da-kest when ashfall most intensive. Great dearth and loss of cattle & sheep; worst in Skálholt (80 cattle died in 1 June–7 July) & Rangár-valla: S,SW-Iceland).Many settle-ments destroyed, churches at the mountains were left. *People fled, left their lands & properties. Entire provinces devastated; so many unrepairable losses in Iceland as never before or after.*	Contemporary sources: Skálholtsannáll (1; eruptions preceded by outstanding snow-rich & long winter), Annálsbrot frá skálholti (2), Konungsannáll (3), Lögmansannáll (4);[345] *Later (non-contemporary) sources: Gottskállskannál (5: 16th century), Oddsson[347] (6: early 17th century – altogether mentioned in relation to the Hekla eruption and the many other Icelandic – mainly southern – eruptions in 1340-1342)* / VEI 3 (tephra-chronology),[25] Smithsonian 372070;[21] VEI 4 (tree-ring, documentary, tephra etc.)[26]
**Eyjafjallajökull** (Iceland)	?	Same time as Hekla (1 contemp. source)	mass loss of livestock mentioned right after (H.+) E. eruption ref.	Skálholtsannáll (1)[345]
Öræfajökull (Hnap-pafellsjökull; **Grimsvötn** volc.?)	?	Same time as Hekla (1 contemporary source)	Only the (app.) timing of eruption mentioned (by contemporary source)	Skálholtsannáll (1)[345] Inactive volcano under Öræfajökull; eruption of **Grimsvötn** is much more likely
Herðubreið (near **Trölladyngja** &**Askja** volcanoes)	?	Same time as Hekla (1 contemp. source)	Only the (app.) timing of eruption mentioned (by contemporary source)	Skálholtsannáll (1)[345] Herðubreið is a tuya; could mean the fissures of the **Trölladyngja** or the **Askja** nearby
**Krýsuvik(?)-Tröl-ladyngja** (Iceland; Reykjanes penins.?)	1	1340? *(and/or in the following 2 years)*	eruption (documented in early 17th century; +tephrachronology with large dating uncertainty)	*Oddsson (6)*: means K.-T. in Reyk-janes or T., mentioned by Skálholts-annáll / Smithsonian 371030[21]
**Eldey** + ***Reykjanes headland*** *(volcanic system; Iceland)*	3 (explosive; *at least partly* underwater)	1340? *(and/or in the following 2 years)*	*over half of the Reykjanes (peninsula) consumed by fire (partly lost in sea)*[6]	*Oddsson (6)* / [348] (based on *Oddson*), Smithsonian 371022[21]
**Brennisteinsfjöll** 'mts. of Selvogur'?	2 (effusive; explosive?)	1341 (±1y)	Part of general impact infor-mation presented by *Oddsson*	*Oddsson (6)*: nearest volcano to the Selvogur area /Smithsonian 371040[21]
**Grímsvötn** (Iceland – *Oddsson*'s Lómagnúpur)	2 (effusive; explosive?)	May 1341 – *1340 (and/or in the following 2 years)*	*Great destruction (south), entire provinces devastated, 'neve-seen' unrepairable changes in Iceland*	*Oddsson (6)*. L. is not volcanic, but Skeiðarárjökull nearby originates from G. / Smithsonian 373010[21]
Siðujökull & 'other mountains in the south' (**Grímsvötn & southern v.-s?**)	*? (high intensity, with irreversible changes)*	*1340 (and/or in the following 2 years)*		*Oddsson (6)*; **Siðujökull** is not a volcano, but Oddsson could gene-rally mean indirect consequences of series of eruptions
Hakkodasan (Japan)	1	1340 (±75y)	(^{14}C-based dating)	Smithsonian 283280[21] – small
Kikai (Japan)	?	1340 (±30y)	(^{14}C-based dating)	Smithsonian 282060[21] – uncertain
**Pelée** (France)	4 (explosive)	1340/1348(±50y)	(^{14}C-based dating)	[349,350] Smithsonian 360120[21]
P.d.l. Fournaise (F)	0?	1340?	(^{14}C-based dating)	Smithsonian 233020[21] – uncertain
Kilauea (USA)	0	1340 (±40y)	(anthropology dating)	Smithsonian 332010[21] – uncertain
Pagan (USA)	?	1340 (±100y)	(^{14}C-based dating)	Smithsonian 284170[21] – uncertain
Baru (Panama)	?	1340 (±75y)	(tephrachronology)	Smithsonian 346010[21] – uncertain

5.2 Global climate and its response to volcanic eruptions and Arctic sea ice decrease

Table 5.2 Potential signs of climate response to (tropical, extratropical) volcanic eruptions and sea ice reduction

Response types in the late 1330s, 1340, 1341, 1342, 1343 over majority of Europe	Typical response for tropical eruption	Typical response for NH extra-tropical eruption	Typical response for Arctic Sea ice reduction
Late 1330s–early 1340s (incl. 1342, 1343): predominant milder winters in Greenland & Franz Josef Land (Suppl. Data 2b)[32]		+ (1342-1343: annual Greenland blocking; see Suppl. Data 2b)	+ [41,32]
Late 1330s–early 1340s: increased sea surface temp. in the North Atlantic (Suppl. Data 2b)			+ [37]
Outstanding mild winter 1340 in Iceland (Skálholtsannáll: Suppl. Data 2a)			(comb. with NAO+, stronger Golf Stream, and change in jet stream; or consequence of AD–/AO+)[351]
Very snowy, long winter in S-Iceland, 1341 (e.g. Skálholtsannáll: Suppl. Data 2a)			+ (esp. in combination with NAO+; and/or consequence of Arctic dipole (AD+)[351,352]
Great northward shift/expansion of the Hadley cell in 1341[334]	+ (poleward shift of the North Atlantic Jet Stream – only in case of winter eruption)[353]	(e.g. southern hemisphere extratropical eruption: ITCZ and Hadley cell northward shift)[33,354]	+ (a potential impact)[39]
Warm, dry summer in Western and Central Europe in 1341[246,249,312]		+ (e.g. Laki eruption: summer 1783)[342]	
Cold(er 1st & 2nd) Eu. winter (current paper)		+ [355] (Laki 1783/84)[342]	+ [356]
Wet winter in (Central) Europe (current paper)	+ (Tambora 1815/16)[357]	+ [355] (Laki 1783/84)[342]	+ [356]
Cool summer in (W. & Central) Europe and in the Northern Hemisphere (PAGES 2k data)[20,249]		+ [27] (but: 1783, 1784 warmer summers) [342]	
Wet summer(s) in Europe (current paper)			+ [356]
Hadley-cell contraction/southward shift[71334]; or maybe related to ENSO mode	+ (contradiction btw. opinions)[334,354]	+ (southward shift)[71,354]	+ [39]
"El-Nino like" 1st (and less or neutral 2nd) year after eruption: from – to + in 1342 (+1343)[358,359]	+ (NH tropical eruption)[35,360]	+ [361]	(may decrease the magnitude of ENSO anomaly)[362]
Weak monsoon in NE India,[48] combined effect of ENSO and Indian Ocean Dipole?[363]	+ [27] (primary through ITCZ contraction)[48]	+ (ITCZ southward shift)[48,350,364]	+ (indirect relationship)[356] (primary through ITCZ shift)[48]
Normal/high Nile flood level (summer),[365] suggesting ENSO-[366] or late start of El Nino[367]	+ [364] (northward shift of the ITCZ)[368]		+ (responsible for multiannual positive Indian Ocean Dipole)[369]
Weakened, wavy jet stream (north-Rossby wave forcing); more persistent waves, ridges, increased mid-latitude storminess, floods (current paper)		+ [27]	+ [40,370,371]
Negative winter NAO- (see current paper)		+ [27]	+ [271]
Positive summer NAO+ (see current paper)	+ [27] (and the Laki)[372]		+ [40]
2nd-year (summer) flood response in Central Eu.: Bodensee, High-Rhine (1343-current paper)	+ (e.g., 1816-1817)[305]		
Greenland winter warming (Suppl. Data 2b)		+ [27]	+ [356]
Barents Sea (Franz Josef Land) cold winter(s), warm summer(s)[339]		+ (e.g., 1784, first winter after Laki eruption)[339]	
(change to) AO- (current paper)		+ [27]	+ [370]
extreme weather signs of sudden stratospheric warming: cold inbreaks in Eurasia, Central, S- and W-Eu (Suppl. Data 2a,b)		+ [373,374]	
extreme cold summer in Yamal, Siberia: (one of the 3 coldest summers in 1200-1500,[376] apart from 1259 (after Samalas) and 1453 (Kuwae e.)	+ [375]	+ [376]	+[27]
Rainy summer 1343 in Iceland (Suppl. Data 2a)			+ [377]
Yucatan, Guatemala, New Mexico droughts in early 1340s[49,50]	+ (major tr. e. often cause Y. drought)[370]	+ (ITCZ southward shift, ENSO reduction)[378]	+ [379-381]
South-west-Central region of the USA (incl. Arkansas) intense hydroclimate regime shifts[51]		+ [382]	+ (general links suggested; no direct connection proved)[383,384]
Increased precipitation (inducing floods) in the Yangtse river basin[45-47]	+ (through intensified E-Asian monsoon)[385]		+ correlation btw. Barents/Kara sea-ice decrease and Yangtse Basin precipitation[386,387]

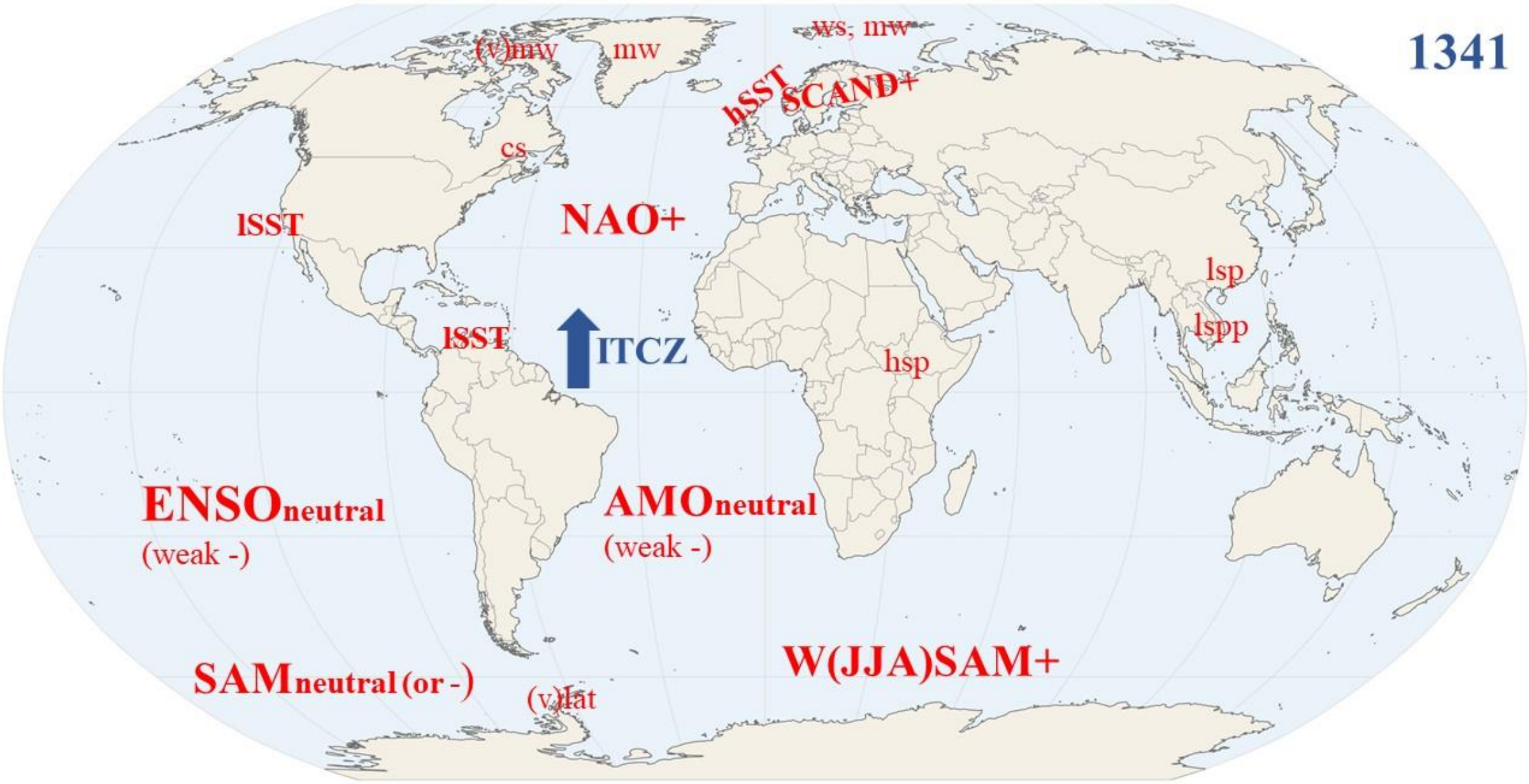

Figure 5.2.1 Overview of prevailing climate mode in 1341 (base map[388] with regional and global climate indices). For background information, see "List of abbreviations and references" below

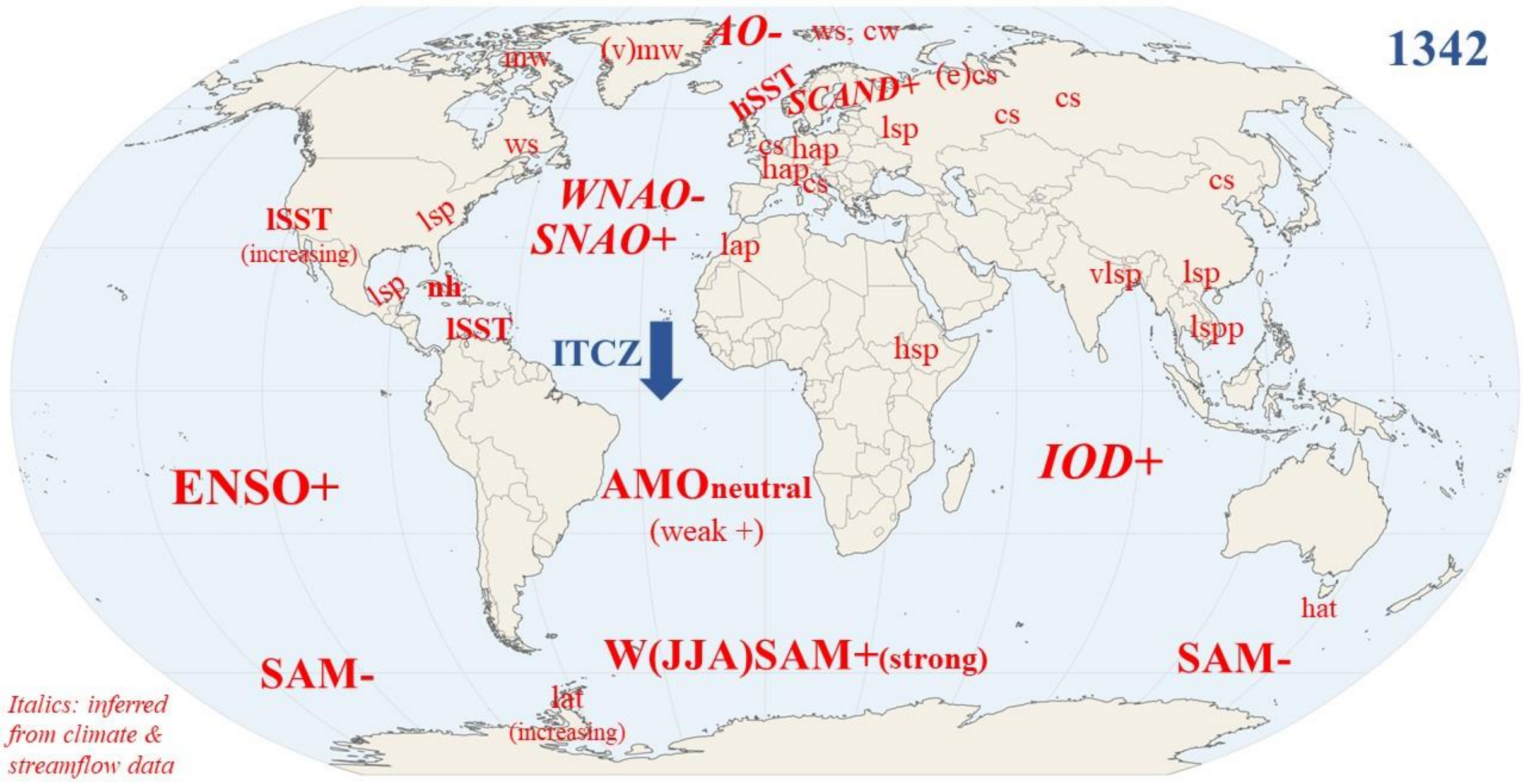

Figure 5.2.2 Overview of prevailing climate mode in 1342 (base map[388] with regional and global climate indices). For background information, see "List of abbreviations and references" below

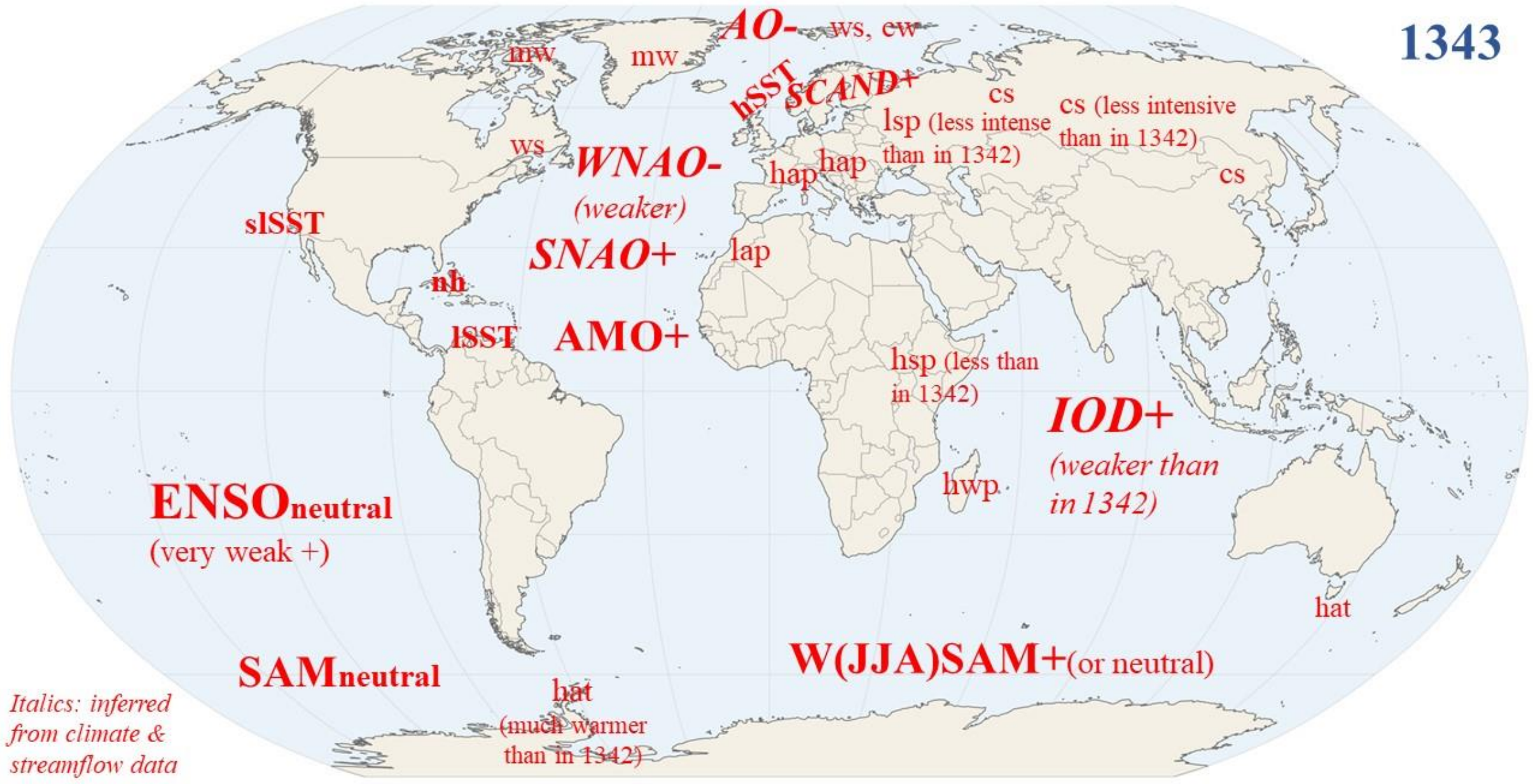

Figure 5.2.3 Overview of prevailing climate mode in 1343 (base map[388] with regional and global climate indices). For background information, see "List of abbreviations and references" below

List of abbreviations and references to Supplementary Information *Figure 5.2.1–3*:

hat=high annual temperature, (v)lat=(very) low annual temperature, ws=warm summer, mw=mild winter, cw=cold winter, (e)cs=(extreme) cool/cold summer.[375,389]

lsp=low summer precipitation, hsp=high summer precipitation, lspp=low spring (monsoon) precipitation, hwp=high winter precipitation, hap=high annual precipitation, lap=low annual precipitation.[46,246,250,312,390]

hSST=(slightly) high sea surface temperature (NE-Atlantic),[38] lSST=(slightly) low sea surface temperature (Venezuela, California).[391,392]

nh=no hurricane (in Bahamas).[393]

AO=Arctic Oscillation (index). See Methods chapter

WNAO=Winter North Atlantic Oscillation (index). See Methods chapter

SNAO=Summer North Atlantic Oscillation (index). See Methods chapter

SCAND=Scandinavian blocking (index), derived from low summer precipitation, low cloud cover and low winter/high summer temperatures (in 1342, 1343: Supplementary Data 2a,b)

AMO=Atlantic Multidecadal Oscillation (index).[394]

ENSO=El Nino Southern Oscillation (index).[358,359]

IOD=Indian Ocean Dipole (index), derived from rel. high Nile flood level, (very) low Brahmaputra discharge and low SE-China summer precipitation.[48,312,365,395]

SAM=Annual Southern Annular Mode (index; neutral SAM for 1341); negative SAM value was suggested based on very low temperatures in James Ross Peninsula, and rel. high temperatures in Tasmania in 1341.[358]

WSAM=Winter Southern Annular Mode (index).[358]
